# Landscape Population Genomics of Forsythia (*Forsythia suspensa*) Reveal That Ecological Habitats Determine the Adaptive Evolution of Species

**DOI:** 10.3389/fpls.2017.00481

**Published:** 2017-04-05

**Authors:** Jie Yang, Cai-Yun Miao, Run-Li Mao, Yong Li

**Affiliations:** Department of Garden, College of Forestry, Henan Agricultural UniversityZhengzhou, China

**Keywords:** adaptation, ecological habitat, environment-associated loci, *Forsythia suspensa*, warm temperate zone

## Abstract

Understanding the genetic mechanisms of adaptation to environmental variables is a key concern in molecular ecology and evolutionary biology. Determining the adaptive evolutionary direction and evaluating the adaptation status of species can improve our understanding of these mechanisms. In this study, we sampled 20 populations of *Forsythia suspensa* to infer the relationship between environmental variables and adaptive genetic variations. Population structure analysis revealed that four genetic groups of *F. suspensa* exist resulting from divergent selection driven by seven environmental variables. A total of 26 outlier loci were identified by both BayeScan and FDIST2, 23 of which were environment-associated loci (EAL). Environmental association analysis revealed that the environmental variables related to the ecological habitats of *F. suspensa* are associated with high numbers of EAL. Results of EAL characterization in *F. suspensa* are consistent with the hypothesis that ecological habitats determine the adaptive evolution of this species. Moreover, a model of species adaptation to environmental variables was proposed in this study. The adaptation model was used to further evaluate the adaptation status of *F. suspensa* to environmental variables. This study will be useful to help us in understanding the adaptive evolution of species in regions lacking strong selection pressure.

## Introduction

Understanding the genetic mechanisms of adaptation to environmental variables is a key issue in molecular ecology and evolutionary biology. Changes in environmental variables are mainly manifested in climate fluctuations on a time scale and in environmental heterogeneity on a spatial scale. Two adaptation strategies are usually adopted by plants in response to environmental variables. One strategy is adjustment in the distribution, and the other is production of adaptive variations in the genome (Davis and Shaw, 2001; Lei et al., 2015). Plants displaying highly efficient seed dispersal capacity often adopt the first strategy as confirmed by phylogeographical studies (Avise, 2009; Hickerson et al., 2010). Conversely, species lacking effective seed dispersal mechanisms are compelled to adapt to local conditions by adopting the second strategy. Substantial adaptive variations in the genome will eventually lead to changes in phenotype and phenological characteristics of a species (Rellstab et al., 2015). These changes provide the basis for adaptive evolution of a species. Interaction between gene flow and natural selection lead to species adaptation in response to heterogeneous regional environment (Rieseberg and Burke, 2001; Ellstrand, 2014), resulting in spatial variation in adaptive allele frequencies among populations (Manel et al., 2012; Schoville et al., 2012). Visualizing adaptive genetic variability across environmental variation is the key in understanding the adaptive evolution of a species (Hoffmann and Willi, 2008). However, detecting adaptive genetic variation remains a great challenge because of the lack of a priori genomic resources for non-model species (Stinchcombe and Hoekstra, 2008).

Joost et al. (2007) have proposed landscape population genomics, which is a relatively new approach used to reveal the relationships between adaptive genetic and environmental variations (Allendorf et al., 2010; Schoville et al., 2012). However, this approach requires genome-wide molecular markers and high-resolution environmental data (Balkenhol et al., 2009; Epperson et al., 2010). To date, amplified fragment length polymorphisms (AFLPs), inter-simple sequence repeats (ISSRs), and genotyping by sequencing are widely used in landscape population genomics studies (De Kort et al., 2014; Rellstab et al., 2015; Wang et al., 2016). High-resolution environmental data are now available from public climate databases, such as the worldclim database (www.worldclim.org). Recent landscape population genomics studies have highlighted the genetic mechanisms of species adaptation to the environment (Ćalić et al., 2016; Pluess et al., 2016). Statistical approaches have been developed to identify outlier loci showing higher differentiation among populations and lower genetic diversity within populations compared with selectively neutral regions of a genome (Abebe et al., 2015); these approaches include BayeScan based on multinomial-Dirichlet model (Foll and Gaggiotti, 2008) and Arlequin based on hierarchical island model (Excoffier and Lischer, 2010). To further determine whether these outlier loci are driven by environmental factors, researchers have developed multiple statistical approaches for association testing; these approaches include Bayesian linear mixed model (Coop et al., 2010; Günther and Coop, 2013), generalized dissimilarity model (Ferrier et al., 2007), generalized estimating equation (Carl and Kuhn, 2007), latent factor mixed model (Frichot et al., 2013), multiple logistic regression analyses (Grivet et al., 2011), and spatial analyses based on univariate logistic regression (Joost et al., 2007).

Although a large number of adaptive loci driven by environmental variables were identified in more than one species, the general and overall result taken from these landscape population genomics studies in regional vegetation is that the commonality on species adaptation response to identical environmental variables is still unclear. Commonality in adaptive evolution in regional vegetation generally requires extremely strong selection pressure produced by one or more environmental variables. Species exposed to extreme heat and drought in desert areas or to high salinity in the ocean tend to produce convergent adaptive evolution in genes or phenotype (Kültz, 2015; Givnish, 2016). However, most regions do not exhibit strong selection pressure produced by these extreme environments mentioned above. Thus, the direction of adaptive evolution in regional vegetation is usually diverse (Manel et al., 2012; Ćalić et al., 2016). Determining the direction of adaptive evolution of the genome driven by environmental variables is an interesting endeavor. Another interesting concern is the means by which the adaptation status of species in response to environmental variables can be evaluated. Understanding the commonality of this diverse adaptive evolution of regional vegetation is useful in developing suitable conservation and management strategies.

The warm temperate zone in China is located between 32°30′–42°30′ and 103°30′–124°10′ (Shangguan et al., 2009). The typical vegetation in this region consists of deciduous broad-leaved forest (Gao et al., 2001). This region does not exert strong selection pressure brought about by extreme environmental variables. In this study, we sampled *Forsythia suspensa* (Thunb.) Vahl (Oleaceae), a deciduous shrub widespread at 300–2,200 m above sea level in the warm temperate zone in China. The flowering period of *F. suspensa* is from March to June, and the fruiting period is from July to September. This species prefers light and tolerates a certain degree of shade; additionally, it prefers warm and humid climate and can tolerate cold and drought but not waterlogging (Niu et al., 2003). We sampled 20 natural populations of *F. suspensa* in the warm temperate zone in China to infer the relationship between environmental variables and adaptive genetic variations. Start codon targeted (SCoT) polymorphism is a gene-targeted marker, which was developed based on short conserved start codon in plant genes (Collard and Mackill, 2009). SCoT markers are highly variable and reproducible and have been widely used to survey population genetics and phylogenetics (Guo et al., 2014; Feng et al., 2015; Sorkheh et al., 2016). However, SCoT markers have been seldom used in landscape population genomics to detect adaptive loci in genomes.

In this study, we used environmental data and 1,242 loci yielded by SCoT markers to study the adaptive evolution of *F. suspensa* in the warm temperate zone in China. The present study aimed to (i) reveal the spatial genetic structure of *F. suspensa*, (ii) identify the key environmental variables that drive adaptive differentiation in *F. suspensa*, and (iii) evaluate the adaptation status of the species in response to environmental variables.

## Methods

### Sample collection

**Figure 1:**
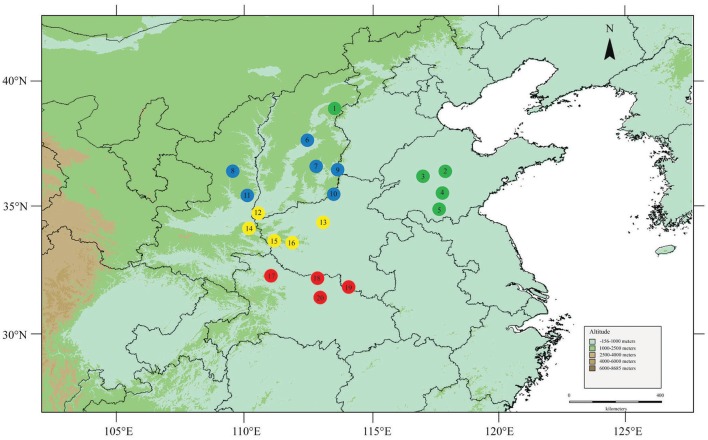
**Locations of the 20 sampled ***F. suspensa*** populations**. Map produced by software DIVA-GIS 7.5.0, URL: http://www.diva-gis.org/download/.

A total of 204 individuals from 20 natural populations were collected from the entire distribution range of *F. suspensa* in China (Figure 1). Most population samples contained 10–12 individuals (Table 1). Although 10–12 individuals of one population might not be an ideal sample size, the sample numbers could meet the needs of genetic analysis to overcome sampling bias (De Kort et al., 2014). All individuals were collected when the population size was <10. Thus, those populations with fewer individuals sufficiently represented their genetic diversity. Fresh leaves were collected and stored in silica gel at room temperature until DNA extraction. Table 1 shows the geographical coordinates of the sampled populations.

**Table 1:** **Details of population locations, sample size, genetic diversity of 20 populations for ***F. suspensa*****.

**Population no. and code**	**Locations**	**Lat. (N)/Long. (E)**	***N***	***N*_A_**	***PPA***	***H*_E_**
1. SXWT	Wutai Mt., Shanxi	39.00/113.58	8	226	18.2	0.061
2. SDYM	Yuan Mt., Shandong	36.47/117.85	10	428	34.5	0.108
3. SDTM	Tai Mt., Shandong	36.25/117.10	12	461	37.1	0.109
4. SDMM	Meng Mt., Shandong	35.50/117.80	12	454	36.6	0.105
5. SDBD	Baodugu, Shandong	35.00/117.70	12	496	39.9	0.110
6. SXTL	Tianlong Mt., Shanxi	37.70/112.43	6	425	34.2	0.137
7. SXLK	Lingkong Mt., Shanxi	36.60/112.83	12	504	40.6	0.109
8. SXBT	Baota Mt., Shaanxi	36.58/109.48	6	340	27.4	0.110
9. HBWZ	Wuzhi Mt., Hebei	36.50/113.65	12	444	35.7	0.101
10. HNJL	Jiulian Mt., Henan	35.58/113.58	10	300	24.2	0.074
11. SXHM	Hua Mt., Shaanxi	35.55/110.10	12	400	32.2	0.090
12. SXWL	Wulaofeng, Shanxi	34.83/110.58	12	419	33.7	0.101
13. HNSM	Song Mt., Henan	34.47/113.08	10	239	19.2	0.060
14. SXLJ	Laojun Mt., Shaanxi	34.33/110.25	12	388	31.2	0.094
15. HNLJ	Laojieling, Henan	33.75/111.33	10	312	25.1	0.077
16. HNLY	Longyuwan, Henan	33.70/111.75	10	298	24.0	0.076
17. HBWD	Wudang Mt., Hubei	32.40/111.00	12	571	46.0	0.136
18. HNTB	Tongbai Mt., Henan	32.38/112.83	10	451	36.3	0.116
19. HNJG	Jigong Mt., Henan	31.83/114.08	10	530	42.7	0.144
20. HBDH	Dahong Mt., Hubei	31.52/112.97	6	649	52.3	0.223

*N_A_, number of polymorphic alleles; PPA, percentage of polymorphic alleles; H_E_, Nei's (1973) measure of gene diversity*.

### Molecular protocols

Genomic DNA was extracted from ~30 mg of dried leaves by using a Plant DNA Extraction Kit DP305 (Tiangen, Beijing, China) according to the manufacturer's recommendations. DNA concentration and quality were measured using a Microcolume Spectrophotometer ND5000 (BioTeke, Beijing, China). After preliminary screening of 36 SCoT primers (Collard and Mackill, 2009), 10 primers (SCoT4, SCoT5, SCoT6, SCoT9, SCoT12, SCoT14, SCoT17, SCoT18, SCoT21, and SCoT31) were selected for polymerase chain reaction (PCR). SCoT4, SCoT5, and SCoT21 were 5′ fluorescent primers labeled with FAM; SCoT9, SCoT17, and SCoT18 were labeled with HEX; SCoT6, SCoT12, SCoT14, and SCoT31 were labeled with TAMRA. PCR amplification was performed in a 20 μL reaction mixture consisting of 20 ng of template DNA, 10 mM reaction buffer (pH 8.3), 0.2 mM dNTPs, 0.3 μM primer, and 1 unit of Taq polymerase (Tiangen, Beijing, China). PCRs were performed in an iCycler gene amplification system (Bioteke, Beijing, China) under the following conditions: initial denaturation for 4 min at 95°C followed by 35 cycles of denaturation for 40 s at 94°C, primer annealing for 45 s at a primer-specific annealing temperature (48°C for SCoT21, 52°C for SCoT4, SCoT5, SCoT6, SCoT9, SCoT12, and SCoT17, 55°C for SCoT31, and 60°C for SCoT14 and SCoT18), extension for 1 min at 72°C with a subsequent extension step for 5 min at 72°C, and termination by a final hold at 4°C. PCR products were mixed with 10 μL of HiDi formamide and separated on an ABI 3730 DNA Analyzer at BGI (Beijing, China). PCR products were sized relative to ROX1000 size standard (Applied Biosystems, Foster City, USA).

### Data analysis

Unambiguous SCoT fragments were scored and transformed into a 1/0 matrix according to the presence or absence of peaks viewed using GeneMarker 2.2.0 (SoftGenetics, State College, Pennsylvania, USA). To reduce the scoring false rate, we scored the peaks within 60–1,000 bp and heights above 500 relative fluorescent units. Subsequent population genetic analyses were performed on the basis of this 1/0 character matrix.

Genetic parameters per population were calculated by AFLPSURV 1.0 (Vekemans, 2002). The estimates included the number of polymorphic alleles (*N*_A_), percentage of polymorphic alleles (*PPA*), gene diversity of Nei (*H*_E_; Nei, 1973), pairwise Nei's unbiased genetic distance (Nei, 1987; Lynch and Milligan, 1994) between populations, and gene frequencies per allele in each population.

Genetic structure of the 20 populations of *F. suspensa* was assessed using the procedures NEIGHBOR and CONSENSE within the program PHYLIP 3.63 (Felsenstein, 2004). Population differentiation was characterized using hierarchical and non-hierarchical analysis of molecular variance (AMOVA) within ARLEQUIN 3.5.1.2 (Excoffier and Lischer, 2010). To infer the contribution of geographical distance to spatial genetic structure, we performed mantel tests of isolation-by-distance (IBD) in IBD 3.23 (Jensen et al., 2005) by regressing the Nei's unbiased genetic distance against geographical distance. In this study, the strong correlated environmental variables (*r* > 0.95) were excluded and the uncorrelated environmental variables were used for further analyses. Auto correlation analysis of environmental variables was performed using Pearson's regression in SPSS 19 (SPSS Inc., Chicago, IL, USA). Ten environmental variables (Bio2, Bio3, Bio4, Bio5, Bio6, Bio8, Bio12, Bio13, Bio15, and Bio17) were identified as uncorrelated environmental variables. To further infer the contribution of environmental variables to spatial genetic structure, we performed redundancy analysis (RDA) by using CANOCO 4.5 (Ter Braak and Smilauer, 2002). In RDA, gene frequencies per allele in each population (Table S1) were used as response variable, and the 10 uncorrelated environmental variables (Tables S2, S3) were used as explanatory variables. Environmental data from 1950 to 2000 at 2.5 arcmin resolution were obtained from the world climate database (http://www.diva-gis.org/climate). Environmental data for each population were extracted using DIVA-GIS 7.5 (Hijmans et al., 2001).

To identify outlier loci, we utilized a Bayesian approach based on the method described by Beaumont and Balding (2004) by using BayeScan 2.1 (Foll and Gaggiotti, 2008). The outliers were calculated using the following parameters: a sample size of 5,000, thinning interval of 10, 20 pilot runs with a run length of 5,000, and additional burn-in of 50,000 iterations. Posterior probability >0.76 corresponding to log_10_-values of the posterior odds (PO) >0.5 were taken as substantial evidence for selection. Thus, the above mentioned loci were regarded as outlier loci. The second approach was based FDIST2 approach proposed by Beaumont and Nichols (1996) by using Arlequin 3.5.1.2 (Excoffier and Lischer, 2010). The outliers were calculated with a hierarchical island model using the following parameters: 100 simulated demes and 20,000 coalescent simulations. To reduce the false discovery rate, loci with minor allele frequency <5% were excluded. The loci outside the 95% confidence interval were regarded as outlier loci. To further test whether these outlier loci are driven by environmental variables, we conducted an environmental association analysis based on latent factor mixed model (LFMM) by using LFMM 1.2 (Frichot et al., 2013). Given that the Bayesian mixed model considers the population structure, this model can avoid the bias caused by population history and isolation by distance and yields a robust result for environment-associated loci (EAL). The association analysis was run using the following parameters: 10,000 sweeps, 1,000 burn-in sweeps, and the number of latent factors used was recommended by PHYLIP. The loci with |*z*| > 3 and *P* < 0.001 were regarded as EAL.

## Results

### Genetic structure

Ten SCoT primers were selected to investigate the population genetic structure of *F. suspensa*. A total of 1,242 unambiguous loci were identified with sizes ranging from 60 to 1,000 bp. The number of loci in 10 primers ranged from 60 (SCoT31) to 161 (SCoT4). The gene diversity of Nei (*H*_E_) at the species level was 0.124. The lowest number of polymorphic alleles (*N*_A_ = 226, *PPA* = 18.2) was exhibited in SXWT (P1) population and the highest (*N*_A_ = 649, *PPA* = 52.3) in HBDH (P20) population. The lowest gene diversity of Nei (*H*_E_ = 0.060) was exhibited in HNSM population and the highest (*H*_E_ = 0.223) in HBDH (P20) population. Overall, Table 1 shows the summary statistics of the genetic diversity analyses of 20 populations of *F. suspensa*.

**Figure 2:**
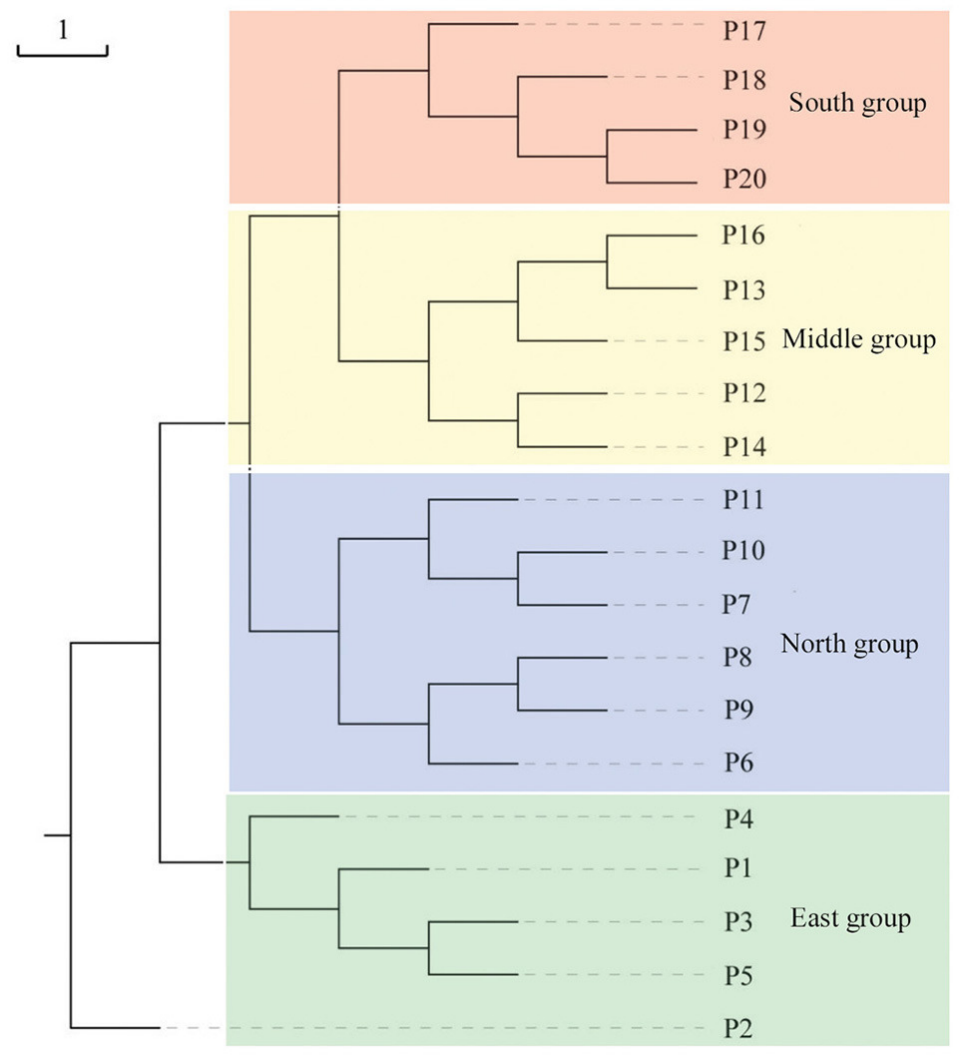
**Neighbor-joining network illustrating the genetic relationships among 20 populations of ***F. suspensa*** based on Nei's (**1987**) unbiased genetic distance**.

**Figure 3:**
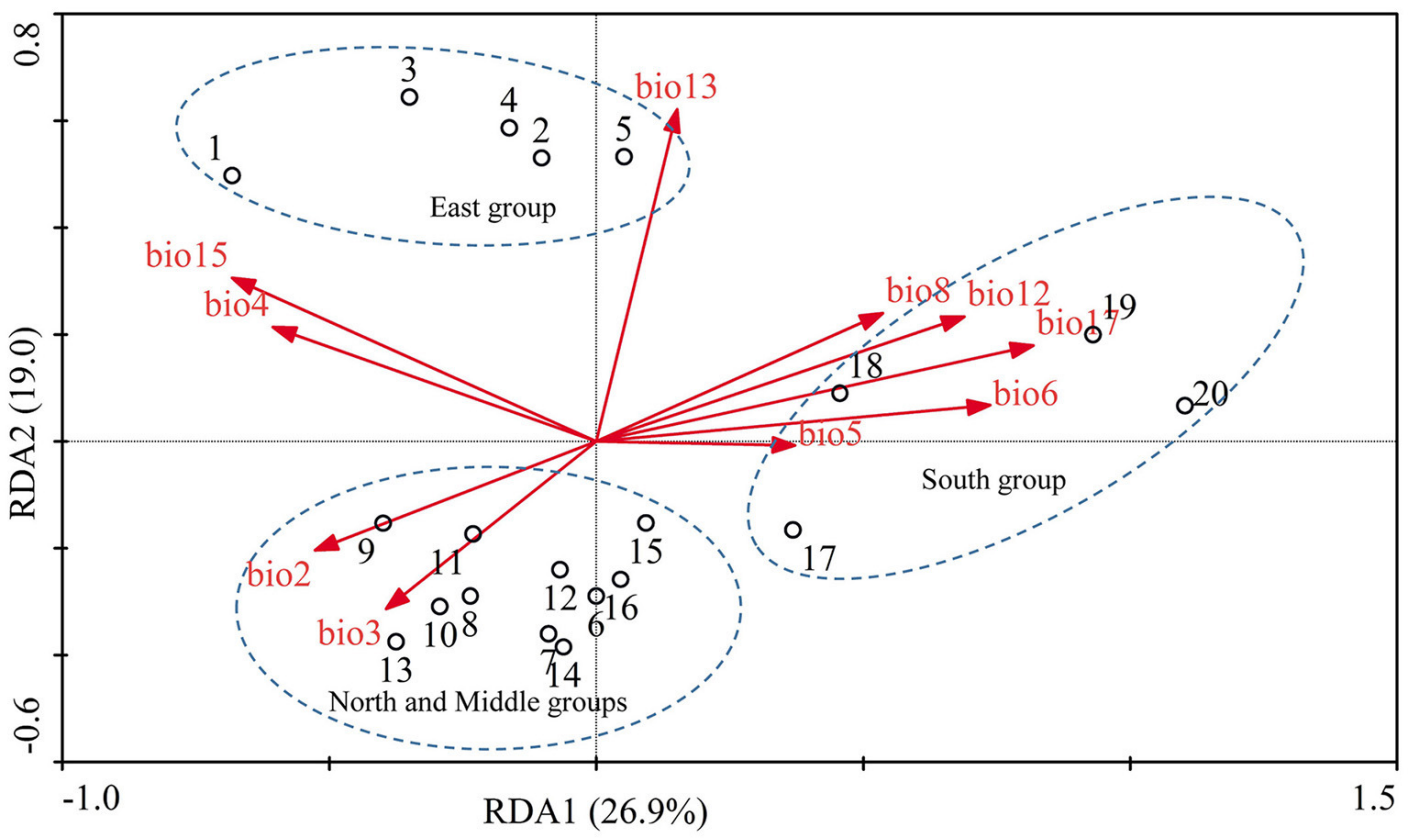
**RDA analysis was performed to determine the relative contribution of environmental variations shaping the genetic structure**. The biplot depicts the eigenvalues and lengths of eigenvectors for the RDA. Population locations on the spatial axes are marked by their number.

Populations of *F. suspensa* were significantly structured as revealed by the clearly population-based NJ network, the 20 populations were subdivided into four genetic groups (Figure 2). The four genetic groups identified were as follows: East group (P1–P5), North group (P6–P11), Middle group (P12–P16), and South group (P17–P20). Non-hierarchical AMOVA (Table 2) revealed significant differences among these populations (*F*_ST_ = 0.115, *P* < 0.001). Despite the clear genetic subdivision in the 20 populations of *F. suspensa*, only minimal genetic variations existed among these four groups (5.30%, *F*_CT_ = 0.053, *P* < 0.05), and most genetic variations occurred within a population (86.06%, *F*_ST_ = 0.139, *P* < 0.001). Non-significant patterns of isolation-by-distance were detected among the 20 populations of *F. suspensa* (*r* = 0.1209, *P* > 0.05), indicating that geographic distance exerted no significant effect on genetic differentiation. RDA analysis was performed to detect the contribution of environmental variables on spatial genetic structure. The RDA results are shown in Table 3 and Figure 3. Correlations between genetic variables and environmental variables in axes 1 and 2 were 0.953 and 0.976, respectively. The percent variance of the genetic variable-environmental variable relationship in axes 1 and 2 were 26.9 and 19.0%, respectively. RDA results showed that seven environmental variables were significantly associated with RDA axes 1 and 2 (Table 3). Among these seven environmental variables, mean diurnal range (Bio2), temperature seasonality (Bio4), and min temperature of coldest month (Bio6) were related to temperature. Meanwhile, annual precipitation (Bio12), precipitation of the wettest month (Bio13), precipitation seasonality (Bio15), and precipitation of driest quarter (Bio17) were all related to precipitation. Bio12, Bio13, and Bio17 showed the highest correlation among the seven environmental variables.

**Table 2:** **Hierarchical AMOVAs for SCoT variation surveyed in ***F. suspensa*****.

**Source of variation**	***d.f***.	**%Total variance**	***F*-statistic**	***P*-value**
**NON-HIERARCHICAL AMOVAs**				
Total	19	10.87	*F*_ST_ = 0.109	*P* < 0.001
East group	4	6.95	*F*_ST_ = 0.070	*P* < 0.001
North group	5	3.08	*F*_ST_ = 0.031	*P* < 0.001
Middle group	4	6.30	*F*_ST_ = 0.063	*P* < 0.001
South group	3	10.16	*F*_ST_ = 0.102	*P* < 0.001
**HIERARCHICAL AMOVAs**				
Among four groups	3	5.87	*F*_CT_ = 0.059	*P* < 0.001
Among populations	16	6.12	*F*_SC_ = 0.065	*P* < 0.001
Within populations	184	88.01	*F*_ST_ = 0.120	*P* < 0.001

**Table 3:** **Correlations between environmental variables and the ordination axes**.

**Environmental variable**	**Axe 1**	**Axe 2**	**Axe 3**	**Axe 4**
Bio2	−0.530^*^	−0.249	0.611^**^	0.108
Bio3	−0.396	−0.383	0.408	0.123
Bio4	−0.609^**^	0.261	0.617^**^	−0.016
Bio5	0.374	−0.010	0.391	−0.306
Bio6	0.742^**^	0.082	−0.260	−0.280
Bio8	0.540^*^	0.294	0.175	−0.290
Bio12	0.693^**^	0.286	−0.502^*^	0.011
Bio13	0.153	0.760^**^	−0.261	−0.303
Bio15	−0.686^**^	0.374	0.475^*^	−0.216
Bio17	0.823^**^	0.220	−0.339	0.125

Statistically significant correlation by

* P < 0.05 and

** *P < 0.01*.

### EAL detection

**Figure 4:**
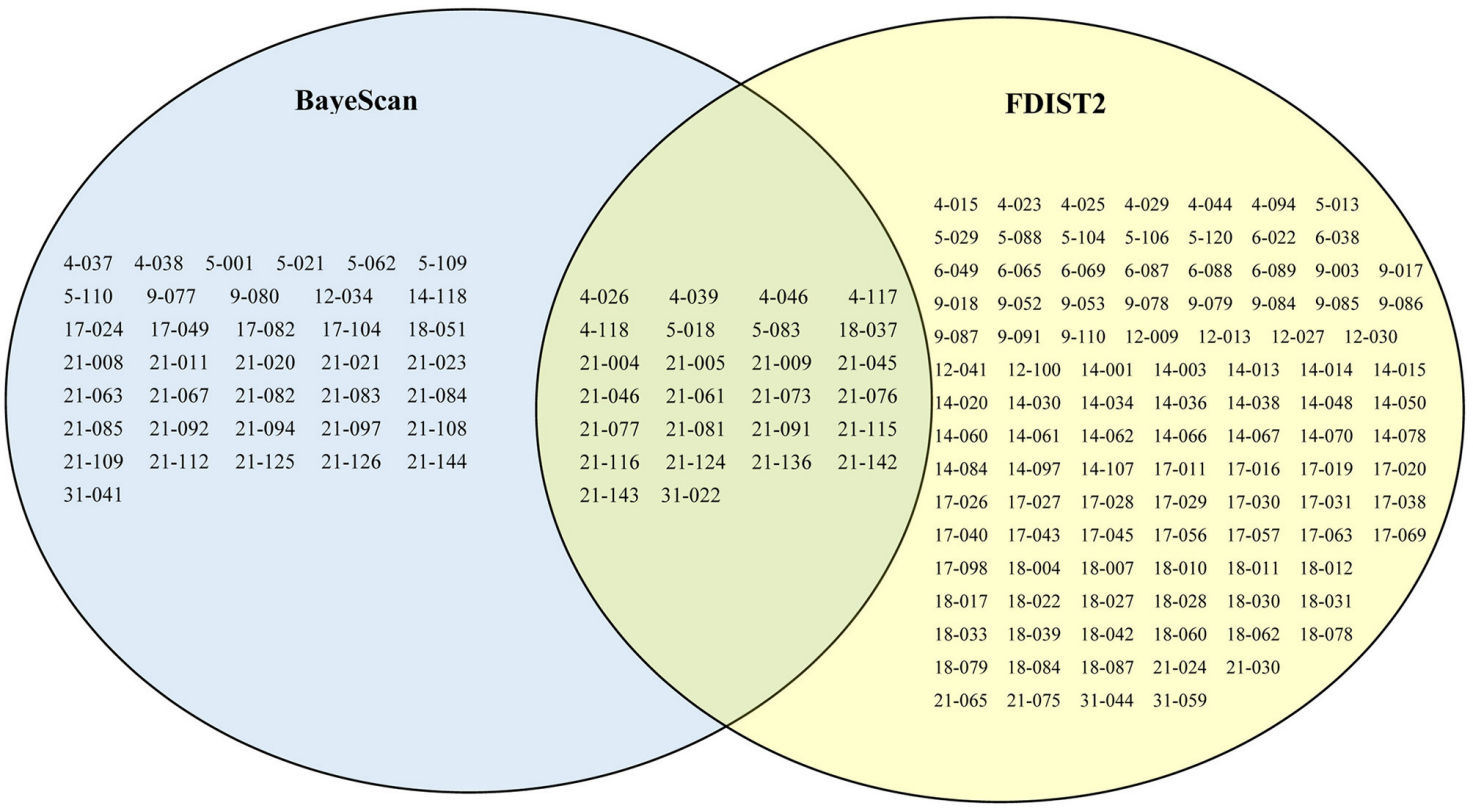
**The results of outlier loci identified by Bayescan and Arlequin**. Sixty-three, one hundred and thirty-two, and twenty six loci were detected as outlier loci in *F. suspensa* using Bayescan, Arlequin, and both with Bayescan and Arlequin, respectively.

Using the BayeScan method, 63 outlier loci were identified (5.1% of the total number of SCoT loci) with a posterior probability of higher than 0.76 (i.e., log_10_ PO > 0.5), which is a threshold for substantial evidence under selection (Table S4). Using the FDIST2 method, a relatively relaxed result of 132 outlier loci (10.6% of the total number of SCoT loci) above the 95% threshold were identified (Table S4). Based on the results yielded by two identified methods, a total of 26 common loci were detected by both methods, which represented more true outlier loci (Figure 4). To further reduce the false discovery rate, we used the 26 common loci for environmental association analyses. Among these outlier loci, 23 environment-associated loci (EAL; 1.9% of the total number of SCoT loci) associated with at least one environmental variable was detected in *F. suspensa* by using LFMM (Table 4). Among these detected EAL, 20 were significantly associated with both temperature and precipitation, three were significantly associated with precipitation (Table 4). Among the 10 environmental variables, Bio13, Bio15, and Bio6 were associated with the highest numbers of EAL, which indicated that these environmental variables might play more important roles in adaptive evolution in *F. suspensa* (Table 4).

**Table 4:** **The EAL as indicated by |***z***|-score**.

**Locus**	**Bio2**	**Bio3**	**Bio4**	**Bio5**	**Bio6**	**Bio8**	**Bio12**	**Bio13**	**Bio15**	**Bio17**
4-026			4.083^***^		4.780^***^	3.392^***^	4.889^***^		5.022^***^	6.150^***^
4-039			3.578^***^		4.466^***^		4.273^***^		3.934^***^	4.685^***^
4-046			3.859^***^		3.790^***^		4.065^***^		4.426^***^	4.946^***^
4-117						3.700^***^		5.691^***^		
4-118						3.913^***^		7.034^***^		
5-018	3.690^***^		8.406^***^					5.828^***^	10.536^***^	3.580^***^
5-083	6.539^***^	3.782^***^	8.600^***^		6.102^***^		6.873^***^		8.491^***^	6.714^***^
18-037				4.195^***^	4.344^***^					
21-004						3.294^***^		6.387^***^	4.402^***^	
21-005						3.848^***^		6.570^***^	4.230^***^	
21-009								5.475^***^		
21-045								4.044^***^	3.312^***^	
21-046								5.012^***^	4.114^***^	
21-061	7.511^***^	6.715^***^	4.228^***^		7.665^***^	5.494^***^	8.349^***^	6.311^***^		7.532^***^
21-073				4.538^***^	5.028^***^	3.812^***^				
21-076			6.053^***^					7.655^***^	7.198^***^	
21-077	4.390^***^		7.796^***^		4.146^***^			3.976^***^	8.423^***^	
21-091				5.201^***^		5.014^***^				3.720^***^
21-115	5.570^***^	6.115^***^			3.600^***^	3.762^***^	5.132^***^	7.759^***^		4.113^***^
21-116	5.333^***^	5.717^***^			4.421^***^	4.887^***^	5.136^***^	7.611^***^		4.088^***^
21-124		3.302^***^		3.856^***^	5.046^***^	6.179^***^	3.616^***^	4.075^***^		3.921^***^
21-136	4.882^***^		6.140^***^		3.475^***^		4.675^***^		5.713^***^	4.338^***^
31-022	3.931^***^		6.169^***^	3.901^***^					5.657^***^	

Statistically significant correlation by

*** *P < 0.001*.

## Discussion

By using 1,242 SCoT loci, we analyzed the adaptive evolution of *F. suspensa* in response to environmental variables. SCoT markers present the advantages of high throughput, non-neutral bias, and non-requirement for a priori genomic information (Deng et al., 2015). Thus, SCoT markers are very suitable for adaptive loci detection. However, markers are seldom applied in landscape population genomics. To date, the genome information of *F. suspensa* (2*n* = 26) is still unknown. According to a high-density genetic linkage map in the related species of *Olea europaea* (2*n* = 46), 5,643 markers produced a high resolution map of 0.53 cM (İpek et al., 2016). Although a smaller number of markers were used in this study, the overall outlier detection rate in *F. suspensa* was 5.1% in BayeScan and 10.6% in FDIST2. These rates are considered with those reported in other studies, such as 2.85% in *Alnus glutinosa* (De Kort et al., 2014) and 4.5% in *Picea mariana* (Prunier et al., 2011) as determined using SNPs, 4.22% in *Cephalotaxus oliveri* (Wang et al., 2016) as determined using ISSRs, and 9% in *Arabis alpina* (Poncet et al., 2010) and 10% in 13 alpine species (Manel et al., 2012) as determined using AFLPs. Although SCoT markers are non-neutral biased, their detection rates in this study are not significantly higher than that of other molecular markers.

Understanding the spatial population genetic structure of species is a key concern in landscape population genomics (Schoville et al., 2012; Hall and Beissinger, 2014). In recent years, research on the influence of environmental variables on spatial structure gradually increased. Identifying the influence of environmental variables on spatial genetic structure remains difficult because of the complex reciprocal interactions of multiple factors (Chung et al., 2004; Ohsawa and Ide, 2008). Our survey demonstrated a significant spatial population genetic structure across the studied populations. These populations could be divided into four genetic groups, namely, East group (P1–P5), North group (P6–P11), Middle group (P12–P16), and South group (P17–P20). Three possible hypotheses can explain the genetic divergence of these populations. First, the current spatial genetic structure resulted from the mixing of the four gene pools. Second, geographical barriers occurred among the four genetic groups, and these barriers blocked the gene flow and ultimately led to genetic differentiation of the four groups. Third, the four groups were exposed to significantly varying environmental conditions, and divergent selection caused by these heterogeneous environmental variables led to genetic divergence of the four groups. Assuming that the first hypothesis is reasonable, high genetic diversity can be expected in populations located at the contact zone. However, results of the genetic diversity index based on SCoT data did not support this explanation. Similarly, the populations (P1, P9, P11, P12, P16, and P17) located at the contact zone of the four groups did not show the expected high genetic diversity. Moreover, *F. suspensa* displays strong pollen dispersal ability, resulting in a high pollen-mediated gene flow among the populations (Fu et al., 2014). The non-significant patterns of IBD in *F. suspensa* also confirmed the strong gene flow among the populations. Long-term strong gene flow among populations would result in a consistent population genetic background. However, the results for population structure are inconsistent with the expected strong gene flow. Thus, the first hypothesis failed to explain the genetic divergence of *F. suspensa*. Assuming that the genetic divergence in *F. suspensa* can be explained by the second hypothesis, we expect to observe considerable genetic divergence among the four groups, with sufficiently strong geographic barrier separating these groups from one another. However, the result of non-hierarchical AMOVA did not support this hypothesis, and only 5.87% genetic variation was observed among the four groups. More importantly, we did not find significant geographical barriers, i.e., mountains and rivers, which separate the four groups. Therefore, the second hypothesis apparently cannot explain the genetic divergence in *F. suspensa*. Assuming that the third hypothesis applies in *F. suspensa*, a shallow genetic divergence among the four groups would be expected because of the interaction between strong gene flow and significant natural selection by environmental variables. The result of non-hierarchical AMOVA (*F*_CT_ = 0.059) was consistent with this expectation. We then performed RDA to test whether the genetic subdivision of the four groups was caused by environmental factors. The RDA results indicated that seven environmental variables related to temperature and precipitation significantly contributed to the spatial genetic structure of *F. suspensa*. Among these environmental variables, Bio12, Bio13, and Bio17 were the most important environmental factors that have shaped the spatial genetic structure of *F. suspensa*. Moreover, RDA results showed that these environmental variables could clearly divide the populations into East group, South group, and the combined Middle and North groups (Figure 3). These findings showed that environmental variables greatly influenced the spatial genetic structure of *F. suspensa*. However, other unknown factors have possibly led to the eventual divergence of the Middle and North groups. Overall, our results for *F. supsensa* support the third hypothesis.

Landscape genomics studies have devoted more attention to the identification of adaptive gene loci (Rellstab et al., 2015; Shryock et al., 2015). However, less attention has been devoted to the environmental variables affecting the adaptive evolution of species. Under extreme environments, a strong selection pressure will facilitate convergent evolution of species (Azua-Bustos et al., 2012). The environmental variables driving the convergent evolution of species in these regions are usually common (Kültz, 2015; Givnish, 2016). However, extreme environmental variables are inexistent in most vegetation distribution areas. In regions lacking extreme environmental variables, the direction of adaptive evolution of the genome is uncertain, and the environmental variables driving their adaptive evolution vary. Thus, identifying whether commonality exists among these environmental variables in driving diverse adaptive evolution of species is important.

In this study, we chose *F. suspensa* as a model to address the above mentioned concerns. This species is distributed in the warm temperate zone in China, and this zone lacks strong selection pressure. We hypothesized that ecological habitats determine adaptive evolution of species and drive the genome to generate a large number of EAL. Thus, understanding the ecological habitats of *F. suspensa* is urgent. In this study, we focused on the ecological habitats related to temperature and precipitation. *F. suspensa* prefers warm and humid climate and can tolerate cold and drought but not waterlogging (Niu et al., 2003). Among the adopted environmental variables, Bio12 was associated with the ecological habitat of “prefer humidity”; Bio6 was associated with the ecological habitat of “tolerate cold”; Bio17 was associated with the ecological habitat of “tolerate drought”; and Bio13 was associated with the ecological habitat of “avoid waterlogging.” Most of the time, the warmest and wettest seasons in the warm temperate zone in China overlap, and the same is true for the coldest and driest seasons. The results of LFMM expectedly showed that the ecological habitat of “avoid waterlogging” (Bio13) were associated with the highest number of EAL; the ecological habitat of “tolerate cold” (Bio6), and “tolerate drought” (Bio17) were associated with increased number of EAL. Unexpectedly, Bio4 and Bio15 were also associated with increased number of EAL. This result implies that seasonal variation in temperature and precipitation exerted strong selection pressure on *F. suspensa* and drove the genome to generate adaptive evolution. However, the ecological habitats of “prefer warmth” and “prefer humidity” was only related to a small number of EAL. Overall, most aspects of the characterized EAL of *F. suspensa* are consistent with the hypothesis that ecological habitats determine adaptive evolution of a species.

Whether this hypothesis demonstrates a certain degree of universality must be determined. The adaptive evolution of *Achyranthes bidentata* and *Cotinus coggygria* in warm temperate zone in China is also associated with their ecological habitats (unpublished data). For *A. bidentata*, the highest number of EAL is associated with its ecological habitats of “cold intolerance” and “avoiding waterlogging.” For *C. coggygria*, the highest number of EAL is associated with its ecological habitat of “avoiding waterlogging.” To further confirm the universality of our hypothesis, we reviewed the recent landscape population genomics studies in other regions. For example, the high number of EAL in *Picea mariana* is related to its ecological habitat of “cold resistance” (Prunier et al., 2011); additionally, EAL is related to “drought resistance” in *Alnus glutinosa* (De Kort et al., 2014), to “temperature and precipitation sensitivity” in *Cephalotaxus oliveri* (Wang et al., 2016), and to “drought and cold tolerance” in *Abies alba* (Roschanski et al., 2016). Based on the above-mentioned evidence, the hypothesis that the ecological habitats determine adaptive evolution of species possibly demonstrate a certain degree of universality.

**Figure 5:**
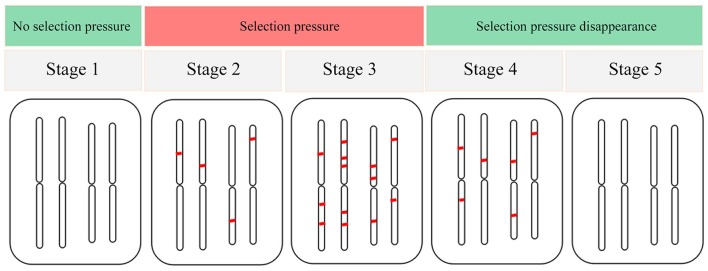
**The adaptation model of species in response to environmental variables**.

Despite the lack of extreme environmental variables in the warm temperate zone in China, various environmental factors more or less produce selection pressure on *F. suspensa*. Therefore, the level of adaptabilities to various environmental variables varies. Another important concern is the evaluation of the adaptation status of species in response to environmental variables. Thus, we propose a model of adaptive evolution of species in response to environmental variables. The adaptive evolution of species was divided into five stages (Figure 5). In stage 1 (S1), environmental variables did not exert selection pressure on the species, and the species did not yield adaptive differentiation. Consequently, EAL could not be detected in the genome. In S2, the altered environmental variables generated selection pressure on the species, and the species genome produced EAL to cope with such selection pressure. Eventually, EAL increased gradually. However, the increased EAL loci were not sufficient to respond to this selection pressure, and species showed sensitivity to this environmental variable at this stage. In S3, the species genome generated an adequate amount of EAL in response to selection pressure. At this stage, species showed adaptability, and this adaptability would be further strengthened with time delay. In S4, selection pressure yielded by environmental variable disappeared or did not occur frequently, and the EAL on the species genome decreased gradually. At this stage, the species was resistant to this environmental variable. In S5, the EAL on the species genome completely disappeared because the selection pressure yielded by this environmental variable has long been inexistent. We used this model to help us identify the adaptation status for *F. suspensa* in response to various environmental variables. Despite a high number of EAL driven by waterlogging (Bio13), *F. suspensa* still showed the ecological habitats of “avoid waterlogging.” Thus, the adaptability for *F. suspensa* in response to waterlogging must have occurred in S2. In the same manner, we detected a high number of EAL associated with cold (Bio6) and drought (Bio17). However, the selection pressure from cold and drought gradually declined and reached a level far below the critical value that *F. suspensa* can tolerate. For example, the minimum temperature during the coldest month (Bio6) from 1950 to 2000 was −2°C, which was far lower than the minimum temperature that *F. suspensa* can tolerate (−30°C; Xu et al., 1996). Thus, we speculate that adaptability of *F. suspensa* to cold and drought occurred in S4. Interestingly, seasonal variation in temperature and precipitation is required as reported by studies on *F. suspensa* cultivation, whereas numerous EALs are associated with temperature seasonality (Bio4) and precipitation seasonality (Bio15). This phenomenon is possibly consistent with the ecological habitats of *F. suspensa*, i.e., “prefer humidity” and “tolerate drought,” as well as “prefer warmth” and “tolerate cold.” Overall, the combination of identification of EAL and ecological habitats and our adaptation model is useful in understanding the adaptation of species to various environmental variables.

## Author contributions

YL conceived and designed the experiments and wrote the paper; JY and CM performed the experiments and analyzed the data; RM checked English grammar and revised the paper. All authors read and approved the final version of the manuscript.

### Conflict of interest statement

The authors declare that the research was conducted in the absence of any commercial or financial relationships that could be construed as a potential conflict of interest.
