# Supplementary material for: Landscape Population Genomics of Forsythia (Forsythia suspensa) Reveal That Ecological Habitats Determine the Adaptive Evolution of Species
Source: Front Plant Sci. 2017 Apr 5;8:481. doi: 10.3389/fpls.2017.00481 (PMC5380681; doi:10.3389/fpls.2017.00481)
Supplement: Supplementary file 1 [file Table1.DOC]

Supplementary Material

**Landscape population genomics of forsythia (*Forsythia suspensa*) reveal that ecological habitats determine the adaptive evolution of species**

**Jie Yang, Cai-Yun Miao, Run-Li Mao, Yong Li***

*** Correspondence:** Yong Li: liyongrui1@126.com

# 1 Supplementary Tables

**Table S1 | Gene frequencies per allele of 1242 alleles for each population.**

| **Locus** | **Allele frequency** | | | | | | | | | | | | | | | | | | | |
| --- | --- | --- | --- | --- | --- | --- | --- | --- | --- | --- | --- | --- | --- | --- | --- | --- | --- | --- | --- | --- |
|  | **1.SXWT** | **2.SDYM** | **3.SDTM** | **4.SDMM** | **5.SDBD** | **6.SXTL** | **7.SXLK** | **8.SXBT** | **9.HBWZ** | **10.HNJL** | **11.SXHM** | **12.SXWL** | **13.HNSM** | **14.SXLJ** | **15.HNLJ** | **16.HNLY** | **17.HBWD** | **18.HNTB** | **19.HNJG** | **20.HBDH** |
| 4-001 | 0.00000 | 0.00000 | 0.00000 | 0.00000 | 0.10000 | 0.00000 | 0.00000 | 0.00000 | 0.00000 | 0.00000 | 0.00000 | 0.00000 | 0.00000 | 0.00000 | 0.00000 | 0.00000 | 0.08333 | 0.10000 | 0.10000 | 0.00000 |
| 4-002 | 0.00000 | 0.12500 | 0.00000 | 0.00000 | 0.10000 | 0.00000 | 0.00000 | 0.00000 | 0.00000 | 0.00000 | 0.10000 | 0.00000 | 0.00000 | 0.00000 | 0.00000 | 0.00000 | 0.16667 | 0.10000 | 0.40000 | 0.66667 |
| 4-003 | 0.00000 | 0.00000 | 0.00000 | 0.00000 | 0.10000 | 0.00000 | 0.00000 | 0.00000 | 0.00000 | 0.00000 | 0.00000 | 0.00000 | 0.00000 | 0.00000 | 0.10000 | 0.00000 | 0.25000 | 0.20000 | 0.30000 | 0.16667 |
| 4-004 | 0.00000 | 0.00000 | 0.00000 | 0.00000 | 0.00000 | 0.00000 | 0.00000 | 0.00000 | 0.00000 | 0.00000 | 0.00000 | 0.00000 | 0.00000 | 0.00000 | 0.00000 | 0.00000 | 0.08333 | 0.00000 | 0.10000 | 0.00000 |
| 4-005 | 0.00000 | 0.00000 | 0.00000 | 0.00000 | 0.10000 | 0.33333 | 0.08333 | 0.50000 | 0.25000 | 0.00000 | 0.20000 | 0.09091 | 0.00000 | 0.00000 | 0.10000 | 0.00000 | 0.16667 | 0.10000 | 0.30000 | 0.33333 |
| 4-006 | 0.00000 | 0.00000 | 0.00000 | 0.00000 | 0.10000 | 0.00000 | 0.00000 | 0.00000 | 0.08333 | 0.00000 | 0.00000 | 0.00000 | 0.00000 | 0.00000 | 0.00000 | 0.00000 | 0.08333 | 0.00000 | 0.30000 | 0.00000 |
| 4-007 | 0.12500 | 0.00000 | 0.00000 | 0.00000 | 0.00000 | 0.50000 | 0.58333 | 0.33333 | 0.41667 | 0.33333 | 0.10000 | 0.27273 | 0.37500 | 0.25000 | 0.10000 | 0.00000 | 0.16667 | 0.00000 | 0.10000 | 0.33333 |
| 4-008 | 0.00000 | 0.00000 | 0.00000 | 0.00000 | 0.00000 | 0.00000 | 0.08333 | 0.00000 | 0.00000 | 0.00000 | 0.00000 | 0.00000 | 0.00000 | 0.25000 | 0.00000 | 0.00000 | 0.08333 | 0.00000 | 0.10000 | 0.33333 |
| 4-009 | 0.00000 | 0.00000 | 0.00000 | 0.00000 | 0.00000 | 0.00000 | 0.00000 | 0.00000 | 0.00000 | 0.00000 | 0.00000 | 0.00000 | 0.00000 | 0.00000 | 0.00000 | 0.00000 | 0.08333 | 0.00000 | 0.10000 | 0.50000 |
| 4-010 | 0.00000 | 0.00000 | 0.00000 | 0.00000 | 0.00000 | 0.00000 | 0.00000 | 0.00000 | 0.00000 | 0.00000 | 0.00000 | 0.00000 | 0.00000 | 0.00000 | 0.00000 | 0.00000 | 0.08333 | 0.10000 | 0.10000 | 0.16667 |
| 4-011 | 0.00000 | 0.00000 | 0.00000 | 0.00000 | 0.00000 | 0.00000 | 0.00000 | 0.00000 | 0.00000 | 0.00000 | 0.00000 | 0.00000 | 0.00000 | 0.00000 | 0.00000 | 0.00000 | 0.00000 | 0.10000 | 0.20000 | 0.66667 |
| 4-012 | 0.00000 | 0.00000 | 0.11111 | 0.00000 | 0.10000 | 0.00000 | 0.00000 | 0.00000 | 0.00000 | 0.00000 | 0.00000 | 0.00000 | 0.00000 | 0.00000 | 0.00000 | 0.00000 | 0.25000 | 0.10000 | 0.30000 | 0.66667 |
| 4-013 | 0.00000 | 0.00000 | 0.00000 | 0.00000 | 0.00000 | 0.00000 | 0.00000 | 0.00000 | 0.00000 | 0.00000 | 0.00000 | 0.00000 | 0.00000 | 0.00000 | 0.00000 | 0.00000 | 0.16667 | 0.00000 | 0.40000 | 0.16667 |
| 4-014 | 0.00000 | 0.00000 | 0.00000 | 0.00000 | 0.00000 | 0.00000 | 0.00000 | 0.00000 | 0.00000 | 0.00000 | 0.10000 | 0.00000 | 0.00000 | 0.00000 | 0.10000 | 0.00000 | 0.08333 | 0.10000 | 0.30000 | 0.00000 |
| 4-015 | 0.00000 | 0.00000 | 0.00000 | 0.00000 | 0.10000 | 0.16667 | 0.08333 | 0.00000 | 0.08333 | 0.00000 | 0.00000 | 0.09091 | 0.00000 | 0.00000 | 0.00000 | 0.00000 | 0.16667 | 0.20000 | 0.20000 | 0.00000 |
| 4-016 | 0.12500 | 0.00000 | 0.00000 | 0.00000 | 0.00000 | 0.16667 | 0.41667 | 0.16667 | 0.25000 | 0.55556 | 0.10000 | 0.00000 | 0.25000 | 0.08333 | 0.00000 | 0.00000 | 0.16667 | 0.20000 | 0.20000 | 0.50000 |
| 4-017 | 0.00000 | 0.25000 | 0.00000 | 0.00000 | 0.10000 | 0.33333 | 0.16667 | 0.33333 | 0.41667 | 0.00000 | 0.30000 | 0.00000 | 0.00000 | 0.16667 | 0.10000 | 0.00000 | 0.08333 | 0.20000 | 0.50000 | 0.00000 |
| 4-018 | 0.12500 | 0.25000 | 0.00000 | 0.16667 | 0.10000 | 0.33333 | 0.25000 | 0.33333 | 0.41667 | 0.00000 | 0.40000 | 0.27273 | 0.25000 | 0.16667 | 0.20000 | 0.30000 | 0.58333 | 0.50000 | 0.70000 | 0.50000 |
| 4-019 | 0.12500 | 0.00000 | 0.11111 | 0.08333 | 0.00000 | 0.33333 | 0.08333 | 0.16667 | 0.08333 | 0.00000 | 0.10000 | 0.00000 | 0.00000 | 0.16667 | 0.00000 | 0.00000 | 0.08333 | 0.20000 | 0.40000 | 0.00000 |
| 4-020 | 0.00000 | 0.00000 | 0.00000 | 0.00000 | 0.10000 | 0.00000 | 0.00000 | 0.16667 | 0.00000 | 0.00000 | 0.00000 | 0.00000 | 0.00000 | 0.00000 | 0.00000 | 0.00000 | 0.16667 | 0.30000 | 0.40000 | 0.00000 |
| 4-021 | 0.00000 | 0.00000 | 0.00000 | 0.00000 | 0.00000 | 0.00000 | 0.08333 | 0.00000 | 0.00000 | 0.00000 | 0.00000 | 0.00000 | 0.00000 | 0.00000 | 0.00000 | 0.00000 | 0.00000 | 0.10000 | 0.10000 | 0.33333 |
| 4-022 | 0.00000 | 0.00000 | 0.00000 | 0.00000 | 0.00000 | 0.00000 | 0.00000 | 0.00000 | 0.00000 | 0.00000 | 0.00000 | 0.00000 | 0.00000 | 0.00000 | 0.00000 | 0.00000 | 0.25000 | 0.20000 | 0.20000 | 0.16667 |
| 4-023 | 0.00000 | 0.00000 | 0.00000 | 0.00000 | 0.00000 | 0.00000 | 0.00000 | 0.00000 | 0.00000 | 0.00000 | 0.00000 | 0.00000 | 0.00000 | 0.00000 | 0.00000 | 0.00000 | 0.41667 | 0.10000 | 0.30000 | 0.66667 |
| 4-024 | 0.00000 | 0.00000 | 0.00000 | 0.00000 | 0.10000 | 0.16667 | 0.08333 | 0.50000 | 0.16667 | 0.00000 | 0.20000 | 0.18182 | 0.12500 | 0.00000 | 0.00000 | 0.00000 | 0.16667 | 0.10000 | 0.50000 | 0.50000 |
| 4-025 | 0.00000 | 0.00000 | 0.00000 | 0.00000 | 0.00000 | 0.00000 | 0.00000 | 0.16667 | 0.08333 | 0.11111 | 0.00000 | 0.00000 | 0.00000 | 0.00000 | 0.00000 | 0.00000 | 0.00000 | 0.00000 | 0.40000 | 0.66667 |
| 4-026 | 0.00000 | 0.25000 | 0.00000 | 0.00000 | 0.10000 | 0.33333 | 0.08333 | 0.33333 | 0.08333 | 0.11111 | 0.10000 | 0.09091 | 0.00000 | 0.00000 | 0.10000 | 0.10000 | 0.66667 | 0.40000 | 0.60000 | 0.83333 |
| 4-027 | 0.12500 | 0.00000 | 0.00000 | 0.00000 | 0.00000 | 0.50000 | 0.16667 | 0.00000 | 0.00000 | 0.00000 | 0.00000 | 0.00000 | 0.00000 | 0.08333 | 0.00000 | 0.00000 | 0.16667 | 0.10000 | 0.20000 | 0.33333 |
| 4-028 | 0.12500 | 0.00000 | 0.00000 | 0.00000 | 0.10000 | 0.33333 | 0.50000 | 0.33333 | 0.41667 | 0.44444 | 0.40000 | 0.36364 | 0.50000 | 0.50000 | 0.30000 | 0.40000 | 0.50000 | 0.20000 | 0.30000 | 0.66667 |
| 4-029 | 0.12500 | 0.12500 | 0.00000 | 0.00000 | 0.00000 | 0.16667 | 0.16667 | 0.00000 | 0.00000 | 0.11111 | 0.20000 | 0.09091 | 0.00000 | 0.25000 | 0.10000 | 0.10000 | 0.16667 | 0.10000 | 0.20000 | 0.33333 |
| 4-030 | 0.00000 | 0.12500 | 0.00000 | 0.00000 | 0.00000 | 0.00000 | 0.16667 | 0.00000 | 0.00000 | 0.11111 | 0.00000 | 0.00000 | 0.00000 | 0.00000 | 0.00000 | 0.00000 | 0.25000 | 0.30000 | 0.40000 | 0.16667 |
| 4-031 | 0.00000 | 0.12500 | 0.00000 | 0.00000 | 0.10000 | 0.00000 | 0.08333 | 0.00000 | 0.00000 | 0.00000 | 0.00000 | 0.00000 | 0.00000 | 0.00000 | 0.00000 | 0.00000 | 0.08333 | 0.10000 | 0.10000 | 0.16667 |
| 4-032 | 0.00000 | 0.12500 | 0.00000 | 0.00000 | 0.00000 | 0.00000 | 0.08333 | 0.00000 | 0.00000 | 0.00000 | 0.00000 | 0.00000 | 0.00000 | 0.00000 | 0.00000 | 0.00000 | 0.08333 | 0.20000 | 0.10000 | 0.00000 |
| 4-033 | 0.00000 | 0.12500 | 0.00000 | 0.00000 | 0.10000 | 0.00000 | 0.00000 | 0.16667 | 0.08333 | 0.00000 | 0.00000 | 0.00000 | 0.00000 | 0.00000 | 0.00000 | 0.00000 | 0.16667 | 0.10000 | 0.70000 | 0.16667 |
| 4-034 | 0.00000 | 0.12500 | 0.00000 | 0.00000 | 0.10000 | 0.50000 | 0.08333 | 0.16667 | 0.25000 | 0.22222 | 0.20000 | 0.00000 | 0.00000 | 0.16667 | 0.00000 | 0.00000 | 0.33333 | 0.30000 | 0.60000 | 0.50000 |
| 4-035 | 0.00000 | 0.25000 | 0.00000 | 0.00000 | 0.10000 | 0.16667 | 0.00000 | 0.50000 | 0.16667 | 0.00000 | 0.00000 | 0.09091 | 0.00000 | 0.00000 | 0.00000 | 0.00000 | 0.33333 | 0.50000 | 0.30000 | 0.00000 |
| 4-036 | 0.12500 | 0.25000 | 0.00000 | 0.00000 | 0.10000 | 0.16667 | 0.00000 | 0.50000 | 0.25000 | 0.11111 | 0.00000 | 0.18182 | 0.12500 | 0.00000 | 0.10000 | 0.10000 | 0.58333 | 0.30000 | 0.30000 | 0.16667 |
| 4-037 | 0.00000 | 0.12500 | 0.00000 | 0.00000 | 0.10000 | 0.16667 | 0.08333 | 0.00000 | 0.33333 | 0.00000 | 0.00000 | 0.00000 | 0.00000 | 0.00000 | 0.10000 | 0.00000 | 0.25000 | 0.40000 | 0.50000 | 0.83333 |
| 4-038 | 0.00000 | 0.25000 | 0.00000 | 0.16667 | 0.10000 | 0.33333 | 0.00000 | 0.16667 | 0.25000 | 0.00000 | 0.00000 | 0.18182 | 0.00000 | 0.00000 | 0.10000 | 0.20000 | 0.83333 | 0.40000 | 0.40000 | 0.50000 |
| 4-039 | 0.00000 | 0.25000 | 0.00000 | 0.00000 | 0.10000 | 0.16667 | 0.00000 | 0.16667 | 0.25000 | 0.00000 | 0.00000 | 0.00000 | 0.00000 | 0.08333 | 0.00000 | 0.10000 | 0.66667 | 0.60000 | 0.50000 | 0.16667 |
| 4-040 | 0.12500 | 0.00000 | 0.00000 | 0.00000 | 0.00000 | 0.00000 | 0.00000 | 0.00000 | 0.00000 | 0.00000 | 0.00000 | 0.00000 | 0.00000 | 0.00000 | 0.10000 | 0.00000 | 0.25000 | 0.10000 | 0.10000 | 0.33333 |
| 4-041 | 0.12500 | 0.00000 | 0.00000 | 0.00000 | 0.10000 | 0.00000 | 0.00000 | 0.00000 | 0.00000 | 0.00000 | 0.00000 | 0.00000 | 0.00000 | 0.08333 | 0.00000 | 0.00000 | 0.08333 | 0.30000 | 0.20000 | 0.50000 |
| 4-042 | 0.00000 | 0.00000 | 0.00000 | 0.00000 | 0.00000 | 0.00000 | 0.00000 | 0.00000 | 0.00000 | 0.00000 | 0.00000 | 0.00000 | 0.00000 | 0.08333 | 0.40000 | 0.00000 | 0.00000 | 0.00000 | 0.20000 | 0.33333 |
| 4-043 | 0.12500 | 0.00000 | 0.00000 | 0.00000 | 0.00000 | 0.00000 | 0.00000 | 0.00000 | 0.08333 | 0.00000 | 0.00000 | 0.00000 | 0.00000 | 0.00000 | 0.00000 | 0.00000 | 0.16667 | 0.00000 | 0.20000 | 0.66667 |
| 4-044 | 0.12500 | 0.00000 | 0.00000 | 0.00000 | 0.00000 | 0.00000 | 0.08333 | 0.00000 | 0.08333 | 0.00000 | 0.00000 | 0.00000 | 0.00000 | 0.08333 | 0.00000 | 0.00000 | 0.33333 | 0.20000 | 0.40000 | 0.83333 |
| 4-045 | 0.12500 | 0.00000 | 0.00000 | 0.00000 | 0.00000 | 0.16667 | 0.16667 | 0.33333 | 0.16667 | 0.00000 | 0.10000 | 0.00000 | 0.00000 | 0.00000 | 0.00000 | 0.00000 | 0.16667 | 0.30000 | 0.30000 | 0.50000 |
| 4-046 | 0.12500 | 0.00000 | 0.00000 | 0.00000 | 0.10000 | 0.16667 | 0.08333 | 0.16667 | 0.16667 | 0.00000 | 0.10000 | 0.00000 | 0.00000 | 0.00000 | 0.10000 | 0.00000 | 0.58333 | 0.40000 | 0.30000 | 0.83333 |
| 4-047 | 0.12500 | 0.12500 | 0.00000 | 0.00000 | 0.10000 | 0.00000 | 0.08333 | 0.16667 | 0.00000 | 0.11111 | 0.20000 | 0.09091 | 0.00000 | 0.16667 | 0.20000 | 0.20000 | 0.16667 | 0.40000 | 0.50000 | 0.16667 |
| 4-048 | 0.00000 | 0.12500 | 0.00000 | 0.00000 | 0.00000 | 0.00000 | 0.00000 | 0.00000 | 0.00000 | 0.00000 | 0.10000 | 0.09091 | 0.00000 | 0.00000 | 0.20000 | 0.00000 | 0.33333 | 0.30000 | 0.60000 | 0.33333 |
| 4-049 | 0.12500 | 0.00000 | 0.00000 | 0.00000 | 0.00000 | 0.00000 | 0.08333 | 0.16667 | 0.08333 | 0.00000 | 0.00000 | 0.00000 | 0.00000 | 0.00000 | 0.00000 | 0.10000 | 0.08333 | 0.30000 | 0.10000 | 0.50000 |
| 4-050 | 0.12500 | 0.00000 | 0.00000 | 0.00000 | 0.00000 | 0.16667 | 0.25000 | 0.33333 | 0.16667 | 0.00000 | 0.00000 | 0.00000 | 0.00000 | 0.16667 | 0.10000 | 0.10000 | 0.25000 | 0.50000 | 0.30000 | 0.33333 |
| 4-051 | 0.00000 | 0.00000 | 0.00000 | 0.00000 | 0.10000 | 0.00000 | 0.00000 | 0.00000 | 0.08333 | 0.00000 | 0.00000 | 0.00000 | 0.00000 | 0.00000 | 0.00000 | 0.00000 | 0.25000 | 0.20000 | 0.40000 | 0.66667 |
| 4-052 | 0.00000 | 0.00000 | 0.00000 | 0.00000 | 0.10000 | 0.33333 | 0.16667 | 0.33333 | 0.33333 | 0.00000 | 0.00000 | 0.00000 | 0.00000 | 0.00000 | 0.00000 | 0.10000 | 0.16667 | 0.30000 | 0.30000 | 0.50000 |
| 4-053 | 0.00000 | 0.00000 | 0.00000 | 0.00000 | 0.00000 | 0.00000 | 0.00000 | 0.00000 | 0.08333 | 0.00000 | 0.00000 | 0.00000 | 0.00000 | 0.00000 | 0.30000 | 0.00000 | 0.41667 | 0.20000 | 0.20000 | 0.16667 |
| 4-054 | 0.00000 | 0.00000 | 0.00000 | 0.00000 | 0.00000 | 0.00000 | 0.08333 | 0.00000 | 0.08333 | 0.00000 | 0.00000 | 0.00000 | 0.00000 | 0.00000 | 0.00000 | 0.00000 | 0.41667 | 0.10000 | 0.10000 | 0.33333 |
| 4-055 | 0.00000 | 0.00000 | 0.00000 | 0.00000 | 0.10000 | 0.00000 | 0.00000 | 0.00000 | 0.00000 | 0.00000 | 0.00000 | 0.00000 | 0.00000 | 0.00000 | 0.00000 | 0.00000 | 0.16667 | 0.10000 | 0.40000 | 0.33333 |
| 4-056 | 0.00000 | 0.25000 | 0.00000 | 0.00000 | 0.00000 | 0.16667 | 0.08333 | 0.00000 | 0.08333 | 0.00000 | 0.00000 | 0.00000 | 0.00000 | 0.08333 | 0.10000 | 0.10000 | 0.50000 | 0.40000 | 0.40000 | 0.66667 |
| 4-057 | 0.00000 | 0.00000 | 0.00000 | 0.00000 | 0.10000 | 0.33333 | 0.00000 | 0.16667 | 0.16667 | 0.22222 | 0.10000 | 0.09091 | 0.00000 | 0.25000 | 0.00000 | 0.10000 | 0.50000 | 0.30000 | 0.30000 | 0.83333 |
| 4-058 | 0.00000 | 0.00000 | 0.00000 | 0.00000 | 0.00000 | 0.33333 | 0.00000 | 0.16667 | 0.25000 | 0.11111 | 0.00000 | 0.09091 | 0.00000 | 0.16667 | 0.10000 | 0.20000 | 0.25000 | 0.20000 | 0.30000 | 0.50000 |
| 4-059 | 0.00000 | 0.25000 | 0.00000 | 0.00000 | 0.10000 | 0.33333 | 0.33333 | 0.16667 | 0.25000 | 0.00000 | 0.40000 | 0.18182 | 0.12500 | 0.25000 | 0.40000 | 0.20000 | 0.66667 | 0.60000 | 0.50000 | 0.33333 |
| 4-060 | 0.00000 | 0.12500 | 0.00000 | 0.00000 | 0.00000 | 0.00000 | 0.16667 | 0.16667 | 0.16667 | 0.00000 | 0.00000 | 0.00000 | 0.00000 | 0.08333 | 0.00000 | 0.00000 | 0.58333 | 0.40000 | 0.10000 | 0.33333 |
| 4-061 | 0.00000 | 0.12500 | 0.00000 | 0.00000 | 0.10000 | 0.33333 | 0.00000 | 0.33333 | 0.00000 | 0.00000 | 0.10000 | 0.09091 | 0.37500 | 0.25000 | 0.40000 | 0.40000 | 0.33333 | 0.30000 | 0.30000 | 0.50000 |
| 4-062 | 0.12500 | 0.25000 | 0.00000 | 0.00000 | 0.10000 | 0.16667 | 0.33333 | 0.16667 | 0.41667 | 0.00000 | 0.10000 | 0.09091 | 0.00000 | 0.00000 | 0.00000 | 0.00000 | 0.16667 | 0.40000 | 0.40000 | 0.16667 |
| 4-063 | 0.00000 | 0.12500 | 0.00000 | 0.00000 | 0.00000 | 0.16667 | 0.16667 | 0.16667 | 0.33333 | 0.00000 | 0.00000 | 0.09091 | 0.00000 | 0.00000 | 0.00000 | 0.00000 | 0.16667 | 0.00000 | 0.50000 | 0.16667 |
| 4-064 | 0.00000 | 0.00000 | 0.00000 | 0.00000 | 0.10000 | 0.00000 | 0.00000 | 0.00000 | 0.08333 | 0.11111 | 0.00000 | 0.00000 | 0.00000 | 0.00000 | 0.00000 | 0.00000 | 0.08333 | 0.00000 | 0.20000 | 0.33333 |
| 4-065 | 0.00000 | 0.12500 | 0.00000 | 0.08333 | 0.10000 | 0.16667 | 0.00000 | 0.00000 | 0.16667 | 0.00000 | 0.00000 | 0.00000 | 0.00000 | 0.00000 | 0.00000 | 0.00000 | 0.25000 | 0.20000 | 0.30000 | 0.16667 |
| 4-066 | 0.00000 | 0.00000 | 0.00000 | 0.00000 | 0.10000 | 0.00000 | 0.08333 | 0.00000 | 0.16667 | 0.00000 | 0.10000 | 0.00000 | 0.00000 | 0.00000 | 0.00000 | 0.00000 | 0.33333 | 0.10000 | 0.20000 | 0.33333 |
| 4-067 | 0.12500 | 0.00000 | 0.00000 | 0.00000 | 0.00000 | 0.00000 | 0.08333 | 0.00000 | 0.16667 | 0.00000 | 0.00000 | 0.09091 | 0.00000 | 0.00000 | 0.00000 | 0.00000 | 0.25000 | 0.30000 | 0.10000 | 0.00000 |
| 4-068 | 0.00000 | 0.12500 | 0.00000 | 0.00000 | 0.00000 | 0.00000 | 0.08333 | 0.00000 | 0.08333 | 0.00000 | 0.00000 | 0.00000 | 0.00000 | 0.00000 | 0.00000 | 0.00000 | 0.41667 | 0.40000 | 0.00000 | 0.33333 |
| 4-069 | 0.00000 | 0.12500 | 0.00000 | 0.00000 | 0.00000 | 0.00000 | 0.08333 | 0.00000 | 0.00000 | 0.00000 | 0.00000 | 0.00000 | 0.00000 | 0.00000 | 0.00000 | 0.00000 | 0.25000 | 0.20000 | 0.10000 | 0.16667 |
| 4-070 | 0.00000 | 0.00000 | 0.00000 | 0.00000 | 0.00000 | 0.00000 | 0.00000 | 0.00000 | 0.00000 | 0.11111 | 0.00000 | 0.00000 | 0.00000 | 0.00000 | 0.00000 | 0.00000 | 0.00000 | 0.00000 | 0.30000 | 0.50000 |
| 4-071 | 0.00000 | 0.00000 | 0.00000 | 0.00000 | 0.00000 | 0.00000 | 0.00000 | 0.00000 | 0.00000 | 0.00000 | 0.00000 | 0.00000 | 0.00000 | 0.00000 | 0.00000 | 0.00000 | 0.16667 | 0.20000 | 0.20000 | 0.16667 |
| 4-072 | 0.00000 | 0.00000 | 0.00000 | 0.00000 | 0.00000 | 0.33333 | 0.50000 | 0.33333 | 0.50000 | 0.55556 | 0.30000 | 0.36364 | 0.37500 | 0.50000 | 0.20000 | 0.30000 | 0.25000 | 0.50000 | 0.50000 | 0.33333 |
| 4-073 | 0.00000 | 0.00000 | 0.00000 | 0.00000 | 0.00000 | 0.50000 | 0.41667 | 0.33333 | 0.16667 | 0.44444 | 0.30000 | 0.27273 | 0.37500 | 0.41667 | 0.20000 | 0.30000 | 0.25000 | 0.20000 | 0.20000 | 0.50000 |
| 4-074 | 0.12500 | 0.00000 | 0.00000 | 0.00000 | 0.10000 | 0.00000 | 0.08333 | 0.00000 | 0.00000 | 0.00000 | 0.00000 | 0.00000 | 0.00000 | 0.00000 | 0.00000 | 0.00000 | 0.00000 | 0.10000 | 0.30000 | 0.16667 |
| 4-075 | 0.12500 | 0.00000 | 0.00000 | 0.08333 | 0.20000 | 0.16667 | 0.25000 | 0.66667 | 0.00000 | 0.00000 | 0.10000 | 0.00000 | 0.25000 | 0.00000 | 0.30000 | 0.00000 | 0.41667 | 0.50000 | 0.50000 | 0.50000 |
| 4-076 | 0.12500 | 0.00000 | 0.00000 | 0.08333 | 0.00000 | 0.50000 | 0.50000 | 0.33333 | 0.33333 | 0.77778 | 0.20000 | 0.45455 | 0.25000 | 0.41667 | 0.10000 | 0.40000 | 0.25000 | 0.30000 | 0.30000 | 0.33333 |
| 4-077 | 0.00000 | 0.00000 | 0.00000 | 0.00000 | 0.00000 | 0.00000 | 0.00000 | 0.00000 | 0.00000 | 0.00000 | 0.00000 | 0.00000 | 0.00000 | 0.00000 | 0.00000 | 0.00000 | 0.00000 | 0.00000 | 0.20000 | 0.33333 |
| 4-078 | 0.12500 | 0.00000 | 0.00000 | 0.08333 | 0.00000 | 0.00000 | 0.08333 | 0.00000 | 0.16667 | 0.00000 | 0.00000 | 0.09091 | 0.00000 | 0.00000 | 0.00000 | 0.00000 | 0.58333 | 0.10000 | 0.00000 | 0.33333 |
| 4-079 | 0.00000 | 0.00000 | 0.00000 | 0.00000 | 0.00000 | 0.00000 | 0.08333 | 0.00000 | 0.08333 | 0.00000 | 0.00000 | 0.09091 | 0.00000 | 0.00000 | 0.00000 | 0.00000 | 0.41667 | 0.20000 | 0.10000 | 0.16667 |
| 4-080 | 0.00000 | 0.37500 | 0.11111 | 0.08333 | 0.10000 | 0.00000 | 0.00000 | 0.00000 | 0.00000 | 0.00000 | 0.00000 | 0.00000 | 0.00000 | 0.00000 | 0.00000 | 0.00000 | 0.16667 | 0.30000 | 0.60000 | 0.16667 |
| 4-081 | 0.00000 | 0.12500 | 0.11111 | 0.08333 | 0.00000 | 0.00000 | 0.08333 | 0.00000 | 0.08333 | 0.00000 | 0.00000 | 0.18182 | 0.00000 | 0.08333 | 0.00000 | 0.00000 | 0.41667 | 0.40000 | 0.30000 | 0.66667 |
| 4-082 | 0.00000 | 0.12500 | 0.00000 | 0.00000 | 0.00000 | 0.00000 | 0.08333 | 0.00000 | 0.16667 | 0.22222 | 0.00000 | 0.00000 | 0.00000 | 0.08333 | 0.10000 | 0.00000 | 0.41667 | 0.10000 | 0.00000 | 0.66667 |
| 4-083 | 0.00000 | 0.00000 | 0.00000 | 0.00000 | 0.00000 | 0.16667 | 0.08333 | 0.00000 | 0.16667 | 0.44444 | 0.00000 | 0.00000 | 0.00000 | 0.00000 | 0.10000 | 0.00000 | 0.00000 | 0.00000 | 0.10000 | 0.16667 |
| 4-084 | 0.00000 | 0.00000 | 0.00000 | 0.08333 | 0.10000 | 0.16667 | 0.00000 | 0.00000 | 0.00000 | 0.00000 | 0.00000 | 0.00000 | 0.00000 | 0.00000 | 0.00000 | 0.00000 | 0.16667 | 0.20000 | 0.20000 | 0.50000 |
| 4-085 | 0.00000 | 0.25000 | 0.00000 | 0.00000 | 0.10000 | 0.16667 | 0.08333 | 0.16667 | 0.25000 | 0.00000 | 0.10000 | 0.18182 | 0.12500 | 0.00000 | 0.00000 | 0.00000 | 0.25000 | 0.20000 | 0.40000 | 0.33333 |
| 4-086 | 0.00000 | 0.25000 | 0.00000 | 0.00000 | 0.00000 | 0.16667 | 0.25000 | 0.16667 | 0.16667 | 0.00000 | 0.10000 | 0.09091 | 0.12500 | 0.00000 | 0.10000 | 0.00000 | 0.08333 | 0.20000 | 0.40000 | 0.33333 |
| 4-087 | 0.00000 | 0.00000 | 0.00000 | 0.00000 | 0.00000 | 0.00000 | 0.00000 | 0.00000 | 0.00000 | 0.00000 | 0.00000 | 0.00000 | 0.00000 | 0.00000 | 0.00000 | 0.00000 | 0.08333 | 0.20000 | 0.10000 | 0.00000 |
| 4-088 | 0.00000 | 0.00000 | 0.00000 | 0.00000 | 0.00000 | 0.00000 | 0.00000 | 0.00000 | 0.00000 | 0.00000 | 0.00000 | 0.00000 | 0.00000 | 0.00000 | 0.00000 | 0.00000 | 0.08333 | 0.00000 | 0.00000 | 0.00000 |
| 4-089 | 0.00000 | 0.00000 | 0.00000 | 0.00000 | 0.00000 | 0.00000 | 0.00000 | 0.00000 | 0.00000 | 0.00000 | 0.00000 | 0.00000 | 0.00000 | 0.00000 | 0.00000 | 0.00000 | 0.25000 | 0.10000 | 0.10000 | 0.00000 |
| 4-090 | 0.25000 | 0.00000 | 0.00000 | 0.00000 | 0.00000 | 0.00000 | 0.00000 | 0.00000 | 0.00000 | 0.00000 | 0.00000 | 0.00000 | 0.00000 | 0.00000 | 0.00000 | 0.00000 | 0.08333 | 0.00000 | 0.10000 | 0.00000 |
| 4-091 | 0.12500 | 0.12500 | 0.00000 | 0.00000 | 0.10000 | 0.00000 | 0.00000 | 0.00000 | 0.00000 | 0.00000 | 0.00000 | 0.00000 | 0.00000 | 0.00000 | 0.00000 | 0.00000 | 0.08333 | 0.10000 | 0.00000 | 0.00000 |
| 4-092 | 0.12500 | 0.00000 | 0.00000 | 0.00000 | 0.00000 | 0.00000 | 0.00000 | 0.00000 | 0.00000 | 0.00000 | 0.00000 | 0.00000 | 0.00000 | 0.00000 | 0.00000 | 0.00000 | 0.08333 | 0.00000 | 0.20000 | 0.16667 |
| 4-093 | 0.12500 | 0.00000 | 0.00000 | 0.00000 | 0.00000 | 0.00000 | 0.00000 | 0.00000 | 0.00000 | 0.00000 | 0.00000 | 0.00000 | 0.00000 | 0.00000 | 0.00000 | 0.00000 | 0.08333 | 0.00000 | 0.10000 | 0.33333 |
| 4-094 | 0.12500 | 0.12500 | 0.00000 | 0.00000 | 0.10000 | 0.33333 | 0.00000 | 0.16667 | 0.08333 | 0.00000 | 0.00000 | 0.09091 | 0.00000 | 0.08333 | 0.00000 | 0.00000 | 0.08333 | 0.20000 | 0.30000 | 0.16667 |
| 4-095 | 0.00000 | 0.12500 | 0.00000 | 0.00000 | 0.00000 | 0.00000 | 0.08333 | 0.00000 | 0.00000 | 0.00000 | 0.00000 | 0.00000 | 0.00000 | 0.00000 | 0.00000 | 0.00000 | 0.00000 | 0.20000 | 0.20000 | 0.00000 |
| 4-096 | 0.00000 | 0.12500 | 0.00000 | 0.00000 | 0.10000 | 0.33333 | 0.08333 | 0.16667 | 0.08333 | 0.00000 | 0.00000 | 0.09091 | 0.00000 | 0.00000 | 0.00000 | 0.00000 | 0.16667 | 0.30000 | 0.20000 | 0.00000 |
| 4-097 | 0.00000 | 0.00000 | 0.00000 | 0.00000 | 0.00000 | 0.00000 | 0.08333 | 0.00000 | 0.08333 | 0.00000 | 0.00000 | 0.00000 | 0.00000 | 0.00000 | 0.00000 | 0.00000 | 0.00000 | 0.00000 | 0.00000 | 0.00000 |
| 4-098 | 0.00000 | 0.00000 | 0.00000 | 0.00000 | 0.00000 | 0.00000 | 0.00000 | 0.00000 | 0.00000 | 0.00000 | 0.00000 | 0.00000 | 0.00000 | 0.00000 | 0.00000 | 0.00000 | 0.08333 | 0.00000 | 0.20000 | 0.16667 |
| 4-099 | 0.00000 | 0.00000 | 0.00000 | 0.00000 | 0.00000 | 0.00000 | 0.00000 | 0.00000 | 0.00000 | 0.00000 | 0.00000 | 0.00000 | 0.00000 | 0.00000 | 0.00000 | 0.00000 | 0.08333 | 0.00000 | 0.00000 | 0.33333 |
| 4-100 | 0.00000 | 0.00000 | 0.00000 | 0.00000 | 0.00000 | 0.00000 | 0.00000 | 0.00000 | 0.00000 | 0.00000 | 0.10000 | 0.00000 | 0.00000 | 0.00000 | 0.10000 | 0.00000 | 0.08333 | 0.00000 | 0.10000 | 0.16667 |
| 4-101 | 0.00000 | 0.12500 | 0.00000 | 0.00000 | 0.10000 | 0.00000 | 0.00000 | 0.00000 | 0.00000 | 0.00000 | 0.10000 | 0.00000 | 0.00000 | 0.00000 | 0.10000 | 0.00000 | 0.08333 | 0.10000 | 0.30000 | 0.00000 |
| 4-102 | 0.00000 | 0.00000 | 0.00000 | 0.00000 | 0.10000 | 0.00000 | 0.00000 | 0.16667 | 0.00000 | 0.00000 | 0.00000 | 0.00000 | 0.00000 | 0.00000 | 0.00000 | 0.00000 | 0.00000 | 0.10000 | 0.10000 | 0.16667 |
| 4-103 | 0.00000 | 0.00000 | 0.00000 | 0.00000 | 0.00000 | 0.00000 | 0.08333 | 0.00000 | 0.00000 | 0.00000 | 0.00000 | 0.00000 | 0.00000 | 0.00000 | 0.00000 | 0.00000 | 0.00000 | 0.10000 | 0.20000 | 0.00000 |
| 4-104 | 0.12500 | 0.00000 | 0.00000 | 0.00000 | 0.00000 | 0.16667 | 0.16667 | 0.33333 | 0.08333 | 0.44444 | 0.10000 | 0.00000 | 0.00000 | 0.08333 | 0.00000 | 0.00000 | 0.00000 | 0.30000 | 0.10000 | 0.00000 |
| 4-105 | 0.00000 | 0.00000 | 0.00000 | 0.00000 | 0.00000 | 0.33333 | 0.50000 | 0.00000 | 0.41667 | 0.33333 | 0.20000 | 0.18182 | 0.12500 | 0.08333 | 0.00000 | 0.10000 | 0.00000 | 0.20000 | 0.10000 | 0.00000 |
| 4-106 | 0.00000 | 0.00000 | 0.00000 | 0.00000 | 0.00000 | 0.00000 | 0.00000 | 0.00000 | 0.00000 | 0.00000 | 0.00000 | 0.00000 | 0.00000 | 0.00000 | 0.00000 | 0.00000 | 0.33333 | 0.00000 | 0.00000 | 0.16667 |
| 4-107 | 0.00000 | 0.00000 | 0.00000 | 0.00000 | 0.00000 | 0.00000 | 0.00000 | 0.00000 | 0.00000 | 0.00000 | 0.00000 | 0.00000 | 0.00000 | 0.00000 | 0.00000 | 0.00000 | 0.00000 | 0.00000 | 0.20000 | 0.16667 |
| 4-108 | 0.00000 | 0.00000 | 0.00000 | 0.00000 | 0.00000 | 0.00000 | 0.00000 | 0.00000 | 0.00000 | 0.00000 | 0.00000 | 0.00000 | 0.00000 | 0.00000 | 0.00000 | 0.00000 | 0.00000 | 0.00000 | 0.20000 | 0.33333 |
| 4-109 | 0.12500 | 0.00000 | 0.00000 | 0.00000 | 0.00000 | 0.00000 | 0.00000 | 0.00000 | 0.00000 | 0.00000 | 0.00000 | 0.00000 | 0.00000 | 0.00000 | 0.00000 | 0.00000 | 0.16667 | 0.00000 | 0.10000 | 0.16667 |
| 4-110 | 0.00000 | 0.00000 | 0.00000 | 0.00000 | 0.00000 | 0.00000 | 0.00000 | 0.00000 | 0.00000 | 0.00000 | 0.00000 | 0.00000 | 0.00000 | 0.00000 | 0.00000 | 0.00000 | 0.00000 | 0.10000 | 0.00000 | 0.16667 |
| 4-111 | 0.00000 | 0.00000 | 0.00000 | 0.00000 | 0.00000 | 0.00000 | 0.00000 | 0.00000 | 0.00000 | 0.00000 | 0.00000 | 0.00000 | 0.00000 | 0.00000 | 0.00000 | 0.00000 | 0.00000 | 0.20000 | 0.10000 | 0.16667 |
| 4-112 | 0.12500 | 0.00000 | 0.00000 | 0.00000 | 0.00000 | 0.00000 | 0.25000 | 0.33333 | 0.33333 | 0.22222 | 0.00000 | 0.00000 | 0.12500 | 0.00000 | 0.00000 | 0.00000 | 0.25000 | 0.10000 | 0.10000 | 0.16667 |
| 4-113 | 0.00000 | 0.00000 | 0.00000 | 0.00000 | 0.00000 | 0.00000 | 0.25000 | 0.33333 | 0.33333 | 0.22222 | 0.10000 | 0.09091 | 0.00000 | 0.08333 | 0.00000 | 0.00000 | 0.58333 | 0.40000 | 0.00000 | 0.16667 |
| 4-114 | 0.50000 | 0.00000 | 0.00000 | 0.16667 | 0.00000 | 0.00000 | 0.00000 | 0.00000 | 0.00000 | 0.00000 | 0.00000 | 0.00000 | 0.00000 | 0.00000 | 0.00000 | 0.00000 | 0.16667 | 0.20000 | 0.20000 | 0.16667 |
| 4-115 | 0.50000 | 0.00000 | 0.00000 | 0.16667 | 0.00000 | 0.00000 | 0.25000 | 0.00000 | 0.33333 | 0.11111 | 0.10000 | 0.00000 | 0.00000 | 0.00000 | 0.10000 | 0.00000 | 0.08333 | 0.00000 | 0.20000 | 0.00000 |
| 4-116 | 0.12500 | 0.12500 | 0.11111 | 0.00000 | 0.30000 | 0.00000 | 0.00000 | 0.00000 | 0.00000 | 0.00000 | 0.00000 | 0.00000 | 0.00000 | 0.00000 | 0.00000 | 0.00000 | 0.00000 | 0.00000 | 0.30000 | 0.16667 |
| 4-117 | 0.37500 | 0.37500 | 0.22222 | 0.50000 | 0.70000 | 0.00000 | 0.00000 | 0.00000 | 0.00000 | 0.00000 | 0.10000 | 0.00000 | 0.00000 | 0.00000 | 0.00000 | 0.00000 | 0.00000 | 0.10000 | 0.30000 | 0.16667 |
| 4-118 | 0.50000 | 0.50000 | 0.33333 | 0.50000 | 0.80000 | 0.00000 | 0.00000 | 0.00000 | 0.16667 | 0.00000 | 0.00000 | 0.00000 | 0.00000 | 0.08333 | 0.00000 | 0.00000 | 0.25000 | 0.00000 | 0.40000 | 0.50000 |
| 4-119 | 0.00000 | 0.00000 | 0.00000 | 0.00000 | 0.00000 | 0.00000 | 0.00000 | 0.00000 | 0.08333 | 0.00000 | 0.00000 | 0.00000 | 0.00000 | 0.08333 | 0.00000 | 0.00000 | 0.50000 | 0.00000 | 0.20000 | 0.33333 |
| 4-120 | 0.00000 | 0.00000 | 0.00000 | 0.00000 | 0.00000 | 0.00000 | 0.00000 | 0.00000 | 0.00000 | 0.11111 | 0.00000 | 0.00000 | 0.00000 | 0.00000 | 0.00000 | 0.00000 | 0.16667 | 0.00000 | 0.00000 | 0.16667 |
| 4-121 | 0.00000 | 0.00000 | 0.00000 | 0.00000 | 0.00000 | 0.00000 | 0.00000 | 0.00000 | 0.00000 | 0.11111 | 0.00000 | 0.00000 | 0.00000 | 0.00000 | 0.00000 | 0.00000 | 0.41667 | 0.20000 | 0.00000 | 0.16667 |
| 4-122 | 0.00000 | 0.00000 | 0.00000 | 0.00000 | 0.00000 | 0.00000 | 0.00000 | 0.00000 | 0.00000 | 0.00000 | 0.00000 | 0.00000 | 0.00000 | 0.00000 | 0.00000 | 0.00000 | 0.33333 | 0.00000 | 0.00000 | 0.33333 |
| 4-123 | 0.00000 | 0.00000 | 0.00000 | 0.00000 | 0.00000 | 0.00000 | 0.00000 | 0.00000 | 0.00000 | 0.00000 | 0.00000 | 0.00000 | 0.00000 | 0.00000 | 0.00000 | 0.00000 | 0.08333 | 0.20000 | 0.00000 | 0.00000 |
| 4-124 | 0.00000 | 0.00000 | 0.00000 | 0.00000 | 0.00000 | 0.00000 | 0.00000 | 0.00000 | 0.00000 | 0.00000 | 0.00000 | 0.00000 | 0.00000 | 0.00000 | 0.00000 | 0.00000 | 0.00000 | 0.00000 | 0.00000 | 0.16667 |
| 4-125 | 0.00000 | 0.00000 | 0.00000 | 0.00000 | 0.00000 | 0.00000 | 0.00000 | 0.00000 | 0.00000 | 0.00000 | 0.00000 | 0.00000 | 0.00000 | 0.00000 | 0.00000 | 0.00000 | 0.08333 | 0.00000 | 0.00000 | 0.16667 |
| 4-126 | 0.00000 | 0.00000 | 0.00000 | 0.00000 | 0.00000 | 0.00000 | 0.00000 | 0.00000 | 0.00000 | 0.00000 | 0.00000 | 0.00000 | 0.00000 | 0.00000 | 0.00000 | 0.00000 | 0.08333 | 0.00000 | 0.10000 | 0.00000 |
| 4-127 | 0.00000 | 0.00000 | 0.00000 | 0.00000 | 0.10000 | 0.00000 | 0.00000 | 0.00000 | 0.00000 | 0.00000 | 0.00000 | 0.00000 | 0.00000 | 0.00000 | 0.00000 | 0.00000 | 0.25000 | 0.10000 | 0.00000 | 0.16667 |
| 4-128 | 0.00000 | 0.00000 | 0.00000 | 0.00000 | 0.10000 | 0.00000 | 0.00000 | 0.00000 | 0.00000 | 0.00000 | 0.00000 | 0.00000 | 0.00000 | 0.00000 | 0.00000 | 0.00000 | 0.00000 | 0.00000 | 0.00000 | 0.00000 |
| 4-129 | 0.12500 | 0.12500 | 0.11111 | 0.00000 | 0.10000 | 0.00000 | 0.00000 | 0.00000 | 0.00000 | 0.00000 | 0.00000 | 0.00000 | 0.00000 | 0.00000 | 0.00000 | 0.00000 | 0.00000 | 0.00000 | 0.00000 | 0.50000 |
| 4-130 | 0.25000 | 0.12500 | 0.22222 | 0.00000 | 0.20000 | 0.00000 | 0.00000 | 0.00000 | 0.00000 | 0.00000 | 0.00000 | 0.00000 | 0.00000 | 0.00000 | 0.00000 | 0.00000 | 0.08333 | 0.00000 | 0.00000 | 0.16667 |
| 4-131 | 0.37500 | 0.25000 | 0.33333 | 0.08333 | 0.20000 | 0.00000 | 0.08333 | 0.00000 | 0.00000 | 0.00000 | 0.00000 | 0.00000 | 0.00000 | 0.00000 | 0.00000 | 0.00000 | 0.00000 | 0.10000 | 0.10000 | 0.16667 |
| 4-132 | 0.50000 | 0.25000 | 0.44444 | 0.08333 | 0.20000 | 0.00000 | 0.08333 | 0.00000 | 0.00000 | 0.00000 | 0.00000 | 0.00000 | 0.00000 | 0.00000 | 0.00000 | 0.00000 | 0.00000 | 0.00000 | 0.10000 | 0.16667 |
| 4-133 | 0.00000 | 0.00000 | 0.00000 | 0.00000 | 0.00000 | 0.00000 | 0.00000 | 0.00000 | 0.00000 | 0.00000 | 0.00000 | 0.00000 | 0.00000 | 0.00000 | 0.00000 | 0.00000 | 0.16667 | 0.00000 | 0.00000 | 0.00000 |
| 4-134 | 0.00000 | 0.00000 | 0.00000 | 0.00000 | 0.00000 | 0.00000 | 0.00000 | 0.00000 | 0.00000 | 0.00000 | 0.00000 | 0.00000 | 0.00000 | 0.00000 | 0.00000 | 0.00000 | 0.08333 | 0.00000 | 0.00000 | 0.00000 |
| 4-135 | 0.00000 | 0.00000 | 0.00000 | 0.00000 | 0.00000 | 0.00000 | 0.00000 | 0.00000 | 0.00000 | 0.00000 | 0.00000 | 0.00000 | 0.00000 | 0.00000 | 0.00000 | 0.00000 | 0.00000 | 0.00000 | 0.10000 | 0.50000 |
| 4-136 | 0.00000 | 0.00000 | 0.00000 | 0.00000 | 0.00000 | 0.00000 | 0.00000 | 0.00000 | 0.00000 | 0.00000 | 0.00000 | 0.00000 | 0.00000 | 0.00000 | 0.00000 | 0.00000 | 0.00000 | 0.00000 | 0.10000 | 0.50000 |
| 4-137 | 0.00000 | 0.00000 | 0.00000 | 0.08333 | 0.00000 | 0.00000 | 0.00000 | 0.00000 | 0.00000 | 0.00000 | 0.00000 | 0.00000 | 0.00000 | 0.00000 | 0.00000 | 0.00000 | 0.08333 | 0.00000 | 0.00000 | 0.16667 |
| 4-138 | 0.00000 | 0.12500 | 0.00000 | 0.08333 | 0.00000 | 0.00000 | 0.00000 | 0.00000 | 0.08333 | 0.00000 | 0.00000 | 0.00000 | 0.00000 | 0.00000 | 0.00000 | 0.00000 | 0.08333 | 0.00000 | 0.00000 | 0.16667 |
| 4-139 | 0.00000 | 0.12500 | 0.00000 | 0.00000 | 0.00000 | 0.00000 | 0.00000 | 0.00000 | 0.00000 | 0.00000 | 0.00000 | 0.00000 | 0.00000 | 0.00000 | 0.00000 | 0.00000 | 0.00000 | 0.00000 | 0.00000 | 0.00000 |
| 4-140 | 0.00000 | 0.00000 | 0.11111 | 0.33333 | 0.00000 | 0.00000 | 0.00000 | 0.00000 | 0.00000 | 0.00000 | 0.00000 | 0.00000 | 0.00000 | 0.00000 | 0.00000 | 0.00000 | 0.08333 | 0.00000 | 0.00000 | 0.33333 |
| 4-141 | 0.00000 | 0.00000 | 0.11111 | 0.33333 | 0.00000 | 0.00000 | 0.00000 | 0.00000 | 0.00000 | 0.00000 | 0.00000 | 0.00000 | 0.00000 | 0.00000 | 0.00000 | 0.00000 | 0.00000 | 0.00000 | 0.00000 | 0.16667 |
| 4-142 | 0.00000 | 0.00000 | 0.00000 | 0.00000 | 0.00000 | 0.00000 | 0.08333 | 0.00000 | 0.08333 | 0.00000 | 0.00000 | 0.00000 | 0.00000 | 0.00000 | 0.00000 | 0.00000 | 0.16667 | 0.20000 | 0.20000 | 0.00000 |
| 4-143 | 0.00000 | 0.00000 | 0.00000 | 0.00000 | 0.00000 | 0.00000 | 0.00000 | 0.00000 | 0.00000 | 0.00000 | 0.00000 | 0.00000 | 0.00000 | 0.00000 | 0.00000 | 0.00000 | 0.08333 | 0.10000 | 0.20000 | 0.00000 |
| 4-144 | 0.00000 | 0.00000 | 0.00000 | 0.00000 | 0.00000 | 0.00000 | 0.00000 | 0.00000 | 0.00000 | 0.00000 | 0.00000 | 0.00000 | 0.00000 | 0.00000 | 0.00000 | 0.00000 | 0.00000 | 0.10000 | 0.00000 | 0.00000 |
| 4-145 | 0.12500 | 0.00000 | 0.00000 | 0.00000 | 0.00000 | 0.00000 | 0.00000 | 0.00000 | 0.00000 | 0.00000 | 0.00000 | 0.00000 | 0.00000 | 0.00000 | 0.00000 | 0.00000 | 0.00000 | 0.00000 | 0.00000 | 0.00000 |
| 4-146 | 0.00000 | 0.00000 | 0.00000 | 0.00000 | 0.00000 | 0.00000 | 0.00000 | 0.00000 | 0.00000 | 0.00000 | 0.00000 | 0.00000 | 0.00000 | 0.00000 | 0.00000 | 0.00000 | 0.00000 | 0.00000 | 0.00000 | 0.00000 |
| 4-147 | 0.00000 | 0.00000 | 0.00000 | 0.00000 | 0.00000 | 0.00000 | 0.00000 | 0.00000 | 0.00000 | 0.11111 | 0.00000 | 0.00000 | 0.00000 | 0.00000 | 0.00000 | 0.00000 | 0.00000 | 0.10000 | 0.00000 | 0.00000 |
| 4-148 | 0.00000 | 0.00000 | 0.00000 | 0.00000 | 0.00000 | 0.00000 | 0.00000 | 0.00000 | 0.00000 | 0.11111 | 0.00000 | 0.00000 | 0.00000 | 0.00000 | 0.00000 | 0.00000 | 0.00000 | 0.10000 | 0.10000 | 0.16667 |
| 4-149 | 0.00000 | 0.00000 | 0.00000 | 0.00000 | 0.00000 | 0.00000 | 0.00000 | 0.00000 | 0.00000 | 0.00000 | 0.00000 | 0.00000 | 0.00000 | 0.00000 | 0.00000 | 0.00000 | 0.00000 | 0.10000 | 0.00000 | 0.16667 |
| 4-150 | 0.00000 | 0.00000 | 0.00000 | 0.00000 | 0.00000 | 0.00000 | 0.00000 | 0.00000 | 0.00000 | 0.00000 | 0.00000 | 0.00000 | 0.00000 | 0.00000 | 0.00000 | 0.00000 | 0.08333 | 0.10000 | 0.00000 | 0.16667 |
| 4-151 | 0.00000 | 0.00000 | 0.00000 | 0.00000 | 0.00000 | 0.00000 | 0.00000 | 0.00000 | 0.00000 | 0.00000 | 0.00000 | 0.00000 | 0.00000 | 0.00000 | 0.00000 | 0.00000 | 0.00000 | 0.10000 | 0.00000 | 0.00000 |
| 4-152 | 0.00000 | 0.00000 | 0.00000 | 0.00000 | 0.00000 | 0.00000 | 0.00000 | 0.00000 | 0.00000 | 0.00000 | 0.00000 | 0.00000 | 0.00000 | 0.00000 | 0.00000 | 0.00000 | 0.08333 | 0.10000 | 0.00000 | 0.50000 |
| 4-153 | 0.00000 | 0.00000 | 0.00000 | 0.00000 | 0.00000 | 0.00000 | 0.08333 | 0.00000 | 0.00000 | 0.00000 | 0.00000 | 0.00000 | 0.00000 | 0.00000 | 0.00000 | 0.00000 | 0.00000 | 0.00000 | 0.00000 | 0.00000 |
| 4-154 | 0.00000 | 0.00000 | 0.00000 | 0.00000 | 0.00000 | 0.00000 | 0.00000 | 0.00000 | 0.00000 | 0.00000 | 0.00000 | 0.00000 | 0.00000 | 0.00000 | 0.00000 | 0.00000 | 0.00000 | 0.10000 | 0.00000 | 0.00000 |
| 4-155 | 0.00000 | 0.00000 | 0.00000 | 0.00000 | 0.00000 | 0.00000 | 0.00000 | 0.00000 | 0.00000 | 0.00000 | 0.00000 | 0.00000 | 0.00000 | 0.00000 | 0.00000 | 0.00000 | 0.08333 | 0.10000 | 0.00000 | 0.66667 |
| 4-156 | 0.00000 | 0.00000 | 0.00000 | 0.00000 | 0.00000 | 0.00000 | 0.00000 | 0.00000 | 0.00000 | 0.00000 | 0.00000 | 0.00000 | 0.00000 | 0.00000 | 0.00000 | 0.00000 | 0.25000 | 0.00000 | 0.00000 | 0.16667 |
| 4-157 | 0.00000 | 0.00000 | 0.00000 | 0.00000 | 0.00000 | 0.00000 | 0.00000 | 0.00000 | 0.00000 | 0.00000 | 0.00000 | 0.00000 | 0.00000 | 0.00000 | 0.00000 | 0.00000 | 0.08333 | 0.00000 | 0.00000 | 0.00000 |
| 4-158 | 0.00000 | 0.12500 | 0.00000 | 0.00000 | 0.00000 | 0.00000 | 0.08333 | 0.00000 | 0.00000 | 0.00000 | 0.00000 | 0.00000 | 0.00000 | 0.00000 | 0.00000 | 0.00000 | 0.08333 | 0.10000 | 0.10000 | 0.16667 |
| 4-159 | 0.00000 | 0.00000 | 0.00000 | 0.00000 | 0.00000 | 0.00000 | 0.08333 | 0.00000 | 0.00000 | 0.00000 | 0.00000 | 0.00000 | 0.00000 | 0.00000 | 0.00000 | 0.00000 | 0.08333 | 0.20000 | 0.10000 | 0.16667 |
| 4-160 | 0.00000 | 0.00000 | 0.00000 | 0.00000 | 0.00000 | 0.00000 | 0.00000 | 0.00000 | 0.00000 | 0.00000 | 0.00000 | 0.00000 | 0.00000 | 0.00000 | 0.00000 | 0.00000 | 0.08333 | 0.10000 | 0.00000 | 0.16667 |
| 4-161 | 0.12500 | 0.12500 | 0.00000 | 0.08333 | 0.10000 | 0.16667 | 0.08333 | 0.16667 | 0.08333 | 0.00000 | 0.00000 | 0.00000 | 0.00000 | 0.00000 | 0.00000 | 0.00000 | 0.08333 | 0.30000 | 0.30000 | 0.16667 |
| 5-001 | 0.28571 | 0.20000 | 0.54545 | 0.66667 | 0.75000 | 0.16667 | 0.09091 | 0.00000 | 0.16667 | 0.00000 | 0.10000 | 0.50000 | 0.50000 | 0.41667 | 0.50000 | 0.60000 | 0.66667 | 0.80000 | 0.90000 | 0.33333 |
| 5-002 | 0.00000 | 0.00000 | 0.00000 | 0.00000 | 0.00000 | 0.00000 | 0.00000 | 0.00000 | 0.00000 | 0.00000 | 0.00000 | 0.08333 | 0.00000 | 0.00000 | 0.00000 | 0.00000 | 0.00000 | 0.00000 | 0.00000 | 0.16667 |
| 5-003 | 0.00000 | 0.00000 | 0.00000 | 0.00000 | 0.00000 | 0.00000 | 0.00000 | 0.00000 | 0.00000 | 0.00000 | 0.00000 | 0.08333 | 0.00000 | 0.08333 | 0.10000 | 0.10000 | 0.08333 | 0.00000 | 0.00000 | 0.00000 |
| 5-004 | 0.00000 | 0.00000 | 0.09091 | 0.00000 | 0.08333 | 0.00000 | 0.00000 | 0.16667 | 0.00000 | 0.00000 | 0.10000 | 0.08333 | 0.00000 | 0.00000 | 0.00000 | 0.00000 | 0.00000 | 0.00000 | 0.00000 | 0.00000 |
| 5-005 | 0.00000 | 0.10000 | 0.00000 | 0.00000 | 0.00000 | 0.00000 | 0.00000 | 0.00000 | 0.00000 | 0.00000 | 0.00000 | 0.16667 | 0.00000 | 0.00000 | 0.00000 | 0.00000 | 0.00000 | 0.00000 | 0.00000 | 0.00000 |
| 5-006 | 0.00000 | 0.10000 | 0.00000 | 0.00000 | 0.00000 | 0.16667 | 0.00000 | 0.00000 | 0.08333 | 0.00000 | 0.00000 | 0.00000 | 0.10000 | 0.16667 | 0.00000 | 0.00000 | 0.00000 | 0.00000 | 0.00000 | 0.00000 |
| 5-007 | 0.00000 | 0.00000 | 0.00000 | 0.00000 | 0.08333 | 0.00000 | 0.00000 | 0.00000 | 0.00000 | 0.00000 | 0.00000 | 0.08333 | 0.00000 | 0.00000 | 0.10000 | 0.10000 | 0.08333 | 0.00000 | 0.00000 | 0.00000 |
| 5-008 | 0.00000 | 0.00000 | 0.00000 | 0.00000 | 0.08333 | 0.00000 | 0.00000 | 0.00000 | 0.00000 | 0.00000 | 0.00000 | 0.00000 | 0.00000 | 0.08333 | 0.00000 | 0.00000 | 0.08333 | 0.00000 | 0.00000 | 0.00000 |
| 5-009 | 0.00000 | 0.10000 | 0.18182 | 0.16667 | 0.16667 | 0.33333 | 0.27273 | 0.33333 | 0.16667 | 0.00000 | 0.00000 | 0.08333 | 0.00000 | 0.25000 | 0.10000 | 0.20000 | 0.08333 | 0.10000 | 0.00000 | 0.66667 |
| 5-010 | 0.00000 | 0.00000 | 0.00000 | 0.00000 | 0.16667 | 0.16667 | 0.00000 | 0.00000 | 0.00000 | 0.00000 | 0.00000 | 0.16667 | 0.10000 | 0.08333 | 0.00000 | 0.00000 | 0.00000 | 0.00000 | 0.00000 | 0.00000 |
| 5-011 | 0.00000 | 0.00000 | 0.00000 | 0.00000 | 0.16667 | 0.00000 | 0.09091 | 0.00000 | 0.08333 | 0.00000 | 0.00000 | 0.00000 | 0.00000 | 0.00000 | 0.00000 | 0.00000 | 0.00000 | 0.00000 | 0.00000 | 0.00000 |
| 5-012 | 0.00000 | 0.00000 | 0.09091 | 0.16667 | 0.25000 | 0.16667 | 0.00000 | 0.00000 | 0.25000 | 0.00000 | 0.20000 | 0.66667 | 0.20000 | 0.50000 | 0.50000 | 0.40000 | 0.50000 | 0.10000 | 0.40000 | 0.00000 |
| 5-013 | 0.00000 | 0.10000 | 0.09091 | 0.00000 | 0.00000 | 0.16667 | 0.09091 | 0.16667 | 0.00000 | 0.00000 | 0.10000 | 0.16667 | 0.00000 | 0.00000 | 0.00000 | 0.20000 | 0.08333 | 0.00000 | 0.10000 | 0.00000 |
| 5-014 | 0.00000 | 0.10000 | 0.00000 | 0.00000 | 0.00000 | 0.16667 | 0.00000 | 0.00000 | 0.00000 | 0.00000 | 0.00000 | 0.00000 | 0.10000 | 0.16667 | 0.00000 | 0.00000 | 0.08333 | 0.10000 | 0.00000 | 0.00000 |
| 5-015 | 0.00000 | 0.00000 | 0.09091 | 0.00000 | 0.00000 | 0.00000 | 0.00000 | 0.16667 | 0.00000 | 0.00000 | 0.00000 | 0.16667 | 0.00000 | 0.00000 | 0.00000 | 0.00000 | 0.00000 | 0.00000 | 0.00000 | 0.00000 |
| 5-016 | 0.00000 | 0.00000 | 0.09091 | 0.00000 | 0.08333 | 0.16667 | 0.00000 | 0.00000 | 0.00000 | 0.00000 | 0.00000 | 0.16667 | 0.00000 | 0.00000 | 0.00000 | 0.00000 | 0.08333 | 0.00000 | 0.00000 | 0.00000 |
| 5-017 | 0.00000 | 0.00000 | 0.00000 | 0.00000 | 0.00000 | 0.00000 | 0.00000 | 0.16667 | 0.00000 | 0.16667 | 0.40000 | 0.75000 | 0.90000 | 0.58333 | 0.70000 | 0.70000 | 0.91667 | 0.80000 | 1.00000 | 0.83333 |
| 5-018 | 0.42857 | 0.60000 | 0.54545 | 0.75000 | 0.66667 | 0.33333 | 0.27273 | 0.16667 | 0.58333 | 0.16667 | 0.00000 | 0.08333 | 0.00000 | 0.00000 | 0.00000 | 0.00000 | 0.00000 | 0.00000 | 0.00000 | 0.00000 |
| 5-019 | 0.00000 | 0.00000 | 0.00000 | 0.00000 | 0.08333 | 0.00000 | 0.00000 | 0.00000 | 0.00000 | 0.00000 | 0.00000 | 0.16667 | 0.00000 | 0.08333 | 0.00000 | 0.00000 | 0.00000 | 0.00000 | 0.00000 | 0.00000 |
| 5-020 | 0.00000 | 0.00000 | 0.00000 | 0.00000 | 0.00000 | 0.16667 | 0.00000 | 0.00000 | 0.00000 | 0.00000 | 0.00000 | 0.16667 | 0.00000 | 0.00000 | 0.00000 | 0.00000 | 0.00000 | 0.00000 | 0.00000 | 0.00000 |
| 5-021 | 0.00000 | 0.10000 | 0.09091 | 0.50000 | 0.33333 | 0.00000 | 0.18182 | 0.16667 | 0.25000 | 0.00000 | 0.10000 | 0.58333 | 0.60000 | 0.58333 | 0.80000 | 0.80000 | 0.75000 | 0.60000 | 0.30000 | 0.66667 |
| 5-022 | 0.00000 | 0.10000 | 0.00000 | 0.00000 | 0.08333 | 0.50000 | 0.36364 | 0.16667 | 0.25000 | 0.00000 | 0.20000 | 0.33333 | 0.00000 | 0.08333 | 0.00000 | 0.40000 | 0.08333 | 0.00000 | 0.10000 | 0.33333 |
| 5-023 | 0.00000 | 0.00000 | 0.00000 | 0.00000 | 0.08333 | 0.16667 | 0.00000 | 0.00000 | 0.08333 | 0.00000 | 0.00000 | 0.41667 | 0.30000 | 0.33333 | 0.00000 | 0.00000 | 0.08333 | 0.20000 | 0.00000 | 0.33333 |
| 5-024 | 0.00000 | 0.30000 | 0.00000 | 0.25000 | 0.08333 | 0.00000 | 0.00000 | 0.00000 | 0.00000 | 0.00000 | 0.10000 | 0.33333 | 0.00000 | 0.25000 | 0.20000 | 0.50000 | 0.41667 | 0.40000 | 0.40000 | 0.16667 |
| 5-025 | 0.00000 | 0.00000 | 0.00000 | 0.00000 | 0.00000 | 0.00000 | 0.00000 | 0.00000 | 0.00000 | 0.00000 | 0.00000 | 0.08333 | 0.00000 | 0.25000 | 0.00000 | 0.10000 | 0.00000 | 0.00000 | 0.10000 | 0.00000 |
| 5-026 | 0.00000 | 0.00000 | 0.00000 | 0.00000 | 0.00000 | 0.16667 | 0.00000 | 0.16667 | 0.00000 | 0.00000 | 0.00000 | 0.00000 | 0.10000 | 0.16667 | 0.00000 | 0.00000 | 0.00000 | 0.00000 | 0.10000 | 0.00000 |
| 5-027 | 0.00000 | 0.20000 | 0.27273 | 0.16667 | 0.16667 | 0.50000 | 0.54545 | 0.50000 | 0.25000 | 0.16667 | 0.30000 | 0.25000 | 0.00000 | 0.41667 | 0.10000 | 0.20000 | 0.16667 | 0.10000 | 0.10000 | 0.66667 |
| 5-028 | 0.00000 | 0.10000 | 0.27273 | 0.08333 | 0.16667 | 0.50000 | 0.18182 | 0.16667 | 0.08333 | 0.00000 | 0.10000 | 0.25000 | 0.00000 | 0.08333 | 0.10000 | 0.00000 | 0.00000 | 0.00000 | 0.00000 | 0.16667 |
| 5-029 | 0.00000 | 0.00000 | 0.00000 | 0.00000 | 0.08333 | 0.00000 | 0.09091 | 0.16667 | 0.00000 | 0.00000 | 0.10000 | 0.16667 | 0.00000 | 0.00000 | 0.00000 | 0.20000 | 0.08333 | 0.10000 | 0.00000 | 0.16667 |
| 5-030 | 0.00000 | 0.00000 | 0.00000 | 0.00000 | 0.00000 | 0.16667 | 0.00000 | 0.00000 | 0.00000 | 0.00000 | 0.00000 | 0.08333 | 0.10000 | 0.08333 | 0.00000 | 0.10000 | 0.08333 | 0.10000 | 0.00000 | 0.16667 |
| 5-031 | 0.00000 | 0.10000 | 0.45455 | 0.33333 | 0.41667 | 0.33333 | 0.45455 | 0.66667 | 0.25000 | 0.00000 | 0.20000 | 0.25000 | 0.20000 | 0.33333 | 0.20000 | 0.40000 | 0.58333 | 0.30000 | 0.40000 | 1.00000 |
| 5-032 | 0.00000 | 0.10000 | 0.18182 | 0.16667 | 0.16667 | 0.00000 | 0.00000 | 0.16667 | 0.08333 | 0.00000 | 0.10000 | 0.00000 | 0.10000 | 0.00000 | 0.20000 | 0.20000 | 0.33333 | 0.10000 | 0.40000 | 0.33333 |
| 5-033 | 0.00000 | 0.00000 | 0.00000 | 0.00000 | 0.08333 | 0.16667 | 0.00000 | 0.00000 | 0.08333 | 0.00000 | 0.00000 | 0.08333 | 0.10000 | 0.08333 | 0.00000 | 0.10000 | 0.08333 | 0.00000 | 0.00000 | 0.50000 |
| 5-034 | 0.00000 | 0.00000 | 0.00000 | 0.08333 | 0.00000 | 0.16667 | 0.09091 | 0.00000 | 0.00000 | 0.00000 | 0.00000 | 0.16667 | 0.00000 | 0.00000 | 0.00000 | 0.00000 | 0.16667 | 0.10000 | 0.00000 | 0.33333 |
| 5-035 | 0.00000 | 0.00000 | 0.27273 | 0.00000 | 0.08333 | 0.00000 | 0.09091 | 0.16667 | 0.08333 | 0.00000 | 0.10000 | 0.50000 | 0.00000 | 0.00000 | 0.10000 | 0.00000 | 0.25000 | 0.20000 | 0.00000 | 0.16667 |
| 5-036 | 0.00000 | 0.00000 | 0.18182 | 0.25000 | 0.25000 | 0.16667 | 0.09091 | 0.16667 | 0.00000 | 0.00000 | 0.00000 | 0.41667 | 0.00000 | 0.00000 | 0.20000 | 0.00000 | 0.08333 | 0.20000 | 0.00000 | 0.16667 |
| 5-037 | 0.00000 | 0.00000 | 0.00000 | 0.00000 | 0.00000 | 0.16667 | 0.09091 | 0.16667 | 0.00000 | 0.00000 | 0.00000 | 0.33333 | 0.00000 | 0.08333 | 0.00000 | 0.40000 | 0.08333 | 0.10000 | 0.00000 | 0.16667 |
| 5-038 | 0.00000 | 0.00000 | 0.00000 | 0.00000 | 0.00000 | 0.16667 | 0.00000 | 0.00000 | 0.08333 | 0.00000 | 0.00000 | 0.25000 | 0.00000 | 0.16667 | 0.00000 | 0.30000 | 0.00000 | 0.10000 | 0.00000 | 0.16667 |
| 5-039 | 0.00000 | 0.10000 | 0.00000 | 0.00000 | 0.00000 | 0.16667 | 0.09091 | 0.16667 | 0.08333 | 0.00000 | 0.00000 | 0.00000 | 0.10000 | 0.25000 | 0.00000 | 0.00000 | 0.00000 | 0.00000 | 0.00000 | 0.00000 |
| 5-040 | 0.00000 | 0.00000 | 0.00000 | 0.00000 | 0.00000 | 0.16667 | 0.00000 | 0.00000 | 0.00000 | 0.00000 | 0.00000 | 0.00000 | 0.10000 | 0.08333 | 0.00000 | 0.10000 | 0.00000 | 0.00000 | 0.00000 | 0.00000 |
| 5-041 | 0.00000 | 0.00000 | 0.00000 | 0.16667 | 0.00000 | 0.00000 | 0.27273 | 0.33333 | 0.00000 | 0.00000 | 0.00000 | 0.08333 | 0.00000 | 0.00000 | 0.10000 | 0.00000 | 0.08333 | 0.00000 | 0.10000 | 0.33333 |
| 5-042 | 0.00000 | 0.00000 | 0.00000 | 0.00000 | 0.08333 | 0.00000 | 0.27273 | 0.50000 | 0.00000 | 0.00000 | 0.60000 | 0.25000 | 0.10000 | 0.08333 | 0.20000 | 0.00000 | 0.08333 | 0.00000 | 0.20000 | 0.33333 |
| 5-043 | 0.28571 | 0.10000 | 0.36364 | 0.41667 | 0.50000 | 0.16667 | 0.27273 | 0.16667 | 0.50000 | 0.16667 | 0.10000 | 0.50000 | 0.30000 | 0.25000 | 0.70000 | 0.40000 | 0.66667 | 0.60000 | 0.90000 | 0.33333 |
| 5-044 | 0.28571 | 0.10000 | 0.36364 | 0.58333 | 0.50000 | 0.33333 | 0.36364 | 0.33333 | 0.58333 | 0.16667 | 0.10000 | 0.66667 | 0.50000 | 0.33333 | 0.70000 | 0.40000 | 0.66667 | 0.70000 | 0.80000 | 0.33333 |
| 5-045 | 0.00000 | 0.00000 | 0.18182 | 0.16667 | 0.08333 | 0.00000 | 0.09091 | 0.33333 | 0.08333 | 0.00000 | 0.00000 | 0.33333 | 0.40000 | 0.50000 | 0.10000 | 0.10000 | 0.16667 | 0.10000 | 0.00000 | 0.16667 |
| 5-046 | 0.00000 | 0.00000 | 0.09091 | 0.08333 | 0.00000 | 0.00000 | 0.00000 | 0.00000 | 0.08333 | 0.00000 | 0.00000 | 0.25000 | 0.40000 | 0.41667 | 0.10000 | 0.10000 | 0.08333 | 0.20000 | 0.20000 | 0.16667 |
| 5-047 | 0.00000 | 0.00000 | 0.00000 | 0.00000 | 0.08333 | 0.00000 | 0.00000 | 0.16667 | 0.00000 | 0.00000 | 0.00000 | 0.16667 | 0.20000 | 0.16667 | 0.10000 | 0.00000 | 0.00000 | 0.20000 | 0.30000 | 0.33333 |
| 5-048 | 0.00000 | 0.00000 | 0.00000 | 0.00000 | 0.00000 | 0.00000 | 0.00000 | 0.16667 | 0.00000 | 0.00000 | 0.00000 | 0.00000 | 0.00000 | 0.00000 | 0.00000 | 0.00000 | 0.00000 | 0.00000 | 0.10000 | 0.16667 |
| 5-049 | 0.00000 | 0.00000 | 0.00000 | 0.00000 | 0.00000 | 0.00000 | 0.00000 | 0.00000 | 0.00000 | 0.00000 | 0.00000 | 0.25000 | 0.10000 | 0.00000 | 0.00000 | 0.00000 | 0.00000 | 0.00000 | 0.00000 | 0.16667 |
| 5-050 | 0.00000 | 0.00000 | 0.09091 | 0.00000 | 0.00000 | 0.00000 | 0.00000 | 0.00000 | 0.16667 | 0.00000 | 0.00000 | 0.16667 | 0.10000 | 0.00000 | 0.00000 | 0.00000 | 0.08333 | 0.00000 | 0.10000 | 0.33333 |
| 5-051 | 0.00000 | 0.00000 | 0.00000 | 0.00000 | 0.00000 | 0.00000 | 0.00000 | 0.00000 | 0.08333 | 0.00000 | 0.00000 | 0.08333 | 0.10000 | 0.08333 | 0.10000 | 0.10000 | 0.08333 | 0.00000 | 0.00000 | 0.16667 |
| 5-052 | 0.00000 | 0.10000 | 0.09091 | 0.08333 | 0.08333 | 0.33333 | 0.45455 | 0.50000 | 0.25000 | 0.16667 | 0.10000 | 0.08333 | 0.00000 | 0.16667 | 0.10000 | 0.20000 | 0.08333 | 0.10000 | 0.00000 | 0.66667 |
| 5-053 | 0.00000 | 0.20000 | 0.09091 | 0.08333 | 0.08333 | 0.50000 | 0.63636 | 0.50000 | 0.33333 | 0.00000 | 0.20000 | 0.41667 | 0.10000 | 0.16667 | 0.10000 | 0.30000 | 0.33333 | 0.40000 | 0.30000 | 0.83333 |
| 5-054 | 0.00000 | 0.10000 | 0.09091 | 0.00000 | 0.00000 | 0.16667 | 0.09091 | 0.00000 | 0.00000 | 0.00000 | 0.00000 | 0.41667 | 0.10000 | 0.08333 | 0.50000 | 0.10000 | 0.41667 | 0.40000 | 0.30000 | 0.50000 |
| 5-055 | 0.00000 | 0.10000 | 0.18182 | 0.00000 | 0.00000 | 0.00000 | 0.09091 | 0.16667 | 0.00000 | 0.33333 | 0.00000 | 0.16667 | 0.00000 | 0.08333 | 0.20000 | 0.30000 | 0.08333 | 0.00000 | 0.00000 | 0.33333 |
| 5-056 | 0.00000 | 0.00000 | 0.00000 | 0.00000 | 0.00000 | 0.00000 | 0.00000 | 0.00000 | 0.08333 | 0.00000 | 0.00000 | 0.25000 | 0.00000 | 0.00000 | 0.00000 | 0.10000 | 0.16667 | 0.00000 | 0.00000 | 0.16667 |
| 5-057 | 0.00000 | 0.00000 | 0.00000 | 0.00000 | 0.00000 | 0.00000 | 0.00000 | 0.16667 | 0.00000 | 0.00000 | 0.00000 | 0.25000 | 0.00000 | 0.00000 | 0.10000 | 0.10000 | 0.25000 | 0.20000 | 0.20000 | 0.83333 |
| 5-058 | 0.00000 | 0.00000 | 0.00000 | 0.00000 | 0.00000 | 0.00000 | 0.09091 | 0.16667 | 0.00000 | 0.00000 | 0.00000 | 0.08333 | 0.00000 | 0.08333 | 0.00000 | 0.10000 | 0.00000 | 0.00000 | 0.10000 | 0.33333 |
| 5-059 | 0.00000 | 0.00000 | 0.00000 | 0.00000 | 0.08333 | 0.00000 | 0.00000 | 0.00000 | 0.00000 | 0.00000 | 0.00000 | 0.00000 | 0.00000 | 0.00000 | 0.10000 | 0.00000 | 0.00000 | 0.00000 | 0.00000 | 0.00000 |
| 5-060 | 0.00000 | 0.00000 | 0.00000 | 0.00000 | 0.00000 | 0.00000 | 0.00000 | 0.00000 | 0.00000 | 0.00000 | 0.00000 | 0.08333 | 0.10000 | 0.00000 | 0.10000 | 0.00000 | 0.00000 | 0.00000 | 0.00000 | 0.00000 |
| 5-061 | 0.00000 | 0.10000 | 0.09091 | 0.00000 | 0.00000 | 0.00000 | 0.09091 | 0.00000 | 0.00000 | 0.00000 | 0.10000 | 0.25000 | 0.00000 | 0.00000 | 0.10000 | 0.00000 | 0.08333 | 0.00000 | 0.00000 | 0.33333 |
| 5-062 | 0.00000 | 0.30000 | 0.00000 | 0.33333 | 0.16667 | 0.00000 | 0.27273 | 0.00000 | 0.16667 | 0.00000 | 0.40000 | 0.66667 | 0.00000 | 0.50000 | 0.30000 | 0.80000 | 0.66667 | 0.60000 | 0.40000 | 0.50000 |
| 5-063 | 0.00000 | 0.00000 | 0.00000 | 0.00000 | 0.00000 | 0.00000 | 0.00000 | 0.16667 | 0.08333 | 0.00000 | 0.00000 | 0.08333 | 0.00000 | 0.08333 | 0.10000 | 0.10000 | 0.08333 | 0.10000 | 0.00000 | 0.66667 |
| 5-064 | 0.00000 | 0.00000 | 0.00000 | 0.00000 | 0.00000 | 0.00000 | 0.00000 | 0.16667 | 0.08333 | 0.00000 | 0.00000 | 0.00000 | 0.00000 | 0.08333 | 0.00000 | 0.10000 | 0.00000 | 0.10000 | 0.10000 | 0.66667 |
| 5-065 | 0.00000 | 0.00000 | 0.00000 | 0.00000 | 0.00000 | 0.00000 | 0.00000 | 0.00000 | 0.00000 | 0.00000 | 0.00000 | 0.08333 | 0.10000 | 0.08333 | 0.00000 | 0.10000 | 0.08333 | 0.00000 | 0.00000 | 0.00000 |
| 5-066 | 0.00000 | 0.00000 | 0.00000 | 0.00000 | 0.00000 | 0.00000 | 0.00000 | 0.00000 | 0.00000 | 0.00000 | 0.00000 | 0.08333 | 0.10000 | 0.08333 | 0.10000 | 0.00000 | 0.08333 | 0.10000 | 0.00000 | 0.50000 |
| 5-067 | 0.00000 | 0.00000 | 0.09091 | 0.08333 | 0.08333 | 0.00000 | 0.00000 | 0.00000 | 0.08333 | 0.00000 | 0.00000 | 0.00000 | 0.10000 | 0.16667 | 0.00000 | 0.10000 | 0.08333 | 0.00000 | 0.00000 | 0.00000 |
| 5-068 | 0.00000 | 0.10000 | 0.00000 | 0.00000 | 0.00000 | 0.00000 | 0.18182 | 0.00000 | 0.16667 | 0.00000 | 0.20000 | 0.08333 | 0.10000 | 0.00000 | 0.00000 | 0.10000 | 0.00000 | 0.10000 | 0.00000 | 0.33333 |
| 5-069 | 0.00000 | 0.00000 | 0.00000 | 0.00000 | 0.00000 | 0.00000 | 0.00000 | 0.00000 | 0.00000 | 0.00000 | 0.00000 | 0.08333 | 0.00000 | 0.00000 | 0.00000 | 0.00000 | 0.00000 | 0.00000 | 0.10000 | 0.00000 |
| 5-070 | 0.00000 | 0.00000 | 0.00000 | 0.00000 | 0.00000 | 0.00000 | 0.00000 | 0.00000 | 0.00000 | 0.00000 | 0.00000 | 0.08333 | 0.00000 | 0.00000 | 0.00000 | 0.10000 | 0.00000 | 0.00000 | 0.10000 | 0.00000 |
| 5-071 | 0.00000 | 0.00000 | 0.09091 | 0.00000 | 0.00000 | 0.00000 | 0.00000 | 0.00000 | 0.00000 | 0.00000 | 0.00000 | 0.00000 | 0.00000 | 0.08333 | 0.00000 | 0.00000 | 0.00000 | 0.00000 | 0.10000 | 0.00000 |
| 5-072 | 0.00000 | 0.00000 | 0.00000 | 0.08333 | 0.16667 | 0.00000 | 0.00000 | 0.00000 | 0.00000 | 0.00000 | 0.00000 | 0.08333 | 0.00000 | 0.00000 | 0.00000 | 0.00000 | 0.25000 | 0.00000 | 0.60000 | 0.33333 |
| 5-073 | 0.00000 | 0.10000 | 0.09091 | 0.08333 | 0.16667 | 0.00000 | 0.00000 | 0.00000 | 0.08333 | 0.00000 | 0.00000 | 0.00000 | 0.00000 | 0.00000 | 0.00000 | 0.00000 | 0.33333 | 0.00000 | 0.30000 | 0.16667 |
| 5-074 | 0.00000 | 0.00000 | 0.00000 | 0.00000 | 0.00000 | 0.00000 | 0.00000 | 0.00000 | 0.00000 | 0.00000 | 0.00000 | 0.08333 | 0.00000 | 0.00000 | 0.00000 | 0.00000 | 0.08333 | 0.00000 | 0.10000 | 0.33333 |
| 5-075 | 0.00000 | 0.00000 | 0.00000 | 0.00000 | 0.08333 | 0.00000 | 0.00000 | 0.00000 | 0.08333 | 0.00000 | 0.00000 | 0.00000 | 0.00000 | 0.00000 | 0.10000 | 0.10000 | 0.00000 | 0.30000 | 0.20000 | 0.50000 |
| 5-076 | 0.00000 | 0.00000 | 0.00000 | 0.00000 | 0.00000 | 0.00000 | 0.00000 | 0.00000 | 0.08333 | 0.00000 | 0.00000 | 0.08333 | 0.00000 | 0.00000 | 0.00000 | 0.00000 | 0.00000 | 0.00000 | 0.00000 | 0.16667 |
| 5-077 | 0.00000 | 0.00000 | 0.00000 | 0.08333 | 0.08333 | 0.00000 | 0.00000 | 0.00000 | 0.08333 | 0.00000 | 0.00000 | 0.16667 | 0.20000 | 0.00000 | 0.10000 | 0.00000 | 0.25000 | 0.00000 | 0.30000 | 0.33333 |
| 5-078 | 0.00000 | 0.00000 | 0.00000 | 0.00000 | 0.08333 | 0.00000 | 0.00000 | 0.00000 | 0.08333 | 0.00000 | 0.00000 | 0.25000 | 0.30000 | 0.33333 | 0.20000 | 0.20000 | 0.00000 | 0.00000 | 0.30000 | 0.50000 |
| 5-079 | 0.00000 | 0.00000 | 0.00000 | 0.00000 | 0.00000 | 0.00000 | 0.00000 | 0.00000 | 0.00000 | 0.00000 | 0.00000 | 0.00000 | 0.30000 | 0.08333 | 0.10000 | 0.20000 | 0.08333 | 0.00000 | 0.50000 | 0.33333 |
| 5-080 | 0.00000 | 0.10000 | 0.00000 | 0.00000 | 0.00000 | 0.00000 | 0.00000 | 0.00000 | 0.08333 | 0.00000 | 0.00000 | 0.08333 | 0.00000 | 0.08333 | 0.00000 | 0.20000 | 0.00000 | 0.00000 | 0.00000 | 0.50000 |
| 5-081 | 0.14286 | 0.10000 | 0.00000 | 0.00000 | 0.08333 | 0.00000 | 0.00000 | 0.00000 | 0.00000 | 0.00000 | 0.00000 | 0.16667 | 0.00000 | 0.08333 | 0.00000 | 0.20000 | 0.00000 | 0.00000 | 0.00000 | 0.66667 |
| 5-082 | 0.00000 | 0.00000 | 0.00000 | 0.00000 | 0.08333 | 0.00000 | 0.00000 | 0.00000 | 0.08333 | 0.00000 | 0.00000 | 0.08333 | 0.00000 | 0.00000 | 0.00000 | 0.00000 | 0.00000 | 0.00000 | 0.00000 | 0.00000 |
| 5-083 | 0.00000 | 0.10000 | 0.27273 | 0.33333 | 0.33333 | 0.00000 | 0.00000 | 0.16667 | 0.08333 | 0.00000 | 0.10000 | 0.58333 | 0.70000 | 0.50000 | 0.60000 | 0.40000 | 0.75000 | 0.60000 | 0.80000 | 0.33333 |
| 5-084 | 0.00000 | 0.10000 | 0.27273 | 0.08333 | 0.00000 | 0.00000 | 0.09091 | 0.00000 | 0.08333 | 0.33333 | 0.10000 | 0.08333 | 0.10000 | 0.16667 | 0.00000 | 0.30000 | 0.00000 | 0.20000 | 0.00000 | 0.66667 |
| 5-085 | 0.00000 | 0.00000 | 0.00000 | 0.00000 | 0.00000 | 0.00000 | 0.00000 | 0.00000 | 0.00000 | 0.00000 | 0.00000 | 0.08333 | 0.00000 | 0.00000 | 0.00000 | 0.00000 | 0.00000 | 0.00000 | 0.00000 | 0.00000 |
| 5-086 | 0.00000 | 0.20000 | 0.00000 | 0.00000 | 0.00000 | 0.16667 | 0.00000 | 0.16667 | 0.00000 | 0.00000 | 0.00000 | 0.16667 | 0.00000 | 0.00000 | 0.10000 | 0.00000 | 0.25000 | 0.00000 | 0.10000 | 0.00000 |
| 5-087 | 0.00000 | 0.10000 | 0.00000 | 0.00000 | 0.00000 | 0.16667 | 0.00000 | 0.16667 | 0.00000 | 0.00000 | 0.00000 | 0.25000 | 0.00000 | 0.00000 | 0.10000 | 0.00000 | 0.16667 | 0.00000 | 0.10000 | 0.00000 |
| 5-088 | 0.00000 | 0.00000 | 0.00000 | 0.00000 | 0.00000 | 0.00000 | 0.00000 | 0.00000 | 0.16667 | 0.00000 | 0.00000 | 0.00000 | 0.00000 | 0.16667 | 0.00000 | 0.10000 | 0.08333 | 0.00000 | 0.00000 | 0.83333 |
| 5-089 | 0.00000 | 0.00000 | 0.09091 | 0.00000 | 0.00000 | 0.00000 | 0.00000 | 0.00000 | 0.00000 | 0.00000 | 0.00000 | 0.16667 | 0.00000 | 0.00000 | 0.00000 | 0.00000 | 0.00000 | 0.00000 | 0.00000 | 0.00000 |
| 5-090 | 0.00000 | 0.00000 | 0.09091 | 0.00000 | 0.00000 | 0.16667 | 0.00000 | 0.00000 | 0.00000 | 0.00000 | 0.00000 | 0.00000 | 0.00000 | 0.00000 | 0.00000 | 0.00000 | 0.00000 | 0.00000 | 0.00000 | 0.00000 |
| 5-091 | 0.00000 | 0.00000 | 0.00000 | 0.00000 | 0.00000 | 0.00000 | 0.00000 | 0.00000 | 0.08333 | 0.00000 | 0.00000 | 0.00000 | 0.00000 | 0.00000 | 0.00000 | 0.00000 | 0.00000 | 0.00000 | 0.00000 | 0.00000 |
| 5-092 | 0.00000 | 0.00000 | 0.00000 | 0.00000 | 0.00000 | 0.00000 | 0.00000 | 0.00000 | 0.08333 | 0.00000 | 0.00000 | 0.00000 | 0.00000 | 0.00000 | 0.00000 | 0.00000 | 0.00000 | 0.00000 | 0.00000 | 0.00000 |
| 5-093 | 0.00000 | 0.00000 | 0.00000 | 0.00000 | 0.00000 | 0.00000 | 0.00000 | 0.00000 | 0.08333 | 0.00000 | 0.00000 | 0.00000 | 0.00000 | 0.00000 | 0.00000 | 0.00000 | 0.16667 | 0.40000 | 0.00000 | 0.16667 |
| 5-094 | 0.00000 | 0.00000 | 0.00000 | 0.00000 | 0.00000 | 0.16667 | 0.00000 | 0.16667 | 0.00000 | 0.00000 | 0.00000 | 0.00000 | 0.10000 | 0.16667 | 0.00000 | 0.00000 | 0.08333 | 0.00000 | 0.00000 | 0.16667 |
| 5-095 | 0.00000 | 0.00000 | 0.00000 | 0.00000 | 0.00000 | 0.00000 | 0.00000 | 0.16667 | 0.00000 | 0.00000 | 0.00000 | 0.00000 | 0.00000 | 0.00000 | 0.00000 | 0.00000 | 0.00000 | 0.00000 | 0.00000 | 0.33333 |
| 5-096 | 0.00000 | 0.00000 | 0.00000 | 0.00000 | 0.00000 | 0.16667 | 0.00000 | 0.00000 | 0.00000 | 0.00000 | 0.00000 | 0.00000 | 0.10000 | 0.16667 | 0.00000 | 0.00000 | 0.00000 | 0.00000 | 0.00000 | 0.00000 |
| 5-097 | 0.00000 | 0.00000 | 0.00000 | 0.00000 | 0.00000 | 0.00000 | 0.00000 | 0.00000 | 0.16667 | 0.00000 | 0.00000 | 0.00000 | 0.10000 | 0.00000 | 0.10000 | 0.00000 | 0.33333 | 0.10000 | 0.30000 | 0.66667 |
| 5-098 | 0.00000 | 0.00000 | 0.00000 | 0.00000 | 0.00000 | 0.00000 | 0.00000 | 0.00000 | 0.16667 | 0.00000 | 0.10000 | 0.00000 | 0.10000 | 0.08333 | 0.10000 | 0.00000 | 0.33333 | 0.10000 | 0.30000 | 0.66667 |
| 5-099 | 0.00000 | 0.00000 | 0.00000 | 0.00000 | 0.00000 | 0.00000 | 0.00000 | 0.00000 | 0.00000 | 0.00000 | 0.10000 | 0.00000 | 0.00000 | 0.00000 | 0.00000 | 0.00000 | 0.08333 | 0.00000 | 0.00000 | 0.00000 |
| 5-100 | 0.00000 | 0.00000 | 0.00000 | 0.00000 | 0.00000 | 0.00000 | 0.00000 | 0.00000 | 0.00000 | 0.00000 | 0.00000 | 0.00000 | 0.00000 | 0.00000 | 0.00000 | 0.00000 | 0.08333 | 0.60000 | 0.20000 | 0.00000 |
| 5-101 | 0.00000 | 0.00000 | 0.00000 | 0.00000 | 0.00000 | 0.00000 | 0.00000 | 0.00000 | 0.00000 | 0.00000 | 0.00000 | 0.08333 | 0.00000 | 0.00000 | 0.00000 | 0.00000 | 0.00000 | 0.00000 | 0.00000 | 0.00000 |
| 5-102 | 0.00000 | 0.00000 | 0.00000 | 0.00000 | 0.00000 | 0.00000 | 0.18182 | 0.00000 | 0.00000 | 0.00000 | 0.00000 | 0.00000 | 0.00000 | 0.00000 | 0.00000 | 0.10000 | 0.16667 | 0.30000 | 0.00000 | 0.00000 |
| 5-103 | 0.00000 | 0.00000 | 0.00000 | 0.00000 | 0.00000 | 0.00000 | 0.09091 | 0.00000 | 0.00000 | 0.00000 | 0.00000 | 0.00000 | 0.00000 | 0.00000 | 0.00000 | 0.10000 | 0.08333 | 0.30000 | 0.00000 | 0.00000 |
| 5-104 | 0.00000 | 0.00000 | 0.18182 | 0.08333 | 0.16667 | 0.00000 | 0.00000 | 0.00000 | 0.16667 | 0.00000 | 0.00000 | 0.16667 | 0.00000 | 0.00000 | 0.00000 | 0.20000 | 0.00000 | 0.00000 | 0.10000 | 0.00000 |
| 5-105 | 0.00000 | 0.10000 | 0.18182 | 0.16667 | 0.08333 | 0.00000 | 0.09091 | 0.00000 | 0.16667 | 0.00000 | 0.10000 | 0.08333 | 0.00000 | 0.08333 | 0.00000 | 0.10000 | 0.00000 | 0.10000 | 0.60000 | 0.00000 |
| 5-106 | 0.00000 | 0.10000 | 0.00000 | 0.00000 | 0.00000 | 0.00000 | 0.09091 | 0.00000 | 0.00000 | 0.00000 | 0.10000 | 0.00000 | 0.00000 | 0.08333 | 0.00000 | 0.00000 | 0.00000 | 0.10000 | 0.70000 | 0.00000 |
| 5-107 | 0.00000 | 0.00000 | 0.00000 | 0.00000 | 0.00000 | 0.00000 | 0.00000 | 0.00000 | 0.00000 | 0.00000 | 0.00000 | 0.08333 | 0.00000 | 0.00000 | 0.10000 | 0.10000 | 0.08333 | 0.30000 | 0.00000 | 0.00000 |
| 5-108 | 0.00000 | 0.00000 | 0.00000 | 0.00000 | 0.00000 | 0.00000 | 0.00000 | 0.00000 | 0.00000 | 0.00000 | 0.00000 | 0.00000 | 0.00000 | 0.00000 | 0.10000 | 0.10000 | 0.00000 | 0.30000 | 0.00000 | 0.16667 |
| 5-109 | 0.00000 | 0.10000 | 0.18182 | 0.00000 | 0.08333 | 0.50000 | 0.45455 | 0.50000 | 0.25000 | 0.00000 | 0.20000 | 0.58333 | 0.60000 | 0.66667 | 0.60000 | 0.50000 | 0.66667 | 0.50000 | 0.40000 | 1.00000 |
| 5-110 | 0.00000 | 0.10000 | 0.09091 | 0.00000 | 0.08333 | 0.50000 | 0.45455 | 0.50000 | 0.25000 | 0.00000 | 0.20000 | 0.58333 | 0.60000 | 0.58333 | 0.60000 | 0.50000 | 0.66667 | 0.50000 | 0.40000 | 1.00000 |
| 5-111 | 0.00000 | 0.00000 | 0.00000 | 0.00000 | 0.08333 | 0.00000 | 0.00000 | 0.00000 | 0.08333 | 0.00000 | 0.00000 | 0.08333 | 0.00000 | 0.33333 | 0.00000 | 0.00000 | 0.16667 | 0.10000 | 0.00000 | 0.00000 |
| 5-112 | 0.00000 | 0.00000 | 0.00000 | 0.00000 | 0.00000 | 0.00000 | 0.00000 | 0.00000 | 0.00000 | 0.00000 | 0.00000 | 0.00000 | 0.00000 | 0.00000 | 0.00000 | 0.00000 | 0.00000 | 0.00000 | 0.00000 | 0.16667 |
| 5-113 | 0.00000 | 0.00000 | 0.00000 | 0.00000 | 0.00000 | 0.00000 | 0.00000 | 0.00000 | 0.00000 | 0.00000 | 0.00000 | 0.00000 | 0.00000 | 0.00000 | 0.10000 | 0.00000 | 0.00000 | 0.00000 | 0.00000 | 1.00000 |
| 5-114 | 0.00000 | 0.00000 | 0.00000 | 0.00000 | 0.00000 | 0.00000 | 0.00000 | 0.00000 | 0.00000 | 0.00000 | 0.00000 | 0.00000 | 0.00000 | 0.00000 | 0.00000 | 0.00000 | 0.00000 | 0.10000 | 0.00000 | 1.00000 |
| 5-115 | 0.00000 | 0.00000 | 0.00000 | 0.00000 | 0.00000 | 0.00000 | 0.00000 | 0.00000 | 0.00000 | 0.00000 | 0.00000 | 0.00000 | 0.00000 | 0.00000 | 0.00000 | 0.00000 | 0.00000 | 0.10000 | 0.00000 | 1.00000 |
| 5-116 | 0.00000 | 0.00000 | 0.00000 | 0.00000 | 0.00000 | 0.00000 | 0.00000 | 0.00000 | 0.00000 | 0.00000 | 0.00000 | 0.00000 | 0.00000 | 0.00000 | 0.00000 | 0.00000 | 0.00000 | 0.00000 | 0.10000 | 0.00000 |
| 5-117 | 0.00000 | 0.00000 | 0.00000 | 0.00000 | 0.00000 | 0.00000 | 0.00000 | 0.00000 | 0.00000 | 0.00000 | 0.00000 | 0.00000 | 0.00000 | 0.00000 | 0.00000 | 0.00000 | 0.00000 | 0.00000 | 0.10000 | 0.00000 |
| 5-118 | 0.00000 | 0.00000 | 0.00000 | 0.00000 | 0.00000 | 0.00000 | 0.00000 | 0.00000 | 0.00000 | 0.00000 | 0.00000 | 0.00000 | 0.20000 | 0.00000 | 0.00000 | 0.00000 | 0.00000 | 0.00000 | 0.20000 | 0.16667 |
| 5-119 | 0.00000 | 0.00000 | 0.00000 | 0.00000 | 0.00000 | 0.00000 | 0.00000 | 0.00000 | 0.00000 | 0.00000 | 0.00000 | 0.08333 | 0.00000 | 0.00000 | 0.00000 | 0.00000 | 0.00000 | 0.00000 | 0.00000 | 0.00000 |
| 5-120 | 0.00000 | 0.00000 | 0.00000 | 0.00000 | 0.00000 | 0.00000 | 0.00000 | 0.00000 | 0.00000 | 0.00000 | 0.00000 | 0.00000 | 0.10000 | 0.00000 | 0.10000 | 0.00000 | 0.00000 | 0.30000 | 0.00000 | 0.83333 |
| 5-121 | 0.00000 | 0.00000 | 0.00000 | 0.00000 | 0.00000 | 0.00000 | 0.00000 | 0.00000 | 0.00000 | 0.00000 | 0.00000 | 0.00000 | 0.00000 | 0.00000 | 0.00000 | 0.00000 | 0.08333 | 0.10000 | 0.00000 | 0.00000 |
| 5-122 | 1.00000 | 0.00000 | 0.00000 | 0.00000 | 0.00000 | 0.00000 | 0.00000 | 0.00000 | 0.00000 | 0.00000 | 0.00000 | 0.00000 | 0.00000 | 0.00000 | 0.00000 | 0.00000 | 0.00000 | 0.00000 | 0.00000 | 0.00000 |
| 5-123 | 0.00000 | 0.00000 | 0.00000 | 0.00000 | 0.00000 | 0.00000 | 0.00000 | 0.00000 | 0.00000 | 0.16667 | 0.00000 | 0.00000 | 0.00000 | 0.00000 | 0.00000 | 0.00000 | 0.00000 | 0.00000 | 0.00000 | 0.00000 |
| 5-124 | 0.00000 | 0.00000 | 0.00000 | 0.00000 | 0.00000 | 0.00000 | 0.00000 | 0.16667 | 0.00000 | 0.00000 | 0.00000 | 0.00000 | 0.00000 | 0.00000 | 0.00000 | 0.00000 | 0.00000 | 0.00000 | 0.00000 | 0.00000 |
| 6-001 | 0.00000 | 0.00000 | 0.00000 | 0.00000 | 0.00000 | 0.00000 | 0.00000 | 0.00000 | 0.00000 | 0.00000 | 0.00000 | 0.00000 | 0.00000 | 0.00000 | 0.00000 | 0.00000 | 0.00000 | 0.00000 | 0.00000 | 0.16667 |
| 6-002 | 0.00000 | 0.00000 | 0.00000 | 0.00000 | 0.00000 | 0.00000 | 0.00000 | 0.00000 | 0.00000 | 0.00000 | 0.00000 | 0.00000 | 0.00000 | 0.00000 | 0.00000 | 0.00000 | 0.00000 | 0.00000 | 0.10000 | 0.33333 |
| 6-003 | 0.12500 | 0.00000 | 0.00000 | 0.00000 | 0.00000 | 0.00000 | 0.00000 | 0.00000 | 0.00000 | 0.00000 | 0.16667 | 0.00000 | 0.00000 | 0.00000 | 0.00000 | 0.00000 | 0.00000 | 0.00000 | 0.10000 | 0.00000 |
| 6-004 | 0.12500 | 0.00000 | 0.00000 | 0.00000 | 0.00000 | 0.00000 | 0.00000 | 0.00000 | 0.00000 | 0.00000 | 0.00000 | 0.00000 | 0.00000 | 0.00000 | 0.00000 | 0.00000 | 0.00000 | 0.00000 | 0.00000 | 0.33333 |
| 6-005 | 0.00000 | 0.00000 | 0.00000 | 0.00000 | 0.00000 | 0.00000 | 0.00000 | 0.00000 | 0.00000 | 0.00000 | 0.00000 | 0.00000 | 0.00000 | 0.00000 | 0.00000 | 0.00000 | 0.08333 | 0.10000 | 0.10000 | 0.33333 |
| 6-006 | 0.00000 | 0.10000 | 0.00000 | 0.00000 | 0.00000 | 0.00000 | 0.00000 | 0.00000 | 0.00000 | 0.00000 | 0.00000 | 0.00000 | 0.00000 | 0.00000 | 0.10000 | 0.00000 | 0.00000 | 0.00000 | 0.10000 | 0.16667 |
| 6-007 | 0.00000 | 0.10000 | 0.08333 | 0.00000 | 0.00000 | 0.00000 | 0.00000 | 0.00000 | 0.00000 | 0.00000 | 0.00000 | 0.00000 | 0.00000 | 0.00000 | 0.00000 | 0.00000 | 0.00000 | 0.00000 | 0.10000 | 0.00000 |
| 6-008 | 0.00000 | 0.00000 | 0.00000 | 0.00000 | 0.00000 | 0.00000 | 0.00000 | 0.00000 | 0.00000 | 0.00000 | 0.00000 | 0.00000 | 0.10000 | 0.00000 | 0.00000 | 0.00000 | 0.00000 | 0.00000 | 0.00000 | 0.16667 |
| 6-009 | 0.00000 | 0.00000 | 0.00000 | 0.00000 | 0.00000 | 0.00000 | 0.00000 | 0.00000 | 0.00000 | 0.00000 | 0.00000 | 0.00000 | 0.00000 | 0.00000 | 0.00000 | 0.00000 | 0.00000 | 0.00000 | 0.10000 | 0.16667 |
| 6-010 | 0.00000 | 0.00000 | 0.00000 | 0.00000 | 0.00000 | 0.16667 | 0.08333 | 0.00000 | 0.00000 | 0.00000 | 0.00000 | 0.00000 | 0.00000 | 0.00000 | 0.00000 | 0.00000 | 0.00000 | 0.00000 | 0.10000 | 0.33333 |
| 6-011 | 0.00000 | 0.00000 | 0.00000 | 0.00000 | 0.00000 | 0.16667 | 0.00000 | 0.00000 | 0.00000 | 0.00000 | 0.00000 | 0.00000 | 0.00000 | 0.00000 | 0.00000 | 0.00000 | 0.08333 | 0.00000 | 0.10000 | 0.16667 |
| 6-012 | 0.00000 | 0.10000 | 0.00000 | 0.00000 | 0.00000 | 0.00000 | 0.00000 | 0.00000 | 0.00000 | 0.00000 | 0.00000 | 0.00000 | 0.10000 | 0.00000 | 0.00000 | 0.00000 | 0.00000 | 0.10000 | 0.10000 | 0.16667 |
| 6-013 | 0.00000 | 0.00000 | 0.00000 | 0.00000 | 0.00000 | 0.00000 | 0.00000 | 0.00000 | 0.00000 | 0.00000 | 0.00000 | 0.00000 | 0.00000 | 0.00000 | 0.00000 | 0.00000 | 0.00000 | 0.00000 | 0.00000 | 0.16667 |
| 6-014 | 0.00000 | 0.00000 | 0.00000 | 0.00000 | 0.00000 | 0.00000 | 0.00000 | 0.00000 | 0.00000 | 0.00000 | 0.00000 | 0.00000 | 0.00000 | 0.00000 | 0.10000 | 0.00000 | 0.00000 | 0.00000 | 0.00000 | 0.16667 |
| 6-015 | 0.00000 | 0.00000 | 0.00000 | 0.00000 | 0.00000 | 0.00000 | 0.00000 | 0.00000 | 0.00000 | 0.00000 | 0.00000 | 0.00000 | 0.00000 | 0.00000 | 0.10000 | 0.00000 | 0.00000 | 0.00000 | 0.10000 | 0.33333 |
| 6-016 | 0.00000 | 0.00000 | 0.00000 | 0.00000 | 0.00000 | 0.00000 | 0.00000 | 0.00000 | 0.00000 | 0.00000 | 0.00000 | 0.00000 | 0.00000 | 0.00000 | 0.00000 | 0.00000 | 0.00000 | 0.00000 | 0.00000 | 0.33333 |
| 6-017 | 0.00000 | 0.00000 | 0.00000 | 0.00000 | 0.00000 | 0.00000 | 0.00000 | 0.00000 | 0.00000 | 0.00000 | 0.00000 | 0.00000 | 0.00000 | 0.00000 | 0.00000 | 0.00000 | 0.00000 | 0.00000 | 0.10000 | 0.33333 |
| 6-018 | 0.00000 | 0.00000 | 0.00000 | 0.08333 | 0.00000 | 0.00000 | 0.00000 | 0.00000 | 0.00000 | 0.00000 | 0.00000 | 0.00000 | 0.00000 | 0.08333 | 0.10000 | 0.00000 | 0.08333 | 0.00000 | 0.00000 | 0.00000 |
| 6-019 | 0.00000 | 0.00000 | 0.00000 | 0.00000 | 0.00000 | 0.16667 | 0.00000 | 0.00000 | 0.00000 | 0.00000 | 0.00000 | 0.00000 | 0.00000 | 0.00000 | 0.10000 | 0.00000 | 0.08333 | 0.00000 | 0.10000 | 0.16667 |
| 6-020 | 0.00000 | 0.00000 | 0.00000 | 0.00000 | 0.00000 | 0.16667 | 0.00000 | 0.00000 | 0.00000 | 0.00000 | 0.00000 | 0.00000 | 0.00000 | 0.00000 | 0.10000 | 0.00000 | 0.00000 | 0.10000 | 0.00000 | 0.00000 |
| 6-021 | 0.00000 | 0.00000 | 0.00000 | 0.00000 | 0.00000 | 0.00000 | 0.00000 | 0.00000 | 0.00000 | 0.00000 | 0.00000 | 0.00000 | 0.00000 | 0.00000 | 0.10000 | 0.10000 | 0.00000 | 0.00000 | 0.20000 | 0.16667 |
| 6-022 | 0.00000 | 0.00000 | 0.00000 | 0.16667 | 0.25000 | 0.16667 | 0.08333 | 0.16667 | 0.16667 | 0.00000 | 0.16667 | 0.08333 | 0.00000 | 0.00000 | 0.30000 | 0.10000 | 0.16667 | 0.30000 | 0.10000 | 0.00000 |
| 6-023 | 0.00000 | 0.00000 | 0.00000 | 0.08333 | 0.08333 | 0.00000 | 0.00000 | 0.00000 | 0.00000 | 0.00000 | 0.08333 | 0.00000 | 0.00000 | 0.00000 | 0.10000 | 0.00000 | 0.00000 | 0.00000 | 0.10000 | 0.16667 |
| 6-024 | 0.12500 | 0.00000 | 0.00000 | 0.00000 | 0.00000 | 0.00000 | 0.00000 | 0.00000 | 0.00000 | 0.00000 | 0.00000 | 0.00000 | 0.00000 | 0.08333 | 0.00000 | 0.00000 | 0.00000 | 0.00000 | 0.20000 | 0.16667 |
| 6-025 | 0.00000 | 0.00000 | 0.08333 | 0.08333 | 0.00000 | 0.00000 | 0.00000 | 0.00000 | 0.00000 | 0.00000 | 0.00000 | 0.00000 | 0.10000 | 0.08333 | 0.10000 | 0.00000 | 0.00000 | 0.10000 | 0.10000 | 0.33333 |
| 6-026 | 0.00000 | 0.10000 | 0.00000 | 0.08333 | 0.00000 | 0.00000 | 0.00000 | 0.00000 | 0.00000 | 0.00000 | 0.00000 | 0.00000 | 0.00000 | 0.08333 | 0.00000 | 0.00000 | 0.00000 | 0.00000 | 0.00000 | 0.16667 |
| 6-027 | 0.12500 | 0.00000 | 0.00000 | 0.00000 | 0.00000 | 0.00000 | 0.00000 | 0.00000 | 0.00000 | 0.00000 | 0.00000 | 0.08333 | 0.00000 | 0.08333 | 0.10000 | 0.10000 | 0.08333 | 0.00000 | 0.40000 | 0.33333 |
| 6-028 | 0.00000 | 0.00000 | 0.00000 | 0.00000 | 0.00000 | 0.00000 | 0.00000 | 0.00000 | 0.00000 | 0.00000 | 0.00000 | 0.00000 | 0.00000 | 0.00000 | 0.00000 | 0.00000 | 0.00000 | 0.00000 | 0.00000 | 0.33333 |
| 6-029 | 0.00000 | 0.00000 | 0.00000 | 0.08333 | 0.16667 | 0.50000 | 0.33333 | 0.33333 | 0.08333 | 0.00000 | 0.08333 | 0.00000 | 0.00000 | 0.00000 | 0.00000 | 0.00000 | 0.25000 | 0.00000 | 0.20000 | 0.50000 |
| 6-030 | 0.00000 | 0.00000 | 0.00000 | 0.00000 | 0.08333 | 0.00000 | 0.00000 | 0.16667 | 0.00000 | 0.00000 | 0.00000 | 0.08333 | 0.00000 | 0.00000 | 0.30000 | 0.00000 | 0.08333 | 0.00000 | 0.20000 | 0.16667 |
| 6-031 | 0.00000 | 0.00000 | 0.00000 | 0.00000 | 0.00000 | 0.00000 | 0.08333 | 0.00000 | 0.00000 | 0.00000 | 0.00000 | 0.08333 | 0.00000 | 0.08333 | 0.00000 | 0.00000 | 0.00000 | 0.10000 | 0.10000 | 0.00000 |
| 6-032 | 0.00000 | 0.10000 | 0.00000 | 0.00000 | 0.00000 | 0.00000 | 0.00000 | 0.16667 | 0.00000 | 0.00000 | 0.08333 | 0.00000 | 0.00000 | 0.00000 | 0.00000 | 0.00000 | 0.00000 | 0.10000 | 0.10000 | 0.00000 |
| 6-033 | 0.00000 | 0.00000 | 0.00000 | 0.00000 | 0.00000 | 0.00000 | 0.00000 | 0.00000 | 0.00000 | 0.00000 | 0.00000 | 0.00000 | 0.00000 | 0.00000 | 0.00000 | 0.00000 | 0.00000 | 0.00000 | 0.10000 | 0.16667 |
| 6-034 | 0.00000 | 0.00000 | 0.00000 | 0.00000 | 0.00000 | 0.00000 | 0.00000 | 0.00000 | 0.00000 | 0.00000 | 0.08333 | 0.00000 | 0.00000 | 0.00000 | 0.10000 | 0.00000 | 0.08333 | 0.10000 | 0.30000 | 0.33333 |
| 6-035 | 0.00000 | 0.00000 | 0.00000 | 0.00000 | 0.00000 | 0.00000 | 0.00000 | 0.00000 | 0.00000 | 0.00000 | 0.00000 | 0.00000 | 0.00000 | 0.00000 | 0.00000 | 0.00000 | 0.00000 | 0.00000 | 0.10000 | 0.00000 |
| 6-036 | 0.00000 | 0.00000 | 0.00000 | 0.00000 | 0.00000 | 0.00000 | 0.00000 | 0.00000 | 0.00000 | 0.00000 | 0.00000 | 0.00000 | 0.00000 | 0.00000 | 0.00000 | 0.00000 | 0.00000 | 0.00000 | 0.00000 | 0.16667 |
| 6-037 | 0.00000 | 0.00000 | 0.00000 | 0.00000 | 0.00000 | 0.00000 | 0.00000 | 0.00000 | 0.00000 | 0.22222 | 0.00000 | 0.08333 | 0.00000 | 0.08333 | 0.10000 | 0.10000 | 0.08333 | 0.00000 | 0.10000 | 0.33333 |
| 6-038 | 0.25000 | 0.10000 | 0.16667 | 0.00000 | 0.16667 | 0.00000 | 0.00000 | 0.16667 | 0.00000 | 0.00000 | 0.16667 | 0.08333 | 0.10000 | 0.00000 | 0.20000 | 0.10000 | 0.08333 | 0.10000 | 0.00000 | 0.00000 |
| 6-039 | 0.12500 | 0.20000 | 0.08333 | 0.16667 | 0.33333 | 0.00000 | 0.08333 | 0.00000 | 0.08333 | 0.00000 | 0.08333 | 0.16667 | 0.00000 | 0.00000 | 0.20000 | 0.00000 | 0.41667 | 0.30000 | 0.20000 | 0.33333 |
| 6-040 | 0.00000 | 0.00000 | 0.00000 | 0.00000 | 0.00000 | 0.00000 | 0.00000 | 0.00000 | 0.00000 | 0.00000 | 0.00000 | 0.00000 | 0.00000 | 0.00000 | 0.00000 | 0.00000 | 0.00000 | 0.10000 | 0.20000 | 0.16667 |
| 6-041 | 0.00000 | 0.00000 | 0.00000 | 0.08333 | 0.00000 | 0.00000 | 0.00000 | 0.00000 | 0.00000 | 0.00000 | 0.00000 | 0.08333 | 0.00000 | 0.00000 | 0.10000 | 0.00000 | 0.08333 | 0.00000 | 0.10000 | 0.16667 |
| 6-042 | 0.00000 | 0.00000 | 0.00000 | 0.00000 | 0.08333 | 0.00000 | 0.00000 | 0.00000 | 0.00000 | 0.00000 | 0.00000 | 0.00000 | 0.00000 | 0.00000 | 0.00000 | 0.00000 | 0.00000 | 0.00000 | 0.10000 | 0.33333 |
| 6-043 | 0.12500 | 0.00000 | 0.00000 | 0.00000 | 0.16667 | 0.00000 | 0.00000 | 0.00000 | 0.00000 | 0.00000 | 0.00000 | 0.00000 | 0.00000 | 0.00000 | 0.20000 | 0.00000 | 0.00000 | 0.10000 | 0.10000 | 0.00000 |
| 6-044 | 0.00000 | 0.00000 | 0.00000 | 0.00000 | 0.00000 | 0.00000 | 0.00000 | 0.00000 | 0.00000 | 0.00000 | 0.00000 | 0.00000 | 0.00000 | 0.00000 | 0.00000 | 0.00000 | 0.00000 | 0.00000 | 0.20000 | 0.50000 |
| 6-045 | 0.00000 | 0.00000 | 0.00000 | 0.00000 | 0.00000 | 0.00000 | 0.00000 | 0.00000 | 0.00000 | 0.00000 | 0.08333 | 0.00000 | 0.00000 | 0.08333 | 0.10000 | 0.00000 | 0.00000 | 0.00000 | 0.00000 | 0.16667 |
| 6-046 | 0.00000 | 0.00000 | 0.00000 | 0.00000 | 0.00000 | 0.00000 | 0.00000 | 0.00000 | 0.00000 | 0.00000 | 0.00000 | 0.00000 | 0.00000 | 0.00000 | 0.00000 | 0.00000 | 0.00000 | 0.10000 | 0.10000 | 0.50000 |
| 6-047 | 0.00000 | 0.10000 | 0.08333 | 0.08333 | 0.08333 | 0.00000 | 0.00000 | 0.00000 | 0.00000 | 0.00000 | 0.00000 | 0.00000 | 0.20000 | 0.00000 | 0.40000 | 0.00000 | 0.16667 | 0.10000 | 0.20000 | 0.33333 |
| 6-048 | 0.00000 | 0.00000 | 0.08333 | 0.00000 | 0.08333 | 0.16667 | 0.25000 | 0.16667 | 0.08333 | 0.11111 | 0.00000 | 0.00000 | 0.00000 | 0.08333 | 0.00000 | 0.00000 | 0.08333 | 0.20000 | 0.20000 | 0.16667 |
| 6-049 | 0.00000 | 0.10000 | 0.08333 | 0.00000 | 0.16667 | 0.00000 | 0.16667 | 0.00000 | 0.08333 | 0.22222 | 0.00000 | 0.00000 | 0.00000 | 0.00000 | 0.00000 | 0.00000 | 0.08333 | 0.20000 | 0.00000 | 0.16667 |
| 6-050 | 0.00000 | 0.00000 | 0.00000 | 0.00000 | 0.08333 | 0.00000 | 0.00000 | 0.00000 | 0.00000 | 0.00000 | 0.00000 | 0.00000 | 0.00000 | 0.08333 | 0.00000 | 0.00000 | 0.00000 | 0.10000 | 0.00000 | 0.00000 |
| 6-051 | 0.00000 | 0.00000 | 0.00000 | 0.00000 | 0.00000 | 0.00000 | 0.00000 | 0.00000 | 0.00000 | 0.00000 | 0.00000 | 0.00000 | 0.00000 | 0.00000 | 0.10000 | 0.10000 | 0.00000 | 0.00000 | 0.00000 | 0.00000 |
| 6-052 | 0.00000 | 0.00000 | 0.08333 | 0.08333 | 0.00000 | 0.00000 | 0.00000 | 0.00000 | 0.00000 | 0.00000 | 0.08333 | 0.00000 | 0.10000 | 0.08333 | 0.00000 | 0.10000 | 0.00000 | 0.10000 | 0.00000 | 0.00000 |
| 6-053 | 0.00000 | 0.00000 | 0.00000 | 0.00000 | 0.00000 | 0.00000 | 0.00000 | 0.00000 | 0.00000 | 0.00000 | 0.00000 | 0.00000 | 0.10000 | 0.00000 | 0.00000 | 0.00000 | 0.00000 | 0.10000 | 0.00000 | 0.16667 |
| 6-054 | 0.00000 | 0.00000 | 0.00000 | 0.00000 | 0.00000 | 0.00000 | 0.00000 | 0.00000 | 0.08333 | 0.11111 | 0.00000 | 0.00000 | 0.00000 | 0.08333 | 0.00000 | 0.00000 | 0.00000 | 0.00000 | 0.10000 | 0.16667 |
| 6-055 | 0.00000 | 0.00000 | 0.00000 | 0.00000 | 0.00000 | 0.00000 | 0.00000 | 0.00000 | 0.00000 | 0.00000 | 0.00000 | 0.00000 | 0.00000 | 0.00000 | 0.00000 | 0.00000 | 0.00000 | 0.00000 | 0.00000 | 0.33333 |
| 6-056 | 0.00000 | 0.00000 | 0.00000 | 0.00000 | 0.00000 | 0.00000 | 0.00000 | 0.00000 | 0.00000 | 0.00000 | 0.00000 | 0.00000 | 0.00000 | 0.08333 | 0.00000 | 0.00000 | 0.08333 | 0.20000 | 0.00000 | 0.33333 |
| 6-057 | 0.00000 | 0.00000 | 0.00000 | 0.08333 | 0.00000 | 0.00000 | 0.00000 | 0.00000 | 0.00000 | 0.00000 | 0.00000 | 0.00000 | 0.00000 | 0.00000 | 0.10000 | 0.00000 | 0.16667 | 0.10000 | 0.00000 | 0.33333 |
| 6-058 | 0.00000 | 0.00000 | 0.08333 | 0.00000 | 0.00000 | 0.00000 | 0.08333 | 0.00000 | 0.08333 | 0.00000 | 0.00000 | 0.00000 | 0.00000 | 0.00000 | 0.00000 | 0.00000 | 0.33333 | 0.20000 | 0.00000 | 0.16667 |
| 6-059 | 0.00000 | 0.00000 | 0.00000 | 0.00000 | 0.00000 | 0.00000 | 0.00000 | 0.00000 | 0.00000 | 0.00000 | 0.00000 | 0.00000 | 0.00000 | 0.00000 | 0.20000 | 0.00000 | 0.08333 | 0.00000 | 0.00000 | 0.50000 |
| 6-060 | 0.00000 | 0.00000 | 0.00000 | 0.00000 | 0.00000 | 0.00000 | 0.00000 | 0.00000 | 0.00000 | 0.00000 | 0.00000 | 0.08333 | 0.00000 | 0.00000 | 0.10000 | 0.00000 | 0.08333 | 0.00000 | 0.00000 | 0.50000 |
| 6-061 | 0.00000 | 0.00000 | 0.08333 | 0.00000 | 0.00000 | 0.00000 | 0.08333 | 0.00000 | 0.00000 | 0.00000 | 0.00000 | 0.00000 | 0.00000 | 0.00000 | 0.00000 | 0.00000 | 0.08333 | 0.00000 | 0.10000 | 0.33333 |
| 6-062 | 0.00000 | 0.00000 | 0.00000 | 0.00000 | 0.00000 | 0.00000 | 0.00000 | 0.00000 | 0.00000 | 0.00000 | 0.00000 | 0.08333 | 0.00000 | 0.00000 | 0.00000 | 0.00000 | 0.33333 | 0.00000 | 0.00000 | 0.16667 |
| 6-063 | 0.00000 | 0.00000 | 0.08333 | 0.00000 | 0.00000 | 0.00000 | 0.00000 | 0.00000 | 0.00000 | 0.00000 | 0.00000 | 0.00000 | 0.10000 | 0.00000 | 0.00000 | 0.00000 | 0.25000 | 0.00000 | 0.10000 | 0.33333 |
| 6-064 | 0.00000 | 0.00000 | 0.00000 | 0.00000 | 0.00000 | 0.00000 | 0.00000 | 0.00000 | 0.00000 | 0.00000 | 0.00000 | 0.00000 | 0.00000 | 0.00000 | 0.00000 | 0.00000 | 0.00000 | 0.20000 | 0.00000 | 0.16667 |
| 6-065 | 0.50000 | 0.60000 | 0.33333 | 0.58333 | 0.66667 | 0.33333 | 0.25000 | 0.66667 | 0.41667 | 0.00000 | 0.33333 | 0.41667 | 0.40000 | 0.33333 | 0.50000 | 0.50000 | 0.33333 | 0.50000 | 0.50000 | 0.00000 |
| 6-066 | 0.00000 | 0.00000 | 0.00000 | 0.00000 | 0.00000 | 0.00000 | 0.00000 | 0.00000 | 0.00000 | 0.00000 | 0.00000 | 0.00000 | 0.00000 | 0.00000 | 0.00000 | 0.00000 | 0.00000 | 0.00000 | 0.00000 | 0.16667 |
| 6-067 | 0.00000 | 0.00000 | 0.00000 | 0.08333 | 0.00000 | 0.00000 | 0.00000 | 0.00000 | 0.00000 | 0.00000 | 0.00000 | 0.00000 | 0.00000 | 0.00000 | 0.00000 | 0.00000 | 0.00000 | 0.00000 | 0.10000 | 0.00000 |
| 6-068 | 0.00000 | 0.00000 | 0.16667 | 0.00000 | 0.00000 | 0.00000 | 0.08333 | 0.00000 | 0.00000 | 0.00000 | 0.16667 | 0.00000 | 0.00000 | 0.00000 | 0.00000 | 0.00000 | 0.00000 | 0.00000 | 0.10000 | 0.33333 |
| 6-069 | 0.00000 | 0.00000 | 0.16667 | 0.16667 | 0.08333 | 0.00000 | 0.00000 | 0.00000 | 0.00000 | 0.22222 | 0.16667 | 0.00000 | 0.00000 | 0.00000 | 0.10000 | 0.00000 | 0.08333 | 0.10000 | 0.10000 | 0.16667 |
| 6-070 | 0.00000 | 0.10000 | 0.00000 | 0.08333 | 0.00000 | 0.16667 | 0.16667 | 0.16667 | 0.08333 | 0.00000 | 0.16667 | 0.25000 | 0.00000 | 0.16667 | 0.50000 | 0.10000 | 0.16667 | 0.20000 | 0.30000 | 0.16667 |
| 6-071 | 0.00000 | 0.00000 | 0.00000 | 0.00000 | 0.00000 | 0.00000 | 0.00000 | 0.00000 | 0.00000 | 0.00000 | 0.00000 | 0.00000 | 0.00000 | 0.08333 | 0.00000 | 0.00000 | 0.08333 | 0.10000 | 0.10000 | 0.00000 |
| 6-072 | 0.00000 | 0.00000 | 0.00000 | 0.00000 | 0.08333 | 0.00000 | 0.08333 | 0.00000 | 0.00000 | 0.00000 | 0.00000 | 0.00000 | 0.00000 | 0.00000 | 0.00000 | 0.00000 | 0.00000 | 0.20000 | 0.00000 | 0.00000 |
| 6-073 | 0.00000 | 0.00000 | 0.00000 | 0.00000 | 0.00000 | 0.00000 | 0.00000 | 0.00000 | 0.00000 | 0.00000 | 0.00000 | 0.00000 | 0.00000 | 0.00000 | 0.00000 | 0.00000 | 0.00000 | 0.00000 | 0.10000 | 0.50000 |
| 6-074 | 0.00000 | 0.00000 | 0.00000 | 0.00000 | 0.08333 | 0.00000 | 0.00000 | 0.00000 | 0.00000 | 0.00000 | 0.00000 | 0.00000 | 0.00000 | 0.00000 | 0.00000 | 0.00000 | 0.00000 | 0.00000 | 0.10000 | 0.50000 |
| 6-075 | 0.00000 | 0.00000 | 0.00000 | 0.00000 | 0.00000 | 0.00000 | 0.08333 | 0.00000 | 0.00000 | 0.00000 | 0.00000 | 0.08333 | 0.00000 | 0.00000 | 0.00000 | 0.00000 | 0.00000 | 0.20000 | 0.10000 | 0.00000 |
| 6-076 | 0.00000 | 0.00000 | 0.00000 | 0.00000 | 0.08333 | 0.00000 | 0.00000 | 0.00000 | 0.00000 | 0.00000 | 0.08333 | 0.00000 | 0.00000 | 0.08333 | 0.00000 | 0.10000 | 0.00000 | 0.00000 | 0.10000 | 0.00000 |
| 6-077 | 0.00000 | 0.00000 | 0.00000 | 0.00000 | 0.08333 | 0.00000 | 0.00000 | 0.00000 | 0.00000 | 0.00000 | 0.00000 | 0.00000 | 0.00000 | 0.00000 | 0.00000 | 0.00000 | 0.00000 | 0.00000 | 0.10000 | 0.50000 |
| 6-078 | 0.12500 | 0.00000 | 0.00000 | 0.00000 | 0.00000 | 0.00000 | 0.00000 | 0.00000 | 0.00000 | 0.00000 | 0.00000 | 0.00000 | 0.00000 | 0.00000 | 0.00000 | 0.00000 | 0.00000 | 0.00000 | 0.00000 | 0.33333 |
| 6-079 | 0.00000 | 0.00000 | 0.08333 | 0.00000 | 0.00000 | 0.00000 | 0.00000 | 0.00000 | 0.00000 | 0.00000 | 0.00000 | 0.00000 | 0.00000 | 0.00000 | 0.00000 | 0.00000 | 0.00000 | 0.00000 | 0.10000 | 0.16667 |
| 6-080 | 0.50000 | 0.30000 | 0.33333 | 0.08333 | 0.25000 | 0.66667 | 0.58333 | 0.33333 | 0.50000 | 0.77778 | 0.58333 | 0.41667 | 0.50000 | 0.58333 | 0.30000 | 0.80000 | 0.16667 | 0.20000 | 0.00000 | 0.00000 |
| 6-081 | 0.00000 | 0.00000 | 0.00000 | 0.00000 | 0.00000 | 0.00000 | 0.00000 | 0.00000 | 0.00000 | 0.00000 | 0.00000 | 0.00000 | 0.00000 | 0.00000 | 0.00000 | 0.20000 | 0.00000 | 0.00000 | 0.00000 | 0.16667 |
| 6-082 | 0.00000 | 0.30000 | 0.16667 | 0.33333 | 0.08333 | 0.00000 | 0.08333 | 0.00000 | 0.08333 | 0.00000 | 0.00000 | 0.25000 | 0.00000 | 0.00000 | 0.00000 | 0.00000 | 0.16667 | 0.30000 | 0.30000 | 0.00000 |
| 6-083 | 0.25000 | 0.20000 | 0.08333 | 0.16667 | 0.16667 | 0.00000 | 0.00000 | 0.00000 | 0.00000 | 0.00000 | 0.00000 | 0.08333 | 0.00000 | 0.00000 | 0.00000 | 0.00000 | 0.25000 | 0.30000 | 0.30000 | 0.00000 |
| 6-084 | 0.00000 | 0.00000 | 0.08333 | 0.00000 | 0.00000 | 0.00000 | 0.08333 | 0.00000 | 0.00000 | 0.00000 | 0.00000 | 0.00000 | 0.10000 | 0.00000 | 0.00000 | 0.00000 | 0.00000 | 0.00000 | 0.00000 | 0.00000 |
| 6-085 | 0.00000 | 0.00000 | 0.25000 | 0.33333 | 0.00000 | 0.16667 | 0.08333 | 0.00000 | 0.08333 | 0.11111 | 0.00000 | 0.00000 | 0.00000 | 0.00000 | 0.00000 | 0.00000 | 0.41667 | 0.30000 | 0.00000 | 0.33333 |
| 6-086 | 0.00000 | 0.10000 | 0.25000 | 0.33333 | 0.08333 | 0.00000 | 0.08333 | 0.00000 | 0.08333 | 0.00000 | 0.00000 | 0.16667 | 0.00000 | 0.08333 | 0.10000 | 0.00000 | 0.50000 | 0.30000 | 0.00000 | 0.33333 |
| 6-087 | 0.00000 | 0.00000 | 0.08333 | 0.08333 | 0.16667 | 0.00000 | 0.00000 | 0.16667 | 0.08333 | 0.00000 | 0.25000 | 0.00000 | 0.00000 | 0.08333 | 0.00000 | 0.00000 | 0.08333 | 0.10000 | 0.00000 | 0.00000 |
| 6-088 | 0.12500 | 0.20000 | 0.25000 | 0.08333 | 0.33333 | 0.83333 | 0.33333 | 0.33333 | 0.50000 | 0.11111 | 0.33333 | 0.33333 | 0.20000 | 0.33333 | 0.20000 | 0.20000 | 0.33333 | 0.10000 | 0.10000 | 0.16667 |
| 6-089 | 0.00000 | 0.10000 | 0.16667 | 0.00000 | 0.16667 | 0.33333 | 0.25000 | 0.33333 | 0.33333 | 0.00000 | 0.08333 | 0.25000 | 0.30000 | 0.25000 | 0.20000 | 0.10000 | 0.25000 | 0.10000 | 0.10000 | 0.00000 |
| 6-090 | 0.00000 | 0.00000 | 0.00000 | 0.00000 | 0.00000 | 0.00000 | 0.08333 | 0.16667 | 0.08333 | 0.00000 | 0.00000 | 0.16667 | 0.00000 | 0.00000 | 0.00000 | 0.00000 | 0.00000 | 0.00000 | 0.00000 | 0.00000 |
| 6-091 | 0.00000 | 0.00000 | 0.08333 | 0.00000 | 0.00000 | 0.00000 | 0.00000 | 0.16667 | 0.00000 | 0.11111 | 0.00000 | 0.00000 | 0.00000 | 0.00000 | 0.00000 | 0.00000 | 0.00000 | 0.00000 | 0.00000 | 0.00000 |
| 6-092 | 0.00000 | 0.00000 | 0.00000 | 0.00000 | 0.00000 | 0.00000 | 0.00000 | 0.00000 | 0.00000 | 0.11111 | 0.00000 | 0.00000 | 0.00000 | 0.00000 | 0.00000 | 0.00000 | 0.00000 | 0.00000 | 0.10000 | 0.00000 |
| 6-093 | 0.00000 | 0.00000 | 0.00000 | 0.00000 | 0.00000 | 0.00000 | 0.00000 | 0.00000 | 0.00000 | 0.00000 | 0.00000 | 0.00000 | 0.00000 | 0.00000 | 0.00000 | 0.00000 | 0.00000 | 0.10000 | 0.00000 | 0.00000 |
| 6-094 | 0.00000 | 0.00000 | 0.00000 | 0.00000 | 0.00000 | 0.00000 | 0.00000 | 0.00000 | 0.00000 | 0.00000 | 0.00000 | 0.00000 | 0.00000 | 0.00000 | 0.00000 | 0.00000 | 0.00000 | 0.10000 | 0.00000 | 0.00000 |
| 6-095 | 0.00000 | 0.00000 | 0.00000 | 0.00000 | 0.00000 | 0.00000 | 0.00000 | 0.00000 | 0.00000 | 0.00000 | 0.00000 | 0.00000 | 0.00000 | 0.00000 | 0.00000 | 0.00000 | 0.16667 | 0.00000 | 0.00000 | 0.16667 |
| 6-096 | 0.00000 | 0.00000 | 0.08333 | 0.33333 | 0.00000 | 0.16667 | 0.16667 | 0.16667 | 0.00000 | 0.00000 | 0.00000 | 0.00000 | 0.00000 | 0.00000 | 0.10000 | 0.00000 | 0.41667 | 0.20000 | 0.00000 | 0.33333 |
| 6-097 | 0.25000 | 0.10000 | 0.08333 | 0.25000 | 0.16667 | 0.00000 | 0.08333 | 0.00000 | 0.00000 | 0.00000 | 0.00000 | 0.08333 | 0.00000 | 0.00000 | 0.00000 | 0.00000 | 0.00000 | 0.10000 | 0.00000 | 0.00000 |
| 6-098 | 0.00000 | 0.00000 | 0.00000 | 0.00000 | 0.00000 | 0.00000 | 0.08333 | 0.00000 | 0.08333 | 0.00000 | 0.00000 | 0.00000 | 0.00000 | 0.00000 | 0.00000 | 0.00000 | 0.00000 | 0.10000 | 0.00000 | 0.33333 |
| 6-099 | 0.37500 | 0.10000 | 0.08333 | 0.25000 | 0.16667 | 0.00000 | 0.00000 | 0.00000 | 0.08333 | 0.00000 | 0.00000 | 0.00000 | 0.00000 | 0.00000 | 0.00000 | 0.00000 | 0.00000 | 0.00000 | 0.00000 | 0.16667 |
| 6-100 | 0.00000 | 0.00000 | 0.00000 | 0.00000 | 0.00000 | 0.00000 | 0.00000 | 0.00000 | 0.00000 | 0.00000 | 0.00000 | 0.00000 | 0.00000 | 0.00000 | 0.00000 | 0.10000 | 0.08333 | 0.00000 | 0.00000 | 0.00000 |
| 6-101 | 0.00000 | 0.00000 | 0.00000 | 0.00000 | 0.00000 | 0.00000 | 0.00000 | 0.00000 | 0.00000 | 0.00000 | 0.00000 | 0.00000 | 0.00000 | 0.00000 | 0.00000 | 0.10000 | 0.00000 | 0.00000 | 0.00000 | 0.33333 |
| 6-102 | 0.00000 | 0.10000 | 0.08333 | 0.00000 | 0.08333 | 0.00000 | 0.00000 | 0.16667 | 0.08333 | 0.00000 | 0.08333 | 0.00000 | 0.00000 | 0.00000 | 0.00000 | 0.00000 | 0.08333 | 0.10000 | 0.10000 | 0.50000 |
| 6-103 | 0.00000 | 0.10000 | 0.08333 | 0.00000 | 0.08333 | 0.00000 | 0.00000 | 0.16667 | 0.08333 | 0.00000 | 0.16667 | 0.00000 | 0.00000 | 0.00000 | 0.00000 | 0.00000 | 0.08333 | 0.10000 | 0.00000 | 0.16667 |
| 6-104 | 0.00000 | 0.00000 | 0.00000 | 0.00000 | 0.00000 | 0.00000 | 0.00000 | 0.00000 | 0.00000 | 0.00000 | 0.00000 | 0.00000 | 0.00000 | 0.00000 | 0.00000 | 0.00000 | 0.16667 | 0.00000 | 0.00000 | 0.00000 |
| 6-105 | 0.00000 | 0.00000 | 0.00000 | 0.00000 | 0.00000 | 0.00000 | 0.00000 | 0.00000 | 0.16667 | 0.00000 | 0.00000 | 0.00000 | 0.00000 | 0.00000 | 0.00000 | 0.00000 | 0.25000 | 0.10000 | 0.00000 | 0.16667 |
| 6-106 | 0.00000 | 0.00000 | 0.08333 | 0.00000 | 0.08333 | 0.16667 | 0.00000 | 0.00000 | 0.25000 | 0.00000 | 0.00000 | 0.00000 | 0.00000 | 0.00000 | 0.00000 | 0.00000 | 0.00000 | 0.20000 | 0.00000 | 0.00000 |
| 6-107 | 0.12500 | 0.00000 | 0.16667 | 0.00000 | 0.08333 | 0.66667 | 0.41667 | 0.00000 | 0.50000 | 0.00000 | 0.16667 | 0.16667 | 0.20000 | 0.16667 | 0.00000 | 0.20000 | 0.00000 | 0.10000 | 0.00000 | 0.00000 |
| 6-108 | 0.00000 | 0.00000 | 0.08333 | 0.00000 | 0.08333 | 0.50000 | 0.41667 | 0.00000 | 0.41667 | 0.00000 | 0.16667 | 0.16667 | 0.00000 | 0.16667 | 0.00000 | 0.30000 | 0.00000 | 0.10000 | 0.00000 | 0.16667 |
| 6-109 | 0.00000 | 0.00000 | 0.00000 | 0.08333 | 0.00000 | 0.00000 | 0.00000 | 0.16667 | 0.00000 | 0.00000 | 0.00000 | 0.00000 | 0.00000 | 0.00000 | 0.00000 | 0.10000 | 0.00000 | 0.00000 | 0.00000 | 0.00000 |
| 6-110 | 0.00000 | 0.00000 | 0.00000 | 0.08333 | 0.00000 | 0.00000 | 0.00000 | 0.00000 | 0.00000 | 0.00000 | 0.00000 | 0.00000 | 0.00000 | 0.00000 | 0.00000 | 0.00000 | 0.00000 | 0.10000 | 0.00000 | 0.16667 |
| 6-111 | 0.00000 | 0.00000 | 0.00000 | 0.00000 | 0.00000 | 0.00000 | 0.00000 | 0.00000 | 0.00000 | 0.00000 | 0.00000 | 0.00000 | 0.00000 | 0.00000 | 0.00000 | 0.00000 | 0.08333 | 0.20000 | 0.00000 | 0.16667 |
| 6-112 | 0.00000 | 0.00000 | 0.00000 | 0.08333 | 0.00000 | 0.00000 | 0.00000 | 0.00000 | 0.00000 | 0.00000 | 0.00000 | 0.00000 | 0.00000 | 0.00000 | 0.10000 | 0.00000 | 0.25000 | 0.10000 | 0.00000 | 0.16667 |
| 6-113 | 0.00000 | 0.00000 | 0.00000 | 0.16667 | 0.00000 | 0.00000 | 0.08333 | 0.00000 | 0.08333 | 0.00000 | 0.00000 | 0.00000 | 0.00000 | 0.00000 | 0.10000 | 0.00000 | 0.16667 | 0.10000 | 0.00000 | 0.16667 |
| 6-114 | 0.00000 | 0.10000 | 0.00000 | 0.25000 | 0.00000 | 0.00000 | 0.00000 | 0.00000 | 0.00000 | 0.00000 | 0.00000 | 0.00000 | 0.00000 | 0.00000 | 0.00000 | 0.00000 | 0.41667 | 0.10000 | 0.00000 | 0.16667 |
| 6-115 | 0.00000 | 0.00000 | 0.00000 | 0.00000 | 0.00000 | 0.00000 | 0.08333 | 0.00000 | 0.00000 | 0.00000 | 0.00000 | 0.00000 | 0.00000 | 0.00000 | 0.00000 | 0.00000 | 0.41667 | 0.00000 | 0.00000 | 0.16667 |
| 6-116 | 0.12500 | 0.00000 | 0.00000 | 0.00000 | 0.00000 | 0.00000 | 0.00000 | 0.00000 | 0.00000 | 0.00000 | 0.00000 | 0.00000 | 0.00000 | 0.00000 | 0.00000 | 0.00000 | 0.00000 | 0.00000 | 0.00000 | 0.00000 |
| 6-117 | 0.00000 | 0.00000 | 0.00000 | 0.00000 | 0.00000 | 0.00000 | 0.00000 | 0.00000 | 0.00000 | 0.00000 | 0.00000 | 0.00000 | 0.00000 | 0.00000 | 0.00000 | 0.00000 | 0.00000 | 0.00000 | 0.00000 | 0.16667 |
| 6-118 | 0.00000 | 0.00000 | 0.00000 | 0.00000 | 0.00000 | 0.00000 | 0.00000 | 0.00000 | 0.00000 | 0.00000 | 0.00000 | 0.00000 | 0.00000 | 0.00000 | 0.00000 | 0.00000 | 0.08333 | 0.00000 | 0.00000 | 0.00000 |
| 6-119 | 0.00000 | 0.00000 | 0.00000 | 0.08333 | 0.00000 | 0.00000 | 0.00000 | 0.00000 | 0.00000 | 0.00000 | 0.00000 | 0.00000 | 0.00000 | 0.00000 | 0.00000 | 0.00000 | 0.00000 | 0.00000 | 0.00000 | 0.00000 |
| 6-120 | 0.00000 | 0.00000 | 0.00000 | 0.00000 | 0.00000 | 0.00000 | 0.00000 | 0.00000 | 0.00000 | 0.00000 | 0.08333 | 0.00000 | 0.00000 | 0.00000 | 0.00000 | 0.00000 | 0.00000 | 0.00000 | 0.00000 | 0.00000 |
| 6-121 | 0.00000 | 0.00000 | 0.00000 | 0.08333 | 0.00000 | 0.00000 | 0.00000 | 0.00000 | 0.00000 | 0.00000 | 0.00000 | 0.00000 | 0.00000 | 0.00000 | 0.00000 | 0.00000 | 0.00000 | 0.00000 | 0.00000 | 0.00000 |
| 6-122 | 0.00000 | 0.00000 | 0.00000 | 0.00000 | 0.00000 | 0.00000 | 0.00000 | 0.00000 | 0.00000 | 0.00000 | 0.00000 | 0.00000 | 0.00000 | 0.00000 | 0.10000 | 0.00000 | 0.00000 | 0.00000 | 0.00000 | 0.00000 |
| 6-123 | 0.00000 | 0.00000 | 0.00000 | 0.00000 | 0.00000 | 0.00000 | 0.00000 | 0.00000 | 0.00000 | 0.00000 | 0.00000 | 0.00000 | 0.00000 | 0.00000 | 0.00000 | 0.00000 | 0.00000 | 0.00000 | 0.00000 | 0.16667 |
| 6-124 | 0.00000 | 0.00000 | 0.00000 | 0.00000 | 0.00000 | 0.00000 | 0.00000 | 0.00000 | 0.00000 | 0.00000 | 0.00000 | 0.00000 | 0.00000 | 0.00000 | 0.00000 | 0.00000 | 0.00000 | 0.00000 | 0.00000 | 0.16667 |
| 6-125 | 0.00000 | 0.00000 | 0.00000 | 0.00000 | 0.00000 | 0.00000 | 0.00000 | 0.00000 | 0.00000 | 0.00000 | 0.00000 | 0.00000 | 0.00000 | 0.00000 | 0.00000 | 0.00000 | 0.00000 | 0.00000 | 0.00000 | 0.16667 |
| 9-001 | 0.00000 | 0.00000 | 0.00000 | 0.00000 | 0.00000 | 0.00000 | 0.00000 | 0.00000 | 0.00000 | 0.00000 | 0.08333 | 0.00000 | 0.00000 | 0.00000 | 0.00000 | 0.00000 | 0.08333 | 0.00000 | 0.00000 | 0.16667 |
| 9-002 | 0.00000 | 0.00000 | 0.00000 | 0.00000 | 0.00000 | 0.00000 | 0.00000 | 0.00000 | 0.00000 | 0.00000 | 0.00000 | 0.00000 | 0.00000 | 0.00000 | 0.00000 | 0.00000 | 0.08333 | 0.00000 | 0.00000 | 0.16667 |
| 9-003 | 0.00000 | 0.00000 | 0.00000 | 0.00000 | 0.25000 | 0.33333 | 0.00000 | 0.00000 | 0.25000 | 0.00000 | 0.16667 | 0.08333 | 0.10000 | 0.16667 | 0.10000 | 0.10000 | 0.08333 | 0.00000 | 0.12500 | 0.16667 |
| 9-004 | 0.00000 | 0.10000 | 0.00000 | 0.00000 | 0.25000 | 0.33333 | 0.00000 | 0.00000 | 0.33333 | 0.00000 | 0.25000 | 0.16667 | 0.00000 | 0.08333 | 0.10000 | 0.10000 | 0.08333 | 0.00000 | 0.12500 | 0.16667 |
| 9-005 | 0.00000 | 0.00000 | 0.00000 | 0.00000 | 0.00000 | 0.00000 | 0.00000 | 0.00000 | 0.00000 | 0.00000 | 0.00000 | 0.00000 | 0.00000 | 0.00000 | 0.10000 | 0.00000 | 0.00000 | 0.00000 | 0.00000 | 0.00000 |
| 9-006 | 0.00000 | 0.00000 | 0.00000 | 0.00000 | 0.00000 | 0.00000 | 0.00000 | 0.00000 | 0.00000 | 0.00000 | 0.00000 | 0.00000 | 0.00000 | 0.00000 | 0.00000 | 0.00000 | 0.08333 | 0.00000 | 0.00000 | 0.00000 |
| 9-007 | 0.00000 | 0.00000 | 0.08333 | 0.08333 | 0.00000 | 0.00000 | 0.00000 | 0.00000 | 0.00000 | 0.00000 | 0.00000 | 0.00000 | 0.10000 | 0.00000 | 0.00000 | 0.00000 | 0.00000 | 0.10000 | 0.12500 | 0.16667 |
| 9-008 | 0.00000 | 0.00000 | 0.08333 | 0.08333 | 0.00000 | 0.00000 | 0.00000 | 0.00000 | 0.00000 | 0.00000 | 0.00000 | 0.00000 | 0.10000 | 0.08333 | 0.00000 | 0.00000 | 0.00000 | 0.10000 | 0.12500 | 0.00000 |
| 9-009 | 0.00000 | 0.00000 | 0.00000 | 0.00000 | 0.00000 | 0.00000 | 0.00000 | 0.00000 | 0.00000 | 0.00000 | 0.00000 | 0.00000 | 0.00000 | 0.08333 | 0.00000 | 0.00000 | 0.00000 | 0.00000 | 0.00000 | 0.00000 |
| 9-010 | 0.00000 | 0.20000 | 0.00000 | 0.00000 | 0.00000 | 0.33333 | 0.08333 | 0.00000 | 0.16667 | 0.00000 | 0.08333 | 0.00000 | 0.30000 | 0.25000 | 0.10000 | 0.20000 | 0.00000 | 0.00000 | 0.12500 | 0.16667 |
| 9-011 | 0.00000 | 0.10000 | 0.00000 | 0.00000 | 0.16667 | 0.50000 | 0.08333 | 0.00000 | 0.08333 | 0.00000 | 0.16667 | 0.00000 | 0.30000 | 0.25000 | 0.10000 | 0.30000 | 0.08333 | 0.00000 | 0.12500 | 0.16667 |
| 9-012 | 0.12500 | 0.20000 | 0.00000 | 0.08333 | 0.33333 | 0.00000 | 0.00000 | 0.00000 | 0.00000 | 0.00000 | 0.00000 | 0.00000 | 0.00000 | 0.00000 | 0.00000 | 0.00000 | 0.00000 | 0.00000 | 0.00000 | 0.00000 |
| 9-013 | 0.00000 | 0.00000 | 0.00000 | 0.00000 | 0.00000 | 0.00000 | 0.00000 | 0.00000 | 0.00000 | 0.00000 | 0.08333 | 0.00000 | 0.00000 | 0.00000 | 0.00000 | 0.00000 | 0.00000 | 0.00000 | 0.00000 | 0.00000 |
| 9-014 | 0.00000 | 0.00000 | 0.00000 | 0.00000 | 0.00000 | 0.00000 | 0.00000 | 0.00000 | 0.00000 | 0.00000 | 0.16667 | 0.00000 | 0.00000 | 0.08333 | 0.00000 | 0.00000 | 0.00000 | 0.00000 | 0.12500 | 0.00000 |
| 9-015 | 0.00000 | 0.00000 | 0.00000 | 0.00000 | 0.00000 | 0.00000 | 0.00000 | 0.00000 | 0.00000 | 0.00000 | 0.08333 | 0.00000 | 0.10000 | 0.00000 | 0.00000 | 0.00000 | 0.00000 | 0.00000 | 0.12500 | 0.00000 |
| 9-016 | 0.00000 | 0.10000 | 0.00000 | 0.00000 | 0.00000 | 0.00000 | 0.00000 | 0.00000 | 0.00000 | 0.00000 | 0.00000 | 0.00000 | 0.00000 | 0.00000 | 0.00000 | 0.00000 | 0.00000 | 0.00000 | 0.00000 | 0.00000 |
| 9-017 | 0.00000 | 0.10000 | 0.00000 | 0.00000 | 0.08333 | 0.00000 | 0.08333 | 0.00000 | 0.08333 | 0.00000 | 0.08333 | 0.16667 | 0.00000 | 0.00000 | 0.00000 | 0.00000 | 0.16667 | 0.00000 | 0.00000 | 0.16667 |
| 9-018 | 0.00000 | 0.20000 | 0.00000 | 0.00000 | 0.08333 | 0.33333 | 0.16667 | 0.00000 | 0.16667 | 0.00000 | 0.00000 | 0.16667 | 0.00000 | 0.08333 | 0.00000 | 0.00000 | 0.16667 | 0.00000 | 0.00000 | 0.16667 |
| 9-019 | 0.00000 | 0.00000 | 0.00000 | 0.00000 | 0.00000 | 0.00000 | 0.00000 | 0.00000 | 0.00000 | 0.00000 | 0.08333 | 0.00000 | 0.00000 | 0.00000 | 0.00000 | 0.00000 | 0.00000 | 0.00000 | 0.00000 | 0.16667 |
| 9-020 | 0.00000 | 0.00000 | 0.00000 | 0.00000 | 0.00000 | 0.00000 | 0.00000 | 0.00000 | 0.00000 | 0.00000 | 0.00000 | 0.00000 | 0.00000 | 0.00000 | 0.00000 | 0.00000 | 0.00000 | 0.00000 | 0.00000 | 0.16667 |
| 9-021 | 0.00000 | 0.00000 | 0.00000 | 0.00000 | 0.00000 | 0.16667 | 0.00000 | 0.00000 | 0.00000 | 0.00000 | 0.00000 | 0.00000 | 0.00000 | 0.00000 | 0.00000 | 0.00000 | 0.00000 | 0.00000 | 0.00000 | 0.00000 |
| 9-022 | 0.12500 | 0.00000 | 0.00000 | 0.00000 | 0.00000 | 0.00000 | 0.00000 | 0.00000 | 0.00000 | 0.00000 | 0.00000 | 0.00000 | 0.00000 | 0.00000 | 0.00000 | 0.00000 | 0.00000 | 0.00000 | 0.00000 | 0.00000 |
| 9-023 | 0.00000 | 0.10000 | 0.00000 | 0.00000 | 0.08333 | 0.00000 | 0.00000 | 0.00000 | 0.00000 | 0.00000 | 0.00000 | 0.00000 | 0.00000 | 0.00000 | 0.10000 | 0.00000 | 0.00000 | 0.00000 | 0.00000 | 0.00000 |
| 9-024 | 0.00000 | 0.10000 | 0.00000 | 0.00000 | 0.08333 | 0.00000 | 0.00000 | 0.00000 | 0.00000 | 0.00000 | 0.00000 | 0.00000 | 0.00000 | 0.00000 | 0.10000 | 0.00000 | 0.00000 | 0.00000 | 0.00000 | 0.00000 |
| 9-025 | 0.00000 | 0.00000 | 0.00000 | 0.00000 | 0.33333 | 0.00000 | 0.00000 | 0.00000 | 0.00000 | 0.00000 | 0.08333 | 0.00000 | 0.00000 | 0.00000 | 0.00000 | 0.10000 | 0.00000 | 0.00000 | 0.00000 | 0.00000 |
| 9-026 | 0.00000 | 0.00000 | 0.00000 | 0.00000 | 0.00000 | 0.00000 | 0.08333 | 0.00000 | 0.08333 | 0.00000 | 0.00000 | 0.08333 | 0.00000 | 0.08333 | 0.00000 | 0.00000 | 0.08333 | 0.00000 | 0.00000 | 0.16667 |
| 9-027 | 0.00000 | 0.00000 | 0.00000 | 0.00000 | 0.00000 | 0.00000 | 0.00000 | 0.00000 | 0.00000 | 0.00000 | 0.08333 | 0.08333 | 0.00000 | 0.16667 | 0.00000 | 0.20000 | 0.00000 | 0.00000 | 0.00000 | 0.00000 |
| 9-028 | 0.00000 | 0.00000 | 0.00000 | 0.00000 | 0.00000 | 0.00000 | 0.00000 | 0.00000 | 0.00000 | 0.00000 | 0.08333 | 0.08333 | 0.00000 | 0.00000 | 0.00000 | 0.00000 | 0.00000 | 0.00000 | 0.00000 | 0.16667 |
| 9-029 | 0.00000 | 0.00000 | 0.00000 | 0.00000 | 0.00000 | 0.00000 | 0.08333 | 0.00000 | 0.00000 | 0.00000 | 0.00000 | 0.00000 | 0.00000 | 0.00000 | 0.00000 | 0.00000 | 0.00000 | 0.00000 | 0.00000 | 0.00000 |
| 9-030 | 0.00000 | 0.00000 | 0.00000 | 0.08333 | 0.00000 | 0.00000 | 0.00000 | 0.00000 | 0.00000 | 0.00000 | 0.00000 | 0.00000 | 0.00000 | 0.00000 | 0.00000 | 0.10000 | 0.00000 | 0.00000 | 0.12500 | 0.00000 |
| 9-031 | 0.00000 | 0.00000 | 0.08333 | 0.08333 | 0.00000 | 0.00000 | 0.00000 | 0.00000 | 0.00000 | 0.00000 | 0.00000 | 0.00000 | 0.00000 | 0.08333 | 0.00000 | 0.20000 | 0.00000 | 0.00000 | 0.00000 | 0.00000 |
| 9-032 | 0.12500 | 0.20000 | 0.08333 | 0.00000 | 0.08333 | 0.16667 | 0.00000 | 0.00000 | 0.08333 | 0.00000 | 0.00000 | 0.00000 | 0.00000 | 0.08333 | 0.00000 | 0.10000 | 0.00000 | 0.00000 | 0.00000 | 0.00000 |
| 9-033 | 0.00000 | 0.00000 | 0.00000 | 0.00000 | 0.00000 | 0.00000 | 0.00000 | 0.00000 | 0.00000 | 0.00000 | 0.08333 | 0.00000 | 0.00000 | 0.00000 | 0.00000 | 0.00000 | 0.00000 | 0.00000 | 0.00000 | 0.00000 |
| 9-034 | 0.00000 | 0.00000 | 0.00000 | 0.00000 | 0.00000 | 0.00000 | 0.00000 | 0.00000 | 0.00000 | 0.00000 | 0.00000 | 0.00000 | 0.00000 | 0.00000 | 0.00000 | 0.00000 | 0.08333 | 0.00000 | 0.00000 | 0.00000 |
| 9-035 | 0.00000 | 0.10000 | 0.00000 | 0.00000 | 0.00000 | 0.16667 | 0.08333 | 0.00000 | 0.00000 | 0.00000 | 0.00000 | 0.00000 | 0.00000 | 0.00000 | 0.00000 | 0.00000 | 0.00000 | 0.10000 | 0.00000 | 0.00000 |
| 9-036 | 0.00000 | 0.00000 | 0.00000 | 0.00000 | 0.00000 | 0.16667 | 0.08333 | 0.00000 | 0.00000 | 0.00000 | 0.08333 | 0.16667 | 0.00000 | 0.00000 | 0.00000 | 0.00000 | 0.00000 | 0.00000 | 0.00000 | 0.00000 |
| 9-037 | 0.00000 | 0.00000 | 0.00000 | 0.00000 | 0.00000 | 0.00000 | 0.00000 | 0.00000 | 0.00000 | 0.00000 | 0.00000 | 0.00000 | 0.00000 | 0.00000 | 0.00000 | 0.00000 | 0.00000 | 0.00000 | 0.12500 | 0.00000 |
| 9-038 | 0.50000 | 0.50000 | 0.08333 | 0.41667 | 0.66667 | 0.00000 | 0.08333 | 0.00000 | 0.33333 | 0.00000 | 0.25000 | 0.08333 | 0.10000 | 0.08333 | 0.30000 | 0.10000 | 0.16667 | 0.00000 | 0.00000 | 0.00000 |
| 9-039 | 0.50000 | 0.50000 | 0.08333 | 0.41667 | 0.66667 | 0.00000 | 0.08333 | 0.00000 | 0.33333 | 0.00000 | 0.16667 | 0.08333 | 0.20000 | 0.08333 | 0.30000 | 0.20000 | 0.25000 | 0.10000 | 0.12500 | 0.16667 |
| 9-040 | 0.00000 | 0.00000 | 0.00000 | 0.00000 | 0.00000 | 0.00000 | 0.08333 | 0.00000 | 0.00000 | 0.00000 | 0.00000 | 0.00000 | 0.00000 | 0.00000 | 0.00000 | 0.00000 | 0.16667 | 0.00000 | 0.00000 | 0.16667 |
| 9-041 | 0.00000 | 0.20000 | 0.00000 | 0.00000 | 0.00000 | 0.00000 | 0.00000 | 0.00000 | 0.00000 | 0.00000 | 0.00000 | 0.00000 | 0.00000 | 0.00000 | 0.00000 | 0.00000 | 0.00000 | 0.00000 | 0.00000 | 0.00000 |
| 9-042 | 0.00000 | 0.10000 | 0.00000 | 0.00000 | 0.16667 | 0.00000 | 0.00000 | 0.00000 | 0.00000 | 0.00000 | 0.00000 | 0.00000 | 0.00000 | 0.00000 | 0.00000 | 0.00000 | 0.00000 | 0.00000 | 0.00000 | 0.16667 |
| 9-043 | 0.00000 | 0.10000 | 0.00000 | 0.08333 | 0.16667 | 0.00000 | 0.00000 | 0.00000 | 0.00000 | 0.00000 | 0.00000 | 0.00000 | 0.00000 | 0.00000 | 0.00000 | 0.00000 | 0.00000 | 0.00000 | 0.00000 | 0.00000 |
| 9-044 | 0.00000 | 0.10000 | 0.00000 | 0.00000 | 0.16667 | 0.00000 | 0.00000 | 0.00000 | 0.00000 | 0.00000 | 0.08333 | 0.00000 | 0.00000 | 0.08333 | 0.10000 | 0.10000 | 0.08333 | 0.00000 | 0.00000 | 0.16667 |
| 9-045 | 0.00000 | 0.00000 | 0.08333 | 0.50000 | 0.33333 | 0.00000 | 0.00000 | 0.00000 | 0.00000 | 0.00000 | 0.00000 | 0.00000 | 0.10000 | 0.08333 | 0.00000 | 0.00000 | 0.00000 | 0.00000 | 0.00000 | 0.00000 |
| 9-046 | 0.50000 | 0.20000 | 0.41667 | 0.16667 | 0.08333 | 0.33333 | 0.08333 | 0.00000 | 0.08333 | 0.00000 | 0.08333 | 0.00000 | 0.30000 | 0.25000 | 0.10000 | 0.30000 | 0.00000 | 0.10000 | 0.12500 | 0.16667 |
| 9-047 | 0.50000 | 0.30000 | 0.41667 | 0.16667 | 0.16667 | 0.33333 | 0.08333 | 0.00000 | 0.08333 | 0.00000 | 0.08333 | 0.00000 | 0.20000 | 0.08333 | 0.10000 | 0.30000 | 0.00000 | 0.00000 | 0.00000 | 0.16667 |
| 9-048 | 0.00000 | 0.00000 | 0.00000 | 0.00000 | 0.16667 | 0.00000 | 0.00000 | 0.00000 | 0.00000 | 0.00000 | 0.00000 | 0.00000 | 0.00000 | 0.00000 | 0.00000 | 0.00000 | 0.00000 | 0.00000 | 0.00000 | 0.00000 |
| 9-049 | 0.00000 | 0.00000 | 0.00000 | 0.00000 | 0.00000 | 0.33333 | 0.00000 | 0.00000 | 0.00000 | 0.00000 | 0.08333 | 0.00000 | 0.00000 | 0.00000 | 0.00000 | 0.00000 | 0.00000 | 0.00000 | 0.12500 | 0.00000 |
| 9-050 | 0.00000 | 0.00000 | 0.08333 | 0.00000 | 0.00000 | 0.16667 | 0.00000 | 0.00000 | 0.00000 | 0.00000 | 0.00000 | 0.00000 | 0.00000 | 0.00000 | 0.00000 | 0.00000 | 0.00000 | 0.00000 | 0.00000 | 0.00000 |
| 9-051 | 0.00000 | 0.00000 | 0.00000 | 0.00000 | 0.00000 | 0.00000 | 0.00000 | 0.00000 | 0.00000 | 0.00000 | 0.00000 | 0.00000 | 0.00000 | 0.00000 | 0.00000 | 0.00000 | 0.00000 | 0.10000 | 0.12500 | 0.00000 |
| 9-052 | 0.00000 | 0.20000 | 0.08333 | 0.33333 | 0.00000 | 0.00000 | 0.16667 | 0.00000 | 0.08333 | 0.00000 | 0.08333 | 0.16667 | 0.10000 | 0.00000 | 0.20000 | 0.00000 | 0.16667 | 0.00000 | 0.00000 | 0.33333 |
| 9-053 | 0.00000 | 0.10000 | 0.08333 | 0.33333 | 0.00000 | 0.00000 | 0.08333 | 0.00000 | 0.08333 | 0.00000 | 0.08333 | 0.16667 | 0.00000 | 0.00000 | 0.20000 | 0.00000 | 0.16667 | 0.20000 | 0.12500 | 0.33333 |
| 9-054 | 0.00000 | 0.20000 | 0.00000 | 0.00000 | 0.00000 | 0.00000 | 0.00000 | 0.00000 | 0.00000 | 0.00000 | 0.00000 | 0.00000 | 0.10000 | 0.00000 | 0.00000 | 0.00000 | 0.00000 | 0.00000 | 0.00000 | 0.00000 |
| 9-055 | 0.00000 | 0.10000 | 0.16667 | 0.00000 | 0.00000 | 0.00000 | 0.00000 | 0.00000 | 0.00000 | 0.00000 | 0.00000 | 0.00000 | 0.00000 | 0.00000 | 0.00000 | 0.00000 | 0.00000 | 0.00000 | 0.00000 | 0.16667 |
| 9-056 | 0.00000 | 0.00000 | 0.00000 | 0.00000 | 0.08333 | 0.00000 | 0.08333 | 0.00000 | 0.00000 | 0.00000 | 0.00000 | 0.00000 | 0.00000 | 0.00000 | 0.10000 | 0.00000 | 0.00000 | 0.00000 | 0.00000 | 0.16667 |
| 9-057 | 0.00000 | 0.00000 | 0.16667 | 0.08333 | 0.25000 | 0.00000 | 0.00000 | 0.00000 | 0.00000 | 0.00000 | 0.00000 | 0.00000 | 0.00000 | 0.00000 | 0.00000 | 0.10000 | 0.08333 | 0.00000 | 0.12500 | 0.00000 |
| 9-058 | 0.00000 | 0.00000 | 0.00000 | 0.08333 | 0.16667 | 0.16667 | 0.00000 | 0.00000 | 0.00000 | 0.00000 | 0.00000 | 0.00000 | 0.00000 | 0.00000 | 0.00000 | 0.00000 | 0.00000 | 0.00000 | 0.00000 | 0.00000 |
| 9-059 | 0.00000 | 0.30000 | 0.00000 | 0.00000 | 0.00000 | 0.00000 | 0.00000 | 0.00000 | 0.00000 | 0.00000 | 0.00000 | 0.00000 | 0.00000 | 0.00000 | 0.00000 | 0.00000 | 0.00000 | 0.00000 | 0.12500 | 0.00000 |
| 9-060 | 0.00000 | 0.00000 | 0.00000 | 0.16667 | 0.00000 | 0.00000 | 0.08333 | 0.00000 | 0.08333 | 0.00000 | 0.00000 | 0.08333 | 0.00000 | 0.00000 | 0.10000 | 0.00000 | 0.08333 | 0.10000 | 0.00000 | 0.16667 |
| 9-061 | 0.00000 | 0.00000 | 0.00000 | 0.08333 | 0.00000 | 0.00000 | 0.08333 | 0.00000 | 0.00000 | 0.00000 | 0.08333 | 0.00000 | 0.00000 | 0.08333 | 0.10000 | 0.00000 | 0.08333 | 0.00000 | 0.12500 | 0.16667 |
| 9-062 | 0.00000 | 0.10000 | 0.00000 | 0.00000 | 0.00000 | 0.33333 | 0.00000 | 0.00000 | 0.00000 | 0.00000 | 0.08333 | 0.00000 | 0.00000 | 0.08333 | 0.00000 | 0.00000 | 0.00000 | 0.00000 | 0.00000 | 0.16667 |
| 9-063 | 0.00000 | 0.00000 | 0.00000 | 0.00000 | 0.00000 | 0.00000 | 0.00000 | 0.00000 | 0.00000 | 0.00000 | 0.00000 | 0.00000 | 0.00000 | 0.00000 | 0.10000 | 0.00000 | 0.08333 | 0.00000 | 0.00000 | 0.33333 |
| 9-064 | 0.12500 | 0.00000 | 0.00000 | 0.00000 | 0.00000 | 0.00000 | 0.08333 | 0.00000 | 0.00000 | 0.00000 | 0.00000 | 0.00000 | 0.00000 | 0.00000 | 0.00000 | 0.10000 | 0.08333 | 0.00000 | 0.00000 | 0.16667 |
| 9-065 | 0.12500 | 0.00000 | 0.00000 | 0.00000 | 0.00000 | 0.00000 | 0.08333 | 0.00000 | 0.00000 | 0.00000 | 0.00000 | 0.00000 | 0.00000 | 0.00000 | 0.00000 | 0.00000 | 0.00000 | 0.00000 | 0.00000 | 0.00000 |
| 9-066 | 0.12500 | 0.00000 | 0.00000 | 0.00000 | 0.00000 | 0.00000 | 0.08333 | 0.00000 | 0.00000 | 0.00000 | 0.16667 | 0.00000 | 0.00000 | 0.00000 | 0.00000 | 0.00000 | 0.00000 | 0.00000 | 0.00000 | 0.00000 |
| 9-067 | 0.12500 | 0.00000 | 0.00000 | 0.00000 | 0.00000 | 0.00000 | 0.08333 | 0.00000 | 0.00000 | 0.00000 | 0.08333 | 0.00000 | 0.00000 | 0.00000 | 0.00000 | 0.10000 | 0.00000 | 0.00000 | 0.00000 | 0.00000 |
| 9-068 | 0.00000 | 0.00000 | 0.00000 | 0.00000 | 0.08333 | 0.00000 | 0.08333 | 0.00000 | 0.00000 | 0.00000 | 0.08333 | 0.00000 | 0.00000 | 0.00000 | 0.00000 | 0.00000 | 0.00000 | 0.00000 | 0.00000 | 0.00000 |
| 9-069 | 0.37500 | 0.10000 | 0.00000 | 0.16667 | 0.33333 | 0.00000 | 0.00000 | 0.00000 | 0.00000 | 0.00000 | 0.08333 | 0.00000 | 0.00000 | 0.08333 | 0.00000 | 0.10000 | 0.16667 | 0.00000 | 0.00000 | 0.16667 |
| 9-070 | 0.37500 | 0.10000 | 0.00000 | 0.08333 | 0.50000 | 0.00000 | 0.00000 | 0.00000 | 0.00000 | 0.00000 | 0.00000 | 0.00000 | 0.00000 | 0.00000 | 0.00000 | 0.00000 | 0.16667 | 0.00000 | 0.00000 | 0.50000 |
| 9-071 | 0.00000 | 0.00000 | 0.00000 | 0.00000 | 0.00000 | 0.00000 | 0.00000 | 0.00000 | 0.00000 | 0.00000 | 0.08333 | 0.00000 | 0.00000 | 0.08333 | 0.00000 | 0.00000 | 0.00000 | 0.00000 | 0.12500 | 0.33333 |
| 9-072 | 0.00000 | 0.00000 | 0.00000 | 0.00000 | 0.00000 | 0.00000 | 0.00000 | 0.00000 | 0.00000 | 0.00000 | 0.00000 | 0.00000 | 0.00000 | 0.08333 | 0.00000 | 0.00000 | 0.00000 | 0.00000 | 0.12500 | 0.00000 |
| 9-073 | 0.00000 | 0.10000 | 0.00000 | 0.00000 | 0.08333 | 0.16667 | 0.00000 | 0.00000 | 0.08333 | 0.00000 | 0.00000 | 0.00000 | 0.00000 | 0.00000 | 0.00000 | 0.00000 | 0.00000 | 0.00000 | 0.00000 | 0.00000 |
| 9-074 | 0.00000 | 0.10000 | 0.00000 | 0.00000 | 0.08333 | 0.16667 | 0.08333 | 0.00000 | 0.00000 | 0.00000 | 0.00000 | 0.00000 | 0.00000 | 0.00000 | 0.00000 | 0.00000 | 0.00000 | 0.00000 | 0.00000 | 0.00000 |
| 9-075 | 0.12500 | 0.00000 | 0.00000 | 0.00000 | 0.00000 | 0.00000 | 0.08333 | 0.00000 | 0.00000 | 0.00000 | 0.00000 | 0.00000 | 0.00000 | 0.08333 | 0.00000 | 0.00000 | 0.00000 | 0.00000 | 0.00000 | 0.00000 |
| 9-076 | 0.00000 | 0.00000 | 0.00000 | 0.00000 | 0.00000 | 0.00000 | 0.00000 | 0.00000 | 0.00000 | 0.25000 | 0.00000 | 0.00000 | 0.00000 | 0.00000 | 0.00000 | 0.00000 | 0.00000 | 0.00000 | 0.00000 | 0.00000 |
| 9-077 | 0.50000 | 0.30000 | 0.33333 | 0.41667 | 0.66667 | 0.00000 | 0.00000 | 0.33333 | 0.00000 | 0.00000 | 0.16667 | 0.08333 | 0.00000 | 0.00000 | 0.00000 | 0.00000 | 0.00000 | 0.00000 | 0.12500 | 0.50000 |
| 9-078 | 0.50000 | 0.50000 | 0.25000 | 0.50000 | 0.66667 | 0.33333 | 0.25000 | 0.66667 | 0.41667 | 0.00000 | 0.33333 | 0.41667 | 0.40000 | 0.33333 | 0.50000 | 0.40000 | 0.33333 | 0.40000 | 0.50000 | 0.00000 |
| 9-079 | 0.50000 | 0.60000 | 0.33333 | 0.50000 | 0.66667 | 0.33333 | 0.25000 | 0.66667 | 0.41667 | 0.00000 | 0.25000 | 0.41667 | 0.40000 | 0.33333 | 0.50000 | 0.40000 | 0.33333 | 0.40000 | 0.62500 | 0.16667 |
| 9-080 | 0.00000 | 0.40000 | 0.25000 | 0.33333 | 0.66667 | 0.00000 | 0.00000 | 0.50000 | 0.00000 | 0.00000 | 0.00000 | 0.08333 | 0.00000 | 0.00000 | 0.00000 | 0.00000 | 0.00000 | 0.10000 | 0.25000 | 0.33333 |
| 9-081 | 0.00000 | 0.00000 | 0.08333 | 0.00000 | 0.16667 | 0.00000 | 0.00000 | 0.00000 | 0.00000 | 0.00000 | 0.00000 | 0.00000 | 0.00000 | 0.08333 | 0.00000 | 0.00000 | 0.00000 | 0.00000 | 0.00000 | 0.00000 |
| 9-082 | 0.00000 | 0.00000 | 0.08333 | 0.00000 | 0.00000 | 0.00000 | 0.00000 | 0.00000 | 0.00000 | 0.00000 | 0.00000 | 0.00000 | 0.00000 | 0.00000 | 0.00000 | 0.10000 | 0.00000 | 0.00000 | 0.00000 | 0.00000 |
| 9-083 | 0.00000 | 0.00000 | 0.08333 | 0.00000 | 0.00000 | 0.00000 | 0.16667 | 0.16667 | 0.16667 | 0.00000 | 0.00000 | 0.00000 | 0.00000 | 0.25000 | 0.00000 | 0.00000 | 0.16667 | 0.00000 | 0.00000 | 0.00000 |
| 9-084 | 0.00000 | 0.00000 | 0.08333 | 0.00000 | 0.00000 | 0.16667 | 0.00000 | 0.16667 | 0.16667 | 0.00000 | 0.00000 | 0.00000 | 0.00000 | 0.16667 | 0.30000 | 0.10000 | 0.16667 | 0.10000 | 0.12500 | 0.00000 |
| 9-085 | 0.25000 | 0.30000 | 0.16667 | 0.25000 | 0.25000 | 0.50000 | 0.33333 | 0.50000 | 0.50000 | 0.00000 | 0.25000 | 0.41667 | 0.50000 | 0.25000 | 0.20000 | 0.30000 | 0.33333 | 0.10000 | 0.37500 | 0.16667 |
| 9-086 | 0.12500 | 0.20000 | 0.16667 | 0.00000 | 0.08333 | 0.33333 | 0.16667 | 0.00000 | 0.08333 | 0.00000 | 0.00000 | 0.08333 | 0.20000 | 0.00000 | 0.30000 | 0.20000 | 0.00000 | 0.20000 | 0.12500 | 0.16667 |
| 9-087 | 0.12500 | 0.20000 | 0.08333 | 0.16667 | 0.16667 | 0.33333 | 0.25000 | 0.66667 | 0.33333 | 0.00000 | 0.25000 | 0.33333 | 0.40000 | 0.25000 | 0.30000 | 0.30000 | 0.33333 | 0.10000 | 0.50000 | 0.33333 |
| 9-088 | 0.00000 | 0.00000 | 0.00000 | 0.00000 | 0.00000 | 0.00000 | 0.08333 | 0.00000 | 0.00000 | 0.00000 | 0.00000 | 0.00000 | 0.00000 | 0.00000 | 0.00000 | 0.00000 | 0.00000 | 0.00000 | 0.00000 | 0.00000 |
| 9-089 | 0.37500 | 0.30000 | 0.41667 | 0.08333 | 0.33333 | 0.16667 | 0.08333 | 0.00000 | 0.08333 | 0.25000 | 0.16667 | 0.16667 | 0.10000 | 0.00000 | 0.00000 | 0.00000 | 0.08333 | 0.00000 | 0.00000 | 0.16667 |
| 9-090 | 0.50000 | 0.30000 | 0.41667 | 0.08333 | 0.33333 | 0.66667 | 0.75000 | 0.33333 | 0.58333 | 0.75000 | 0.58333 | 0.58333 | 0.50000 | 0.58333 | 0.30000 | 0.60000 | 0.16667 | 0.30000 | 0.12500 | 0.16667 |
| 9-091 | 0.50000 | 0.20000 | 0.41667 | 0.33333 | 0.33333 | 0.66667 | 0.66667 | 0.33333 | 0.58333 | 0.62500 | 0.58333 | 0.58333 | 0.50000 | 0.58333 | 0.30000 | 0.60000 | 0.25000 | 0.20000 | 0.25000 | 0.33333 |
| 9-092 | 0.00000 | 0.60000 | 0.41667 | 0.08333 | 0.33333 | 0.16667 | 0.08333 | 0.16667 | 0.08333 | 0.12500 | 0.08333 | 0.25000 | 0.00000 | 0.16667 | 0.00000 | 0.30000 | 0.00000 | 0.10000 | 0.25000 | 0.00000 |
| 9-093 | 0.00000 | 0.00000 | 0.08333 | 0.00000 | 0.00000 | 0.16667 | 0.00000 | 0.00000 | 0.00000 | 0.00000 | 0.00000 | 0.00000 | 0.00000 | 0.00000 | 0.10000 | 0.00000 | 0.00000 | 0.00000 | 0.00000 | 0.00000 |
| 9-094 | 0.00000 | 0.00000 | 0.00000 | 0.00000 | 0.00000 | 0.00000 | 0.08333 | 0.00000 | 0.08333 | 0.12500 | 0.00000 | 0.00000 | 0.10000 | 0.16667 | 0.00000 | 0.00000 | 0.08333 | 0.00000 | 0.00000 | 0.16667 |
| 9-095 | 0.00000 | 0.00000 | 0.00000 | 0.00000 | 0.00000 | 0.33333 | 0.16667 | 0.16667 | 0.00000 | 0.00000 | 0.00000 | 0.00000 | 0.20000 | 0.16667 | 0.10000 | 0.00000 | 0.00000 | 0.10000 | 0.12500 | 0.00000 |
| 9-096 | 0.00000 | 0.10000 | 0.00000 | 0.00000 | 0.08333 | 0.33333 | 0.33333 | 0.00000 | 0.25000 | 0.00000 | 0.00000 | 0.00000 | 0.20000 | 0.00000 | 0.00000 | 0.00000 | 0.00000 | 0.00000 | 0.00000 | 0.16667 |
| 9-097 | 0.25000 | 0.00000 | 0.08333 | 0.25000 | 0.08333 | 0.33333 | 0.00000 | 0.33333 | 0.41667 | 0.12500 | 0.25000 | 0.41667 | 0.40000 | 0.41667 | 0.20000 | 0.50000 | 0.16667 | 0.20000 | 0.12500 | 0.16667 |
| 9-098 | 0.00000 | 0.00000 | 0.08333 | 0.33333 | 0.00000 | 0.33333 | 0.16667 | 0.16667 | 0.00000 | 0.12500 | 0.00000 | 0.00000 | 0.10000 | 0.00000 | 0.30000 | 0.00000 | 0.41667 | 0.30000 | 0.12500 | 0.16667 |
| 9-099 | 0.00000 | 0.10000 | 0.08333 | 0.25000 | 0.08333 | 0.50000 | 0.25000 | 0.00000 | 0.16667 | 0.12500 | 0.00000 | 0.00000 | 0.20000 | 0.00000 | 0.00000 | 0.00000 | 0.41667 | 0.10000 | 0.00000 | 0.33333 |
| 9-100 | 0.25000 | 0.00000 | 0.00000 | 0.16667 | 0.00000 | 0.16667 | 0.16667 | 0.33333 | 0.41667 | 0.12500 | 0.25000 | 0.58333 | 0.30000 | 0.41667 | 0.20000 | 0.50000 | 0.16667 | 0.30000 | 0.12500 | 0.16667 |
| 9-101 | 0.00000 | 0.00000 | 0.00000 | 0.00000 | 0.00000 | 0.00000 | 0.00000 | 0.00000 | 0.00000 | 0.00000 | 0.00000 | 0.00000 | 0.00000 | 0.00000 | 0.00000 | 0.00000 | 0.16667 | 0.00000 | 0.00000 | 0.00000 |
| 9-102 | 0.00000 | 0.00000 | 0.00000 | 0.16667 | 0.00000 | 0.00000 | 0.00000 | 0.00000 | 0.00000 | 0.00000 | 0.00000 | 0.08333 | 0.00000 | 0.00000 | 0.00000 | 0.00000 | 0.41667 | 0.10000 | 0.00000 | 0.16667 |
| 9-103 | 0.00000 | 0.00000 | 0.00000 | 0.08333 | 0.00000 | 0.00000 | 0.00000 | 0.00000 | 0.00000 | 0.00000 | 0.00000 | 0.00000 | 0.00000 | 0.00000 | 0.00000 | 0.00000 | 0.33333 | 0.10000 | 0.00000 | 0.16667 |
| 9-104 | 0.00000 | 0.00000 | 0.00000 | 0.16667 | 0.00000 | 0.00000 | 0.00000 | 0.00000 | 0.00000 | 0.00000 | 0.00000 | 0.00000 | 0.00000 | 0.00000 | 0.00000 | 0.00000 | 0.00000 | 0.10000 | 0.00000 | 0.16667 |
| 9-105 | 0.00000 | 0.00000 | 0.00000 | 0.08333 | 0.00000 | 0.00000 | 0.00000 | 0.00000 | 0.00000 | 0.00000 | 0.08333 | 0.08333 | 0.00000 | 0.00000 | 0.00000 | 0.00000 | 0.33333 | 0.10000 | 0.00000 | 0.16667 |
| 9-106 | 0.00000 | 0.00000 | 0.00000 | 0.00000 | 0.00000 | 0.00000 | 0.00000 | 0.00000 | 0.00000 | 0.00000 | 0.00000 | 0.00000 | 0.00000 | 0.00000 | 0.00000 | 0.00000 | 0.08333 | 0.00000 | 0.00000 | 0.00000 |
| 9-107 | 0.12500 | 0.00000 | 0.00000 | 0.00000 | 0.00000 | 0.00000 | 0.00000 | 0.00000 | 0.00000 | 0.00000 | 0.00000 | 0.00000 | 0.00000 | 0.00000 | 0.00000 | 0.00000 | 0.00000 | 0.00000 | 0.00000 | 0.00000 |
| 9-108 | 0.00000 | 0.00000 | 0.00000 | 0.00000 | 0.00000 | 0.00000 | 0.00000 | 0.00000 | 0.00000 | 0.00000 | 0.00000 | 0.00000 | 0.00000 | 0.00000 | 0.10000 | 0.00000 | 0.00000 | 0.00000 | 0.00000 | 0.00000 |
| 9-109 | 0.00000 | 0.00000 | 0.00000 | 0.00000 | 0.00000 | 0.00000 | 0.16667 | 0.00000 | 0.08333 | 0.00000 | 0.00000 | 0.00000 | 0.10000 | 0.08333 | 0.00000 | 0.00000 | 0.08333 | 0.00000 | 0.00000 | 0.00000 |
| 9-110 | 0.00000 | 0.00000 | 0.08333 | 0.08333 | 0.00000 | 0.33333 | 0.08333 | 0.00000 | 0.16667 | 0.00000 | 0.08333 | 0.00000 | 0.10000 | 0.08333 | 0.00000 | 0.00000 | 0.08333 | 0.00000 | 0.12500 | 0.00000 |
| 9-111 | 0.00000 | 0.00000 | 0.00000 | 0.00000 | 0.00000 | 0.00000 | 0.00000 | 0.00000 | 0.00000 | 0.00000 | 0.00000 | 0.00000 | 0.00000 | 0.00000 | 0.00000 | 0.00000 | 0.00000 | 0.10000 | 0.00000 | 0.00000 |
| 12-001 | 0.00000 | 0.00000 | 0.00000 | 0.00000 | 0.00000 | 0.50000 | 0.50000 | 0.33333 | 0.33333 | 0.11111 | 0.45455 | 0.18182 | 0.00000 | 0.12500 | 0.00000 | 0.00000 | 0.10000 | 0.14286 | 0.00000 | 0.33333 |
| 12-002 | 0.00000 | 0.00000 | 0.00000 | 0.00000 | 0.00000 | 0.00000 | 0.08333 | 0.00000 | 0.00000 | 0.00000 | 0.00000 | 0.00000 | 0.00000 | 0.00000 | 0.00000 | 0.00000 | 0.00000 | 0.00000 | 0.00000 | 0.00000 |
| 12-003 | 0.00000 | 0.00000 | 0.00000 | 0.00000 | 0.08333 | 0.00000 | 0.08333 | 0.00000 | 0.00000 | 0.00000 | 0.09091 | 0.09091 | 0.00000 | 0.00000 | 0.10000 | 0.00000 | 0.10000 | 0.00000 | 0.00000 | 0.00000 |
| 12-004 | 0.00000 | 0.00000 | 0.00000 | 0.00000 | 0.00000 | 0.16667 | 0.08333 | 0.33333 | 0.00000 | 0.00000 | 0.09091 | 0.09091 | 0.00000 | 0.00000 | 0.00000 | 0.00000 | 0.00000 | 0.00000 | 0.00000 | 0.00000 |
| 12-005 | 0.00000 | 0.00000 | 0.00000 | 0.00000 | 0.08333 | 0.00000 | 0.00000 | 0.00000 | 0.00000 | 0.00000 | 0.00000 | 0.00000 | 0.00000 | 0.00000 | 0.00000 | 0.00000 | 0.00000 | 0.00000 | 0.00000 | 0.00000 |
| 12-006 | 0.00000 | 0.00000 | 0.08333 | 0.09091 | 0.08333 | 0.00000 | 0.08333 | 0.00000 | 0.08333 | 0.00000 | 0.00000 | 0.00000 | 0.00000 | 0.00000 | 0.00000 | 0.00000 | 0.00000 | 0.00000 | 0.00000 | 0.00000 |
| 12-007 | 0.00000 | 0.00000 | 0.00000 | 0.00000 | 0.08333 | 0.16667 | 0.08333 | 0.16667 | 0.08333 | 0.00000 | 0.00000 | 0.00000 | 0.10000 | 0.25000 | 0.00000 | 0.00000 | 0.00000 | 0.00000 | 0.00000 | 0.00000 |
| 12-008 | 0.00000 | 0.00000 | 0.08333 | 0.00000 | 0.08333 | 0.16667 | 0.08333 | 0.16667 | 0.00000 | 0.00000 | 0.00000 | 0.00000 | 0.00000 | 0.00000 | 0.00000 | 0.00000 | 0.00000 | 0.00000 | 0.00000 | 0.00000 |
| 12-009 | 0.00000 | 0.10000 | 0.08333 | 0.00000 | 0.00000 | 0.16667 | 0.08333 | 0.16667 | 0.00000 | 0.00000 | 0.27273 | 0.18182 | 0.10000 | 0.12500 | 0.00000 | 0.00000 | 0.00000 | 0.00000 | 0.00000 | 0.00000 |
| 12-010 | 0.00000 | 0.00000 | 0.00000 | 0.00000 | 0.00000 | 0.00000 | 0.00000 | 0.33333 | 0.00000 | 0.00000 | 0.09091 | 0.00000 | 0.00000 | 0.00000 | 0.00000 | 0.00000 | 0.00000 | 0.00000 | 0.00000 | 0.00000 |
| 12-011 | 0.50000 | 0.60000 | 0.50000 | 0.72727 | 0.66667 | 0.50000 | 0.25000 | 0.33333 | 0.33333 | 0.11111 | 0.00000 | 0.36364 | 0.30000 | 0.25000 | 0.70000 | 0.28571 | 0.60000 | 0.00000 | 0.37500 | 0.33333 |
| 12-012 | 0.00000 | 0.00000 | 0.00000 | 0.00000 | 0.08333 | 0.16667 | 0.08333 | 0.00000 | 0.00000 | 0.00000 | 0.00000 | 0.09091 | 0.00000 | 0.00000 | 0.00000 | 0.00000 | 0.00000 | 0.00000 | 0.00000 | 0.00000 |
| 12-013 | 0.00000 | 0.00000 | 0.08333 | 0.00000 | 0.25000 | 0.00000 | 0.08333 | 0.00000 | 0.00000 | 0.00000 | 0.18182 | 0.09091 | 0.00000 | 0.00000 | 0.10000 | 0.00000 | 0.10000 | 0.00000 | 0.00000 | 0.00000 |
| 12-014 | 0.00000 | 0.00000 | 0.00000 | 0.00000 | 0.08333 | 0.16667 | 0.08333 | 0.00000 | 0.00000 | 0.00000 | 0.00000 | 0.09091 | 0.10000 | 0.00000 | 0.00000 | 0.00000 | 0.00000 | 0.00000 | 0.00000 | 0.00000 |
| 12-015 | 0.00000 | 0.00000 | 0.00000 | 0.00000 | 0.00000 | 0.00000 | 0.08333 | 0.00000 | 0.00000 | 0.00000 | 0.00000 | 0.00000 | 0.00000 | 0.00000 | 0.00000 | 0.00000 | 0.00000 | 0.00000 | 0.00000 | 0.00000 |
| 12-016 | 0.00000 | 0.10000 | 0.00000 | 0.00000 | 0.00000 | 0.16667 | 0.00000 | 0.16667 | 0.00000 | 0.00000 | 0.18182 | 0.18182 | 0.00000 | 0.00000 | 0.00000 | 0.00000 | 0.00000 | 0.00000 | 0.00000 | 0.00000 |
| 12-017 | 0.00000 | 0.10000 | 0.08333 | 0.00000 | 0.08333 | 0.16667 | 0.00000 | 0.00000 | 0.08333 | 0.00000 | 0.09091 | 0.00000 | 0.10000 | 0.25000 | 0.00000 | 0.00000 | 0.00000 | 0.00000 | 0.00000 | 0.00000 |
| 12-018 | 0.00000 | 0.00000 | 0.00000 | 0.00000 | 0.00000 | 0.16667 | 0.00000 | 0.00000 | 0.08333 | 0.00000 | 0.00000 | 0.00000 | 0.00000 | 0.00000 | 0.00000 | 0.00000 | 0.00000 | 0.00000 | 0.00000 | 0.00000 |
| 12-019 | 0.00000 | 0.00000 | 0.08333 | 0.00000 | 0.08333 | 0.00000 | 0.00000 | 0.00000 | 0.00000 | 0.00000 | 0.09091 | 0.09091 | 0.00000 | 0.00000 | 0.10000 | 0.00000 | 0.00000 | 0.00000 | 0.00000 | 0.00000 |
| 12-020 | 0.00000 | 0.20000 | 0.25000 | 0.18182 | 0.16667 | 0.50000 | 0.58333 | 0.66667 | 0.33333 | 0.11111 | 0.36364 | 0.09091 | 0.00000 | 0.12500 | 0.00000 | 0.14286 | 0.00000 | 0.00000 | 0.00000 | 0.33333 |
| 12-021 | 0.00000 | 0.00000 | 0.08333 | 0.00000 | 0.08333 | 0.16667 | 0.08333 | 0.16667 | 0.08333 | 0.00000 | 0.18182 | 0.18182 | 0.00000 | 0.00000 | 0.00000 | 0.00000 | 0.10000 | 0.00000 | 0.00000 | 0.00000 |
| 12-022 | 0.00000 | 0.00000 | 0.16667 | 0.00000 | 0.16667 | 0.33333 | 0.08333 | 0.33333 | 0.00000 | 0.00000 | 0.27273 | 0.27273 | 0.00000 | 0.00000 | 0.10000 | 0.00000 | 0.10000 | 0.00000 | 0.00000 | 0.00000 |
| 12-023 | 0.00000 | 0.10000 | 0.08333 | 0.00000 | 0.08333 | 0.33333 | 0.00000 | 0.16667 | 0.00000 | 0.00000 | 0.27273 | 0.09091 | 0.00000 | 0.12500 | 0.00000 | 0.00000 | 0.00000 | 0.00000 | 0.00000 | 0.00000 |
| 12-024 | 0.00000 | 0.00000 | 0.00000 | 0.00000 | 0.08333 | 0.00000 | 0.08333 | 0.16667 | 0.00000 | 0.00000 | 0.18182 | 0.00000 | 0.00000 | 0.00000 | 0.00000 | 0.00000 | 0.00000 | 0.00000 | 0.00000 | 0.00000 |
| 12-025 | 0.00000 | 0.00000 | 0.08333 | 0.00000 | 0.00000 | 0.00000 | 0.08333 | 0.33333 | 0.00000 | 0.00000 | 0.09091 | 0.09091 | 0.00000 | 0.00000 | 0.00000 | 0.00000 | 0.00000 | 0.00000 | 0.00000 | 0.00000 |
| 12-026 | 0.00000 | 0.10000 | 0.08333 | 0.00000 | 0.00000 | 0.16667 | 0.00000 | 0.00000 | 0.00000 | 0.00000 | 0.00000 | 0.09091 | 0.10000 | 0.25000 | 0.00000 | 0.00000 | 0.00000 | 0.00000 | 0.00000 | 0.00000 |
| 12-027 | 0.00000 | 0.10000 | 0.00000 | 0.09091 | 0.16667 | 0.00000 | 0.00000 | 0.16667 | 0.16667 | 0.00000 | 0.18182 | 0.00000 | 0.30000 | 0.12500 | 0.20000 | 0.14286 | 0.00000 | 0.28571 | 0.00000 | 0.00000 |
| 12-028 | 0.00000 | 0.00000 | 0.00000 | 0.00000 | 0.08333 | 0.16667 | 0.16667 | 0.00000 | 0.00000 | 0.00000 | 0.00000 | 0.00000 | 0.10000 | 0.00000 | 0.20000 | 0.00000 | 0.30000 | 0.28571 | 0.12500 | 0.33333 |
| 12-029 | 0.00000 | 0.00000 | 0.00000 | 0.00000 | 0.00000 | 0.16667 | 0.00000 | 0.00000 | 0.16667 | 0.00000 | 0.00000 | 0.00000 | 0.10000 | 0.12500 | 0.00000 | 0.00000 | 0.00000 | 0.00000 | 0.00000 | 0.00000 |
| 12-030 | 0.00000 | 0.00000 | 0.00000 | 0.00000 | 0.08333 | 0.00000 | 0.08333 | 0.16667 | 0.08333 | 0.00000 | 0.09091 | 0.00000 | 0.10000 | 0.25000 | 0.10000 | 0.14286 | 0.00000 | 0.00000 | 0.00000 | 0.00000 |
| 12-031 | 0.00000 | 0.20000 | 0.25000 | 0.00000 | 0.16667 | 0.33333 | 0.16667 | 0.16667 | 0.08333 | 0.00000 | 0.00000 | 0.00000 | 0.10000 | 0.25000 | 0.00000 | 0.00000 | 0.00000 | 0.00000 | 0.00000 | 0.00000 |
| 12-032 | 0.00000 | 0.10000 | 0.08333 | 0.00000 | 0.16667 | 0.50000 | 0.08333 | 0.16667 | 0.08333 | 0.00000 | 0.00000 | 0.00000 | 0.00000 | 0.12500 | 0.00000 | 0.00000 | 0.00000 | 0.00000 | 0.00000 | 0.00000 |
| 12-033 | 0.00000 | 0.00000 | 0.00000 | 0.09091 | 0.00000 | 0.33333 | 0.08333 | 0.16667 | 0.00000 | 0.00000 | 0.00000 | 0.00000 | 0.00000 | 0.00000 | 0.00000 | 0.00000 | 0.00000 | 0.00000 | 0.12500 | 0.00000 |
| 12-034 | 0.50000 | 0.50000 | 0.41667 | 0.45455 | 0.58333 | 0.16667 | 0.25000 | 0.33333 | 0.33333 | 0.88889 | 0.09091 | 0.00000 | 0.70000 | 0.12500 | 0.80000 | 0.28571 | 0.00000 | 0.00000 | 0.87500 | 0.00000 |
| 12-035 | 0.00000 | 0.00000 | 0.00000 | 0.00000 | 0.08333 | 0.00000 | 0.00000 | 0.00000 | 0.00000 | 0.00000 | 0.18182 | 0.09091 | 0.10000 | 0.00000 | 0.10000 | 0.00000 | 0.00000 | 0.00000 | 0.00000 | 0.00000 |
| 12-036 | 0.00000 | 0.00000 | 0.08333 | 0.00000 | 0.00000 | 0.00000 | 0.16667 | 0.50000 | 0.16667 | 0.00000 | 0.00000 | 0.00000 | 0.00000 | 0.00000 | 0.00000 | 0.14286 | 0.00000 | 0.00000 | 0.00000 | 0.33333 |
| 12-037 | 0.00000 | 0.30000 | 0.50000 | 0.63636 | 0.41667 | 0.66667 | 0.50000 | 0.50000 | 0.41667 | 0.11111 | 0.18182 | 0.36364 | 0.50000 | 0.25000 | 0.10000 | 0.28571 | 0.40000 | 0.28571 | 0.37500 | 0.83333 |
| 12-038 | 0.00000 | 0.40000 | 0.41667 | 0.72727 | 0.50000 | 0.33333 | 0.25000 | 0.33333 | 0.58333 | 0.33333 | 0.18182 | 0.54545 | 0.90000 | 0.37500 | 0.70000 | 0.71429 | 0.70000 | 0.71429 | 0.62500 | 0.33333 |
| 12-039 | 0.00000 | 0.00000 | 0.08333 | 0.00000 | 0.16667 | 0.16667 | 0.16667 | 0.33333 | 0.08333 | 0.11111 | 0.18182 | 0.00000 | 0.00000 | 0.00000 | 0.00000 | 0.00000 | 0.00000 | 0.00000 | 0.00000 | 0.16667 |
| 12-040 | 0.00000 | 0.10000 | 0.08333 | 0.00000 | 0.08333 | 0.16667 | 0.25000 | 0.33333 | 0.08333 | 0.00000 | 0.18182 | 0.00000 | 0.00000 | 0.00000 | 0.00000 | 0.00000 | 0.00000 | 0.00000 | 0.00000 | 0.00000 |
| 12-041 | 0.16667 | 0.10000 | 0.00000 | 0.00000 | 0.00000 | 0.16667 | 0.16667 | 0.16667 | 0.08333 | 0.00000 | 0.00000 | 0.09091 | 0.00000 | 0.25000 | 0.00000 | 0.00000 | 0.00000 | 0.00000 | 0.00000 | 0.00000 |
| 12-042 | 0.00000 | 0.10000 | 0.00000 | 0.00000 | 0.00000 | 0.33333 | 0.08333 | 0.00000 | 0.08333 | 0.00000 | 0.00000 | 0.36364 | 0.00000 | 0.00000 | 0.00000 | 0.00000 | 0.00000 | 0.00000 | 0.00000 | 0.00000 |
| 12-043 | 0.00000 | 0.00000 | 0.00000 | 0.00000 | 0.00000 | 0.00000 | 0.00000 | 0.16667 | 0.00000 | 0.00000 | 0.00000 | 0.00000 | 0.00000 | 0.00000 | 0.00000 | 0.00000 | 0.00000 | 0.00000 | 0.00000 | 0.00000 |
| 12-044 | 0.00000 | 0.20000 | 0.25000 | 0.09091 | 0.08333 | 0.00000 | 0.50000 | 0.33333 | 0.08333 | 0.11111 | 0.18182 | 0.09091 | 0.00000 | 0.12500 | 0.00000 | 0.00000 | 0.00000 | 0.00000 | 0.00000 | 0.33333 |
| 12-045 | 0.00000 | 0.00000 | 0.00000 | 0.00000 | 0.00000 | 0.00000 | 0.00000 | 0.00000 | 0.08333 | 0.00000 | 0.00000 | 0.00000 | 0.00000 | 0.00000 | 0.00000 | 0.00000 | 0.00000 | 0.00000 | 0.00000 | 0.00000 |
| 12-046 | 0.00000 | 0.10000 | 0.16667 | 0.09091 | 0.00000 | 0.33333 | 0.50000 | 0.50000 | 0.33333 | 0.11111 | 0.18182 | 0.09091 | 0.00000 | 0.25000 | 0.00000 | 0.14286 | 0.00000 | 0.14286 | 0.00000 | 0.66667 |
| 12-047 | 0.00000 | 0.10000 | 0.25000 | 0.09091 | 0.00000 | 0.33333 | 0.58333 | 0.50000 | 0.33333 | 0.11111 | 0.27273 | 0.09091 | 0.00000 | 0.25000 | 0.00000 | 0.14286 | 0.10000 | 0.14286 | 0.00000 | 0.66667 |
| 12-048 | 0.00000 | 0.00000 | 0.25000 | 0.18182 | 0.16667 | 0.33333 | 0.50000 | 0.16667 | 0.08333 | 0.00000 | 0.18182 | 0.00000 | 0.00000 | 0.00000 | 0.00000 | 0.00000 | 0.00000 | 0.14286 | 0.00000 | 0.16667 |
| 12-049 | 0.00000 | 0.00000 | 0.08333 | 0.09091 | 0.16667 | 0.00000 | 0.25000 | 0.00000 | 0.08333 | 0.00000 | 0.18182 | 0.00000 | 0.00000 | 0.00000 | 0.00000 | 0.00000 | 0.00000 | 0.00000 | 0.00000 | 0.00000 |
| 12-050 | 0.50000 | 0.10000 | 0.00000 | 0.09091 | 0.25000 | 0.16667 | 0.16667 | 0.16667 | 0.16667 | 0.00000 | 0.00000 | 0.09091 | 0.00000 | 0.00000 | 0.00000 | 0.00000 | 0.00000 | 0.00000 | 0.00000 | 0.00000 |
| 12-051 | 0.16667 | 0.30000 | 0.50000 | 0.45455 | 0.33333 | 0.16667 | 0.25000 | 0.16667 | 0.16667 | 0.00000 | 0.00000 | 0.00000 | 0.00000 | 0.00000 | 0.00000 | 0.00000 | 0.00000 | 0.00000 | 0.00000 | 0.00000 |
| 12-052 | 0.16667 | 0.30000 | 0.33333 | 0.27273 | 0.41667 | 0.00000 | 0.16667 | 0.16667 | 0.08333 | 0.00000 | 0.00000 | 0.00000 | 0.00000 | 0.00000 | 0.00000 | 0.00000 | 0.00000 | 0.00000 | 0.00000 | 0.00000 |
| 12-053 | 0.00000 | 0.00000 | 0.08333 | 0.00000 | 0.00000 | 0.00000 | 0.08333 | 0.00000 | 0.00000 | 0.00000 | 0.00000 | 0.00000 | 0.00000 | 0.00000 | 0.00000 | 0.00000 | 0.00000 | 0.00000 | 0.00000 | 0.00000 |
| 12-054 | 0.00000 | 0.00000 | 0.16667 | 0.09091 | 0.00000 | 0.00000 | 0.00000 | 0.00000 | 0.00000 | 0.00000 | 0.00000 | 0.00000 | 0.00000 | 0.00000 | 0.00000 | 0.14286 | 0.00000 | 0.00000 | 0.00000 | 0.16667 |
| 12-055 | 0.00000 | 0.00000 | 0.08333 | 0.00000 | 0.08333 | 0.00000 | 0.00000 | 0.00000 | 0.00000 | 0.00000 | 0.00000 | 0.00000 | 0.00000 | 0.00000 | 0.00000 | 0.00000 | 0.00000 | 0.00000 | 0.00000 | 0.00000 |
| 12-056 | 0.00000 | 0.00000 | 0.16667 | 0.09091 | 0.00000 | 0.00000 | 0.08333 | 0.00000 | 0.00000 | 0.00000 | 0.00000 | 0.00000 | 0.00000 | 0.00000 | 0.00000 | 0.00000 | 0.00000 | 0.00000 | 0.00000 | 0.00000 |
| 12-057 | 0.00000 | 0.00000 | 0.33333 | 0.18182 | 0.00000 | 0.00000 | 0.16667 | 0.00000 | 0.16667 | 0.00000 | 0.00000 | 0.00000 | 0.00000 | 0.00000 | 0.00000 | 0.00000 | 0.00000 | 0.14286 | 0.00000 | 0.00000 |
| 12-058 | 0.00000 | 0.10000 | 0.33333 | 0.18182 | 0.00000 | 0.16667 | 0.41667 | 0.33333 | 0.25000 | 0.00000 | 0.09091 | 0.09091 | 0.00000 | 0.00000 | 0.00000 | 0.00000 | 0.00000 | 0.14286 | 0.00000 | 0.00000 |
| 12-059 | 0.00000 | 0.00000 | 0.33333 | 0.09091 | 0.00000 | 0.00000 | 0.41667 | 0.00000 | 0.08333 | 0.00000 | 0.00000 | 0.00000 | 0.00000 | 0.00000 | 0.00000 | 0.00000 | 0.00000 | 0.00000 | 0.00000 | 0.00000 |
| 12-060 | 0.00000 | 0.10000 | 0.25000 | 0.09091 | 0.08333 | 0.16667 | 0.50000 | 0.16667 | 0.08333 | 0.11111 | 0.18182 | 0.00000 | 0.00000 | 0.12500 | 0.00000 | 0.14286 | 0.00000 | 0.00000 | 0.00000 | 0.00000 |
| 12-061 | 0.00000 | 0.00000 | 0.00000 | 0.00000 | 0.00000 | 0.16667 | 0.00000 | 0.33333 | 0.16667 | 0.00000 | 0.00000 | 0.09091 | 0.00000 | 0.12500 | 0.00000 | 0.00000 | 0.00000 | 0.14286 | 0.00000 | 0.66667 |
| 12-062 | 0.00000 | 0.20000 | 0.16667 | 0.09091 | 0.00000 | 0.00000 | 0.00000 | 0.00000 | 0.00000 | 0.00000 | 0.00000 | 0.00000 | 0.00000 | 0.00000 | 0.00000 | 0.00000 | 0.00000 | 0.00000 | 0.00000 | 0.00000 |
| 12-063 | 0.00000 | 0.00000 | 0.00000 | 0.00000 | 0.00000 | 0.00000 | 0.08333 | 0.00000 | 0.00000 | 0.00000 | 0.00000 | 0.00000 | 0.00000 | 0.00000 | 0.00000 | 0.00000 | 0.00000 | 0.00000 | 0.00000 | 0.00000 |
| 12-064 | 0.00000 | 0.00000 | 0.08333 | 0.00000 | 0.00000 | 0.00000 | 0.00000 | 0.00000 | 0.00000 | 0.00000 | 0.00000 | 0.00000 | 0.00000 | 0.00000 | 0.00000 | 0.00000 | 0.00000 | 0.00000 | 0.00000 | 0.00000 |
| 12-065 | 0.00000 | 0.00000 | 0.00000 | 0.09091 | 0.00000 | 0.00000 | 0.08333 | 0.00000 | 0.08333 | 0.00000 | 0.00000 | 0.00000 | 0.00000 | 0.00000 | 0.00000 | 0.00000 | 0.00000 | 0.00000 | 0.00000 | 0.00000 |
| 12-066 | 0.00000 | 0.00000 | 0.08333 | 0.00000 | 0.00000 | 0.00000 | 0.00000 | 0.00000 | 0.00000 | 0.00000 | 0.00000 | 0.00000 | 0.00000 | 0.00000 | 0.00000 | 0.00000 | 0.00000 | 0.00000 | 0.00000 | 0.00000 |
| 12-067 | 0.00000 | 0.00000 | 0.08333 | 0.00000 | 0.00000 | 0.00000 | 0.00000 | 0.00000 | 0.08333 | 0.00000 | 0.00000 | 0.00000 | 0.00000 | 0.00000 | 0.00000 | 0.00000 | 0.00000 | 0.00000 | 0.00000 | 0.00000 |
| 12-068 | 0.00000 | 0.00000 | 0.00000 | 0.00000 | 0.00000 | 0.00000 | 0.08333 | 0.00000 | 0.00000 | 0.00000 | 0.00000 | 0.00000 | 0.00000 | 0.00000 | 0.00000 | 0.00000 | 0.00000 | 0.00000 | 0.00000 | 0.00000 |
| 12-069 | 0.00000 | 0.10000 | 0.00000 | 0.00000 | 0.00000 | 0.00000 | 0.00000 | 0.00000 | 0.00000 | 0.00000 | 0.00000 | 0.00000 | 0.00000 | 0.00000 | 0.00000 | 0.00000 | 0.00000 | 0.00000 | 0.00000 | 0.00000 |
| 12-070 | 0.00000 | 0.10000 | 0.00000 | 0.00000 | 0.00000 | 0.33333 | 0.08333 | 0.16667 | 0.08333 | 0.00000 | 0.00000 | 0.09091 | 0.00000 | 0.00000 | 0.00000 | 0.00000 | 0.00000 | 0.00000 | 0.00000 | 0.00000 |
| 12-071 | 0.00000 | 0.00000 | 0.00000 | 0.00000 | 0.00000 | 0.16667 | 0.08333 | 0.16667 | 0.00000 | 0.00000 | 0.00000 | 0.00000 | 0.00000 | 0.00000 | 0.00000 | 0.00000 | 0.00000 | 0.00000 | 0.00000 | 0.00000 |
| 12-072 | 0.00000 | 0.00000 | 0.08333 | 0.09091 | 0.00000 | 0.00000 | 0.00000 | 0.00000 | 0.00000 | 0.00000 | 0.00000 | 0.00000 | 0.00000 | 0.00000 | 0.00000 | 0.00000 | 0.00000 | 0.00000 | 0.00000 | 0.00000 |
| 12-073 | 0.16667 | 0.10000 | 0.16667 | 0.00000 | 0.08333 | 0.33333 | 0.08333 | 0.00000 | 0.00000 | 0.00000 | 0.00000 | 0.00000 | 0.00000 | 0.00000 | 0.00000 | 0.00000 | 0.00000 | 0.00000 | 0.00000 | 0.00000 |
| 12-074 | 0.83333 | 0.00000 | 0.00000 | 0.00000 | 0.00000 | 0.16667 | 0.08333 | 0.00000 | 0.00000 | 0.00000 | 0.09091 | 0.00000 | 0.00000 | 0.00000 | 0.00000 | 0.00000 | 0.00000 | 0.00000 | 0.00000 | 0.16667 |
| 12-075 | 0.00000 | 0.00000 | 0.00000 | 0.00000 | 0.00000 | 0.00000 | 0.00000 | 0.00000 | 0.00000 | 0.00000 | 0.18182 | 0.00000 | 0.00000 | 0.00000 | 0.00000 | 0.00000 | 0.00000 | 0.00000 | 0.00000 | 0.16667 |
| 12-076 | 0.16667 | 0.00000 | 0.00000 | 0.00000 | 0.00000 | 0.00000 | 0.00000 | 0.00000 | 0.08333 | 0.00000 | 0.00000 | 0.00000 | 0.00000 | 0.00000 | 0.00000 | 0.00000 | 0.00000 | 0.00000 | 0.00000 | 0.00000 |
| 12-077 | 0.16667 | 0.00000 | 0.16667 | 0.00000 | 0.08333 | 0.00000 | 0.00000 | 0.00000 | 0.08333 | 0.00000 | 0.00000 | 0.00000 | 0.00000 | 0.00000 | 0.00000 | 0.00000 | 0.00000 | 0.00000 | 0.00000 | 0.00000 |
| 12-078 | 0.00000 | 0.00000 | 0.00000 | 0.00000 | 0.00000 | 0.00000 | 0.00000 | 0.00000 | 0.00000 | 0.11111 | 0.00000 | 0.00000 | 0.00000 | 0.00000 | 0.00000 | 0.00000 | 0.00000 | 0.14286 | 0.00000 | 0.00000 |
| 12-079 | 0.00000 | 0.00000 | 0.00000 | 0.00000 | 0.00000 | 0.16667 | 0.00000 | 0.00000 | 0.00000 | 0.00000 | 0.00000 | 0.00000 | 0.00000 | 0.00000 | 0.00000 | 0.00000 | 0.00000 | 0.00000 | 0.00000 | 0.00000 |
| 12-080 | 0.00000 | 0.00000 | 0.00000 | 0.00000 | 0.00000 | 0.16667 | 0.00000 | 0.00000 | 0.00000 | 0.11111 | 0.00000 | 0.00000 | 0.00000 | 0.00000 | 0.00000 | 0.00000 | 0.00000 | 0.00000 | 0.00000 | 0.00000 |
| 12-081 | 0.00000 | 0.00000 | 0.08333 | 0.00000 | 0.00000 | 0.00000 | 0.00000 | 0.00000 | 0.00000 | 0.00000 | 0.00000 | 0.00000 | 0.00000 | 0.00000 | 0.00000 | 0.00000 | 0.00000 | 0.00000 | 0.00000 | 0.00000 |
| 12-082 | 0.00000 | 0.00000 | 0.08333 | 0.00000 | 0.00000 | 0.00000 | 0.00000 | 0.00000 | 0.00000 | 0.00000 | 0.00000 | 0.00000 | 0.00000 | 0.00000 | 0.00000 | 0.00000 | 0.00000 | 0.00000 | 0.00000 | 0.00000 |
| 12-083 | 0.00000 | 0.00000 | 0.00000 | 0.00000 | 0.00000 | 0.00000 | 0.00000 | 0.16667 | 0.00000 | 0.00000 | 0.00000 | 0.00000 | 0.00000 | 0.00000 | 0.00000 | 0.00000 | 0.00000 | 0.00000 | 0.00000 | 0.00000 |
| 12-084 | 0.00000 | 0.00000 | 0.00000 | 0.00000 | 0.00000 | 0.00000 | 0.00000 | 0.16667 | 0.00000 | 0.00000 | 0.00000 | 0.00000 | 0.00000 | 0.00000 | 0.00000 | 0.00000 | 0.00000 | 0.00000 | 0.00000 | 0.66667 |
| 12-085 | 0.00000 | 0.00000 | 0.00000 | 0.00000 | 0.00000 | 0.00000 | 0.00000 | 0.00000 | 0.00000 | 0.00000 | 0.09091 | 0.00000 | 0.00000 | 0.00000 | 0.00000 | 0.00000 | 0.00000 | 0.00000 | 0.00000 | 0.66667 |
| 12-086 | 0.00000 | 0.00000 | 0.00000 | 0.00000 | 0.00000 | 0.00000 | 0.00000 | 0.00000 | 0.00000 | 0.11111 | 0.18182 | 0.00000 | 0.00000 | 0.00000 | 0.00000 | 0.00000 | 0.00000 | 0.00000 | 0.00000 | 0.00000 |
| 12-087 | 0.00000 | 0.00000 | 0.08333 | 0.00000 | 0.00000 | 0.00000 | 0.00000 | 0.16667 | 0.00000 | 0.00000 | 0.00000 | 0.00000 | 0.00000 | 0.00000 | 0.00000 | 0.00000 | 0.00000 | 0.00000 | 0.00000 | 0.00000 |
| 12-088 | 0.00000 | 0.00000 | 0.00000 | 0.00000 | 0.00000 | 0.00000 | 0.00000 | 0.00000 | 0.00000 | 0.00000 | 0.00000 | 0.00000 | 0.00000 | 0.00000 | 0.00000 | 0.00000 | 0.00000 | 0.00000 | 0.00000 | 0.16667 |
| 12-089 | 0.00000 | 0.00000 | 0.00000 | 0.09091 | 0.00000 | 0.00000 | 0.00000 | 0.00000 | 0.00000 | 0.00000 | 0.09091 | 0.00000 | 0.00000 | 0.00000 | 0.00000 | 0.00000 | 0.00000 | 0.00000 | 0.00000 | 0.00000 |
| 12-090 | 0.00000 | 0.00000 | 0.00000 | 0.45455 | 0.16667 | 0.50000 | 0.33333 | 0.33333 | 0.08333 | 0.11111 | 0.09091 | 0.00000 | 0.00000 | 0.00000 | 0.00000 | 0.00000 | 0.00000 | 0.28571 | 0.12500 | 0.00000 |
| 12-091 | 0.00000 | 0.00000 | 0.00000 | 0.09091 | 0.00000 | 0.16667 | 0.33333 | 0.16667 | 0.16667 | 0.11111 | 0.09091 | 0.00000 | 0.00000 | 0.00000 | 0.00000 | 0.14286 | 0.10000 | 0.42857 | 0.25000 | 0.66667 |
| 12-092 | 0.00000 | 0.10000 | 0.08333 | 0.27273 | 0.25000 | 0.16667 | 0.00000 | 0.16667 | 0.16667 | 0.00000 | 0.00000 | 0.00000 | 0.00000 | 0.00000 | 0.00000 | 0.00000 | 0.00000 | 0.00000 | 0.00000 | 0.33333 |
| 12-093 | 0.00000 | 0.00000 | 0.00000 | 0.09091 | 0.00000 | 0.00000 | 0.25000 | 0.00000 | 0.08333 | 0.00000 | 0.00000 | 0.00000 | 0.00000 | 0.00000 | 0.00000 | 0.00000 | 0.00000 | 0.28571 | 0.12500 | 0.00000 |
| 12-094 | 0.00000 | 0.00000 | 0.00000 | 0.00000 | 0.00000 | 0.33333 | 0.41667 | 0.16667 | 0.08333 | 0.22222 | 0.45455 | 0.00000 | 0.00000 | 0.00000 | 0.00000 | 0.00000 | 0.00000 | 0.00000 | 0.00000 | 0.00000 |
| 12-095 | 0.16667 | 0.00000 | 0.00000 | 0.00000 | 0.00000 | 0.00000 | 0.08333 | 0.00000 | 0.00000 | 0.00000 | 0.00000 | 0.00000 | 0.00000 | 0.00000 | 0.00000 | 0.00000 | 0.00000 | 0.14286 | 0.00000 | 0.83333 |
| 12-096 | 0.00000 | 0.00000 | 0.08333 | 0.00000 | 0.00000 | 0.00000 | 0.16667 | 0.00000 | 0.00000 | 0.00000 | 0.00000 | 0.00000 | 0.00000 | 0.00000 | 0.00000 | 0.00000 | 0.00000 | 0.00000 | 0.00000 | 0.83333 |
| 12-097 | 0.00000 | 0.00000 | 0.00000 | 0.00000 | 0.00000 | 0.00000 | 0.00000 | 0.00000 | 0.00000 | 0.00000 | 0.00000 | 0.00000 | 0.00000 | 0.00000 | 0.00000 | 0.00000 | 0.00000 | 0.00000 | 0.00000 | 0.16667 |
| 12-098 | 0.00000 | 0.00000 | 0.00000 | 0.00000 | 0.08333 | 0.00000 | 0.00000 | 0.00000 | 0.00000 | 0.00000 | 0.00000 | 0.00000 | 0.00000 | 0.00000 | 0.00000 | 0.00000 | 0.00000 | 0.00000 | 0.00000 | 0.00000 |
| 12-099 | 0.00000 | 0.00000 | 0.00000 | 0.00000 | 0.00000 | 0.00000 | 0.08333 | 0.00000 | 0.00000 | 0.00000 | 0.00000 | 0.00000 | 0.00000 | 0.00000 | 0.00000 | 0.00000 | 0.00000 | 0.00000 | 0.00000 | 0.00000 |
| 12-100 | 0.00000 | 0.10000 | 0.08333 | 0.00000 | 0.08333 | 0.16667 | 0.08333 | 0.16667 | 0.00000 | 0.00000 | 0.00000 | 0.27273 | 0.00000 | 0.00000 | 0.10000 | 0.00000 | 0.10000 | 0.00000 | 0.00000 | 0.00000 |
| 12-101 | 0.00000 | 0.00000 | 0.00000 | 0.00000 | 0.00000 | 0.00000 | 0.00000 | 0.00000 | 0.00000 | 0.00000 | 0.27273 | 0.00000 | 0.00000 | 0.00000 | 0.00000 | 0.00000 | 0.00000 | 0.00000 | 0.00000 | 0.00000 |
| 12-102 | 0.00000 | 0.10000 | 0.00000 | 0.00000 | 0.00000 | 0.16667 | 0.00000 | 0.00000 | 0.08333 | 0.00000 | 0.00000 | 0.00000 | 0.10000 | 0.00000 | 0.00000 | 0.00000 | 0.00000 | 0.00000 | 0.00000 | 0.00000 |
| 12-103 | 0.00000 | 0.00000 | 0.00000 | 0.00000 | 0.00000 | 0.00000 | 0.00000 | 0.00000 | 0.00000 | 0.00000 | 0.00000 | 0.00000 | 0.00000 | 0.25000 | 0.00000 | 0.00000 | 0.00000 | 0.00000 | 0.00000 | 0.00000 |
| 12-104 | 0.00000 | 0.00000 | 0.00000 | 0.00000 | 0.00000 | 0.00000 | 0.00000 | 0.00000 | 0.00000 | 0.00000 | 0.00000 | 0.00000 | 0.00000 | 0.00000 | 0.00000 | 0.00000 | 0.00000 | 0.00000 | 0.00000 | 0.16667 |
| 12-105 | 0.00000 | 0.00000 | 0.00000 | 0.00000 | 0.00000 | 0.00000 | 0.00000 | 0.00000 | 0.00000 | 0.00000 | 0.00000 | 0.00000 | 0.00000 | 0.00000 | 0.00000 | 0.00000 | 0.00000 | 0.00000 | 0.00000 | 0.16667 |
| 12-106 | 0.00000 | 0.00000 | 0.16667 | 0.00000 | 0.00000 | 0.00000 | 0.00000 | 0.16667 | 0.00000 | 0.00000 | 0.00000 | 0.00000 | 0.00000 | 0.00000 | 0.00000 | 0.00000 | 0.00000 | 0.00000 | 0.00000 | 0.00000 |
| 14-001 | 0.00000 | 0.10000 | 0.16667 | 0.25000 | 0.08333 | 0.16667 | 0.00000 | 0.16667 | 0.08333 | 0.00000 | 0.00000 | 0.00000 | 0.00000 | 0.00000 | 0.00000 | 0.00000 | 0.08333 | 0.00000 | 0.12500 | 0.00000 |
| 14-002 | 0.00000 | 0.00000 | 0.00000 | 0.08333 | 0.00000 | 0.00000 | 0.00000 | 0.00000 | 0.00000 | 0.00000 | 0.00000 | 0.00000 | 0.00000 | 0.00000 | 0.00000 | 0.00000 | 0.00000 | 0.00000 | 0.00000 | 0.00000 |
| 14-003 | 0.00000 | 0.00000 | 0.16667 | 0.08333 | 0.25000 | 0.16667 | 0.08333 | 0.00000 | 0.00000 | 0.12500 | 0.00000 | 0.00000 | 0.10000 | 0.09091 | 0.00000 | 0.00000 | 0.08333 | 0.00000 | 0.12500 | 0.00000 |
| 14-004 | 0.00000 | 0.00000 | 0.16667 | 0.08333 | 0.08333 | 0.00000 | 0.00000 | 0.00000 | 0.00000 | 0.00000 | 0.00000 | 0.00000 | 0.00000 | 0.09091 | 0.00000 | 0.00000 | 0.00000 | 0.11111 | 0.12500 | 0.00000 |
| 14-005 | 0.00000 | 0.20000 | 0.16667 | 0.08333 | 0.25000 | 0.00000 | 0.08333 | 0.00000 | 0.00000 | 0.37500 | 0.08333 | 0.00000 | 0.10000 | 0.09091 | 0.50000 | 0.10000 | 0.08333 | 0.11111 | 0.62500 | 0.16667 |
| 14-006 | 0.71429 | 0.00000 | 0.00000 | 0.08333 | 0.08333 | 0.00000 | 0.00000 | 0.00000 | 0.00000 | 0.12500 | 0.00000 | 0.00000 | 0.10000 | 0.00000 | 0.00000 | 0.00000 | 0.25000 | 0.11111 | 0.00000 | 0.00000 |
| 14-007 | 0.00000 | 0.00000 | 0.08333 | 0.00000 | 0.16667 | 0.00000 | 0.00000 | 0.00000 | 0.00000 | 0.00000 | 0.16667 | 0.00000 | 0.00000 | 0.00000 | 0.00000 | 0.00000 | 0.16667 | 0.00000 | 0.00000 | 0.00000 |
| 14-008 | 0.00000 | 0.00000 | 0.16667 | 0.08333 | 0.16667 | 0.16667 | 0.00000 | 0.00000 | 0.00000 | 0.25000 | 0.00000 | 0.00000 | 0.00000 | 0.00000 | 0.00000 | 0.00000 | 0.00000 | 0.00000 | 0.00000 | 0.00000 |
| 14-009 | 0.00000 | 0.00000 | 0.00000 | 0.16667 | 0.00000 | 0.00000 | 0.00000 | 0.16667 | 0.08333 | 0.12500 | 0.00000 | 0.00000 | 0.00000 | 0.00000 | 0.00000 | 0.00000 | 0.00000 | 0.00000 | 0.00000 | 0.00000 |
| 14-010 | 0.00000 | 0.10000 | 0.16667 | 0.16667 | 0.08333 | 0.33333 | 0.08333 | 0.00000 | 0.00000 | 0.50000 | 0.00000 | 0.00000 | 0.00000 | 0.00000 | 0.00000 | 0.00000 | 0.08333 | 0.00000 | 0.00000 | 0.00000 |
| 14-011 | 0.00000 | 0.00000 | 0.25000 | 0.33333 | 0.33333 | 0.00000 | 0.16667 | 0.33333 | 0.16667 | 0.00000 | 0.16667 | 0.00000 | 0.10000 | 0.45455 | 0.00000 | 0.00000 | 0.33333 | 0.00000 | 0.00000 | 0.00000 |
| 14-012 | 0.00000 | 0.00000 | 0.00000 | 0.00000 | 0.08333 | 0.00000 | 0.00000 | 0.00000 | 0.00000 | 0.00000 | 0.00000 | 0.00000 | 0.00000 | 0.00000 | 0.00000 | 0.00000 | 0.00000 | 0.00000 | 0.00000 | 0.00000 |
| 14-013 | 0.00000 | 0.20000 | 0.08333 | 0.00000 | 0.08333 | 0.00000 | 0.16667 | 0.00000 | 0.25000 | 0.25000 | 0.16667 | 0.20000 | 0.00000 | 0.27273 | 0.10000 | 0.00000 | 0.08333 | 0.00000 | 0.25000 | 0.00000 |
| 14-014 | 0.14286 | 0.10000 | 0.16667 | 0.08333 | 0.08333 | 0.00000 | 0.08333 | 0.16667 | 0.00000 | 0.25000 | 0.16667 | 0.10000 | 0.00000 | 0.00000 | 0.00000 | 0.00000 | 0.08333 | 0.00000 | 0.25000 | 0.00000 |
| 14-015 | 0.00000 | 0.30000 | 0.16667 | 0.08333 | 0.16667 | 0.00000 | 0.08333 | 0.00000 | 0.08333 | 0.00000 | 0.08333 | 0.20000 | 0.10000 | 0.18182 | 0.00000 | 0.20000 | 0.08333 | 0.11111 | 0.25000 | 0.16667 |
| 14-016 | 0.00000 | 0.00000 | 0.08333 | 0.08333 | 0.08333 | 0.00000 | 0.00000 | 0.00000 | 0.25000 | 0.00000 | 0.08333 | 0.00000 | 0.00000 | 0.00000 | 0.00000 | 0.00000 | 0.16667 | 0.00000 | 0.00000 | 0.00000 |
| 14-017 | 0.00000 | 0.20000 | 0.50000 | 0.25000 | 0.08333 | 0.16667 | 0.00000 | 0.00000 | 0.08333 | 0.00000 | 0.00000 | 0.40000 | 0.10000 | 0.27273 | 0.00000 | 0.10000 | 0.16667 | 0.11111 | 0.25000 | 0.00000 |
| 14-018 | 0.00000 | 0.00000 | 0.41667 | 0.08333 | 0.08333 | 0.00000 | 0.00000 | 0.00000 | 0.16667 | 0.12500 | 0.00000 | 0.20000 | 0.00000 | 0.18182 | 0.00000 | 0.10000 | 0.08333 | 0.11111 | 0.12500 | 0.00000 |
| 14-019 | 0.00000 | 0.20000 | 0.08333 | 0.08333 | 0.08333 | 0.00000 | 0.00000 | 0.00000 | 0.00000 | 0.00000 | 0.00000 | 0.00000 | 0.00000 | 0.00000 | 0.00000 | 0.00000 | 0.08333 | 0.00000 | 0.00000 | 0.00000 |
| 14-020 | 0.00000 | 0.20000 | 0.16667 | 0.16667 | 0.16667 | 0.16667 | 0.16667 | 0.16667 | 0.25000 | 0.12500 | 0.16667 | 0.30000 | 0.30000 | 0.36364 | 0.30000 | 0.30000 | 0.33333 | 0.00000 | 0.62500 | 0.16667 |
| 14-021 | 0.00000 | 0.00000 | 0.00000 | 0.08333 | 0.00000 | 0.00000 | 0.00000 | 0.00000 | 0.08333 | 0.00000 | 0.00000 | 0.00000 | 0.00000 | 0.00000 | 0.00000 | 0.00000 | 0.00000 | 0.00000 | 0.00000 | 0.00000 |
| 14-022 | 0.00000 | 0.00000 | 0.00000 | 0.08333 | 0.08333 | 0.00000 | 0.00000 | 0.00000 | 0.00000 | 0.12500 | 0.08333 | 0.00000 | 0.10000 | 0.00000 | 0.00000 | 0.00000 | 0.00000 | 0.00000 | 0.00000 | 0.00000 |
| 14-023 | 0.00000 | 0.00000 | 0.08333 | 0.33333 | 0.16667 | 0.16667 | 0.08333 | 0.00000 | 0.00000 | 0.25000 | 0.08333 | 0.00000 | 0.00000 | 0.09091 | 0.40000 | 0.20000 | 0.08333 | 0.22222 | 0.50000 | 0.16667 |
| 14-024 | 0.00000 | 0.40000 | 0.00000 | 0.25000 | 0.25000 | 0.00000 | 0.08333 | 0.00000 | 0.00000 | 0.25000 | 0.00000 | 0.00000 | 0.00000 | 0.00000 | 0.10000 | 0.00000 | 0.08333 | 0.11111 | 0.75000 | 0.33333 |
| 14-025 | 0.00000 | 0.00000 | 0.16667 | 0.08333 | 0.08333 | 0.00000 | 0.00000 | 0.00000 | 0.00000 | 0.00000 | 0.00000 | 0.00000 | 0.10000 | 0.00000 | 0.00000 | 0.00000 | 0.08333 | 0.00000 | 0.25000 | 0.00000 |
| 14-026 | 0.00000 | 0.20000 | 0.00000 | 0.00000 | 0.08333 | 0.00000 | 0.00000 | 0.00000 | 0.00000 | 0.12500 | 0.08333 | 0.00000 | 0.00000 | 0.00000 | 0.00000 | 0.00000 | 0.00000 | 0.00000 | 0.00000 | 0.00000 |
| 14-027 | 0.00000 | 0.30000 | 0.08333 | 0.25000 | 0.08333 | 0.00000 | 0.00000 | 0.00000 | 0.08333 | 0.00000 | 0.00000 | 0.00000 | 0.10000 | 0.00000 | 0.00000 | 0.40000 | 0.08333 | 0.11111 | 0.25000 | 0.16667 |
| 14-028 | 0.00000 | 0.00000 | 0.08333 | 0.16667 | 0.16667 | 0.00000 | 0.00000 | 0.00000 | 0.08333 | 0.00000 | 0.00000 | 0.00000 | 0.10000 | 0.00000 | 0.10000 | 0.00000 | 0.00000 | 0.11111 | 0.00000 | 0.00000 |
| 14-029 | 0.00000 | 0.00000 | 0.08333 | 0.25000 | 0.08333 | 0.00000 | 0.00000 | 0.00000 | 0.00000 | 0.12500 | 0.00000 | 0.00000 | 0.10000 | 0.00000 | 0.00000 | 0.00000 | 0.00000 | 0.00000 | 0.00000 | 0.00000 |
| 14-030 | 0.00000 | 0.30000 | 0.25000 | 0.33333 | 0.08333 | 0.16667 | 0.08333 | 0.16667 | 0.08333 | 0.37500 | 0.00000 | 0.10000 | 0.10000 | 0.18182 | 0.40000 | 0.10000 | 0.08333 | 0.11111 | 0.00000 | 0.16667 |
| 14-031 | 0.00000 | 0.00000 | 0.08333 | 0.08333 | 0.08333 | 0.00000 | 0.00000 | 0.00000 | 0.00000 | 0.00000 | 0.00000 | 0.00000 | 0.10000 | 0.00000 | 0.00000 | 0.00000 | 0.16667 | 0.00000 | 0.00000 | 0.00000 |
| 14-032 | 0.00000 | 0.00000 | 0.00000 | 0.16667 | 0.16667 | 0.00000 | 0.00000 | 0.00000 | 0.00000 | 0.00000 | 0.00000 | 0.00000 | 0.10000 | 0.00000 | 0.00000 | 0.00000 | 0.16667 | 0.00000 | 0.00000 | 0.00000 |
| 14-033 | 0.00000 | 0.50000 | 0.50000 | 0.16667 | 0.25000 | 0.16667 | 0.41667 | 0.16667 | 0.08333 | 0.12500 | 0.33333 | 0.50000 | 0.00000 | 0.27273 | 0.10000 | 0.20000 | 0.41667 | 0.00000 | 0.12500 | 0.00000 |
| 14-034 | 0.00000 | 0.30000 | 0.16667 | 0.33333 | 0.16667 | 0.16667 | 0.41667 | 0.50000 | 0.16667 | 0.12500 | 0.33333 | 0.30000 | 0.10000 | 0.27273 | 0.10000 | 0.10000 | 0.41667 | 0.11111 | 0.25000 | 0.00000 |
| 14-035 | 0.00000 | 0.00000 | 0.00000 | 0.00000 | 0.00000 | 0.00000 | 0.00000 | 0.00000 | 0.16667 | 0.00000 | 0.00000 | 0.00000 | 0.00000 | 0.00000 | 0.00000 | 0.00000 | 0.08333 | 0.00000 | 0.12500 | 0.00000 |
| 14-036 | 0.00000 | 0.10000 | 0.08333 | 0.16667 | 0.00000 | 0.00000 | 0.00000 | 0.00000 | 0.16667 | 0.00000 | 0.08333 | 0.30000 | 0.00000 | 0.00000 | 0.10000 | 0.00000 | 0.08333 | 0.00000 | 0.12500 | 0.00000 |
| 14-037 | 0.00000 | 0.00000 | 0.00000 | 0.08333 | 0.16667 | 0.00000 | 0.00000 | 0.00000 | 0.00000 | 0.00000 | 0.00000 | 0.00000 | 0.00000 | 0.00000 | 0.00000 | 0.00000 | 0.08333 | 0.00000 | 0.00000 | 0.00000 |
| 14-038 | 0.28571 | 0.20000 | 0.16667 | 0.25000 | 0.33333 | 0.16667 | 0.08333 | 0.33333 | 0.33333 | 0.37500 | 0.25000 | 0.30000 | 0.40000 | 0.18182 | 0.00000 | 0.20000 | 0.50000 | 0.11111 | 0.12500 | 0.16667 |
| 14-039 | 0.00000 | 0.30000 | 0.33333 | 0.16667 | 0.00000 | 0.16667 | 0.08333 | 0.00000 | 0.08333 | 0.00000 | 0.00000 | 0.20000 | 0.30000 | 0.09091 | 0.00000 | 0.00000 | 0.25000 | 0.00000 | 0.12500 | 0.16667 |
| 14-040 | 0.00000 | 0.10000 | 0.00000 | 0.08333 | 0.08333 | 0.00000 | 0.00000 | 0.00000 | 0.00000 | 0.00000 | 0.00000 | 0.10000 | 0.00000 | 0.00000 | 0.00000 | 0.00000 | 0.00000 | 0.00000 | 0.00000 | 0.16667 |
| 14-041 | 0.00000 | 0.20000 | 0.25000 | 0.25000 | 0.08333 | 0.33333 | 0.00000 | 0.00000 | 0.00000 | 0.50000 | 0.00000 | 0.30000 | 0.00000 | 0.36364 | 0.00000 | 0.20000 | 0.41667 | 0.00000 | 0.12500 | 0.33333 |
| 14-042 | 0.14286 | 0.00000 | 0.00000 | 0.08333 | 0.00000 | 0.00000 | 0.00000 | 0.00000 | 0.00000 | 0.00000 | 0.00000 | 0.10000 | 0.00000 | 0.00000 | 0.00000 | 0.00000 | 0.00000 | 0.00000 | 0.00000 | 0.50000 |
| 14-043 | 0.14286 | 0.10000 | 0.16667 | 0.08333 | 0.00000 | 0.00000 | 0.33333 | 0.33333 | 0.33333 | 0.00000 | 0.25000 | 0.10000 | 0.00000 | 0.00000 | 0.00000 | 0.00000 | 0.08333 | 0.00000 | 0.00000 | 0.33333 |
| 14-044 | 0.00000 | 0.00000 | 0.16667 | 0.08333 | 0.16667 | 0.33333 | 0.25000 | 0.16667 | 0.00000 | 0.00000 | 0.08333 | 0.00000 | 0.10000 | 0.00000 | 0.60000 | 0.30000 | 0.08333 | 0.33333 | 0.50000 | 0.83333 |
| 14-045 | 0.00000 | 0.10000 | 0.08333 | 0.08333 | 0.08333 | 0.33333 | 0.25000 | 0.16667 | 0.00000 | 0.12500 | 0.08333 | 0.10000 | 0.10000 | 0.18182 | 0.60000 | 0.20000 | 0.00000 | 0.44444 | 0.37500 | 0.66667 |
| 14-046 | 0.00000 | 0.10000 | 0.16667 | 0.08333 | 0.00000 | 0.16667 | 0.00000 | 0.00000 | 0.00000 | 0.00000 | 0.00000 | 0.40000 | 0.00000 | 0.00000 | 0.00000 | 0.10000 | 0.00000 | 0.00000 | 0.00000 | 0.66667 |
| 14-047 | 0.14286 | 0.10000 | 0.08333 | 0.16667 | 0.16667 | 0.00000 | 0.08333 | 0.00000 | 0.25000 | 0.00000 | 0.00000 | 0.20000 | 0.10000 | 0.00000 | 0.00000 | 0.00000 | 0.00000 | 0.00000 | 0.12500 | 0.83333 |
| 14-048 | 0.00000 | 0.20000 | 0.08333 | 0.08333 | 0.16667 | 0.16667 | 0.00000 | 0.00000 | 0.00000 | 0.00000 | 0.16667 | 0.10000 | 0.10000 | 0.00000 | 0.20000 | 0.00000 | 0.00000 | 0.11111 | 0.25000 | 0.00000 |
| 14-049 | 0.14286 | 0.10000 | 0.50000 | 0.08333 | 0.16667 | 0.16667 | 0.16667 | 0.00000 | 0.16667 | 0.12500 | 0.41667 | 0.10000 | 0.50000 | 0.09091 | 0.10000 | 0.20000 | 0.00000 | 0.22222 | 0.12500 | 0.16667 |
| 14-050 | 0.42857 | 0.20000 | 0.50000 | 0.16667 | 0.16667 | 0.33333 | 0.33333 | 0.00000 | 0.25000 | 0.12500 | 0.08333 | 0.50000 | 0.20000 | 0.45455 | 0.00000 | 0.20000 | 0.33333 | 0.22222 | 0.37500 | 0.33333 |
| 14-051 | 0.28571 | 0.10000 | 0.16667 | 0.16667 | 0.00000 | 0.16667 | 0.16667 | 0.33333 | 0.25000 | 0.00000 | 0.33333 | 0.30000 | 0.20000 | 0.27273 | 0.10000 | 0.10000 | 0.41667 | 0.11111 | 0.12500 | 0.83333 |
| 14-052 | 0.00000 | 0.10000 | 0.00000 | 0.25000 | 0.08333 | 0.16667 | 0.25000 | 0.16667 | 0.41667 | 0.00000 | 0.25000 | 0.20000 | 0.10000 | 0.09091 | 0.00000 | 0.10000 | 0.25000 | 0.00000 | 0.12500 | 0.83333 |
| 14-053 | 0.00000 | 0.10000 | 0.00000 | 0.00000 | 0.00000 | 0.16667 | 0.08333 | 0.33333 | 0.00000 | 0.12500 | 0.00000 | 0.00000 | 0.00000 | 0.09091 | 0.00000 | 0.10000 | 0.08333 | 0.22222 | 0.00000 | 0.33333 |
| 14-054 | 0.00000 | 0.00000 | 0.00000 | 0.08333 | 0.00000 | 0.00000 | 0.08333 | 0.00000 | 0.00000 | 0.00000 | 0.00000 | 0.10000 | 0.10000 | 0.00000 | 0.00000 | 0.00000 | 0.08333 | 0.00000 | 0.00000 | 0.00000 |
| 14-055 | 0.00000 | 0.00000 | 0.50000 | 0.25000 | 0.50000 | 0.00000 | 0.08333 | 0.00000 | 0.00000 | 0.00000 | 0.00000 | 0.10000 | 0.30000 | 0.09091 | 0.00000 | 0.30000 | 0.16667 | 0.33333 | 0.00000 | 0.00000 |
| 14-056 | 0.00000 | 0.00000 | 0.50000 | 0.25000 | 0.41667 | 0.16667 | 0.08333 | 0.00000 | 0.08333 | 0.00000 | 0.00000 | 0.10000 | 0.00000 | 0.00000 | 0.00000 | 0.00000 | 0.16667 | 0.11111 | 0.00000 | 0.50000 |
| 14-057 | 0.00000 | 0.10000 | 0.00000 | 0.08333 | 0.00000 | 0.33333 | 0.08333 | 0.00000 | 0.08333 | 0.12500 | 0.00000 | 0.00000 | 0.00000 | 0.00000 | 0.00000 | 0.20000 | 0.16667 | 0.11111 | 0.12500 | 0.33333 |
| 14-058 | 0.00000 | 0.10000 | 0.08333 | 0.08333 | 0.25000 | 0.16667 | 0.25000 | 0.33333 | 0.66667 | 0.12500 | 0.33333 | 0.20000 | 0.10000 | 0.00000 | 0.10000 | 0.00000 | 0.16667 | 0.11111 | 0.12500 | 0.50000 |
| 14-059 | 0.00000 | 0.20000 | 0.08333 | 0.25000 | 0.08333 | 0.33333 | 0.25000 | 0.16667 | 0.00000 | 0.00000 | 0.08333 | 0.10000 | 0.10000 | 0.09091 | 0.20000 | 0.10000 | 0.08333 | 0.22222 | 0.37500 | 0.66667 |
| 14-060 | 0.00000 | 0.00000 | 0.16667 | 0.16667 | 0.25000 | 0.16667 | 0.08333 | 0.33333 | 0.25000 | 0.12500 | 0.08333 | 0.00000 | 0.10000 | 0.09091 | 0.10000 | 0.20000 | 0.08333 | 0.00000 | 0.00000 | 0.00000 |
| 14-061 | 0.00000 | 0.00000 | 0.00000 | 0.08333 | 0.16667 | 0.00000 | 0.08333 | 0.33333 | 0.08333 | 0.00000 | 0.08333 | 0.10000 | 0.00000 | 0.00000 | 0.00000 | 0.10000 | 0.00000 | 0.00000 | 0.00000 | 0.00000 |
| 14-062 | 0.28571 | 0.00000 | 0.00000 | 0.08333 | 0.16667 | 0.00000 | 0.08333 | 0.00000 | 0.00000 | 0.12500 | 0.08333 | 0.00000 | 0.00000 | 0.09091 | 0.00000 | 0.10000 | 0.16667 | 0.11111 | 0.37500 | 0.16667 |
| 14-063 | 0.00000 | 0.00000 | 0.00000 | 0.08333 | 0.08333 | 0.00000 | 0.00000 | 0.16667 | 0.00000 | 0.00000 | 0.00000 | 0.20000 | 0.00000 | 0.00000 | 0.00000 | 0.00000 | 0.08333 | 0.00000 | 0.12500 | 0.00000 |
| 14-064 | 0.14286 | 0.00000 | 0.16667 | 0.00000 | 0.00000 | 0.00000 | 0.08333 | 0.33333 | 0.33333 | 0.00000 | 0.16667 | 0.00000 | 0.00000 | 0.09091 | 0.00000 | 0.00000 | 0.00000 | 0.00000 | 0.25000 | 0.16667 |
| 14-065 | 0.00000 | 0.10000 | 0.08333 | 0.00000 | 0.08333 | 0.00000 | 0.16667 | 0.00000 | 0.33333 | 0.00000 | 0.08333 | 0.10000 | 0.00000 | 0.00000 | 0.10000 | 0.30000 | 0.00000 | 0.22222 | 0.12500 | 0.66667 |
| 14-066 | 0.14286 | 0.00000 | 0.16667 | 0.08333 | 0.08333 | 0.16667 | 0.00000 | 0.16667 | 0.08333 | 0.12500 | 0.00000 | 0.20000 | 0.00000 | 0.00000 | 0.20000 | 0.20000 | 0.16667 | 0.11111 | 0.25000 | 0.50000 |
| 14-067 | 0.00000 | 0.20000 | 0.08333 | 0.16667 | 0.08333 | 0.00000 | 0.08333 | 0.16667 | 0.00000 | 0.12500 | 0.00000 | 0.10000 | 0.00000 | 0.00000 | 0.00000 | 0.10000 | 0.00000 | 0.00000 | 0.12500 | 0.33333 |
| 14-068 | 0.00000 | 0.00000 | 0.00000 | 0.16667 | 0.00000 | 0.00000 | 0.00000 | 0.00000 | 0.00000 | 0.00000 | 0.00000 | 0.10000 | 0.00000 | 0.00000 | 0.10000 | 0.00000 | 0.08333 | 0.00000 | 0.00000 | 0.33333 |
| 14-069 | 0.00000 | 0.10000 | 0.08333 | 0.25000 | 0.25000 | 0.33333 | 0.16667 | 0.00000 | 0.25000 | 0.00000 | 0.16667 | 0.20000 | 0.00000 | 0.00000 | 0.00000 | 0.10000 | 0.25000 | 0.00000 | 0.00000 | 0.50000 |
| 14-070 | 0.14286 | 0.10000 | 0.08333 | 0.08333 | 0.25000 | 0.33333 | 0.25000 | 0.00000 | 0.25000 | 0.25000 | 0.08333 | 0.20000 | 0.00000 | 0.00000 | 0.00000 | 0.10000 | 0.16667 | 0.00000 | 0.00000 | 0.00000 |
| 14-071 | 0.00000 | 0.10000 | 0.08333 | 0.08333 | 0.16667 | 0.16667 | 0.16667 | 0.16667 | 0.08333 | 0.25000 | 0.16667 | 0.00000 | 0.10000 | 0.00000 | 0.80000 | 0.30000 | 0.16667 | 0.22222 | 0.62500 | 0.16667 |
| 14-072 | 0.00000 | 0.10000 | 0.00000 | 0.00000 | 0.00000 | 0.16667 | 0.00000 | 0.00000 | 0.00000 | 0.00000 | 0.08333 | 0.00000 | 0.00000 | 0.00000 | 0.10000 | 0.10000 | 0.00000 | 0.00000 | 0.25000 | 0.00000 |
| 14-073 | 0.00000 | 0.00000 | 0.00000 | 0.08333 | 0.16667 | 0.16667 | 0.08333 | 0.00000 | 0.00000 | 0.00000 | 0.00000 | 0.00000 | 0.00000 | 0.09091 | 0.00000 | 0.00000 | 0.08333 | 0.00000 | 0.00000 | 0.00000 |
| 14-074 | 0.14286 | 0.30000 | 0.16667 | 0.25000 | 0.33333 | 0.33333 | 0.41667 | 0.16667 | 0.16667 | 0.25000 | 0.25000 | 0.10000 | 0.20000 | 0.09091 | 0.90000 | 0.40000 | 0.08333 | 0.33333 | 0.62500 | 0.66667 |
| 14-075 | 0.00000 | 0.20000 | 0.00000 | 0.08333 | 0.00000 | 0.00000 | 0.00000 | 0.00000 | 0.00000 | 0.00000 | 0.00000 | 0.00000 | 0.00000 | 0.00000 | 0.00000 | 0.00000 | 0.00000 | 0.00000 | 0.00000 | 0.16667 |
| 14-076 | 0.00000 | 0.00000 | 0.00000 | 0.08333 | 0.00000 | 0.00000 | 0.00000 | 0.00000 | 0.08333 | 0.00000 | 0.00000 | 0.20000 | 0.00000 | 0.00000 | 0.00000 | 0.00000 | 0.16667 | 0.00000 | 0.00000 | 0.16667 |
| 14-077 | 0.00000 | 0.00000 | 0.00000 | 0.08333 | 0.08333 | 0.00000 | 0.00000 | 0.00000 | 0.00000 | 0.00000 | 0.00000 | 0.00000 | 0.00000 | 0.00000 | 0.00000 | 0.00000 | 0.16667 | 0.11111 | 0.00000 | 0.00000 |
| 14-078 | 0.00000 | 0.20000 | 0.08333 | 0.16667 | 0.08333 | 0.33333 | 0.33333 | 0.16667 | 0.16667 | 0.00000 | 0.16667 | 0.10000 | 0.00000 | 0.00000 | 0.20000 | 0.10000 | 0.16667 | 0.22222 | 0.25000 | 0.50000 |
| 14-079 | 0.00000 | 0.10000 | 0.00000 | 0.16667 | 0.00000 | 0.16667 | 0.08333 | 0.16667 | 0.00000 | 0.00000 | 0.00000 | 0.00000 | 0.00000 | 0.00000 | 0.00000 | 0.00000 | 0.00000 | 0.22222 | 0.00000 | 0.16667 |
| 14-080 | 0.00000 | 0.00000 | 0.00000 | 0.08333 | 0.00000 | 0.00000 | 0.00000 | 0.00000 | 0.00000 | 0.00000 | 0.00000 | 0.00000 | 0.00000 | 0.00000 | 0.00000 | 0.00000 | 0.00000 | 0.00000 | 0.00000 | 0.00000 |
| 14-081 | 0.00000 | 0.00000 | 0.08333 | 0.00000 | 0.00000 | 0.16667 | 0.08333 | 0.00000 | 0.08333 | 0.00000 | 0.00000 | 0.00000 | 0.00000 | 0.00000 | 0.00000 | 0.00000 | 0.00000 | 0.00000 | 0.25000 | 0.16667 |
| 14-082 | 0.00000 | 0.00000 | 0.00000 | 0.25000 | 0.08333 | 0.16667 | 0.00000 | 0.00000 | 0.08333 | 0.12500 | 0.00000 | 0.00000 | 0.00000 | 0.00000 | 0.00000 | 0.10000 | 0.00000 | 0.00000 | 0.00000 | 0.16667 |
| 14-083 | 0.00000 | 0.10000 | 0.08333 | 0.08333 | 0.00000 | 0.33333 | 0.08333 | 0.00000 | 0.00000 | 0.37500 | 0.00000 | 0.10000 | 0.10000 | 0.45455 | 0.10000 | 0.20000 | 0.16667 | 0.00000 | 0.00000 | 0.16667 |
| 14-084 | 0.00000 | 0.10000 | 0.00000 | 0.08333 | 0.08333 | 0.00000 | 0.16667 | 0.00000 | 0.08333 | 0.00000 | 0.00000 | 0.10000 | 0.00000 | 0.00000 | 0.20000 | 0.00000 | 0.08333 | 0.11111 | 0.00000 | 0.00000 |
| 14-085 | 0.00000 | 0.00000 | 0.00000 | 0.00000 | 0.25000 | 0.00000 | 0.08333 | 0.00000 | 0.00000 | 0.00000 | 0.00000 | 0.00000 | 0.00000 | 0.00000 | 0.00000 | 0.10000 | 0.00000 | 0.11111 | 0.00000 | 0.16667 |
| 14-086 | 0.00000 | 0.40000 | 0.25000 | 0.16667 | 0.16667 | 0.16667 | 0.08333 | 0.33333 | 0.08333 | 0.12500 | 0.08333 | 0.00000 | 0.20000 | 0.18182 | 0.60000 | 0.00000 | 0.08333 | 0.11111 | 0.37500 | 0.50000 |
| 14-087 | 0.00000 | 0.00000 | 0.00000 | 0.16667 | 0.16667 | 0.16667 | 0.33333 | 0.00000 | 0.25000 | 0.00000 | 0.00000 | 0.00000 | 0.00000 | 0.00000 | 0.40000 | 0.10000 | 0.00000 | 0.00000 | 0.37500 | 0.33333 |
| 14-088 | 0.00000 | 0.00000 | 0.00000 | 0.00000 | 0.00000 | 0.16667 | 0.00000 | 0.00000 | 0.08333 | 0.00000 | 0.00000 | 0.10000 | 0.00000 | 0.00000 | 0.00000 | 0.00000 | 0.08333 | 0.00000 | 0.12500 | 0.16667 |
| 14-089 | 0.00000 | 0.10000 | 0.00000 | 0.08333 | 0.08333 | 0.00000 | 0.00000 | 0.00000 | 0.00000 | 0.00000 | 0.00000 | 0.00000 | 0.00000 | 0.00000 | 0.00000 | 0.10000 | 0.00000 | 0.11111 | 0.12500 | 0.50000 |
| 14-090 | 0.00000 | 0.00000 | 0.00000 | 0.08333 | 0.08333 | 0.16667 | 0.00000 | 0.00000 | 0.00000 | 0.00000 | 0.00000 | 0.20000 | 0.00000 | 0.00000 | 0.00000 | 0.10000 | 0.16667 | 0.00000 | 0.25000 | 0.16667 |
| 14-091 | 0.00000 | 0.00000 | 0.00000 | 0.00000 | 0.00000 | 0.33333 | 0.00000 | 0.00000 | 0.16667 | 0.00000 | 0.00000 | 0.00000 | 0.00000 | 0.00000 | 0.00000 | 0.00000 | 0.08333 | 0.00000 | 0.12500 | 0.33333 |
| 14-092 | 0.00000 | 0.30000 | 0.08333 | 0.08333 | 0.08333 | 0.00000 | 0.00000 | 0.00000 | 0.08333 | 0.00000 | 0.00000 | 0.00000 | 0.00000 | 0.00000 | 0.00000 | 0.00000 | 0.00000 | 0.11111 | 0.00000 | 0.16667 |
| 14-093 | 0.00000 | 0.00000 | 0.08333 | 0.00000 | 0.08333 | 0.00000 | 0.25000 | 0.00000 | 0.16667 | 0.00000 | 0.08333 | 0.10000 | 0.00000 | 0.00000 | 0.20000 | 0.30000 | 0.16667 | 0.22222 | 0.62500 | 0.33333 |
| 14-094 | 0.57143 | 0.00000 | 0.08333 | 0.08333 | 0.00000 | 0.00000 | 0.08333 | 0.16667 | 0.16667 | 0.00000 | 0.16667 | 0.00000 | 0.00000 | 0.00000 | 0.00000 | 0.10000 | 0.00000 | 0.00000 | 0.00000 | 0.50000 |
| 14-095 | 0.00000 | 0.00000 | 0.00000 | 0.08333 | 0.00000 | 0.16667 | 0.00000 | 0.00000 | 0.00000 | 0.00000 | 0.00000 | 0.20000 | 0.00000 | 0.00000 | 0.00000 | 0.30000 | 0.16667 | 0.00000 | 0.12500 | 0.33333 |
| 14-096 | 0.00000 | 0.30000 | 0.08333 | 0.08333 | 0.16667 | 0.50000 | 0.00000 | 0.00000 | 0.16667 | 0.00000 | 0.00000 | 0.30000 | 0.00000 | 0.00000 | 0.00000 | 0.30000 | 0.08333 | 0.11111 | 0.00000 | 0.66667 |
| 14-097 | 0.00000 | 0.10000 | 0.08333 | 0.00000 | 0.08333 | 0.16667 | 0.16667 | 0.00000 | 0.00000 | 0.00000 | 0.08333 | 0.20000 | 0.00000 | 0.00000 | 0.00000 | 0.00000 | 0.00000 | 0.11111 | 0.12500 | 0.16667 |
| 14-098 | 0.00000 | 0.00000 | 0.00000 | 0.08333 | 0.08333 | 0.33333 | 0.00000 | 0.00000 | 0.00000 | 0.00000 | 0.00000 | 0.00000 | 0.00000 | 0.00000 | 0.00000 | 0.00000 | 0.16667 | 0.00000 | 0.00000 | 0.00000 |
| 14-099 | 0.00000 | 0.00000 | 0.00000 | 0.08333 | 0.08333 | 0.00000 | 0.00000 | 0.00000 | 0.00000 | 0.00000 | 0.00000 | 0.10000 | 0.00000 | 0.00000 | 0.00000 | 0.10000 | 0.00000 | 0.00000 | 0.12500 | 0.00000 |
| 14-100 | 0.00000 | 0.40000 | 0.41667 | 0.16667 | 0.16667 | 0.00000 | 0.16667 | 0.00000 | 0.08333 | 0.00000 | 0.00000 | 0.00000 | 0.00000 | 0.00000 | 0.20000 | 0.10000 | 0.00000 | 0.44444 | 0.12500 | 0.33333 |
| 14-101 | 0.00000 | 0.00000 | 0.00000 | 0.00000 | 0.00000 | 0.00000 | 0.08333 | 0.00000 | 0.00000 | 0.00000 | 0.00000 | 0.00000 | 0.00000 | 0.00000 | 0.10000 | 0.00000 | 0.00000 | 0.00000 | 0.00000 | 0.16667 |
| 14-102 | 0.00000 | 0.00000 | 0.08333 | 0.00000 | 0.00000 | 0.00000 | 0.00000 | 0.00000 | 0.08333 | 0.00000 | 0.00000 | 0.00000 | 0.00000 | 0.09091 | 0.00000 | 0.00000 | 0.00000 | 0.00000 | 0.00000 | 0.16667 |
| 14-103 | 0.00000 | 0.10000 | 0.16667 | 0.08333 | 0.00000 | 0.50000 | 0.08333 | 0.00000 | 0.08333 | 0.00000 | 0.08333 | 0.10000 | 0.00000 | 0.00000 | 0.00000 | 0.00000 | 0.00000 | 0.11111 | 0.00000 | 0.00000 |
| 14-104 | 0.00000 | 0.20000 | 0.00000 | 0.00000 | 0.00000 | 0.16667 | 0.00000 | 0.00000 | 0.16667 | 0.00000 | 0.08333 | 0.10000 | 0.10000 | 0.18182 | 0.10000 | 0.10000 | 0.25000 | 0.22222 | 0.25000 | 0.50000 |
| 14-105 | 0.71429 | 0.30000 | 0.50000 | 0.25000 | 0.33333 | 0.33333 | 0.58333 | 0.33333 | 0.33333 | 0.12500 | 0.08333 | 0.00000 | 0.20000 | 0.45455 | 0.10000 | 0.40000 | 0.33333 | 0.55556 | 0.62500 | 0.50000 |
| 14-106 | 0.00000 | 0.10000 | 0.00000 | 0.08333 | 0.00000 | 0.16667 | 0.08333 | 0.00000 | 0.00000 | 0.00000 | 0.00000 | 0.00000 | 0.00000 | 0.00000 | 0.60000 | 0.30000 | 0.00000 | 0.00000 | 0.00000 | 0.16667 |
| 14-107 | 0.00000 | 0.00000 | 0.16667 | 0.08333 | 0.00000 | 0.16667 | 0.08333 | 0.00000 | 0.08333 | 0.00000 | 0.00000 | 0.10000 | 0.00000 | 0.09091 | 0.20000 | 0.20000 | 0.08333 | 0.00000 | 0.37500 | 0.16667 |
| 14-108 | 0.00000 | 0.20000 | 0.00000 | 0.08333 | 0.00000 | 0.33333 | 0.08333 | 0.16667 | 0.16667 | 0.00000 | 0.00000 | 0.00000 | 0.00000 | 0.00000 | 0.00000 | 0.00000 | 0.16667 | 0.00000 | 0.00000 | 0.16667 |
| 14-109 | 0.00000 | 0.20000 | 0.00000 | 0.00000 | 0.00000 | 0.33333 | 0.08333 | 0.00000 | 0.25000 | 0.00000 | 0.00000 | 0.00000 | 0.00000 | 0.00000 | 0.00000 | 0.00000 | 0.16667 | 0.00000 | 0.00000 | 0.00000 |
| 14-110 | 0.00000 | 0.00000 | 0.00000 | 0.00000 | 0.00000 | 0.00000 | 0.00000 | 0.00000 | 0.08333 | 0.00000 | 0.00000 | 0.00000 | 0.00000 | 0.00000 | 0.00000 | 0.00000 | 0.00000 | 0.00000 | 0.00000 | 0.00000 |
| 14-111 | 0.00000 | 0.00000 | 0.00000 | 0.00000 | 0.00000 | 0.00000 | 0.00000 | 0.00000 | 0.00000 | 0.00000 | 0.00000 | 0.10000 | 0.00000 | 0.00000 | 0.00000 | 0.20000 | 0.00000 | 0.00000 | 0.00000 | 0.16667 |
| 14-112 | 0.00000 | 0.00000 | 0.00000 | 0.00000 | 0.00000 | 0.00000 | 0.00000 | 0.33333 | 0.00000 | 0.00000 | 0.00000 | 0.10000 | 0.00000 | 0.09091 | 0.00000 | 0.20000 | 0.00000 | 0.22222 | 0.50000 | 0.33333 |
| 14-113 | 0.00000 | 0.10000 | 0.41667 | 0.16667 | 0.16667 | 0.16667 | 0.08333 | 0.00000 | 0.00000 | 0.00000 | 0.33333 | 0.00000 | 0.00000 | 0.00000 | 0.00000 | 0.00000 | 0.00000 | 0.00000 | 0.12500 | 0.00000 |
| 14-114 | 0.00000 | 0.00000 | 0.00000 | 0.08333 | 0.00000 | 0.33333 | 0.08333 | 0.00000 | 0.08333 | 0.00000 | 0.08333 | 0.10000 | 0.10000 | 0.18182 | 0.00000 | 0.20000 | 0.00000 | 0.33333 | 0.12500 | 0.50000 |
| 14-115 | 0.00000 | 0.00000 | 0.00000 | 0.00000 | 0.08333 | 0.33333 | 0.08333 | 0.00000 | 0.08333 | 0.00000 | 0.08333 | 0.00000 | 0.00000 | 0.00000 | 0.00000 | 0.20000 | 0.00000 | 0.11111 | 0.00000 | 0.00000 |
| 14-116 | 0.00000 | 0.10000 | 0.00000 | 0.00000 | 0.00000 | 0.00000 | 0.00000 | 0.00000 | 0.00000 | 0.00000 | 0.00000 | 0.00000 | 0.00000 | 0.00000 | 0.00000 | 0.00000 | 0.00000 | 0.00000 | 0.12500 | 0.33333 |
| 14-117 | 0.00000 | 0.10000 | 0.08333 | 0.00000 | 0.00000 | 0.16667 | 0.00000 | 0.00000 | 0.00000 | 0.00000 | 0.00000 | 0.00000 | 0.00000 | 0.00000 | 0.00000 | 0.10000 | 0.00000 | 0.00000 | 0.00000 | 0.33333 |
| 14-118 | 0.00000 | 0.20000 | 0.25000 | 0.08333 | 0.00000 | 0.16667 | 0.50000 | 0.50000 | 0.16667 | 0.12500 | 0.33333 | 0.00000 | 0.00000 | 0.09091 | 0.30000 | 0.20000 | 0.08333 | 0.77778 | 0.75000 | 0.16667 |
| 14-119 | 0.14286 | 0.20000 | 0.16667 | 0.08333 | 0.00000 | 0.16667 | 0.00000 | 0.00000 | 0.08333 | 0.12500 | 0.00000 | 0.00000 | 0.00000 | 0.00000 | 0.00000 | 0.00000 | 0.00000 | 0.44444 | 0.25000 | 0.16667 |
| 14-120 | 0.14286 | 0.10000 | 0.00000 | 0.00000 | 0.00000 | 0.00000 | 0.08333 | 0.16667 | 0.00000 | 0.12500 | 0.00000 | 0.00000 | 0.00000 | 0.00000 | 0.10000 | 0.20000 | 0.00000 | 0.11111 | 0.37500 | 0.66667 |
| 14-121 | 0.00000 | 0.10000 | 0.00000 | 0.00000 | 0.00000 | 0.00000 | 0.00000 | 0.00000 | 0.00000 | 0.00000 | 0.00000 | 0.00000 | 0.00000 | 0.00000 | 0.00000 | 0.10000 | 0.00000 | 0.11111 | 0.37500 | 0.16667 |
| 14-122 | 0.00000 | 0.00000 | 0.00000 | 0.08333 | 0.00000 | 0.00000 | 0.00000 | 0.00000 | 0.00000 | 0.00000 | 0.16667 | 0.00000 | 0.00000 | 0.00000 | 0.00000 | 0.00000 | 0.08333 | 0.33333 | 0.12500 | 0.16667 |
| 14-123 | 0.00000 | 0.00000 | 0.00000 | 0.00000 | 0.08333 | 0.00000 | 0.00000 | 0.00000 | 0.00000 | 0.00000 | 0.00000 | 0.00000 | 0.00000 | 0.00000 | 0.00000 | 0.10000 | 0.00000 | 0.11111 | 0.00000 | 0.16667 |
| 14-124 | 0.42857 | 0.00000 | 0.00000 | 0.00000 | 0.00000 | 0.00000 | 0.00000 | 0.00000 | 0.00000 | 0.00000 | 0.00000 | 0.00000 | 0.00000 | 0.00000 | 0.00000 | 0.00000 | 0.00000 | 0.00000 | 0.00000 | 0.00000 |
| 14-125 | 0.42857 | 0.00000 | 0.00000 | 0.08333 | 0.00000 | 0.00000 | 0.08333 | 0.00000 | 0.00000 | 0.00000 | 0.00000 | 0.00000 | 0.00000 | 0.00000 | 0.00000 | 0.10000 | 0.00000 | 0.11111 | 0.00000 | 0.16667 |
| 14-126 | 0.00000 | 0.00000 | 0.00000 | 0.00000 | 0.00000 | 0.00000 | 0.00000 | 0.00000 | 0.00000 | 0.00000 | 0.00000 | 0.00000 | 0.00000 | 0.00000 | 0.00000 | 0.00000 | 0.00000 | 0.11111 | 0.00000 | 0.00000 |
| 14-127 | 0.00000 | 0.10000 | 0.00000 | 0.08333 | 0.00000 | 0.33333 | 0.08333 | 0.00000 | 0.00000 | 0.00000 | 0.00000 | 0.00000 | 0.00000 | 0.00000 | 0.00000 | 0.10000 | 0.00000 | 0.00000 | 0.00000 | 0.00000 |
| 14-128 | 0.00000 | 0.00000 | 0.00000 | 0.00000 | 0.00000 | 0.00000 | 0.00000 | 0.00000 | 0.00000 | 0.00000 | 0.00000 | 0.00000 | 0.00000 | 0.00000 | 0.00000 | 0.00000 | 0.08333 | 0.00000 | 0.25000 | 0.00000 |
| 14-129 | 0.00000 | 0.00000 | 0.00000 | 0.00000 | 0.00000 | 0.00000 | 0.00000 | 0.00000 | 0.00000 | 0.00000 | 0.00000 | 0.00000 | 0.00000 | 0.00000 | 0.00000 | 0.00000 | 0.08333 | 0.00000 | 0.25000 | 0.00000 |
| 14-130 | 0.00000 | 0.00000 | 0.00000 | 0.00000 | 0.00000 | 0.00000 | 0.00000 | 0.00000 | 0.00000 | 0.00000 | 0.00000 | 0.00000 | 0.00000 | 0.00000 | 0.00000 | 0.00000 | 0.00000 | 0.00000 | 0.00000 | 0.16667 |
| 14-131 | 0.00000 | 0.00000 | 0.00000 | 0.08333 | 0.00000 | 0.00000 | 0.00000 | 0.00000 | 0.00000 | 0.00000 | 0.00000 | 0.00000 | 0.00000 | 0.00000 | 0.00000 | 0.00000 | 0.00000 | 0.00000 | 0.00000 | 0.00000 |
| 14-132 | 0.14286 | 0.00000 | 0.00000 | 0.00000 | 0.08333 | 0.00000 | 0.00000 | 0.00000 | 0.00000 | 0.00000 | 0.00000 | 0.00000 | 0.00000 | 0.00000 | 0.00000 | 0.00000 | 0.00000 | 0.00000 | 0.00000 | 0.16667 |
| 14-133 | 0.00000 | 0.00000 | 0.00000 | 0.00000 | 0.00000 | 0.00000 | 0.00000 | 0.00000 | 0.00000 | 0.00000 | 0.00000 | 0.00000 | 0.00000 | 0.00000 | 0.00000 | 0.00000 | 0.00000 | 0.00000 | 0.00000 | 0.16667 |
| 14-134 | 0.00000 | 0.00000 | 0.00000 | 0.00000 | 0.00000 | 0.00000 | 0.00000 | 0.00000 | 0.08333 | 0.00000 | 0.00000 | 0.00000 | 0.00000 | 0.00000 | 0.00000 | 0.00000 | 0.00000 | 0.00000 | 0.00000 | 0.00000 |
| 14-135 | 0.00000 | 0.10000 | 0.00000 | 0.00000 | 0.00000 | 0.00000 | 0.00000 | 0.00000 | 0.08333 | 0.00000 | 0.00000 | 0.00000 | 0.00000 | 0.00000 | 0.00000 | 0.10000 | 0.00000 | 0.11111 | 0.00000 | 0.16667 |
| 14-136 | 0.00000 | 0.10000 | 0.00000 | 0.00000 | 0.00000 | 0.00000 | 0.00000 | 0.00000 | 0.00000 | 0.00000 | 0.00000 | 0.00000 | 0.00000 | 0.00000 | 0.00000 | 0.00000 | 0.00000 | 0.00000 | 0.00000 | 0.16667 |
| 14-137 | 0.00000 | 0.00000 | 0.00000 | 0.00000 | 0.00000 | 0.00000 | 0.00000 | 0.00000 | 0.08333 | 0.00000 | 0.00000 | 0.00000 | 0.00000 | 0.00000 | 0.00000 | 0.00000 | 0.00000 | 0.00000 | 0.00000 | 0.00000 |
| 14-138 | 0.00000 | 0.00000 | 0.00000 | 0.00000 | 0.00000 | 0.00000 | 0.00000 | 0.00000 | 0.00000 | 0.00000 | 0.00000 | 0.00000 | 0.00000 | 0.00000 | 0.00000 | 0.00000 | 0.00000 | 0.11111 | 0.00000 | 0.16667 |
| 14-139 | 0.00000 | 0.00000 | 0.00000 | 0.00000 | 0.00000 | 0.00000 | 0.00000 | 0.00000 | 0.08333 | 0.00000 | 0.00000 | 0.00000 | 0.00000 | 0.00000 | 0.00000 | 0.00000 | 0.00000 | 0.11111 | 0.00000 | 0.16667 |
| 14-140 | 0.00000 | 0.10000 | 0.00000 | 0.00000 | 0.00000 | 0.00000 | 0.00000 | 0.00000 | 0.00000 | 0.00000 | 0.00000 | 0.00000 | 0.00000 | 0.00000 | 0.00000 | 0.00000 | 0.00000 | 0.00000 | 0.00000 | 0.00000 |
| 14-141 | 0.00000 | 0.10000 | 0.00000 | 0.00000 | 0.00000 | 0.00000 | 0.00000 | 0.00000 | 0.00000 | 0.00000 | 0.00000 | 0.00000 | 0.00000 | 0.00000 | 0.00000 | 0.00000 | 0.08333 | 0.44444 | 0.12500 | 0.16667 |
| 14-142 | 0.00000 | 0.00000 | 0.00000 | 0.00000 | 0.00000 | 0.00000 | 0.00000 | 0.00000 | 0.00000 | 0.00000 | 0.00000 | 0.00000 | 0.00000 | 0.00000 | 0.00000 | 0.00000 | 0.00000 | 0.11111 | 0.00000 | 0.33333 |
| 14-143 | 0.00000 | 0.00000 | 0.00000 | 0.00000 | 0.00000 | 0.00000 | 0.08333 | 0.00000 | 0.00000 | 0.00000 | 0.00000 | 0.00000 | 0.00000 | 0.00000 | 0.00000 | 0.00000 | 0.00000 | 0.00000 | 0.00000 | 0.00000 |
| 14-144 | 0.00000 | 0.00000 | 0.00000 | 0.00000 | 0.00000 | 0.00000 | 0.16667 | 0.00000 | 0.00000 | 0.00000 | 0.00000 | 0.00000 | 0.00000 | 0.00000 | 0.00000 | 0.00000 | 0.00000 | 0.00000 | 0.00000 | 0.00000 |
| 14-145 | 0.00000 | 0.00000 | 0.00000 | 0.00000 | 0.00000 | 0.16667 | 0.00000 | 0.00000 | 0.00000 | 0.00000 | 0.00000 | 0.00000 | 0.00000 | 0.00000 | 0.00000 | 0.00000 | 0.00000 | 0.11111 | 0.00000 | 0.16667 |
| 17-001 | 0.00000 | 0.11111 | 0.00000 | 0.00000 | 0.00000 | 0.16667 | 0.00000 | 0.00000 | 0.08333 | 0.00000 | 0.00000 | 0.00000 | 0.00000 | 0.00000 | 0.00000 | 0.00000 | 0.00000 | 0.00000 | 0.00000 | 0.00000 |
| 17-002 | 0.00000 | 0.00000 | 0.00000 | 0.00000 | 0.00000 | 0.00000 | 0.08333 | 0.16667 | 0.00000 | 0.00000 | 0.00000 | 0.08333 | 0.00000 | 0.00000 | 0.00000 | 0.00000 | 0.00000 | 0.00000 | 0.00000 | 0.00000 |
| 17-003 | 0.00000 | 0.11111 | 0.00000 | 0.00000 | 0.00000 | 0.16667 | 0.08333 | 0.16667 | 0.00000 | 0.00000 | 0.27273 | 0.16667 | 0.00000 | 0.00000 | 0.00000 | 0.00000 | 0.00000 | 0.00000 | 0.00000 | 0.16667 |
| 17-004 | 0.00000 | 0.11111 | 0.00000 | 0.00000 | 0.00000 | 0.16667 | 0.00000 | 0.00000 | 0.08333 | 0.00000 | 0.00000 | 0.00000 | 0.30000 | 0.25000 | 0.50000 | 0.00000 | 0.00000 | 0.00000 | 0.25000 | 0.00000 |
| 17-005 | 0.00000 | 0.00000 | 0.00000 | 0.00000 | 0.00000 | 0.00000 | 0.00000 | 0.00000 | 0.00000 | 0.00000 | 0.00000 | 0.08333 | 0.10000 | 0.08333 | 0.30000 | 0.00000 | 0.00000 | 0.00000 | 0.25000 | 0.00000 |
| 17-006 | 0.00000 | 0.00000 | 0.00000 | 0.00000 | 0.00000 | 0.16667 | 0.00000 | 0.00000 | 0.00000 | 0.00000 | 0.00000 | 0.16667 | 0.00000 | 0.00000 | 0.00000 | 0.00000 | 0.00000 | 0.00000 | 0.00000 | 0.16667 |
| 17-007 | 0.00000 | 0.00000 | 0.08333 | 0.08333 | 0.00000 | 0.33333 | 0.41667 | 0.50000 | 0.33333 | 0.11111 | 0.27273 | 0.08333 | 0.00000 | 0.16667 | 0.00000 | 0.12500 | 0.09091 | 0.00000 | 0.00000 | 0.33333 |
| 17-008 | 0.00000 | 0.00000 | 0.00000 | 0.00000 | 0.08333 | 0.16667 | 0.00000 | 0.00000 | 0.00000 | 0.00000 | 0.00000 | 0.00000 | 0.00000 | 0.00000 | 0.00000 | 0.00000 | 0.00000 | 0.00000 | 0.00000 | 0.00000 |
| 17-009 | 0.00000 | 0.00000 | 0.00000 | 0.00000 | 0.00000 | 0.00000 | 0.00000 | 0.00000 | 0.00000 | 0.00000 | 0.00000 | 0.00000 | 0.00000 | 0.08333 | 0.00000 | 0.00000 | 0.00000 | 0.00000 | 0.00000 | 0.00000 |
| 17-010 | 0.00000 | 0.00000 | 0.00000 | 0.00000 | 0.00000 | 0.00000 | 0.00000 | 0.00000 | 0.00000 | 0.00000 | 0.18182 | 0.08333 | 0.10000 | 0.08333 | 0.00000 | 0.00000 | 0.09091 | 0.00000 | 0.00000 | 0.00000 |
| 17-011 | 0.00000 | 0.00000 | 0.08333 | 0.00000 | 0.00000 | 0.16667 | 0.00000 | 0.00000 | 0.08333 | 0.11111 | 0.09091 | 0.16667 | 0.30000 | 0.16667 | 0.20000 | 0.12500 | 0.09091 | 0.00000 | 0.00000 | 0.00000 |
| 17-012 | 0.00000 | 0.00000 | 0.00000 | 0.00000 | 0.00000 | 0.00000 | 0.00000 | 0.16667 | 0.00000 | 0.22222 | 0.00000 | 0.08333 | 0.00000 | 0.00000 | 0.00000 | 0.12500 | 0.00000 | 0.00000 | 0.00000 | 0.00000 |
| 17-013 | 0.00000 | 0.00000 | 0.00000 | 0.00000 | 0.08333 | 0.16667 | 0.00000 | 0.16667 | 0.00000 | 0.00000 | 0.18182 | 0.08333 | 0.10000 | 0.00000 | 0.10000 | 0.00000 | 0.00000 | 0.00000 | 0.00000 | 0.00000 |
| 17-014 | 0.12500 | 0.00000 | 0.08333 | 0.00000 | 0.08333 | 0.00000 | 0.00000 | 0.00000 | 0.00000 | 0.00000 | 0.00000 | 0.00000 | 0.10000 | 0.00000 | 0.00000 | 0.00000 | 0.00000 | 0.00000 | 0.00000 | 0.00000 |
| 17-015 | 0.00000 | 0.00000 | 0.16667 | 0.00000 | 0.08333 | 0.16667 | 0.08333 | 0.16667 | 0.00000 | 0.00000 | 0.27273 | 0.25000 | 0.00000 | 0.08333 | 0.00000 | 0.00000 | 0.09091 | 0.00000 | 0.00000 | 0.00000 |
| 17-016 | 0.12500 | 0.00000 | 0.08333 | 0.00000 | 0.00000 | 0.16667 | 0.08333 | 0.16667 | 0.00000 | 0.11111 | 0.27273 | 0.16667 | 0.00000 | 0.08333 | 0.00000 | 0.00000 | 0.09091 | 0.00000 | 0.00000 | 0.00000 |
| 17-017 | 0.00000 | 0.00000 | 0.08333 | 0.00000 | 0.00000 | 0.16667 | 0.00000 | 0.00000 | 0.00000 | 0.00000 | 0.18182 | 0.25000 | 0.10000 | 0.00000 | 0.10000 | 0.00000 | 0.09091 | 0.00000 | 0.00000 | 0.00000 |
| 17-018 | 0.00000 | 0.11111 | 0.16667 | 0.16667 | 0.08333 | 0.00000 | 0.00000 | 0.00000 | 0.00000 | 0.11111 | 0.09091 | 0.33333 | 0.00000 | 0.00000 | 0.10000 | 0.00000 | 0.09091 | 0.00000 | 0.00000 | 0.00000 |
| 17-019 | 0.00000 | 0.11111 | 0.25000 | 0.16667 | 0.08333 | 0.16667 | 0.08333 | 0.00000 | 0.00000 | 0.11111 | 0.09091 | 0.16667 | 0.00000 | 0.00000 | 0.00000 | 0.00000 | 0.00000 | 0.00000 | 0.00000 | 0.16667 |
| 17-020 | 0.00000 | 0.00000 | 0.08333 | 0.00000 | 0.00000 | 0.16667 | 0.08333 | 0.16667 | 0.00000 | 0.00000 | 0.09091 | 0.16667 | 0.00000 | 0.00000 | 0.20000 | 0.00000 | 0.09091 | 0.00000 | 0.00000 | 0.00000 |
| 17-021 | 0.00000 | 0.00000 | 0.00000 | 0.00000 | 0.00000 | 0.00000 | 0.00000 | 0.00000 | 0.00000 | 0.00000 | 0.00000 | 0.08333 | 0.00000 | 0.00000 | 0.10000 | 0.00000 | 0.00000 | 0.00000 | 0.00000 | 0.00000 |
| 17-022 | 0.00000 | 0.00000 | 0.00000 | 0.00000 | 0.00000 | 0.00000 | 0.00000 | 0.00000 | 0.08333 | 0.00000 | 0.09091 | 0.00000 | 0.00000 | 0.00000 | 0.00000 | 0.00000 | 0.09091 | 0.00000 | 0.00000 | 0.00000 |
| 17-023 | 0.12500 | 0.22222 | 0.25000 | 0.00000 | 0.08333 | 0.50000 | 0.16667 | 0.16667 | 0.33333 | 0.00000 | 0.09091 | 0.75000 | 0.70000 | 0.41667 | 0.30000 | 0.50000 | 0.36364 | 0.00000 | 0.12500 | 0.33333 |
| 17-024 | 0.12500 | 0.11111 | 0.33333 | 0.08333 | 0.08333 | 0.50000 | 0.16667 | 0.16667 | 0.50000 | 0.00000 | 0.09091 | 0.58333 | 0.80000 | 0.58333 | 0.20000 | 0.62500 | 0.27273 | 0.00000 | 0.12500 | 0.33333 |
| 17-025 | 0.00000 | 0.00000 | 0.00000 | 0.00000 | 0.00000 | 0.16667 | 0.00000 | 0.16667 | 0.08333 | 0.00000 | 0.00000 | 0.00000 | 0.10000 | 0.16667 | 0.10000 | 0.00000 | 0.00000 | 0.00000 | 0.00000 | 0.00000 |
| 17-026 | 0.00000 | 0.11111 | 0.08333 | 0.00000 | 0.00000 | 0.33333 | 0.00000 | 0.00000 | 0.16667 | 0.00000 | 0.09091 | 0.08333 | 0.00000 | 0.16667 | 0.00000 | 0.00000 | 0.09091 | 0.00000 | 0.12500 | 0.00000 |
| 17-027 | 0.37500 | 0.22222 | 0.33333 | 0.33333 | 0.50000 | 0.50000 | 0.08333 | 0.16667 | 0.33333 | 0.22222 | 0.27273 | 0.58333 | 0.50000 | 0.50000 | 0.10000 | 0.25000 | 0.45455 | 0.00000 | 0.25000 | 0.16667 |
| 17-028 | 0.37500 | 0.33333 | 0.25000 | 0.25000 | 0.50000 | 0.33333 | 0.00000 | 0.33333 | 0.33333 | 0.11111 | 0.27273 | 0.50000 | 0.40000 | 0.33333 | 0.10000 | 0.25000 | 0.36364 | 0.11111 | 0.37500 | 0.16667 |
| 17-029 | 0.00000 | 0.00000 | 0.00000 | 0.08333 | 0.00000 | 0.16667 | 0.08333 | 0.16667 | 0.08333 | 0.11111 | 0.09091 | 0.08333 | 0.00000 | 0.08333 | 0.00000 | 0.00000 | 0.00000 | 0.00000 | 0.00000 | 0.16667 |
| 17-030 | 0.00000 | 0.00000 | 0.16667 | 0.00000 | 0.08333 | 0.16667 | 0.00000 | 0.00000 | 0.08333 | 0.00000 | 0.18182 | 0.25000 | 0.20000 | 0.16667 | 0.10000 | 0.00000 | 0.18182 | 0.00000 | 0.12500 | 0.33333 |
| 17-031 | 0.00000 | 0.00000 | 0.16667 | 0.08333 | 0.00000 | 0.00000 | 0.08333 | 0.00000 | 0.00000 | 0.00000 | 0.09091 | 0.08333 | 0.10000 | 0.08333 | 0.10000 | 0.00000 | 0.18182 | 0.00000 | 0.00000 | 0.16667 |
| 17-032 | 0.12500 | 0.00000 | 0.00000 | 0.00000 | 0.00000 | 0.33333 | 0.16667 | 0.66667 | 0.16667 | 0.11111 | 0.09091 | 0.08333 | 0.10000 | 0.33333 | 0.10000 | 0.00000 | 0.00000 | 0.00000 | 0.12500 | 0.33333 |
| 17-033 | 0.00000 | 0.00000 | 0.00000 | 0.08333 | 0.00000 | 0.33333 | 0.25000 | 0.16667 | 0.41667 | 0.33333 | 0.18182 | 0.16667 | 0.10000 | 0.33333 | 0.40000 | 0.25000 | 0.18182 | 0.00000 | 0.12500 | 0.00000 |
| 17-034 | 0.12500 | 0.00000 | 0.16667 | 0.08333 | 0.00000 | 0.50000 | 0.25000 | 0.66667 | 0.50000 | 0.33333 | 0.27273 | 0.50000 | 0.10000 | 0.33333 | 0.40000 | 0.37500 | 0.09091 | 0.00000 | 0.12500 | 1.00000 |
| 17-035 | 0.00000 | 0.00000 | 0.08333 | 0.00000 | 0.00000 | 0.16667 | 0.00000 | 0.33333 | 0.00000 | 0.00000 | 0.00000 | 0.16667 | 0.00000 | 0.00000 | 0.00000 | 0.00000 | 0.00000 | 0.00000 | 0.00000 | 0.00000 |
| 17-036 | 0.00000 | 0.11111 | 0.00000 | 0.00000 | 0.00000 | 0.16667 | 0.08333 | 0.50000 | 0.00000 | 0.00000 | 0.00000 | 0.00000 | 0.10000 | 0.16667 | 0.00000 | 0.00000 | 0.00000 | 0.00000 | 0.00000 | 0.00000 |
| 17-037 | 0.00000 | 0.11111 | 0.00000 | 0.08333 | 0.00000 | 0.33333 | 0.25000 | 0.50000 | 0.08333 | 0.00000 | 0.18182 | 0.00000 | 0.00000 | 0.08333 | 0.00000 | 0.00000 | 0.00000 | 0.00000 | 0.00000 | 0.50000 |
| 17-038 | 0.00000 | 0.11111 | 0.00000 | 0.16667 | 0.08333 | 0.16667 | 0.33333 | 0.16667 | 0.33333 | 0.11111 | 0.27273 | 0.08333 | 0.20000 | 0.33333 | 0.00000 | 0.12500 | 0.36364 | 0.11111 | 0.25000 | 0.33333 |
| 17-039 | 0.00000 | 0.00000 | 0.00000 | 0.08333 | 0.00000 | 0.00000 | 0.00000 | 0.33333 | 0.08333 | 0.11111 | 0.00000 | 0.00000 | 0.20000 | 0.25000 | 0.00000 | 0.12500 | 0.27273 | 0.00000 | 0.00000 | 0.16667 |
| 17-040 | 0.12500 | 0.00000 | 0.00000 | 0.08333 | 0.00000 | 0.00000 | 0.16667 | 0.16667 | 0.08333 | 0.00000 | 0.09091 | 0.08333 | 0.00000 | 0.08333 | 0.00000 | 0.00000 | 0.00000 | 0.00000 | 0.00000 | 0.16667 |
| 17-041 | 0.00000 | 0.00000 | 0.00000 | 0.00000 | 0.00000 | 0.00000 | 0.00000 | 0.16667 | 0.00000 | 0.00000 | 0.00000 | 0.00000 | 0.10000 | 0.16667 | 0.00000 | 0.12500 | 0.00000 | 0.00000 | 0.00000 | 0.16667 |
| 17-042 | 0.00000 | 0.11111 | 0.33333 | 0.00000 | 0.00000 | 0.16667 | 0.00000 | 0.00000 | 0.08333 | 0.00000 | 0.00000 | 0.08333 | 0.00000 | 0.00000 | 0.10000 | 0.00000 | 0.09091 | 0.00000 | 0.12500 | 0.16667 |
| 17-043 | 0.00000 | 0.00000 | 0.08333 | 0.00000 | 0.00000 | 0.00000 | 0.16667 | 0.00000 | 0.08333 | 0.00000 | 0.09091 | 0.08333 | 0.00000 | 0.25000 | 0.10000 | 0.12500 | 0.00000 | 0.00000 | 0.00000 | 0.00000 |
| 17-044 | 0.12500 | 0.11111 | 0.16667 | 0.08333 | 0.08333 | 0.00000 | 0.08333 | 0.33333 | 0.08333 | 0.00000 | 0.18182 | 0.33333 | 0.20000 | 0.25000 | 0.00000 | 0.12500 | 0.36364 | 0.55556 | 0.37500 | 0.50000 |
| 17-045 | 0.00000 | 0.00000 | 0.00000 | 0.00000 | 0.08333 | 0.00000 | 0.16667 | 0.00000 | 0.00000 | 0.00000 | 0.00000 | 0.00000 | 0.00000 | 0.08333 | 0.20000 | 0.00000 | 0.18182 | 0.11111 | 0.00000 | 0.16667 |
| 17-046 | 0.00000 | 0.33333 | 0.00000 | 0.33333 | 0.00000 | 0.00000 | 0.16667 | 0.16667 | 0.00000 | 0.00000 | 0.27273 | 0.33333 | 0.00000 | 0.16667 | 0.30000 | 0.37500 | 0.18182 | 0.66667 | 0.25000 | 0.00000 |
| 17-047 | 0.12500 | 0.33333 | 0.08333 | 0.33333 | 0.00000 | 0.16667 | 0.33333 | 0.16667 | 0.00000 | 0.11111 | 0.45455 | 0.58333 | 0.00000 | 0.08333 | 0.20000 | 0.37500 | 0.36364 | 0.55556 | 0.12500 | 0.00000 |
| 17-048 | 0.00000 | 0.00000 | 0.16667 | 0.16667 | 0.08333 | 0.33333 | 0.00000 | 0.50000 | 0.25000 | 0.11111 | 0.09091 | 0.08333 | 0.00000 | 0.08333 | 0.00000 | 0.00000 | 0.09091 | 0.00000 | 0.00000 | 0.50000 |
| 17-049 | 0.00000 | 0.11111 | 0.25000 | 0.16667 | 0.16667 | 0.33333 | 0.00000 | 0.33333 | 0.58333 | 0.00000 | 0.36364 | 0.50000 | 0.40000 | 0.50000 | 0.80000 | 0.37500 | 0.36364 | 0.00000 | 0.75000 | 0.66667 |
| 17-050 | 0.00000 | 0.00000 | 0.00000 | 0.00000 | 0.00000 | 0.00000 | 0.00000 | 0.00000 | 0.00000 | 0.00000 | 0.18182 | 0.00000 | 0.00000 | 0.00000 | 0.10000 | 0.12500 | 0.00000 | 0.00000 | 0.00000 | 0.00000 |
| 17-051 | 0.00000 | 0.00000 | 0.00000 | 0.00000 | 0.00000 | 0.00000 | 0.00000 | 0.00000 | 0.00000 | 0.00000 | 0.00000 | 0.00000 | 0.00000 | 0.00000 | 0.10000 | 0.00000 | 0.00000 | 0.00000 | 0.00000 | 0.00000 |
| 17-052 | 0.00000 | 0.00000 | 0.08333 | 0.00000 | 0.00000 | 0.33333 | 0.08333 | 0.00000 | 0.25000 | 0.00000 | 0.09091 | 0.00000 | 0.10000 | 0.25000 | 0.00000 | 0.00000 | 0.00000 | 0.00000 | 0.12500 | 0.00000 |
| 17-053 | 0.00000 | 0.00000 | 0.08333 | 0.00000 | 0.00000 | 0.00000 | 0.16667 | 0.00000 | 0.00000 | 0.00000 | 0.00000 | 0.00000 | 0.00000 | 0.08333 | 0.00000 | 0.00000 | 0.00000 | 0.00000 | 0.00000 | 0.00000 |
| 17-054 | 0.00000 | 0.11111 | 0.00000 | 0.00000 | 0.00000 | 0.00000 | 0.00000 | 0.00000 | 0.00000 | 0.00000 | 0.09091 | 0.00000 | 0.00000 | 0.25000 | 0.00000 | 0.00000 | 0.09091 | 0.00000 | 0.00000 | 0.66667 |
| 17-055 | 0.00000 | 0.00000 | 0.08333 | 0.00000 | 0.08333 | 0.00000 | 0.00000 | 0.00000 | 0.00000 | 0.00000 | 0.18182 | 0.08333 | 0.00000 | 0.00000 | 0.00000 | 0.00000 | 0.09091 | 0.00000 | 0.00000 | 0.16667 |
| 17-056 | 0.00000 | 0.00000 | 0.16667 | 0.16667 | 0.00000 | 0.00000 | 0.08333 | 0.00000 | 0.08333 | 0.00000 | 0.09091 | 0.08333 | 0.00000 | 0.08333 | 0.00000 | 0.00000 | 0.09091 | 0.11111 | 0.00000 | 0.33333 |
| 17-057 | 0.00000 | 0.00000 | 0.16667 | 0.16667 | 0.08333 | 0.00000 | 0.08333 | 0.00000 | 0.08333 | 0.00000 | 0.18182 | 0.16667 | 0.00000 | 0.08333 | 0.00000 | 0.00000 | 0.00000 | 0.11111 | 0.00000 | 0.33333 |
| 17-058 | 0.12500 | 0.00000 | 0.00000 | 0.00000 | 0.00000 | 0.00000 | 0.00000 | 0.00000 | 0.00000 | 0.00000 | 0.00000 | 0.00000 | 0.00000 | 0.16667 | 0.00000 | 0.00000 | 0.00000 | 0.00000 | 0.00000 | 0.00000 |
| 17-059 | 0.00000 | 0.00000 | 0.08333 | 0.00000 | 0.16667 | 0.16667 | 0.16667 | 0.00000 | 0.00000 | 0.00000 | 0.09091 | 0.00000 | 0.00000 | 0.08333 | 0.00000 | 0.00000 | 0.00000 | 0.00000 | 0.00000 | 0.00000 |
| 17-060 | 0.00000 | 0.00000 | 0.08333 | 0.08333 | 0.16667 | 0.33333 | 0.08333 | 0.00000 | 0.00000 | 0.00000 | 0.18182 | 0.25000 | 0.10000 | 0.41667 | 0.00000 | 0.00000 | 0.09091 | 0.00000 | 0.00000 | 0.50000 |
| 17-061 | 0.00000 | 0.00000 | 0.00000 | 0.00000 | 0.00000 | 0.16667 | 0.00000 | 0.00000 | 0.00000 | 0.00000 | 0.00000 | 0.00000 | 0.00000 | 0.00000 | 0.00000 | 0.00000 | 0.00000 | 0.00000 | 0.00000 | 0.00000 |
| 17-062 | 0.00000 | 0.00000 | 0.00000 | 0.00000 | 0.08333 | 0.00000 | 0.00000 | 0.00000 | 0.00000 | 0.00000 | 0.00000 | 0.00000 | 0.00000 | 0.00000 | 0.00000 | 0.00000 | 0.09091 | 0.00000 | 0.00000 | 0.16667 |
| 17-063 | 0.00000 | 0.00000 | 0.16667 | 0.00000 | 0.08333 | 0.16667 | 0.08333 | 0.16667 | 0.00000 | 0.11111 | 0.00000 | 0.00000 | 0.00000 | 0.00000 | 0.00000 | 0.00000 | 0.18182 | 0.00000 | 0.00000 | 0.16667 |
| 17-064 | 0.00000 | 0.00000 | 0.08333 | 0.00000 | 0.00000 | 0.16667 | 0.00000 | 0.00000 | 0.00000 | 0.11111 | 0.00000 | 0.00000 | 0.00000 | 0.00000 | 0.00000 | 0.00000 | 0.09091 | 0.00000 | 0.00000 | 0.00000 |
| 17-065 | 0.00000 | 0.00000 | 0.08333 | 0.00000 | 0.00000 | 0.16667 | 0.00000 | 0.00000 | 0.00000 | 0.00000 | 0.00000 | 0.00000 | 0.00000 | 0.08333 | 0.00000 | 0.12500 | 0.00000 | 0.11111 | 0.00000 | 0.00000 |
| 17-066 | 0.00000 | 0.11111 | 0.08333 | 0.00000 | 0.00000 | 0.00000 | 0.08333 | 0.00000 | 0.00000 | 0.00000 | 0.00000 | 0.08333 | 0.00000 | 0.00000 | 0.00000 | 0.00000 | 0.00000 | 0.11111 | 0.00000 | 0.00000 |
| 17-067 | 0.12500 | 0.00000 | 0.00000 | 0.00000 | 0.00000 | 0.00000 | 0.08333 | 0.16667 | 0.00000 | 0.00000 | 0.00000 | 0.08333 | 0.00000 | 0.00000 | 0.00000 | 0.00000 | 0.00000 | 0.00000 | 0.00000 | 0.00000 |
| 17-068 | 0.12500 | 0.00000 | 0.08333 | 0.00000 | 0.00000 | 0.00000 | 0.16667 | 0.00000 | 0.16667 | 0.00000 | 0.00000 | 0.00000 | 0.00000 | 0.00000 | 0.00000 | 0.00000 | 0.00000 | 0.00000 | 0.00000 | 0.00000 |
| 17-069 | 0.12500 | 0.33333 | 0.16667 | 0.25000 | 0.08333 | 0.16667 | 0.08333 | 0.00000 | 0.16667 | 0.33333 | 0.09091 | 0.16667 | 0.00000 | 0.08333 | 0.50000 | 0.12500 | 0.18182 | 0.00000 | 0.12500 | 0.00000 |
| 17-070 | 0.12500 | 0.00000 | 0.00000 | 0.00000 | 0.00000 | 0.00000 | 0.08333 | 0.16667 | 0.00000 | 0.00000 | 0.00000 | 0.00000 | 0.00000 | 0.00000 | 0.00000 | 0.00000 | 0.00000 | 0.00000 | 0.00000 | 0.00000 |
| 17-071 | 0.00000 | 0.00000 | 0.00000 | 0.00000 | 0.00000 | 0.16667 | 0.16667 | 0.00000 | 0.00000 | 0.00000 | 0.00000 | 0.00000 | 0.00000 | 0.25000 | 0.00000 | 0.00000 | 0.00000 | 0.00000 | 0.00000 | 0.50000 |
| 17-072 | 0.00000 | 0.00000 | 0.08333 | 0.00000 | 0.00000 | 0.00000 | 0.00000 | 0.16667 | 0.00000 | 0.00000 | 0.00000 | 0.00000 | 0.00000 | 0.08333 | 0.00000 | 0.25000 | 0.09091 | 0.00000 | 0.00000 | 0.16667 |
| 17-073 | 0.00000 | 0.11111 | 0.25000 | 0.16667 | 0.08333 | 0.16667 | 0.00000 | 0.00000 | 0.33333 | 0.00000 | 0.00000 | 0.00000 | 0.40000 | 0.25000 | 0.30000 | 0.25000 | 0.36364 | 0.00000 | 0.00000 | 0.16667 |
| 17-074 | 0.00000 | 0.11111 | 0.00000 | 0.08333 | 0.08333 | 0.00000 | 0.08333 | 0.00000 | 0.16667 | 0.00000 | 0.09091 | 0.08333 | 0.00000 | 0.00000 | 0.00000 | 0.12500 | 0.00000 | 0.00000 | 0.00000 | 0.50000 |
| 17-075 | 0.00000 | 0.00000 | 0.00000 | 0.00000 | 0.00000 | 0.00000 | 0.25000 | 0.00000 | 0.00000 | 0.00000 | 0.00000 | 0.00000 | 0.00000 | 0.00000 | 0.00000 | 0.12500 | 0.09091 | 0.00000 | 0.00000 | 0.00000 |
| 17-076 | 0.00000 | 0.11111 | 0.08333 | 0.00000 | 0.08333 | 0.00000 | 0.25000 | 0.00000 | 0.08333 | 0.00000 | 0.00000 | 0.00000 | 0.00000 | 0.00000 | 0.10000 | 0.00000 | 0.00000 | 0.00000 | 0.00000 | 0.00000 |
| 17-077 | 0.12500 | 0.11111 | 0.00000 | 0.08333 | 0.00000 | 0.33333 | 0.16667 | 0.33333 | 0.16667 | 0.00000 | 0.27273 | 0.08333 | 0.00000 | 0.16667 | 0.10000 | 0.00000 | 0.00000 | 0.11111 | 0.00000 | 0.66667 |
| 17-078 | 0.00000 | 0.22222 | 0.16667 | 0.00000 | 0.00000 | 0.33333 | 0.08333 | 0.16667 | 0.00000 | 0.11111 | 0.36364 | 0.33333 | 0.00000 | 0.00000 | 0.00000 | 0.00000 | 0.09091 | 0.00000 | 0.00000 | 0.00000 |
| 17-079 | 0.00000 | 0.22222 | 0.16667 | 0.00000 | 0.08333 | 0.16667 | 0.16667 | 0.33333 | 0.00000 | 0.00000 | 0.27273 | 0.41667 | 0.00000 | 0.00000 | 0.00000 | 0.00000 | 0.00000 | 0.00000 | 0.00000 | 0.16667 |
| 17-080 | 0.75000 | 0.11111 | 0.16667 | 0.08333 | 0.33333 | 0.33333 | 0.08333 | 0.16667 | 0.00000 | 0.00000 | 0.27273 | 0.25000 | 0.40000 | 0.00000 | 0.00000 | 0.00000 | 0.09091 | 0.22222 | 0.00000 | 0.00000 |
| 17-081 | 0.00000 | 0.00000 | 0.00000 | 0.00000 | 0.00000 | 0.00000 | 0.08333 | 0.00000 | 0.00000 | 0.00000 | 0.00000 | 0.00000 | 0.00000 | 0.08333 | 0.00000 | 0.00000 | 0.00000 | 0.00000 | 0.00000 | 0.16667 |
| 17-082 | 1.00000 | 0.00000 | 0.16667 | 0.08333 | 0.25000 | 0.50000 | 0.08333 | 0.16667 | 0.00000 | 0.00000 | 0.09091 | 0.25000 | 0.40000 | 0.16667 | 0.00000 | 0.00000 | 0.00000 | 0.22222 | 0.00000 | 0.16667 |
| 17-083 | 0.00000 | 0.00000 | 0.00000 | 0.00000 | 0.00000 | 0.16667 | 0.08333 | 0.00000 | 0.00000 | 0.00000 | 0.00000 | 0.00000 | 0.20000 | 0.08333 | 0.00000 | 0.00000 | 0.00000 | 0.00000 | 0.00000 | 0.00000 |
| 17-084 | 0.12500 | 0.00000 | 0.00000 | 0.00000 | 0.00000 | 0.00000 | 0.00000 | 0.00000 | 0.00000 | 0.00000 | 0.00000 | 0.00000 | 0.00000 | 0.08333 | 0.00000 | 0.00000 | 0.00000 | 0.00000 | 0.00000 | 0.00000 |
| 17-085 | 0.00000 | 0.00000 | 0.00000 | 0.00000 | 0.00000 | 0.16667 | 0.00000 | 0.00000 | 0.08333 | 0.00000 | 0.00000 | 0.00000 | 0.00000 | 0.00000 | 0.00000 | 0.00000 | 0.00000 | 0.00000 | 0.00000 | 0.00000 |
| 17-086 | 0.00000 | 0.00000 | 0.00000 | 0.00000 | 0.00000 | 0.33333 | 0.00000 | 0.00000 | 0.08333 | 0.11111 | 0.00000 | 0.00000 | 0.00000 | 0.00000 | 0.00000 | 0.00000 | 0.00000 | 0.00000 | 0.12500 | 0.00000 |
| 17-087 | 0.00000 | 0.11111 | 0.00000 | 0.00000 | 0.00000 | 0.16667 | 0.00000 | 0.00000 | 0.08333 | 0.11111 | 0.00000 | 0.00000 | 0.10000 | 0.25000 | 0.00000 | 0.00000 | 0.00000 | 0.11111 | 0.00000 | 0.00000 |
| 17-088 | 0.00000 | 0.11111 | 0.00000 | 0.08333 | 0.00000 | 0.16667 | 0.08333 | 0.16667 | 0.08333 | 0.00000 | 0.00000 | 0.00000 | 0.20000 | 0.25000 | 0.00000 | 0.00000 | 0.00000 | 0.00000 | 0.00000 | 0.00000 |
| 17-089 | 0.75000 | 0.22222 | 0.25000 | 0.08333 | 0.08333 | 0.33333 | 0.16667 | 0.16667 | 0.50000 | 0.22222 | 0.00000 | 0.08333 | 0.20000 | 0.33333 | 0.40000 | 0.12500 | 0.27273 | 0.11111 | 0.00000 | 0.83333 |
| 17-090 | 0.00000 | 0.00000 | 0.00000 | 0.00000 | 0.00000 | 0.00000 | 0.08333 | 0.16667 | 0.08333 | 0.00000 | 0.00000 | 0.00000 | 0.00000 | 0.08333 | 0.10000 | 0.00000 | 0.09091 | 0.00000 | 0.00000 | 0.00000 |
| 17-091 | 0.00000 | 0.00000 | 0.08333 | 0.00000 | 0.00000 | 0.00000 | 0.00000 | 0.16667 | 0.00000 | 0.00000 | 0.00000 | 0.00000 | 0.00000 | 0.00000 | 0.00000 | 0.00000 | 0.00000 | 0.00000 | 0.12500 | 0.00000 |
| 17-092 | 0.00000 | 0.00000 | 0.08333 | 0.00000 | 0.00000 | 0.00000 | 0.00000 | 0.00000 | 0.00000 | 0.00000 | 0.00000 | 0.00000 | 0.00000 | 0.00000 | 0.00000 | 0.00000 | 0.00000 | 0.00000 | 0.12500 | 0.00000 |
| 17-093 | 0.12500 | 0.00000 | 0.08333 | 0.00000 | 0.00000 | 0.00000 | 0.00000 | 0.16667 | 0.00000 | 0.00000 | 0.00000 | 0.00000 | 0.00000 | 0.00000 | 0.00000 | 0.12500 | 0.00000 | 0.00000 | 0.00000 | 0.00000 |
| 17-094 | 0.00000 | 0.00000 | 0.16667 | 0.00000 | 0.08333 | 0.00000 | 0.00000 | 0.16667 | 0.00000 | 0.00000 | 0.09091 | 0.00000 | 0.00000 | 0.00000 | 0.00000 | 0.00000 | 0.00000 | 0.00000 | 0.00000 | 0.00000 |
| 17-095 | 0.00000 | 0.00000 | 0.16667 | 0.00000 | 0.08333 | 0.00000 | 0.08333 | 0.16667 | 0.00000 | 0.11111 | 0.00000 | 0.00000 | 0.00000 | 0.00000 | 0.20000 | 0.00000 | 0.00000 | 0.00000 | 0.00000 | 0.00000 |
| 17-096 | 0.00000 | 0.11111 | 0.00000 | 0.00000 | 0.00000 | 0.00000 | 0.00000 | 0.00000 | 0.16667 | 0.00000 | 0.00000 | 0.00000 | 0.10000 | 0.00000 | 0.00000 | 0.12500 | 0.00000 | 0.00000 | 0.00000 | 0.16667 |
| 17-097 | 0.12500 | 0.00000 | 0.00000 | 0.00000 | 0.00000 | 0.00000 | 0.00000 | 0.00000 | 0.00000 | 0.00000 | 0.00000 | 0.00000 | 0.00000 | 0.16667 | 0.20000 | 0.00000 | 0.00000 | 0.00000 | 0.12500 | 0.16667 |
| 17-098 | 0.00000 | 0.00000 | 0.00000 | 0.00000 | 0.00000 | 0.16667 | 0.16667 | 0.00000 | 0.16667 | 0.11111 | 0.00000 | 0.00000 | 0.30000 | 0.08333 | 0.20000 | 0.00000 | 0.09091 | 0.11111 | 0.00000 | 0.16667 |
| 17-099 | 0.00000 | 0.00000 | 0.00000 | 0.00000 | 0.00000 | 0.00000 | 0.16667 | 0.00000 | 0.08333 | 0.00000 | 0.00000 | 0.08333 | 0.20000 | 0.00000 | 0.10000 | 0.12500 | 0.00000 | 0.00000 | 0.00000 | 0.16667 |
| 17-100 | 0.75000 | 0.00000 | 0.16667 | 0.16667 | 0.25000 | 0.16667 | 0.00000 | 0.16667 | 0.16667 | 0.00000 | 0.00000 | 0.00000 | 0.00000 | 0.00000 | 0.00000 | 0.00000 | 0.09091 | 0.11111 | 0.00000 | 0.00000 |
| 17-101 | 0.75000 | 0.00000 | 0.08333 | 0.16667 | 0.25000 | 0.00000 | 0.08333 | 0.00000 | 0.25000 | 0.00000 | 0.00000 | 0.00000 | 0.00000 | 0.08333 | 0.20000 | 0.00000 | 0.00000 | 0.11111 | 0.00000 | 0.00000 |
| 17-102 | 0.00000 | 0.00000 | 0.08333 | 0.08333 | 0.00000 | 0.16667 | 0.25000 | 0.16667 | 0.41667 | 0.22222 | 0.09091 | 0.08333 | 0.00000 | 0.25000 | 0.20000 | 0.00000 | 0.00000 | 0.00000 | 0.12500 | 0.33333 |
| 17-103 | 0.00000 | 0.66667 | 0.50000 | 0.83333 | 0.66667 | 0.66667 | 0.75000 | 0.66667 | 0.91667 | 0.44444 | 0.45455 | 0.66667 | 0.80000 | 0.41667 | 0.20000 | 0.37500 | 0.72727 | 0.33333 | 0.75000 | 0.83333 |
| 17-104 | 0.00000 | 0.77778 | 0.50000 | 0.75000 | 0.58333 | 0.50000 | 0.75000 | 0.66667 | 0.91667 | 0.66667 | 0.81818 | 0.50000 | 0.80000 | 0.33333 | 0.20000 | 0.50000 | 0.90909 | 0.44444 | 0.62500 | 1.00000 |
| 17-105 | 0.00000 | 0.11111 | 0.00000 | 0.00000 | 0.00000 | 0.16667 | 0.00000 | 0.00000 | 0.00000 | 0.00000 | 0.00000 | 0.16667 | 0.10000 | 0.08333 | 0.00000 | 0.12500 | 0.09091 | 0.11111 | 0.00000 | 0.00000 |
| 17-106 | 0.12500 | 0.00000 | 0.08333 | 0.00000 | 0.00000 | 0.16667 | 0.00000 | 0.00000 | 0.00000 | 0.11111 | 0.36364 | 0.08333 | 0.10000 | 0.00000 | 0.10000 | 0.25000 | 0.09091 | 0.11111 | 0.00000 | 0.00000 |
| 17-107 | 0.00000 | 0.00000 | 0.00000 | 0.00000 | 0.00000 | 0.00000 | 0.00000 | 0.16667 | 0.00000 | 0.00000 | 0.00000 | 0.00000 | 0.00000 | 0.00000 | 0.00000 | 0.00000 | 0.00000 | 0.00000 | 0.00000 | 0.00000 |
| 17-108 | 0.00000 | 0.00000 | 0.08333 | 0.16667 | 0.00000 | 0.00000 | 0.00000 | 0.00000 | 0.00000 | 0.00000 | 0.00000 | 0.00000 | 0.00000 | 0.00000 | 0.00000 | 0.00000 | 0.00000 | 0.00000 | 0.00000 | 0.00000 |
| 17-109 | 0.00000 | 0.00000 | 0.00000 | 0.16667 | 0.00000 | 0.16667 | 0.00000 | 0.00000 | 0.00000 | 0.00000 | 0.00000 | 0.00000 | 0.00000 | 0.16667 | 0.00000 | 0.00000 | 0.00000 | 0.00000 | 0.00000 | 0.00000 |
| 17-110 | 0.00000 | 0.00000 | 0.00000 | 0.00000 | 0.00000 | 0.16667 | 0.00000 | 0.00000 | 0.00000 | 0.00000 | 0.00000 | 0.00000 | 0.00000 | 0.16667 | 0.00000 | 0.00000 | 0.00000 | 0.00000 | 0.00000 | 0.00000 |
| 17-111 | 0.00000 | 0.00000 | 0.00000 | 0.00000 | 0.08333 | 0.16667 | 0.00000 | 0.00000 | 0.16667 | 0.00000 | 0.00000 | 0.00000 | 0.00000 | 0.00000 | 0.00000 | 0.00000 | 0.09091 | 0.00000 | 0.00000 | 0.00000 |
| 17-112 | 0.00000 | 0.00000 | 0.00000 | 0.00000 | 0.16667 | 0.16667 | 0.00000 | 0.00000 | 0.08333 | 0.00000 | 0.00000 | 0.00000 | 0.00000 | 0.00000 | 0.00000 | 0.00000 | 0.00000 | 0.00000 | 0.00000 | 0.00000 |
| 17-113 | 0.12500 | 0.00000 | 0.00000 | 0.00000 | 0.00000 | 0.00000 | 0.00000 | 0.00000 | 0.00000 | 0.00000 | 0.00000 | 0.00000 | 0.00000 | 0.00000 | 0.00000 | 0.00000 | 0.00000 | 0.00000 | 0.00000 | 0.00000 |
| 17-114 | 0.12500 | 0.00000 | 0.00000 | 0.00000 | 0.00000 | 0.00000 | 0.00000 | 0.00000 | 0.00000 | 0.00000 | 0.00000 | 0.00000 | 0.00000 | 0.00000 | 0.00000 | 0.00000 | 0.00000 | 0.00000 | 0.00000 | 0.00000 |
| 17-115 | 0.00000 | 0.11111 | 0.00000 | 0.00000 | 0.08333 | 0.00000 | 0.00000 | 0.00000 | 0.00000 | 0.00000 | 0.00000 | 0.00000 | 0.00000 | 0.00000 | 0.00000 | 0.00000 | 0.00000 | 0.00000 | 0.00000 | 0.00000 |
| 17-116 | 0.00000 | 0.00000 | 0.00000 | 0.00000 | 0.08333 | 0.00000 | 0.00000 | 0.00000 | 0.00000 | 0.00000 | 0.00000 | 0.00000 | 0.00000 | 0.00000 | 0.00000 | 0.00000 | 0.00000 | 0.00000 | 0.00000 | 0.00000 |
| 17-117 | 0.00000 | 0.00000 | 0.08333 | 0.41667 | 0.00000 | 0.50000 | 0.16667 | 0.16667 | 0.00000 | 0.11111 | 0.45455 | 0.08333 | 0.10000 | 0.08333 | 0.00000 | 0.37500 | 0.54545 | 0.11111 | 0.00000 | 0.00000 |
| 17-118 | 0.00000 | 0.00000 | 0.08333 | 0.33333 | 0.08333 | 0.50000 | 0.16667 | 0.16667 | 0.00000 | 0.11111 | 0.36364 | 0.08333 | 0.10000 | 0.08333 | 0.00000 | 0.37500 | 0.54545 | 0.11111 | 0.00000 | 0.00000 |
| 17-119 | 0.00000 | 0.00000 | 0.00000 | 0.00000 | 0.00000 | 0.00000 | 0.00000 | 0.33333 | 0.00000 | 0.00000 | 0.00000 | 0.00000 | 0.00000 | 0.00000 | 0.00000 | 0.00000 | 0.00000 | 0.00000 | 0.00000 | 0.00000 |
| 17-120 | 0.00000 | 0.00000 | 0.00000 | 0.00000 | 0.00000 | 0.00000 | 0.00000 | 0.33333 | 0.00000 | 0.11111 | 0.00000 | 0.00000 | 0.00000 | 0.00000 | 0.00000 | 0.00000 | 0.00000 | 0.00000 | 0.00000 | 0.00000 |
| 17-121 | 0.00000 | 0.00000 | 0.08333 | 0.00000 | 0.00000 | 0.00000 | 0.00000 | 0.00000 | 0.00000 | 0.00000 | 0.00000 | 0.00000 | 0.00000 | 0.00000 | 0.00000 | 0.00000 | 0.00000 | 0.00000 | 0.00000 | 0.00000 |
| 18-001 | 0.00000 | 0.20000 | 0.08333 | 0.33333 | 0.08333 | 0.00000 | 0.00000 | 0.00000 | 0.00000 | 0.00000 | 0.00000 | 0.08333 | 0.00000 | 0.08333 | 0.00000 | 0.00000 | 0.00000 | 0.00000 | 0.11111 | 0.00000 |
| 18-002 | 0.00000 | 0.20000 | 0.25000 | 0.08333 | 0.08333 | 0.16667 | 0.00000 | 0.16667 | 0.00000 | 0.20000 | 0.00000 | 0.00000 | 0.10000 | 0.41667 | 0.00000 | 0.00000 | 0.16667 | 0.10000 | 0.00000 | 0.00000 |
| 18-003 | 0.00000 | 0.10000 | 0.16667 | 0.33333 | 0.16667 | 0.16667 | 0.00000 | 0.00000 | 0.08333 | 0.10000 | 0.08333 | 0.16667 | 0.00000 | 0.50000 | 0.00000 | 0.00000 | 0.25000 | 0.00000 | 0.00000 | 0.16667 |
| 18-004 | 0.00000 | 0.20000 | 0.16667 | 0.25000 | 0.25000 | 0.00000 | 0.16667 | 0.33333 | 0.33333 | 0.10000 | 0.33333 | 0.16667 | 0.10000 | 0.16667 | 0.00000 | 0.00000 | 0.25000 | 0.00000 | 0.11111 | 0.16667 |
| 18-005 | 0.00000 | 0.10000 | 0.00000 | 0.00000 | 0.16667 | 0.16667 | 0.00000 | 0.33333 | 0.50000 | 0.00000 | 0.33333 | 0.33333 | 0.10000 | 0.25000 | 0.10000 | 0.00000 | 0.16667 | 0.00000 | 0.00000 | 0.16667 |
| 18-006 | 0.00000 | 0.20000 | 0.08333 | 0.08333 | 0.00000 | 0.16667 | 0.00000 | 0.00000 | 0.00000 | 0.20000 | 0.00000 | 0.00000 | 0.00000 | 0.08333 | 0.00000 | 0.00000 | 0.16667 | 0.00000 | 0.00000 | 0.00000 |
| 18-007 | 0.00000 | 0.00000 | 0.16667 | 0.08333 | 0.16667 | 0.16667 | 0.00000 | 0.00000 | 0.00000 | 0.00000 | 0.00000 | 0.16667 | 0.00000 | 0.16667 | 0.00000 | 0.00000 | 0.08333 | 0.00000 | 0.00000 | 0.00000 |
| 18-008 | 0.87500 | 0.30000 | 0.33333 | 0.16667 | 0.25000 | 0.16667 | 0.33333 | 0.33333 | 0.41667 | 0.00000 | 0.25000 | 0.58333 | 0.50000 | 0.58333 | 0.00000 | 0.30000 | 0.41667 | 0.20000 | 0.00000 | 0.00000 |
| 18-009 | 0.00000 | 0.10000 | 0.08333 | 0.16667 | 0.00000 | 0.00000 | 0.25000 | 0.33333 | 0.33333 | 0.00000 | 0.33333 | 0.33333 | 0.10000 | 0.08333 | 0.10000 | 0.00000 | 0.08333 | 0.10000 | 0.00000 | 0.50000 |
| 18-010 | 0.00000 | 0.00000 | 0.08333 | 0.08333 | 0.25000 | 0.00000 | 0.08333 | 0.16667 | 0.08333 | 0.00000 | 0.08333 | 0.08333 | 0.10000 | 0.16667 | 0.10000 | 0.00000 | 0.08333 | 0.00000 | 0.00000 | 0.00000 |
| 18-011 | 0.00000 | 0.10000 | 0.16667 | 0.16667 | 0.08333 | 0.16667 | 0.08333 | 0.00000 | 0.16667 | 0.00000 | 0.16667 | 0.08333 | 0.00000 | 0.08333 | 0.00000 | 0.00000 | 0.16667 | 0.10000 | 0.00000 | 0.00000 |
| 18-012 | 0.00000 | 0.10000 | 0.33333 | 0.16667 | 0.08333 | 0.16667 | 0.08333 | 0.33333 | 0.16667 | 0.10000 | 0.16667 | 0.41667 | 0.00000 | 0.41667 | 0.10000 | 0.10000 | 0.25000 | 0.10000 | 0.00000 | 0.00000 |
| 18-013 | 0.00000 | 0.10000 | 0.08333 | 0.16667 | 0.08333 | 0.00000 | 0.00000 | 0.33333 | 0.25000 | 0.10000 | 0.16667 | 0.25000 | 0.10000 | 0.41667 | 0.00000 | 0.00000 | 0.16667 | 0.00000 | 0.00000 | 0.16667 |
| 18-014 | 0.12500 | 0.30000 | 0.25000 | 0.25000 | 0.25000 | 0.16667 | 0.16667 | 0.00000 | 0.00000 | 0.20000 | 0.16667 | 0.16667 | 0.00000 | 0.00000 | 0.30000 | 0.10000 | 0.00000 | 0.10000 | 0.44444 | 0.66667 |
| 18-015 | 0.00000 | 0.20000 | 0.16667 | 0.25000 | 0.16667 | 0.16667 | 0.16667 | 0.00000 | 0.00000 | 0.00000 | 0.00000 | 0.08333 | 0.00000 | 0.08333 | 0.00000 | 0.00000 | 0.25000 | 0.10000 | 0.00000 | 0.33333 |
| 18-016 | 0.00000 | 0.40000 | 0.16667 | 0.25000 | 0.16667 | 0.00000 | 0.00000 | 0.00000 | 0.00000 | 0.30000 | 0.08333 | 0.08333 | 0.00000 | 0.41667 | 0.00000 | 0.00000 | 0.00000 | 0.00000 | 0.00000 | 0.16667 |
| 18-017 | 0.00000 | 0.00000 | 0.08333 | 0.25000 | 0.08333 | 0.00000 | 0.00000 | 0.00000 | 0.00000 | 0.10000 | 0.00000 | 0.00000 | 0.20000 | 0.08333 | 0.10000 | 0.00000 | 0.08333 | 0.00000 | 0.11111 | 0.00000 |
| 18-018 | 0.00000 | 0.10000 | 0.25000 | 0.08333 | 0.08333 | 0.00000 | 0.00000 | 0.00000 | 0.00000 | 0.00000 | 0.00000 | 0.08333 | 0.00000 | 0.00000 | 0.20000 | 0.00000 | 0.00000 | 0.10000 | 0.00000 | 0.50000 |
| 18-019 | 0.00000 | 0.50000 | 0.16667 | 0.16667 | 0.25000 | 0.00000 | 0.00000 | 0.00000 | 0.00000 | 0.00000 | 0.08333 | 0.16667 | 0.10000 | 0.00000 | 0.10000 | 0.00000 | 0.16667 | 0.10000 | 0.00000 | 0.50000 |
| 18-020 | 0.00000 | 0.10000 | 0.08333 | 0.16667 | 0.00000 | 0.00000 | 0.00000 | 0.00000 | 0.16667 | 0.00000 | 0.00000 | 0.00000 | 0.00000 | 0.00000 | 0.00000 | 0.00000 | 0.16667 | 0.00000 | 0.11111 | 0.00000 |
| 18-021 | 0.00000 | 0.10000 | 0.16667 | 0.41667 | 0.08333 | 0.00000 | 0.00000 | 0.16667 | 0.08333 | 0.30000 | 0.00000 | 0.08333 | 0.00000 | 0.00000 | 0.00000 | 0.00000 | 0.08333 | 0.10000 | 0.11111 | 0.00000 |
| 18-022 | 0.87500 | 0.20000 | 0.08333 | 0.16667 | 0.16667 | 0.16667 | 0.00000 | 0.00000 | 0.00000 | 0.20000 | 0.00000 | 0.08333 | 0.00000 | 0.00000 | 0.00000 | 0.00000 | 0.00000 | 0.00000 | 0.00000 | 0.16667 |
| 18-023 | 0.87500 | 0.40000 | 0.66667 | 0.33333 | 0.58333 | 0.33333 | 0.41667 | 0.50000 | 0.33333 | 0.20000 | 0.58333 | 0.75000 | 0.80000 | 0.83333 | 0.10000 | 0.50000 | 0.75000 | 0.50000 | 0.33333 | 0.00000 |
| 18-024 | 0.12500 | 0.20000 | 0.00000 | 0.16667 | 0.16667 | 0.00000 | 0.00000 | 0.16667 | 0.00000 | 0.40000 | 0.08333 | 0.00000 | 0.00000 | 0.00000 | 0.00000 | 0.00000 | 0.08333 | 0.00000 | 0.00000 | 0.00000 |
| 18-025 | 0.12500 | 0.10000 | 0.00000 | 0.33333 | 0.16667 | 0.00000 | 0.00000 | 0.00000 | 0.00000 | 0.10000 | 0.16667 | 0.08333 | 0.10000 | 0.25000 | 0.10000 | 0.00000 | 0.50000 | 0.00000 | 0.11111 | 0.00000 |
| 18-026 | 0.00000 | 0.20000 | 0.00000 | 0.33333 | 0.16667 | 0.33333 | 0.00000 | 0.16667 | 0.00000 | 0.00000 | 0.08333 | 0.00000 | 0.10000 | 0.08333 | 0.00000 | 0.00000 | 0.16667 | 0.00000 | 0.11111 | 0.16667 |
| 18-027 | 0.12500 | 0.00000 | 0.08333 | 0.33333 | 0.16667 | 0.00000 | 0.08333 | 0.16667 | 0.00000 | 0.10000 | 0.25000 | 0.16667 | 0.00000 | 0.25000 | 0.00000 | 0.00000 | 0.16667 | 0.30000 | 0.00000 | 0.00000 |
| 18-028 | 0.25000 | 0.50000 | 0.58333 | 0.41667 | 0.58333 | 0.33333 | 0.41667 | 0.50000 | 0.25000 | 0.30000 | 0.41667 | 0.41667 | 0.60000 | 0.41667 | 0.90000 | 0.60000 | 0.16667 | 0.60000 | 0.66667 | 0.66667 |
| 18-029 | 0.00000 | 0.00000 | 0.08333 | 0.25000 | 0.33333 | 0.16667 | 0.00000 | 0.16667 | 0.16667 | 0.20000 | 0.33333 | 0.16667 | 0.00000 | 0.08333 | 0.00000 | 0.00000 | 0.16667 | 0.00000 | 0.33333 | 0.50000 |
| 18-030 | 0.00000 | 0.10000 | 0.00000 | 0.25000 | 0.08333 | 0.16667 | 0.00000 | 0.00000 | 0.08333 | 0.10000 | 0.33333 | 0.08333 | 0.00000 | 0.16667 | 0.00000 | 0.10000 | 0.08333 | 0.10000 | 0.11111 | 0.33333 |
| 18-031 | 0.12500 | 0.20000 | 0.16667 | 0.41667 | 0.08333 | 0.16667 | 0.00000 | 0.16667 | 0.08333 | 0.20000 | 0.25000 | 0.16667 | 0.00000 | 0.33333 | 0.00000 | 0.10000 | 0.16667 | 0.30000 | 0.00000 | 0.16667 |
| 18-032 | 0.25000 | 0.20000 | 0.25000 | 0.25000 | 0.08333 | 0.00000 | 0.00000 | 0.33333 | 0.08333 | 0.20000 | 0.00000 | 0.08333 | 0.00000 | 0.25000 | 0.00000 | 0.00000 | 0.25000 | 0.10000 | 0.00000 | 0.00000 |
| 18-033 | 0.00000 | 0.10000 | 0.08333 | 0.16667 | 0.08333 | 0.00000 | 0.00000 | 0.16667 | 0.08333 | 0.00000 | 0.08333 | 0.25000 | 0.20000 | 0.00000 | 0.10000 | 0.20000 | 0.08333 | 0.10000 | 0.00000 | 0.33333 |
| 18-034 | 0.00000 | 0.20000 | 0.50000 | 0.08333 | 0.50000 | 0.16667 | 0.33333 | 0.00000 | 0.33333 | 0.20000 | 0.58333 | 0.50000 | 0.40000 | 0.58333 | 0.00000 | 0.30000 | 0.50000 | 0.30000 | 0.22222 | 0.33333 |
| 18-035 | 0.00000 | 0.40000 | 0.66667 | 0.25000 | 0.33333 | 0.16667 | 0.33333 | 0.16667 | 0.25000 | 0.20000 | 0.58333 | 0.58333 | 0.50000 | 0.58333 | 0.10000 | 0.50000 | 0.33333 | 0.40000 | 0.44444 | 0.50000 |
| 18-036 | 0.12500 | 0.30000 | 0.08333 | 0.33333 | 0.25000 | 0.00000 | 0.00000 | 0.00000 | 0.00000 | 0.00000 | 0.08333 | 0.00000 | 0.10000 | 0.08333 | 0.30000 | 0.10000 | 0.16667 | 0.00000 | 0.11111 | 0.66667 |
| 18-037 | 0.87500 | 0.00000 | 0.08333 | 0.25000 | 0.00000 | 0.00000 | 0.00000 | 0.00000 | 0.00000 | 0.00000 | 0.00000 | 0.00000 | 0.00000 | 0.08333 | 0.00000 | 0.00000 | 0.00000 | 0.00000 | 0.00000 | 0.00000 |
| 18-038 | 0.00000 | 0.00000 | 0.00000 | 0.00000 | 0.00000 | 0.00000 | 0.00000 | 0.00000 | 0.00000 | 0.00000 | 0.08333 | 0.00000 | 0.00000 | 0.00000 | 0.00000 | 0.10000 | 0.00000 | 0.00000 | 0.00000 | 0.00000 |
| 18-039 | 0.87500 | 0.00000 | 0.00000 | 0.16667 | 0.16667 | 0.16667 | 0.00000 | 0.00000 | 0.00000 | 0.00000 | 0.16667 | 0.00000 | 0.00000 | 0.08333 | 0.00000 | 0.00000 | 0.08333 | 0.00000 | 0.00000 | 0.00000 |
| 18-040 | 0.00000 | 0.00000 | 0.00000 | 0.16667 | 0.00000 | 0.00000 | 0.00000 | 0.00000 | 0.00000 | 0.00000 | 0.16667 | 0.00000 | 0.00000 | 0.00000 | 0.00000 | 0.10000 | 0.00000 | 0.00000 | 0.00000 | 0.00000 |
| 18-041 | 0.87500 | 0.40000 | 0.66667 | 0.25000 | 0.50000 | 0.16667 | 0.25000 | 0.66667 | 0.25000 | 0.30000 | 0.41667 | 0.50000 | 0.40000 | 0.33333 | 0.00000 | 0.30000 | 0.41667 | 0.30000 | 0.44444 | 0.50000 |
| 18-042 | 0.37500 | 0.30000 | 0.50000 | 0.16667 | 0.16667 | 0.00000 | 0.08333 | 0.50000 | 0.16667 | 0.30000 | 0.25000 | 0.25000 | 0.20000 | 0.33333 | 0.10000 | 0.40000 | 0.16667 | 0.20000 | 0.33333 | 0.33333 |
| 18-043 | 0.00000 | 0.20000 | 0.25000 | 0.16667 | 0.08333 | 0.16667 | 0.08333 | 0.16667 | 0.08333 | 0.10000 | 0.16667 | 0.50000 | 0.50000 | 0.25000 | 0.10000 | 0.30000 | 0.33333 | 0.00000 | 0.11111 | 0.00000 |
| 18-044 | 0.00000 | 0.40000 | 0.16667 | 0.25000 | 0.16667 | 0.16667 | 0.33333 | 0.00000 | 0.16667 | 0.30000 | 0.08333 | 0.25000 | 0.30000 | 0.16667 | 0.30000 | 0.50000 | 0.08333 | 0.40000 | 0.66667 | 0.66667 |
| 18-045 | 0.00000 | 0.20000 | 0.16667 | 0.41667 | 0.16667 | 0.16667 | 0.33333 | 0.00000 | 0.16667 | 0.40000 | 0.16667 | 0.25000 | 0.10000 | 0.16667 | 0.30000 | 0.40000 | 0.08333 | 0.40000 | 0.66667 | 0.66667 |
| 18-046 | 0.00000 | 0.20000 | 0.08333 | 0.08333 | 0.00000 | 0.00000 | 0.00000 | 0.00000 | 0.00000 | 0.00000 | 0.00000 | 0.00000 | 0.00000 | 0.00000 | 0.00000 | 0.00000 | 0.00000 | 0.00000 | 0.22222 | 0.00000 |
| 18-047 | 0.12500 | 0.00000 | 0.00000 | 0.00000 | 0.00000 | 0.00000 | 0.08333 | 0.00000 | 0.08333 | 0.10000 | 0.00000 | 0.08333 | 0.00000 | 0.08333 | 0.00000 | 0.00000 | 0.00000 | 0.00000 | 0.00000 | 0.00000 |
| 18-048 | 0.00000 | 0.00000 | 0.00000 | 0.00000 | 0.00000 | 0.16667 | 0.08333 | 0.00000 | 0.00000 | 0.00000 | 0.00000 | 0.08333 | 0.00000 | 0.08333 | 0.00000 | 0.00000 | 0.00000 | 0.00000 | 0.00000 | 0.00000 |
| 18-049 | 0.00000 | 0.00000 | 0.00000 | 0.08333 | 0.00000 | 0.00000 | 0.00000 | 0.00000 | 0.00000 | 0.00000 | 0.00000 | 0.00000 | 0.00000 | 0.00000 | 0.00000 | 0.10000 | 0.00000 | 0.00000 | 0.00000 | 0.00000 |
| 18-050 | 0.12500 | 0.00000 | 0.00000 | 0.00000 | 0.25000 | 0.00000 | 0.00000 | 0.00000 | 0.08333 | 0.00000 | 0.00000 | 0.08333 | 0.00000 | 0.00000 | 0.00000 | 0.00000 | 0.00000 | 0.00000 | 0.00000 | 0.16667 |
| 18-051 | 0.12500 | 0.30000 | 0.25000 | 0.41667 | 0.33333 | 0.16667 | 0.08333 | 0.00000 | 0.00000 | 0.20000 | 0.16667 | 0.25000 | 0.00000 | 0.00000 | 0.90000 | 0.10000 | 0.00000 | 0.40000 | 0.33333 | 0.50000 |
| 18-052 | 0.00000 | 0.40000 | 0.25000 | 0.33333 | 0.16667 | 0.33333 | 0.16667 | 0.16667 | 0.00000 | 0.30000 | 0.16667 | 0.16667 | 0.10000 | 0.00000 | 0.60000 | 0.20000 | 0.00000 | 0.40000 | 0.66667 | 0.50000 |
| 18-053 | 0.00000 | 0.40000 | 0.00000 | 0.08333 | 0.25000 | 0.00000 | 0.08333 | 0.16667 | 0.08333 | 0.10000 | 0.00000 | 0.16667 | 0.20000 | 0.00000 | 0.70000 | 0.10000 | 0.00000 | 0.00000 | 0.11111 | 0.66667 |
| 18-054 | 0.00000 | 0.10000 | 0.00000 | 0.08333 | 0.08333 | 0.00000 | 0.08333 | 0.16667 | 0.08333 | 0.00000 | 0.08333 | 0.08333 | 0.10000 | 0.00000 | 0.00000 | 0.00000 | 0.00000 | 0.00000 | 0.00000 | 0.50000 |
| 18-055 | 0.00000 | 0.00000 | 0.00000 | 0.33333 | 0.00000 | 0.00000 | 0.00000 | 0.00000 | 0.00000 | 0.10000 | 0.08333 | 0.08333 | 0.00000 | 0.00000 | 0.00000 | 0.00000 | 0.08333 | 0.00000 | 0.22222 | 0.33333 |
| 18-056 | 0.00000 | 0.00000 | 0.00000 | 0.08333 | 0.00000 | 0.00000 | 0.00000 | 0.00000 | 0.00000 | 0.00000 | 0.00000 | 0.00000 | 0.00000 | 0.00000 | 0.00000 | 0.00000 | 0.00000 | 0.00000 | 0.00000 | 0.00000 |
| 18-057 | 0.00000 | 0.00000 | 0.00000 | 0.00000 | 0.08333 | 0.00000 | 0.00000 | 0.00000 | 0.00000 | 0.00000 | 0.00000 | 0.00000 | 0.00000 | 0.00000 | 0.00000 | 0.00000 | 0.00000 | 0.00000 | 0.00000 | 0.33333 |
| 18-058 | 0.00000 | 0.00000 | 0.00000 | 0.16667 | 0.08333 | 0.00000 | 0.00000 | 0.00000 | 0.00000 | 0.10000 | 0.08333 | 0.00000 | 0.00000 | 0.00000 | 0.00000 | 0.00000 | 0.00000 | 0.00000 | 0.11111 | 0.33333 |
| 18-059 | 0.00000 | 0.00000 | 0.00000 | 0.08333 | 0.00000 | 0.00000 | 0.00000 | 0.00000 | 0.00000 | 0.00000 | 0.00000 | 0.00000 | 0.00000 | 0.00000 | 0.00000 | 0.00000 | 0.00000 | 0.00000 | 0.00000 | 0.00000 |
| 18-060 | 0.87500 | 0.00000 | 0.08333 | 0.16667 | 0.16667 | 0.00000 | 0.00000 | 0.00000 | 0.00000 | 0.00000 | 0.08333 | 0.08333 | 0.00000 | 0.00000 | 0.00000 | 0.00000 | 0.08333 | 0.00000 | 0.00000 | 0.16667 |
| 18-061 | 0.00000 | 0.00000 | 0.00000 | 0.00000 | 0.00000 | 0.16667 | 0.08333 | 0.00000 | 0.00000 | 0.00000 | 0.00000 | 0.00000 | 0.00000 | 0.00000 | 0.00000 | 0.00000 | 0.08333 | 0.10000 | 0.00000 | 0.33333 |
| 18-062 | 0.12500 | 0.10000 | 0.08333 | 0.25000 | 0.16667 | 0.16667 | 0.08333 | 0.00000 | 0.00000 | 0.00000 | 0.08333 | 0.08333 | 0.00000 | 0.00000 | 0.00000 | 0.00000 | 0.00000 | 0.00000 | 0.00000 | 0.00000 |
| 18-063 | 0.00000 | 0.10000 | 0.08333 | 0.00000 | 0.00000 | 0.00000 | 0.00000 | 0.00000 | 0.00000 | 0.00000 | 0.08333 | 0.00000 | 0.00000 | 0.00000 | 0.00000 | 0.00000 | 0.00000 | 0.00000 | 0.00000 | 0.00000 |
| 18-064 | 0.12500 | 0.10000 | 0.16667 | 0.08333 | 0.00000 | 0.00000 | 0.00000 | 0.00000 | 0.00000 | 0.00000 | 0.00000 | 0.00000 | 0.00000 | 0.00000 | 0.00000 | 0.00000 | 0.08333 | 0.10000 | 0.11111 | 0.00000 |
| 18-065 | 0.00000 | 0.00000 | 0.16667 | 0.08333 | 0.08333 | 0.00000 | 0.00000 | 0.00000 | 0.00000 | 0.10000 | 0.00000 | 0.00000 | 0.00000 | 0.00000 | 0.10000 | 0.00000 | 0.08333 | 0.10000 | 0.00000 | 0.00000 |
| 18-066 | 0.00000 | 0.20000 | 0.08333 | 0.00000 | 0.00000 | 0.00000 | 0.00000 | 0.00000 | 0.00000 | 0.00000 | 0.08333 | 0.00000 | 0.00000 | 0.00000 | 0.10000 | 0.00000 | 0.08333 | 0.10000 | 0.22222 | 0.00000 |
| 18-067 | 0.00000 | 0.00000 | 0.00000 | 0.08333 | 0.00000 | 0.00000 | 0.00000 | 0.00000 | 0.00000 | 0.00000 | 0.08333 | 0.00000 | 0.00000 | 0.00000 | 0.00000 | 0.00000 | 0.08333 | 0.00000 | 0.11111 | 0.00000 |
| 18-068 | 0.00000 | 0.00000 | 0.08333 | 0.00000 | 0.00000 | 0.00000 | 0.00000 | 0.00000 | 0.00000 | 0.00000 | 0.00000 | 0.00000 | 0.00000 | 0.00000 | 0.00000 | 0.00000 | 0.00000 | 0.00000 | 0.00000 | 0.00000 |
| 18-069 | 0.00000 | 0.10000 | 0.00000 | 0.08333 | 0.08333 | 0.00000 | 0.00000 | 0.00000 | 0.00000 | 0.00000 | 0.00000 | 0.08333 | 0.00000 | 0.00000 | 0.00000 | 0.00000 | 0.08333 | 0.00000 | 0.00000 | 0.00000 |
| 18-070 | 0.00000 | 0.30000 | 0.16667 | 0.41667 | 0.16667 | 0.00000 | 0.00000 | 0.66667 | 0.25000 | 0.00000 | 0.25000 | 0.00000 | 0.10000 | 0.00000 | 0.20000 | 0.10000 | 0.08333 | 0.30000 | 0.00000 | 0.33333 |
| 18-071 | 0.37500 | 0.10000 | 0.08333 | 0.25000 | 0.25000 | 0.00000 | 0.00000 | 0.16667 | 0.00000 | 0.00000 | 0.08333 | 0.08333 | 0.00000 | 0.08333 | 0.20000 | 0.00000 | 0.08333 | 0.00000 | 0.22222 | 0.00000 |
| 18-072 | 0.00000 | 0.00000 | 0.08333 | 0.08333 | 0.00000 | 0.00000 | 0.00000 | 0.00000 | 0.00000 | 0.10000 | 0.00000 | 0.08333 | 0.00000 | 0.00000 | 0.00000 | 0.00000 | 0.16667 | 0.00000 | 0.22222 | 0.00000 |
| 18-073 | 0.12500 | 0.00000 | 0.00000 | 0.08333 | 0.00000 | 0.00000 | 0.00000 | 0.00000 | 0.00000 | 0.20000 | 0.00000 | 0.00000 | 0.00000 | 0.00000 | 0.00000 | 0.00000 | 0.00000 | 0.00000 | 0.00000 | 0.00000 |
| 18-074 | 0.00000 | 0.00000 | 0.00000 | 0.00000 | 0.08333 | 0.00000 | 0.00000 | 0.16667 | 0.00000 | 0.00000 | 0.00000 | 0.00000 | 0.00000 | 0.00000 | 0.00000 | 0.00000 | 0.00000 | 0.00000 | 0.00000 | 0.16667 |
| 18-075 | 0.87500 | 0.00000 | 0.16667 | 0.08333 | 0.25000 | 0.00000 | 0.00000 | 0.33333 | 0.00000 | 0.20000 | 0.33333 | 0.08333 | 0.00000 | 0.00000 | 0.00000 | 0.10000 | 0.08333 | 0.10000 | 0.11111 | 0.16667 |
| 18-076 | 0.87500 | 0.10000 | 0.08333 | 0.08333 | 0.16667 | 0.00000 | 0.00000 | 0.33333 | 0.00000 | 0.30000 | 0.25000 | 0.08333 | 0.00000 | 0.00000 | 0.00000 | 0.20000 | 0.00000 | 0.10000 | 0.11111 | 0.00000 |
| 18-077 | 0.00000 | 0.20000 | 0.00000 | 0.00000 | 0.25000 | 0.33333 | 0.00000 | 0.16667 | 0.08333 | 0.00000 | 0.16667 | 0.00000 | 0.00000 | 0.00000 | 0.00000 | 0.00000 | 0.00000 | 0.00000 | 0.00000 | 0.00000 |
| 18-078 | 0.00000 | 0.20000 | 0.08333 | 0.16667 | 0.08333 | 0.16667 | 0.00000 | 0.00000 | 0.08333 | 0.00000 | 0.00000 | 0.00000 | 0.00000 | 0.00000 | 0.10000 | 0.00000 | 0.00000 | 0.00000 | 0.11111 | 0.16667 |
| 18-079 | 0.12500 | 0.20000 | 0.16667 | 0.08333 | 0.00000 | 0.16667 | 0.00000 | 0.16667 | 0.00000 | 0.10000 | 0.00000 | 0.00000 | 0.00000 | 0.08333 | 0.00000 | 0.00000 | 0.08333 | 0.10000 | 0.00000 | 0.16667 |
| 18-080 | 0.00000 | 0.00000 | 0.00000 | 0.08333 | 0.08333 | 0.00000 | 0.08333 | 0.00000 | 0.00000 | 0.20000 | 0.00000 | 0.00000 | 0.00000 | 0.00000 | 0.00000 | 0.00000 | 0.00000 | 0.00000 | 0.00000 | 0.00000 |
| 18-081 | 0.00000 | 0.00000 | 0.00000 | 0.00000 | 0.00000 | 0.00000 | 0.00000 | 0.00000 | 0.00000 | 0.00000 | 0.08333 | 0.00000 | 0.00000 | 0.00000 | 0.00000 | 0.00000 | 0.00000 | 0.00000 | 0.00000 | 0.16667 |
| 18-082 | 0.00000 | 0.00000 | 0.00000 | 0.00000 | 0.00000 | 0.16667 | 0.00000 | 0.00000 | 0.00000 | 0.00000 | 0.00000 | 0.00000 | 0.00000 | 0.00000 | 0.00000 | 0.00000 | 0.00000 | 0.00000 | 0.00000 | 0.00000 |
| 18-083 | 0.00000 | 0.00000 | 0.00000 | 0.00000 | 0.08333 | 0.00000 | 0.00000 | 0.00000 | 0.00000 | 0.10000 | 0.00000 | 0.08333 | 0.00000 | 0.00000 | 0.00000 | 0.00000 | 0.08333 | 0.00000 | 0.11111 | 0.00000 |
| 18-084 | 0.00000 | 0.00000 | 0.08333 | 0.16667 | 0.08333 | 0.00000 | 0.08333 | 0.00000 | 0.08333 | 0.20000 | 0.00000 | 0.16667 | 0.00000 | 0.00000 | 0.00000 | 0.00000 | 0.08333 | 0.10000 | 0.00000 | 0.00000 |
| 18-085 | 0.00000 | 0.00000 | 0.08333 | 0.08333 | 0.00000 | 0.00000 | 0.08333 | 0.00000 | 0.00000 | 0.00000 | 0.08333 | 0.16667 | 0.00000 | 0.00000 | 0.00000 | 0.00000 | 0.16667 | 0.10000 | 0.11111 | 0.00000 |
| 18-086 | 0.00000 | 0.00000 | 0.00000 | 0.08333 | 0.08333 | 0.00000 | 0.00000 | 0.16667 | 0.00000 | 0.00000 | 0.00000 | 0.00000 | 0.00000 | 0.00000 | 0.00000 | 0.00000 | 0.08333 | 0.00000 | 0.11111 | 0.00000 |
| 18-087 | 0.00000 | 0.00000 | 0.08333 | 0.16667 | 0.08333 | 0.00000 | 0.00000 | 0.16667 | 0.00000 | 0.20000 | 0.08333 | 0.00000 | 0.30000 | 0.00000 | 0.00000 | 0.20000 | 0.08333 | 0.00000 | 0.22222 | 0.16667 |
| 18-088 | 0.12500 | 0.20000 | 0.00000 | 0.25000 | 0.08333 | 0.16667 | 0.00000 | 0.00000 | 0.00000 | 0.00000 | 0.00000 | 0.00000 | 0.00000 | 0.00000 | 0.00000 | 0.00000 | 0.00000 | 0.00000 | 0.22222 | 0.33333 |
| 18-089 | 0.12500 | 0.20000 | 0.00000 | 0.00000 | 0.00000 | 0.00000 | 0.00000 | 0.16667 | 0.00000 | 0.10000 | 0.08333 | 0.08333 | 0.10000 | 0.00000 | 0.00000 | 0.00000 | 0.00000 | 0.00000 | 0.33333 | 0.16667 |
| 18-090 | 0.00000 | 0.10000 | 0.00000 | 0.00000 | 0.00000 | 0.00000 | 0.00000 | 0.00000 | 0.00000 | 0.10000 | 0.08333 | 0.00000 | 0.00000 | 0.00000 | 0.00000 | 0.00000 | 0.00000 | 0.00000 | 0.00000 | 0.00000 |
| 18-091 | 0.00000 | 0.00000 | 0.00000 | 0.00000 | 0.00000 | 0.00000 | 0.00000 | 0.00000 | 0.00000 | 0.00000 | 0.08333 | 0.00000 | 0.00000 | 0.00000 | 0.00000 | 0.00000 | 0.00000 | 0.00000 | 0.00000 | 0.00000 |
| 18-092 | 0.00000 | 0.00000 | 0.00000 | 0.00000 | 0.00000 | 0.00000 | 0.00000 | 0.00000 | 0.00000 | 0.00000 | 0.08333 | 0.00000 | 0.00000 | 0.00000 | 0.00000 | 0.00000 | 0.00000 | 0.00000 | 0.00000 | 0.00000 |
| 18-093 | 0.00000 | 0.00000 | 0.00000 | 0.08333 | 0.08333 | 0.00000 | 0.00000 | 0.00000 | 0.00000 | 0.00000 | 0.00000 | 0.00000 | 0.00000 | 0.00000 | 0.00000 | 0.00000 | 0.00000 | 0.00000 | 0.11111 | 0.16667 |
| 18-094 | 0.00000 | 0.00000 | 0.00000 | 0.08333 | 0.00000 | 0.00000 | 0.00000 | 0.00000 | 0.00000 | 0.00000 | 0.00000 | 0.00000 | 0.00000 | 0.00000 | 0.00000 | 0.00000 | 0.00000 | 0.00000 | 0.00000 | 0.00000 |
| 18-095 | 0.00000 | 0.00000 | 0.00000 | 0.08333 | 0.08333 | 0.00000 | 0.00000 | 0.16667 | 0.00000 | 0.00000 | 0.08333 | 0.00000 | 0.00000 | 0.00000 | 0.00000 | 0.10000 | 0.00000 | 0.00000 | 0.11111 | 0.00000 |
| 18-096 | 0.00000 | 0.00000 | 0.00000 | 0.00000 | 0.00000 | 0.00000 | 0.00000 | 0.00000 | 0.00000 | 0.00000 | 0.00000 | 0.00000 | 0.00000 | 0.00000 | 0.00000 | 0.10000 | 0.00000 | 0.00000 | 0.11111 | 0.00000 |
| 18-097 | 0.12500 | 0.00000 | 0.00000 | 0.08333 | 0.08333 | 0.00000 | 0.00000 | 0.00000 | 0.00000 | 0.00000 | 0.00000 | 0.00000 | 0.00000 | 0.00000 | 0.00000 | 0.00000 | 0.08333 | 0.00000 | 0.11111 | 0.00000 |
| 18-098 | 0.12500 | 0.00000 | 0.00000 | 0.00000 | 0.08333 | 0.00000 | 0.00000 | 0.00000 | 0.00000 | 0.00000 | 0.00000 | 0.00000 | 0.00000 | 0.00000 | 0.00000 | 0.00000 | 0.00000 | 0.00000 | 0.00000 | 0.00000 |
| 18-099 | 0.00000 | 0.10000 | 0.00000 | 0.08333 | 0.16667 | 0.16667 | 0.00000 | 0.00000 | 0.00000 | 0.00000 | 0.08333 | 0.00000 | 0.00000 | 0.00000 | 0.00000 | 0.00000 | 0.08333 | 0.00000 | 0.11111 | 0.00000 |
| 18-100 | 0.00000 | 0.00000 | 0.00000 | 0.00000 | 0.00000 | 0.00000 | 0.00000 | 0.00000 | 0.00000 | 0.00000 | 0.08333 | 0.00000 | 0.00000 | 0.00000 | 0.00000 | 0.00000 | 0.00000 | 0.00000 | 0.11111 | 0.00000 |
| 18-101 | 0.00000 | 0.00000 | 0.00000 | 0.00000 | 0.00000 | 0.00000 | 0.00000 | 0.00000 | 0.00000 | 0.00000 | 0.00000 | 0.00000 | 0.00000 | 0.00000 | 0.00000 | 0.10000 | 0.00000 | 0.00000 | 0.00000 | 0.00000 |
| 18-102 | 0.00000 | 0.30000 | 0.08333 | 0.16667 | 0.08333 | 0.16667 | 0.08333 | 0.00000 | 0.00000 | 0.00000 | 0.08333 | 0.00000 | 0.00000 | 0.00000 | 0.00000 | 0.00000 | 0.08333 | 0.00000 | 0.00000 | 0.16667 |
| 18-103 | 0.00000 | 0.00000 | 0.00000 | 0.00000 | 0.00000 | 0.16667 | 0.08333 | 0.00000 | 0.00000 | 0.00000 | 0.16667 | 0.00000 | 0.00000 | 0.00000 | 0.00000 | 0.10000 | 0.08333 | 0.00000 | 0.00000 | 0.00000 |
| 18-104 | 0.00000 | 0.30000 | 0.08333 | 0.16667 | 0.08333 | 0.16667 | 0.00000 | 0.00000 | 0.00000 | 0.00000 | 0.00000 | 0.00000 | 0.00000 | 0.00000 | 0.00000 | 0.00000 | 0.16667 | 0.00000 | 0.00000 | 0.16667 |
| 18-105 | 0.00000 | 0.00000 | 0.00000 | 0.08333 | 0.00000 | 0.00000 | 0.00000 | 0.00000 | 0.00000 | 0.00000 | 0.00000 | 0.00000 | 0.00000 | 0.00000 | 0.00000 | 0.00000 | 0.00000 | 0.00000 | 0.00000 | 0.00000 |
| 18-106 | 0.00000 | 0.00000 | 0.00000 | 0.00000 | 0.00000 | 0.00000 | 0.00000 | 0.16667 | 0.00000 | 0.00000 | 0.08333 | 0.00000 | 0.00000 | 0.00000 | 0.00000 | 0.10000 | 0.00000 | 0.00000 | 0.11111 | 0.00000 |
| 18-107 | 0.00000 | 0.00000 | 0.00000 | 0.08333 | 0.00000 | 0.00000 | 0.00000 | 0.16667 | 0.00000 | 0.00000 | 0.08333 | 0.00000 | 0.00000 | 0.00000 | 0.00000 | 0.10000 | 0.00000 | 0.00000 | 0.00000 | 0.00000 |
| 18-108 | 0.00000 | 0.10000 | 0.08333 | 0.08333 | 0.08333 | 0.16667 | 0.00000 | 0.16667 | 0.00000 | 0.20000 | 0.33333 | 0.08333 | 0.00000 | 0.00000 | 0.00000 | 0.00000 | 0.08333 | 0.00000 | 0.00000 | 0.00000 |
| 18-109 | 0.12500 | 0.10000 | 0.08333 | 0.00000 | 0.08333 | 0.00000 | 0.00000 | 0.16667 | 0.00000 | 0.20000 | 0.08333 | 0.08333 | 0.00000 | 0.00000 | 0.00000 | 0.00000 | 0.00000 | 0.00000 | 0.11111 | 0.00000 |
| 18-110 | 0.12500 | 0.00000 | 0.00000 | 0.00000 | 0.00000 | 0.00000 | 0.00000 | 0.00000 | 0.00000 | 0.00000 | 0.00000 | 0.00000 | 0.00000 | 0.00000 | 0.00000 | 0.00000 | 0.00000 | 0.00000 | 0.00000 | 0.00000 |
| 18-111 | 0.00000 | 0.30000 | 0.33333 | 0.08333 | 0.41667 | 0.16667 | 0.50000 | 0.16667 | 0.25000 | 0.20000 | 0.16667 | 0.41667 | 0.40000 | 0.00000 | 0.60000 | 0.10000 | 0.33333 | 0.50000 | 0.44444 | 0.00000 |
| 18-112 | 0.00000 | 0.30000 | 0.25000 | 0.08333 | 0.33333 | 0.16667 | 0.41667 | 0.00000 | 0.16667 | 0.00000 | 0.16667 | 0.41667 | 0.20000 | 0.00000 | 0.50000 | 0.10000 | 0.33333 | 0.50000 | 0.22222 | 0.00000 |
| 18-113 | 0.00000 | 0.00000 | 0.00000 | 0.00000 | 0.00000 | 0.00000 | 0.00000 | 0.00000 | 0.00000 | 0.00000 | 0.00000 | 0.00000 | 0.00000 | 0.00000 | 0.00000 | 0.00000 | 0.08333 | 0.00000 | 0.00000 | 0.00000 |
| 18-114 | 0.00000 | 0.30000 | 0.16667 | 0.00000 | 0.00000 | 0.33333 | 0.08333 | 0.00000 | 0.25000 | 0.00000 | 0.00000 | 0.00000 | 0.00000 | 0.00000 | 0.00000 | 0.10000 | 0.08333 | 0.00000 | 0.11111 | 0.16667 |
| 18-115 | 0.00000 | 0.00000 | 0.08333 | 0.00000 | 0.00000 | 0.00000 | 0.08333 | 0.00000 | 0.00000 | 0.00000 | 0.00000 | 0.00000 | 0.00000 | 0.00000 | 0.00000 | 0.00000 | 0.08333 | 0.00000 | 0.11111 | 0.00000 |
| 18-116 | 0.12500 | 0.00000 | 0.00000 | 0.00000 | 0.00000 | 0.00000 | 0.00000 | 0.00000 | 0.00000 | 0.00000 | 0.00000 | 0.00000 | 0.00000 | 0.00000 | 0.00000 | 0.10000 | 0.00000 | 0.10000 | 0.00000 | 0.00000 |
| 18-117 | 0.00000 | 0.10000 | 0.00000 | 0.00000 | 0.00000 | 0.00000 | 0.00000 | 0.00000 | 0.00000 | 0.00000 | 0.00000 | 0.00000 | 0.00000 | 0.00000 | 0.00000 | 0.10000 | 0.00000 | 0.10000 | 0.00000 | 0.00000 |
| 18-118 | 0.00000 | 0.00000 | 0.00000 | 0.00000 | 0.00000 | 0.00000 | 0.00000 | 0.00000 | 0.00000 | 0.00000 | 0.00000 | 0.00000 | 0.00000 | 0.00000 | 0.00000 | 0.00000 | 0.00000 | 0.00000 | 0.11111 | 0.00000 |
| 18-119 | 0.75000 | 0.30000 | 0.33333 | 0.08333 | 0.08333 | 0.33333 | 0.50000 | 0.33333 | 0.25000 | 0.30000 | 0.16667 | 0.33333 | 0.80000 | 0.33333 | 0.80000 | 0.50000 | 0.25000 | 0.60000 | 0.55556 | 0.33333 |
| 18-120 | 0.37500 | 0.00000 | 0.00000 | 0.00000 | 0.00000 | 0.00000 | 0.00000 | 0.00000 | 0.00000 | 0.00000 | 0.00000 | 0.00000 | 0.00000 | 0.00000 | 0.00000 | 0.00000 | 0.00000 | 0.00000 | 0.00000 | 0.00000 |
| 18-121 | 0.00000 | 0.00000 | 0.00000 | 0.00000 | 0.00000 | 0.00000 | 0.00000 | 0.16667 | 0.00000 | 0.00000 | 0.00000 | 0.00000 | 0.00000 | 0.00000 | 0.00000 | 0.00000 | 0.00000 | 0.00000 | 0.00000 | 0.00000 |
| 18-122 | 0.12500 | 0.00000 | 0.00000 | 0.00000 | 0.00000 | 0.00000 | 0.00000 | 0.00000 | 0.00000 | 0.00000 | 0.00000 | 0.00000 | 0.00000 | 0.00000 | 0.00000 | 0.00000 | 0.00000 | 0.00000 | 0.00000 | 0.00000 |
| 18-123 | 0.12500 | 0.00000 | 0.00000 | 0.00000 | 0.00000 | 0.00000 | 0.00000 | 0.00000 | 0.00000 | 0.00000 | 0.00000 | 0.00000 | 0.00000 | 0.00000 | 0.00000 | 0.10000 | 0.00000 | 0.10000 | 0.11111 | 0.16667 |
| 18-124 | 0.00000 | 0.00000 | 0.00000 | 0.00000 | 0.00000 | 0.00000 | 0.00000 | 0.00000 | 0.00000 | 0.00000 | 0.00000 | 0.00000 | 0.00000 | 0.00000 | 0.00000 | 0.00000 | 0.00000 | 0.00000 | 0.11111 | 0.00000 |
| 18-125 | 0.00000 | 0.00000 | 0.00000 | 0.00000 | 0.00000 | 0.00000 | 0.08333 | 0.00000 | 0.00000 | 0.00000 | 0.00000 | 0.00000 | 0.00000 | 0.00000 | 0.00000 | 0.00000 | 0.00000 | 0.00000 | 0.00000 | 0.00000 |
| 18-126 | 0.00000 | 0.00000 | 0.00000 | 0.00000 | 0.00000 | 0.00000 | 0.08333 | 0.00000 | 0.00000 | 0.00000 | 0.00000 | 0.00000 | 0.00000 | 0.00000 | 0.00000 | 0.00000 | 0.00000 | 0.00000 | 0.00000 | 0.00000 |
| 18-127 | 0.12500 | 0.00000 | 0.00000 | 0.00000 | 0.00000 | 0.00000 | 0.00000 | 0.00000 | 0.00000 | 0.00000 | 0.00000 | 0.00000 | 0.00000 | 0.00000 | 0.00000 | 0.00000 | 0.00000 | 0.00000 | 0.00000 | 0.00000 |
| 18-128 | 0.00000 | 0.00000 | 0.08333 | 0.00000 | 0.00000 | 0.00000 | 0.00000 | 0.00000 | 0.00000 | 0.00000 | 0.00000 | 0.00000 | 0.00000 | 0.00000 | 0.00000 | 0.00000 | 0.00000 | 0.00000 | 0.00000 | 0.00000 |
| 18-129 | 0.00000 | 0.00000 | 0.00000 | 0.08333 | 0.00000 | 0.00000 | 0.00000 | 0.00000 | 0.00000 | 0.00000 | 0.00000 | 0.00000 | 0.00000 | 0.00000 | 0.00000 | 0.00000 | 0.08333 | 0.00000 | 0.00000 | 0.00000 |
| 21-001 | 0.50000 | 0.20000 | 0.00000 | 0.00000 | 0.00000 | 0.16667 | 0.08333 | 0.00000 | 0.00000 | 0.11111 | 0.08333 | 0.00000 | 0.00000 | 0.00000 | 0.00000 | 0.00000 | 0.00000 | 0.00000 | 0.20000 | 0.16667 |
| 21-002 | 0.00000 | 0.20000 | 0.09091 | 0.00000 | 0.00000 | 0.00000 | 0.00000 | 0.00000 | 0.08333 | 0.11111 | 0.00000 | 0.00000 | 0.00000 | 0.08333 | 0.00000 | 0.00000 | 0.00000 | 0.00000 | 0.00000 | 0.33333 |
| 21-003 | 0.00000 | 0.00000 | 0.27273 | 0.08333 | 0.50000 | 0.00000 | 0.00000 | 0.00000 | 0.00000 | 0.00000 | 0.00000 | 0.16667 | 0.00000 | 0.08333 | 0.30000 | 0.00000 | 0.00000 | 0.10000 | 0.20000 | 0.16667 |
| 21-004 | 0.00000 | 0.40000 | 0.45455 | 0.50000 | 0.41667 | 0.00000 | 0.00000 | 0.00000 | 0.00000 | 0.00000 | 0.00000 | 0.00000 | 0.00000 | 0.00000 | 0.00000 | 0.00000 | 0.00000 | 0.00000 | 0.00000 | 0.00000 |
| 21-005 | 0.00000 | 0.40000 | 0.45455 | 0.50000 | 0.50000 | 0.00000 | 0.00000 | 0.00000 | 0.00000 | 0.00000 | 0.00000 | 0.00000 | 0.00000 | 0.00000 | 0.00000 | 0.00000 | 0.00000 | 0.00000 | 0.00000 | 0.16667 |
| 21-006 | 0.00000 | 0.10000 | 0.54545 | 0.16667 | 0.25000 | 0.16667 | 0.00000 | 0.00000 | 0.08333 | 0.00000 | 0.00000 | 0.00000 | 0.00000 | 0.00000 | 0.10000 | 0.00000 | 0.00000 | 0.00000 | 0.00000 | 0.66667 |
| 21-007 | 0.00000 | 0.20000 | 0.00000 | 0.16667 | 0.00000 | 0.16667 | 0.16667 | 0.00000 | 0.08333 | 0.11111 | 0.00000 | 0.00000 | 0.00000 | 0.00000 | 0.00000 | 0.00000 | 0.00000 | 0.00000 | 0.00000 | 0.16667 |
| 21-008 | 0.12500 | 0.50000 | 0.36364 | 0.50000 | 0.50000 | 0.16667 | 0.08333 | 0.00000 | 0.00000 | 0.00000 | 0.00000 | 0.00000 | 0.00000 | 0.00000 | 0.10000 | 0.00000 | 0.00000 | 0.00000 | 0.20000 | 0.16667 |
| 21-009 | 0.87500 | 0.20000 | 0.63636 | 0.25000 | 0.75000 | 0.33333 | 0.00000 | 0.16667 | 0.00000 | 0.11111 | 0.00000 | 0.00000 | 0.00000 | 0.00000 | 0.30000 | 0.00000 | 0.00000 | 0.10000 | 0.30000 | 0.50000 |
| 21-010 | 0.00000 | 0.00000 | 0.00000 | 0.16667 | 0.00000 | 0.00000 | 0.00000 | 0.00000 | 0.00000 | 0.11111 | 0.00000 | 0.00000 | 0.00000 | 0.00000 | 0.00000 | 0.00000 | 0.00000 | 0.00000 | 0.10000 | 0.00000 |
| 21-011 | 0.00000 | 0.30000 | 0.45455 | 0.25000 | 0.58333 | 0.16667 | 0.00000 | 0.00000 | 0.16667 | 0.00000 | 0.08333 | 0.00000 | 0.00000 | 0.00000 | 0.00000 | 0.00000 | 0.00000 | 0.00000 | 0.20000 | 0.00000 |
| 21-012 | 0.87500 | 0.00000 | 0.09091 | 0.25000 | 0.08333 | 0.16667 | 0.08333 | 0.00000 | 0.16667 | 0.22222 | 0.16667 | 0.25000 | 0.00000 | 0.00000 | 0.20000 | 0.00000 | 0.00000 | 0.40000 | 0.40000 | 0.50000 |
| 21-013 | 0.00000 | 0.00000 | 0.00000 | 0.00000 | 0.08333 | 0.00000 | 0.00000 | 0.00000 | 0.41667 | 0.00000 | 0.25000 | 0.00000 | 0.00000 | 0.00000 | 0.00000 | 0.00000 | 0.00000 | 0.00000 | 0.10000 | 0.00000 |
| 21-014 | 0.00000 | 0.00000 | 0.00000 | 0.00000 | 0.00000 | 0.00000 | 0.00000 | 0.00000 | 0.00000 | 0.11111 | 0.00000 | 0.00000 | 0.00000 | 0.08333 | 0.00000 | 0.00000 | 0.00000 | 0.00000 | 0.00000 | 0.00000 |
| 21-015 | 0.00000 | 0.00000 | 0.00000 | 0.00000 | 0.00000 | 0.00000 | 0.00000 | 0.00000 | 0.00000 | 0.11111 | 0.00000 | 0.00000 | 0.00000 | 0.00000 | 0.00000 | 0.00000 | 0.00000 | 0.00000 | 0.10000 | 0.00000 |
| 21-016 | 0.00000 | 0.00000 | 0.00000 | 0.00000 | 0.08333 | 0.00000 | 0.00000 | 0.00000 | 0.00000 | 0.00000 | 0.00000 | 0.00000 | 0.00000 | 0.00000 | 0.00000 | 0.00000 | 0.00000 | 0.00000 | 0.10000 | 0.00000 |
| 21-017 | 0.00000 | 0.00000 | 0.00000 | 0.00000 | 0.08333 | 0.00000 | 0.08333 | 0.00000 | 0.00000 | 0.11111 | 0.00000 | 0.00000 | 0.00000 | 0.00000 | 0.00000 | 0.00000 | 0.00000 | 0.00000 | 0.00000 | 0.50000 |
| 21-018 | 0.00000 | 0.00000 | 0.09091 | 0.00000 | 0.08333 | 0.00000 | 0.08333 | 0.00000 | 0.00000 | 0.11111 | 0.00000 | 0.00000 | 0.00000 | 0.00000 | 0.00000 | 0.00000 | 0.00000 | 0.00000 | 0.00000 | 0.50000 |
| 21-019 | 0.00000 | 0.10000 | 0.00000 | 0.00000 | 0.00000 | 0.00000 | 0.08333 | 0.00000 | 0.00000 | 0.00000 | 0.00000 | 0.00000 | 0.00000 | 0.00000 | 0.00000 | 0.00000 | 0.00000 | 0.00000 | 0.00000 | 0.50000 |
| 21-020 | 0.12500 | 0.50000 | 0.45455 | 0.25000 | 0.50000 | 0.00000 | 0.08333 | 0.00000 | 0.00000 | 0.11111 | 0.00000 | 0.00000 | 0.00000 | 0.00000 | 0.00000 | 0.00000 | 0.00000 | 0.00000 | 0.10000 | 0.00000 |
| 21-021 | 0.25000 | 0.50000 | 0.45455 | 0.41667 | 0.58333 | 0.16667 | 0.08333 | 0.00000 | 0.00000 | 0.22222 | 0.00000 | 0.00000 | 0.00000 | 0.00000 | 0.00000 | 0.00000 | 0.27273 | 0.00000 | 0.20000 | 0.16667 |
| 21-022 | 0.00000 | 0.00000 | 0.00000 | 0.00000 | 0.00000 | 0.16667 | 0.00000 | 0.00000 | 0.00000 | 0.00000 | 0.00000 | 0.08333 | 0.00000 | 0.08333 | 0.00000 | 0.00000 | 0.09091 | 0.00000 | 0.20000 | 0.16667 |
| 21-023 | 0.25000 | 0.40000 | 0.54545 | 0.41667 | 0.66667 | 0.16667 | 0.00000 | 0.16667 | 0.00000 | 0.00000 | 0.00000 | 0.00000 | 0.00000 | 0.16667 | 0.40000 | 0.00000 | 0.18182 | 0.20000 | 0.10000 | 0.33333 |
| 21-024 | 0.12500 | 0.20000 | 0.18182 | 0.00000 | 0.00000 | 0.16667 | 0.00000 | 0.16667 | 0.08333 | 0.11111 | 0.00000 | 0.08333 | 0.00000 | 0.08333 | 0.00000 | 0.00000 | 0.09091 | 0.20000 | 0.20000 | 0.33333 |
| 21-025 | 0.00000 | 0.00000 | 0.09091 | 0.16667 | 0.33333 | 0.00000 | 0.08333 | 0.00000 | 0.16667 | 0.00000 | 0.00000 | 0.00000 | 0.00000 | 0.00000 | 0.00000 | 0.00000 | 0.00000 | 0.00000 | 0.00000 | 0.00000 |
| 21-026 | 0.00000 | 0.00000 | 0.09091 | 0.00000 | 0.08333 | 0.16667 | 0.00000 | 0.00000 | 0.00000 | 0.00000 | 0.00000 | 0.00000 | 0.00000 | 0.00000 | 0.20000 | 0.00000 | 0.09091 | 0.00000 | 0.10000 | 0.16667 |
| 21-027 | 0.00000 | 0.00000 | 0.00000 | 0.00000 | 0.00000 | 0.16667 | 0.08333 | 0.00000 | 0.00000 | 0.22222 | 0.08333 | 0.00000 | 0.00000 | 0.00000 | 0.00000 | 0.00000 | 0.09091 | 0.00000 | 0.20000 | 0.00000 |
| 21-028 | 0.00000 | 0.00000 | 0.00000 | 0.00000 | 0.08333 | 0.00000 | 0.00000 | 0.00000 | 0.08333 | 0.11111 | 0.00000 | 0.00000 | 0.00000 | 0.08333 | 0.20000 | 0.00000 | 0.09091 | 0.00000 | 0.30000 | 0.33333 |
| 21-029 | 0.00000 | 0.00000 | 0.09091 | 0.16667 | 0.08333 | 0.00000 | 0.00000 | 0.00000 | 0.08333 | 0.11111 | 0.00000 | 0.00000 | 0.00000 | 0.08333 | 0.20000 | 0.00000 | 0.00000 | 0.00000 | 0.20000 | 0.33333 |
| 21-030 | 0.00000 | 0.10000 | 0.00000 | 0.16667 | 0.16667 | 0.00000 | 0.08333 | 0.00000 | 0.08333 | 0.11111 | 0.00000 | 0.00000 | 0.00000 | 0.16667 | 0.10000 | 0.00000 | 0.00000 | 0.00000 | 0.10000 | 0.33333 |
| 21-031 | 0.00000 | 0.00000 | 0.09091 | 0.00000 | 0.00000 | 0.00000 | 0.08333 | 0.16667 | 0.25000 | 0.00000 | 0.08333 | 0.08333 | 0.00000 | 0.00000 | 0.00000 | 0.00000 | 0.00000 | 0.00000 | 0.00000 | 0.00000 |
| 21-032 | 0.00000 | 0.00000 | 0.00000 | 0.00000 | 0.00000 | 0.16667 | 0.08333 | 0.00000 | 0.00000 | 0.00000 | 0.00000 | 0.00000 | 0.00000 | 0.00000 | 0.00000 | 0.00000 | 0.00000 | 0.00000 | 0.00000 | 0.00000 |
| 21-033 | 0.00000 | 0.00000 | 0.00000 | 0.00000 | 0.00000 | 0.00000 | 0.00000 | 0.00000 | 0.00000 | 0.00000 | 0.00000 | 0.00000 | 0.00000 | 0.00000 | 0.00000 | 0.00000 | 0.00000 | 0.00000 | 0.00000 | 0.16667 |
| 21-034 | 0.00000 | 0.00000 | 0.00000 | 0.00000 | 0.08333 | 0.00000 | 0.00000 | 0.00000 | 0.00000 | 0.00000 | 0.00000 | 0.00000 | 0.00000 | 0.00000 | 0.00000 | 0.00000 | 0.00000 | 0.10000 | 0.20000 | 0.33333 |
| 21-035 | 0.12500 | 0.00000 | 0.00000 | 0.00000 | 0.00000 | 0.16667 | 0.00000 | 0.00000 | 0.00000 | 0.00000 | 0.00000 | 0.00000 | 0.00000 | 0.08333 | 0.00000 | 0.10000 | 0.09091 | 0.00000 | 0.10000 | 0.50000 |
| 21-036 | 0.00000 | 0.20000 | 0.09091 | 0.00000 | 0.00000 | 0.16667 | 0.08333 | 0.16667 | 0.00000 | 0.00000 | 0.00000 | 0.00000 | 0.00000 | 0.00000 | 0.00000 | 0.00000 | 0.09091 | 0.00000 | 0.30000 | 0.00000 |
| 21-037 | 0.00000 | 0.00000 | 0.00000 | 0.08333 | 0.08333 | 0.00000 | 0.00000 | 0.16667 | 0.00000 | 0.00000 | 0.08333 | 0.00000 | 0.00000 | 0.00000 | 0.00000 | 0.00000 | 0.09091 | 0.00000 | 0.20000 | 0.00000 |
| 21-038 | 0.00000 | 0.00000 | 0.00000 | 0.08333 | 0.00000 | 0.00000 | 0.08333 | 0.16667 | 0.00000 | 0.00000 | 0.08333 | 0.00000 | 0.00000 | 0.00000 | 0.00000 | 0.00000 | 0.00000 | 0.00000 | 0.20000 | 0.00000 |
| 21-039 | 0.00000 | 0.20000 | 0.00000 | 0.00000 | 0.08333 | 0.16667 | 0.00000 | 0.00000 | 0.00000 | 0.11111 | 0.08333 | 0.00000 | 0.00000 | 0.00000 | 0.00000 | 0.00000 | 0.09091 | 0.00000 | 0.30000 | 0.00000 |
| 21-040 | 0.00000 | 0.00000 | 0.00000 | 0.00000 | 0.00000 | 0.00000 | 0.00000 | 0.00000 | 0.00000 | 0.00000 | 0.00000 | 0.00000 | 0.00000 | 0.08333 | 0.00000 | 0.00000 | 0.00000 | 0.00000 | 0.00000 | 0.00000 |
| 21-041 | 0.00000 | 0.00000 | 0.00000 | 0.00000 | 0.00000 | 0.00000 | 0.00000 | 0.00000 | 0.08333 | 0.11111 | 0.08333 | 0.00000 | 0.00000 | 0.08333 | 0.00000 | 0.00000 | 0.18182 | 0.10000 | 0.20000 | 0.50000 |
| 21-042 | 0.00000 | 0.20000 | 0.09091 | 0.00000 | 0.08333 | 0.16667 | 0.00000 | 0.00000 | 0.00000 | 0.11111 | 0.16667 | 0.00000 | 0.00000 | 0.00000 | 0.00000 | 0.00000 | 0.09091 | 0.40000 | 0.40000 | 0.50000 |
| 21-043 | 0.00000 | 0.00000 | 0.00000 | 0.16667 | 0.00000 | 0.00000 | 0.00000 | 0.00000 | 0.00000 | 0.00000 | 0.00000 | 0.25000 | 0.00000 | 0.00000 | 0.00000 | 0.00000 | 0.00000 | 0.00000 | 0.00000 | 0.00000 |
| 21-044 | 0.00000 | 0.10000 | 0.09091 | 0.41667 | 0.50000 | 0.00000 | 0.00000 | 0.00000 | 0.00000 | 0.11111 | 0.00000 | 0.16667 | 0.00000 | 0.08333 | 0.00000 | 0.00000 | 0.27273 | 0.10000 | 0.30000 | 0.50000 |
| 21-045 | 0.87500 | 0.20000 | 0.63636 | 0.66667 | 0.25000 | 0.16667 | 0.00000 | 0.00000 | 0.00000 | 0.22222 | 0.00000 | 0.16667 | 0.00000 | 0.25000 | 0.00000 | 0.00000 | 0.18182 | 0.20000 | 0.00000 | 0.33333 |
| 21-046 | 0.62500 | 0.30000 | 0.63636 | 0.75000 | 0.25000 | 0.16667 | 0.08333 | 0.00000 | 0.00000 | 0.11111 | 0.00000 | 0.16667 | 0.00000 | 0.25000 | 0.10000 | 0.00000 | 0.00000 | 0.10000 | 0.20000 | 0.00000 |
| 21-047 | 0.25000 | 0.10000 | 0.18182 | 0.00000 | 0.25000 | 0.00000 | 0.00000 | 0.00000 | 0.00000 | 0.00000 | 0.08333 | 0.08333 | 0.00000 | 0.00000 | 0.00000 | 0.10000 | 0.00000 | 0.10000 | 0.40000 | 0.33333 |
| 21-048 | 0.00000 | 0.00000 | 0.09091 | 0.00000 | 0.00000 | 0.00000 | 0.00000 | 0.00000 | 0.00000 | 0.11111 | 0.00000 | 0.00000 | 0.00000 | 0.00000 | 0.00000 | 0.00000 | 0.09091 | 0.00000 | 0.10000 | 0.00000 |
| 21-049 | 0.00000 | 0.00000 | 0.18182 | 0.00000 | 0.00000 | 0.00000 | 0.00000 | 0.00000 | 0.00000 | 0.00000 | 0.08333 | 0.00000 | 0.00000 | 0.00000 | 0.00000 | 0.00000 | 0.00000 | 0.00000 | 0.00000 | 0.50000 |
| 21-050 | 0.00000 | 0.00000 | 0.00000 | 0.00000 | 0.00000 | 0.00000 | 0.00000 | 0.00000 | 0.00000 | 0.00000 | 0.08333 | 0.00000 | 0.00000 | 0.25000 | 0.00000 | 0.00000 | 0.18182 | 0.00000 | 0.10000 | 0.33333 |
| 21-051 | 0.12500 | 0.20000 | 0.09091 | 0.00000 | 0.08333 | 0.00000 | 0.00000 | 0.00000 | 0.08333 | 0.00000 | 0.00000 | 0.00000 | 0.00000 | 0.00000 | 0.00000 | 0.00000 | 0.00000 | 0.00000 | 0.20000 | 0.00000 |
| 21-052 | 0.12500 | 0.00000 | 0.00000 | 0.00000 | 0.16667 | 0.00000 | 0.00000 | 0.00000 | 0.00000 | 0.00000 | 0.00000 | 0.00000 | 0.00000 | 0.08333 | 0.00000 | 0.00000 | 0.09091 | 0.00000 | 0.10000 | 0.50000 |
| 21-053 | 0.00000 | 0.00000 | 0.00000 | 0.00000 | 0.00000 | 0.00000 | 0.00000 | 0.00000 | 0.00000 | 0.11111 | 0.00000 | 0.00000 | 0.00000 | 0.00000 | 0.00000 | 0.00000 | 0.09091 | 0.00000 | 0.10000 | 0.50000 |
| 21-054 | 0.00000 | 0.00000 | 0.00000 | 0.00000 | 0.08333 | 0.16667 | 0.16667 | 0.00000 | 0.00000 | 0.33333 | 0.08333 | 0.00000 | 0.00000 | 0.00000 | 0.00000 | 0.00000 | 0.00000 | 0.00000 | 0.10000 | 0.16667 |
| 21-055 | 0.12500 | 0.20000 | 0.36364 | 0.41667 | 0.33333 | 0.00000 | 0.00000 | 0.00000 | 0.00000 | 0.11111 | 0.08333 | 0.00000 | 0.00000 | 0.16667 | 0.00000 | 0.00000 | 0.09091 | 0.00000 | 0.00000 | 0.16667 |
| 21-056 | 0.00000 | 0.20000 | 0.18182 | 0.33333 | 0.33333 | 0.00000 | 0.00000 | 0.00000 | 0.00000 | 0.11111 | 0.00000 | 0.00000 | 0.00000 | 0.00000 | 0.00000 | 0.00000 | 0.09091 | 0.10000 | 0.00000 | 0.00000 |
| 21-057 | 0.00000 | 0.10000 | 0.09091 | 0.00000 | 0.00000 | 0.16667 | 0.16667 | 0.00000 | 0.00000 | 0.33333 | 0.08333 | 0.00000 | 0.00000 | 0.00000 | 0.00000 | 0.00000 | 0.00000 | 0.10000 | 0.00000 | 0.50000 |
| 21-058 | 0.00000 | 0.10000 | 0.18182 | 0.08333 | 0.00000 | 0.33333 | 0.25000 | 0.00000 | 0.00000 | 0.11111 | 0.08333 | 0.00000 | 0.00000 | 0.00000 | 0.00000 | 0.10000 | 0.00000 | 0.00000 | 0.00000 | 0.50000 |
| 21-059 | 0.62500 | 0.00000 | 0.09091 | 0.08333 | 0.00000 | 0.00000 | 0.00000 | 0.00000 | 0.00000 | 0.00000 | 0.08333 | 0.00000 | 0.00000 | 0.00000 | 0.00000 | 0.00000 | 0.09091 | 0.00000 | 0.10000 | 0.16667 |
| 21-060 | 0.00000 | 0.00000 | 0.36364 | 0.00000 | 0.08333 | 0.00000 | 0.00000 | 0.00000 | 0.00000 | 0.00000 | 0.00000 | 0.00000 | 0.00000 | 0.00000 | 0.00000 | 0.00000 | 0.00000 | 0.10000 | 0.00000 | 0.00000 |
| 21-061 | 0.00000 | 0.30000 | 0.72727 | 0.50000 | 0.58333 | 0.00000 | 0.00000 | 0.00000 | 0.00000 | 0.22222 | 0.00000 | 0.16667 | 0.00000 | 0.58333 | 0.30000 | 0.00000 | 0.72727 | 0.60000 | 0.80000 | 0.83333 |
| 21-062 | 0.00000 | 0.20000 | 0.36364 | 0.08333 | 0.00000 | 0.00000 | 0.33333 | 0.00000 | 0.00000 | 0.33333 | 0.08333 | 0.16667 | 0.00000 | 0.00000 | 0.00000 | 0.00000 | 0.00000 | 0.20000 | 0.60000 | 0.33333 |
| 21-063 | 0.00000 | 0.30000 | 0.63636 | 0.33333 | 0.50000 | 0.00000 | 0.25000 | 0.00000 | 0.00000 | 0.22222 | 0.08333 | 0.33333 | 0.00000 | 0.58333 | 0.30000 | 0.00000 | 0.63636 | 0.60000 | 0.60000 | 0.50000 |
| 21-064 | 0.00000 | 0.10000 | 0.00000 | 0.00000 | 0.00000 | 0.00000 | 0.00000 | 0.00000 | 0.00000 | 0.11111 | 0.00000 | 0.00000 | 0.00000 | 0.00000 | 0.00000 | 0.00000 | 0.09091 | 0.00000 | 0.00000 | 0.16667 |
| 21-065 | 0.00000 | 0.20000 | 0.09091 | 0.08333 | 0.08333 | 0.00000 | 0.16667 | 0.00000 | 0.00000 | 0.00000 | 0.00000 | 0.08333 | 0.00000 | 0.00000 | 0.00000 | 0.00000 | 0.09091 | 0.10000 | 0.00000 | 0.33333 |
| 21-066 | 0.00000 | 0.40000 | 0.54545 | 0.33333 | 0.25000 | 0.00000 | 0.16667 | 0.00000 | 0.00000 | 0.22222 | 0.00000 | 0.33333 | 0.00000 | 0.08333 | 0.40000 | 0.00000 | 0.18182 | 0.20000 | 0.20000 | 0.33333 |
| 21-067 | 0.00000 | 0.60000 | 0.54545 | 0.41667 | 0.41667 | 0.00000 | 0.00000 | 0.00000 | 0.08333 | 0.22222 | 0.00000 | 0.08333 | 0.00000 | 0.08333 | 0.00000 | 0.00000 | 0.18182 | 0.30000 | 0.60000 | 0.33333 |
| 21-068 | 0.00000 | 0.20000 | 0.36364 | 0.33333 | 0.50000 | 0.16667 | 0.08333 | 0.00000 | 0.08333 | 0.00000 | 0.00000 | 0.00000 | 0.00000 | 0.25000 | 0.10000 | 0.10000 | 0.00000 | 0.40000 | 0.30000 | 0.33333 |
| 21-069 | 0.00000 | 0.00000 | 0.00000 | 0.00000 | 0.00000 | 0.00000 | 0.00000 | 0.00000 | 0.00000 | 0.00000 | 0.08333 | 0.00000 | 0.00000 | 0.00000 | 0.00000 | 0.00000 | 0.09091 | 0.00000 | 0.10000 | 0.16667 |
| 21-070 | 0.00000 | 0.00000 | 0.00000 | 0.00000 | 0.00000 | 0.00000 | 0.16667 | 0.00000 | 0.00000 | 0.11111 | 0.00000 | 0.00000 | 0.00000 | 0.00000 | 0.00000 | 0.00000 | 0.09091 | 0.00000 | 0.00000 | 0.16667 |
| 21-071 | 0.00000 | 0.00000 | 0.09091 | 0.00000 | 0.00000 | 0.00000 | 0.00000 | 0.00000 | 0.00000 | 0.11111 | 0.00000 | 0.00000 | 0.00000 | 0.00000 | 0.00000 | 0.00000 | 0.18182 | 0.00000 | 0.00000 | 0.00000 |
| 21-072 | 0.00000 | 0.00000 | 0.00000 | 0.08333 | 0.00000 | 0.00000 | 0.00000 | 0.00000 | 0.00000 | 0.22222 | 0.00000 | 0.00000 | 0.00000 | 0.00000 | 0.00000 | 0.00000 | 0.18182 | 0.00000 | 0.00000 | 0.33333 |
| 21-073 | 1.00000 | 0.00000 | 0.09091 | 0.08333 | 0.00000 | 0.00000 | 0.08333 | 0.00000 | 0.00000 | 0.22222 | 0.00000 | 0.00000 | 0.00000 | 0.00000 | 0.00000 | 0.00000 | 0.00000 | 0.00000 | 0.00000 | 0.33333 |
| 21-074 | 0.00000 | 0.00000 | 0.00000 | 0.00000 | 0.00000 | 0.00000 | 0.08333 | 0.00000 | 0.00000 | 0.11111 | 0.00000 | 0.00000 | 0.00000 | 0.00000 | 0.00000 | 0.00000 | 0.00000 | 0.00000 | 0.00000 | 0.33333 |
| 21-075 | 0.00000 | 0.10000 | 0.09091 | 0.08333 | 0.00000 | 0.00000 | 0.16667 | 0.00000 | 0.00000 | 0.11111 | 0.00000 | 0.00000 | 0.00000 | 0.16667 | 0.00000 | 0.00000 | 0.09091 | 0.00000 | 0.10000 | 0.16667 |
| 21-076 | 1.00000 | 0.80000 | 0.90909 | 0.83333 | 0.83333 | 0.66667 | 0.16667 | 0.00000 | 0.16667 | 0.11111 | 0.00000 | 0.00000 | 0.00000 | 0.16667 | 0.00000 | 0.00000 | 0.09091 | 0.20000 | 0.40000 | 0.16667 |
| 21-077 | 0.00000 | 0.20000 | 0.09091 | 0.16667 | 0.25000 | 0.00000 | 0.16667 | 1.00000 | 0.75000 | 0.22222 | 0.50000 | 1.00000 | 0.50000 | 1.00000 | 0.80000 | 0.80000 | 0.81818 | 0.90000 | 0.80000 | 0.66667 |
| 21-078 | 0.00000 | 0.00000 | 0.27273 | 0.00000 | 0.25000 | 0.00000 | 0.00000 | 0.00000 | 0.00000 | 0.11111 | 0.00000 | 0.00000 | 0.00000 | 0.00000 | 0.00000 | 0.00000 | 0.18182 | 0.20000 | 0.10000 | 0.33333 |
| 21-079 | 0.00000 | 0.00000 | 0.09091 | 0.00000 | 0.00000 | 0.00000 | 0.00000 | 0.00000 | 0.00000 | 0.00000 | 0.00000 | 0.00000 | 0.00000 | 0.00000 | 0.00000 | 0.00000 | 0.00000 | 0.00000 | 0.00000 | 0.16667 |
| 21-080 | 0.00000 | 0.00000 | 0.00000 | 0.00000 | 0.00000 | 0.00000 | 0.00000 | 0.00000 | 0.00000 | 0.33333 | 0.00000 | 0.16667 | 0.60000 | 0.08333 | 0.20000 | 0.10000 | 0.00000 | 0.00000 | 0.20000 | 0.00000 |
| 21-081 | 0.00000 | 0.10000 | 0.09091 | 0.00000 | 0.08333 | 0.00000 | 0.08333 | 0.00000 | 0.08333 | 0.22222 | 0.00000 | 0.16667 | 0.90000 | 0.16667 | 0.40000 | 0.60000 | 0.00000 | 0.00000 | 0.30000 | 0.16667 |
| 21-082 | 1.00000 | 0.40000 | 0.72727 | 0.33333 | 0.58333 | 0.33333 | 0.25000 | 1.00000 | 0.83333 | 0.44444 | 0.50000 | 0.33333 | 0.00000 | 0.41667 | 0.20000 | 0.10000 | 0.63636 | 0.50000 | 0.80000 | 0.33333 |
| 21-083 | 0.00000 | 0.20000 | 0.09091 | 0.25000 | 0.25000 | 0.16667 | 0.08333 | 0.83333 | 0.66667 | 0.33333 | 0.50000 | 0.08333 | 0.00000 | 0.41667 | 0.40000 | 0.00000 | 0.63636 | 0.60000 | 0.60000 | 0.33333 |
| 21-084 | 0.87500 | 0.90000 | 0.72727 | 0.50000 | 0.25000 | 0.16667 | 0.08333 | 0.16667 | 0.16667 | 0.22222 | 0.16667 | 0.41667 | 0.00000 | 0.58333 | 0.40000 | 0.00000 | 0.45455 | 0.50000 | 0.20000 | 0.16667 |
| 21-085 | 0.00000 | 0.60000 | 0.36364 | 0.25000 | 0.08333 | 0.16667 | 0.00000 | 0.00000 | 0.00000 | 0.11111 | 0.00000 | 0.25000 | 0.00000 | 0.41667 | 0.40000 | 0.00000 | 0.54545 | 0.40000 | 0.60000 | 0.00000 |
| 21-086 | 0.00000 | 0.10000 | 0.00000 | 0.00000 | 0.08333 | 0.00000 | 0.00000 | 0.00000 | 0.00000 | 0.11111 | 0.00000 | 0.00000 | 0.00000 | 0.08333 | 0.00000 | 0.00000 | 0.00000 | 0.00000 | 0.10000 | 0.00000 |
| 21-087 | 0.00000 | 0.50000 | 0.09091 | 0.25000 | 0.00000 | 0.00000 | 0.00000 | 0.00000 | 0.00000 | 0.11111 | 0.00000 | 0.08333 | 0.00000 | 0.00000 | 0.00000 | 0.00000 | 0.00000 | 0.00000 | 0.00000 | 0.00000 |
| 21-088 | 0.12500 | 0.70000 | 0.36364 | 0.08333 | 0.08333 | 0.00000 | 0.00000 | 0.00000 | 0.00000 | 0.00000 | 0.00000 | 0.00000 | 0.00000 | 0.16667 | 0.00000 | 0.00000 | 0.00000 | 0.00000 | 0.10000 | 0.16667 |
| 21-089 | 0.37500 | 0.00000 | 0.36364 | 0.08333 | 0.00000 | 0.00000 | 0.08333 | 0.00000 | 0.00000 | 0.11111 | 0.00000 | 0.00000 | 0.00000 | 0.00000 | 0.00000 | 0.00000 | 0.00000 | 0.00000 | 0.00000 | 0.33333 |
| 21-090 | 0.00000 | 0.00000 | 0.27273 | 0.00000 | 0.08333 | 0.16667 | 0.08333 | 0.00000 | 0.08333 | 0.00000 | 0.00000 | 0.00000 | 0.00000 | 0.00000 | 0.00000 | 0.00000 | 0.00000 | 0.20000 | 0.30000 | 0.66667 |
| 21-091 | 0.00000 | 0.00000 | 0.00000 | 0.25000 | 0.16667 | 0.50000 | 0.16667 | 0.50000 | 0.41667 | 0.22222 | 0.00000 | 0.00000 | 0.00000 | 0.00000 | 0.00000 | 0.00000 | 0.00000 | 0.60000 | 0.80000 | 0.33333 |
| 21-092 | 1.00000 | 0.50000 | 0.45455 | 0.25000 | 0.66667 | 0.33333 | 0.25000 | 0.66667 | 0.33333 | 0.22222 | 0.16667 | 0.33333 | 0.00000 | 0.33333 | 0.20000 | 0.00000 | 0.27273 | 0.90000 | 0.80000 | 0.33333 |
| 21-093 | 0.00000 | 0.50000 | 0.54545 | 0.16667 | 0.66667 | 0.33333 | 0.08333 | 0.16667 | 0.25000 | 0.11111 | 0.08333 | 0.33333 | 0.00000 | 0.33333 | 0.30000 | 0.00000 | 0.54545 | 0.60000 | 0.60000 | 0.33333 |
| 21-094 | 1.00000 | 0.20000 | 0.36364 | 0.08333 | 0.25000 | 0.50000 | 0.16667 | 0.50000 | 0.25000 | 0.11111 | 0.08333 | 0.33333 | 0.00000 | 0.08333 | 0.00000 | 0.00000 | 0.54545 | 0.40000 | 0.20000 | 0.16667 |
| 21-095 | 0.75000 | 0.00000 | 0.00000 | 0.00000 | 0.00000 | 0.33333 | 0.08333 | 0.50000 | 0.16667 | 0.11111 | 0.00000 | 0.25000 | 0.00000 | 0.00000 | 0.00000 | 0.00000 | 0.18182 | 0.10000 | 0.30000 | 0.16667 |
| 21-096 | 0.00000 | 0.00000 | 0.18182 | 0.00000 | 0.00000 | 0.00000 | 0.00000 | 0.00000 | 0.00000 | 0.00000 | 0.00000 | 0.00000 | 0.00000 | 0.08333 | 0.00000 | 0.00000 | 0.09091 | 0.00000 | 0.00000 | 0.16667 |
| 21-097 | 0.12500 | 0.30000 | 0.45455 | 0.25000 | 0.25000 | 0.66667 | 0.25000 | 0.16667 | 0.33333 | 0.11111 | 0.33333 | 0.33333 | 1.00000 | 0.75000 | 0.90000 | 0.80000 | 0.81818 | 0.30000 | 0.20000 | 0.50000 |
| 21-098 | 0.12500 | 0.80000 | 0.45455 | 0.33333 | 0.41667 | 0.16667 | 0.25000 | 0.16667 | 0.08333 | 0.33333 | 0.25000 | 0.08333 | 0.00000 | 0.41667 | 0.50000 | 0.00000 | 0.63636 | 0.10000 | 0.40000 | 0.66667 |
| 21-099 | 0.00000 | 0.50000 | 0.27273 | 0.00000 | 0.00000 | 0.00000 | 0.00000 | 0.00000 | 0.00000 | 0.22222 | 0.08333 | 0.08333 | 0.00000 | 0.00000 | 0.10000 | 0.00000 | 0.00000 | 0.00000 | 0.00000 | 0.33333 |
| 21-100 | 0.00000 | 0.00000 | 0.09091 | 0.00000 | 0.00000 | 0.00000 | 0.00000 | 0.00000 | 0.00000 | 0.11111 | 0.00000 | 0.00000 | 0.00000 | 0.00000 | 0.00000 | 0.00000 | 0.00000 | 0.00000 | 0.00000 | 0.00000 |
| 21-101 | 0.00000 | 0.00000 | 0.00000 | 0.00000 | 0.00000 | 0.00000 | 0.00000 | 0.00000 | 0.00000 | 0.00000 | 0.00000 | 0.00000 | 0.00000 | 0.08333 | 0.00000 | 0.00000 | 0.00000 | 0.00000 | 0.10000 | 0.00000 |
| 21-102 | 0.00000 | 0.00000 | 0.00000 | 0.00000 | 0.00000 | 0.00000 | 0.00000 | 0.00000 | 0.00000 | 0.00000 | 0.00000 | 0.00000 | 0.00000 | 0.16667 | 0.00000 | 0.00000 | 0.00000 | 0.10000 | 0.10000 | 0.16667 |
| 21-103 | 0.00000 | 0.00000 | 0.00000 | 0.00000 | 0.00000 | 0.00000 | 0.00000 | 0.00000 | 0.08333 | 0.00000 | 0.00000 | 0.00000 | 0.00000 | 0.00000 | 0.00000 | 0.00000 | 0.00000 | 0.00000 | 0.00000 | 0.00000 |
| 21-104 | 0.00000 | 0.00000 | 0.00000 | 0.00000 | 0.00000 | 0.00000 | 0.00000 | 0.00000 | 0.00000 | 0.00000 | 0.00000 | 0.00000 | 0.00000 | 0.00000 | 0.00000 | 0.00000 | 0.00000 | 0.00000 | 0.10000 | 0.00000 |
| 21-105 | 0.00000 | 0.20000 | 0.00000 | 0.00000 | 0.08333 | 0.00000 | 0.00000 | 0.00000 | 0.08333 | 0.00000 | 0.00000 | 0.00000 | 0.00000 | 0.00000 | 0.10000 | 0.00000 | 0.09091 | 0.00000 | 0.00000 | 0.00000 |
| 21-106 | 0.00000 | 0.20000 | 0.09091 | 0.00000 | 0.00000 | 0.00000 | 0.08333 | 0.00000 | 0.00000 | 0.00000 | 0.00000 | 0.00000 | 0.00000 | 0.00000 | 0.10000 | 0.00000 | 0.18182 | 0.00000 | 0.30000 | 0.33333 |
| 21-107 | 0.25000 | 0.30000 | 0.27273 | 0.16667 | 0.25000 | 0.00000 | 0.00000 | 0.16667 | 0.08333 | 0.11111 | 0.08333 | 0.16667 | 0.00000 | 0.16667 | 0.00000 | 0.00000 | 0.45455 | 0.00000 | 0.40000 | 0.33333 |
| 21-108 | 0.12500 | 0.10000 | 0.63636 | 0.16667 | 0.33333 | 0.00000 | 0.00000 | 0.00000 | 0.00000 | 0.33333 | 0.00000 | 0.33333 | 0.00000 | 0.33333 | 0.00000 | 0.10000 | 0.45455 | 0.40000 | 0.70000 | 0.16667 |
| 21-109 | 0.00000 | 0.40000 | 0.72727 | 0.08333 | 0.33333 | 0.00000 | 0.08333 | 0.00000 | 0.00000 | 0.22222 | 0.00000 | 0.16667 | 0.00000 | 0.33333 | 0.00000 | 0.00000 | 0.36364 | 0.30000 | 0.20000 | 0.50000 |
| 21-110 | 0.00000 | 0.10000 | 0.18182 | 0.00000 | 0.08333 | 0.00000 | 0.00000 | 0.00000 | 0.00000 | 0.11111 | 0.00000 | 0.00000 | 0.00000 | 0.00000 | 0.00000 | 0.00000 | 0.00000 | 0.10000 | 0.20000 | 0.16667 |
| 21-111 | 0.25000 | 0.00000 | 0.09091 | 0.08333 | 0.00000 | 0.00000 | 0.00000 | 0.16667 | 0.00000 | 0.00000 | 0.00000 | 0.00000 | 0.00000 | 0.08333 | 0.00000 | 0.00000 | 0.00000 | 0.10000 | 0.00000 | 0.00000 |
| 21-112 | 1.00000 | 0.20000 | 0.27273 | 0.08333 | 0.16667 | 0.16667 | 0.33333 | 0.00000 | 0.08333 | 0.11111 | 0.08333 | 0.25000 | 0.00000 | 0.00000 | 0.00000 | 0.00000 | 0.00000 | 0.30000 | 0.20000 | 0.33333 |
| 21-113 | 0.12500 | 0.00000 | 0.00000 | 0.00000 | 0.00000 | 0.00000 | 0.08333 | 0.00000 | 0.00000 | 0.00000 | 0.00000 | 0.08333 | 0.00000 | 0.00000 | 0.00000 | 0.00000 | 0.00000 | 0.00000 | 0.00000 | 0.00000 |
| 21-114 | 0.00000 | 0.10000 | 0.18182 | 0.25000 | 0.25000 | 0.00000 | 0.08333 | 0.00000 | 0.00000 | 0.11111 | 0.00000 | 0.08333 | 0.00000 | 0.25000 | 0.00000 | 0.00000 | 0.09091 | 0.10000 | 0.30000 | 0.00000 |
| 21-115 | 0.75000 | 0.90000 | 1.00000 | 0.83333 | 0.91667 | 0.00000 | 0.00000 | 0.00000 | 0.08333 | 0.33333 | 0.00000 | 0.50000 | 0.00000 | 0.66667 | 0.40000 | 0.20000 | 0.63636 | 0.90000 | 0.80000 | 0.33333 |
| 21-116 | 0.50000 | 1.00000 | 1.00000 | 0.91667 | 1.00000 | 0.00000 | 0.00000 | 0.50000 | 0.08333 | 0.33333 | 0.00000 | 0.50000 | 0.00000 | 0.75000 | 0.40000 | 0.20000 | 0.63636 | 0.90000 | 0.90000 | 0.33333 |
| 21-117 | 0.00000 | 0.00000 | 0.09091 | 0.00000 | 0.00000 | 0.00000 | 0.00000 | 0.50000 | 0.00000 | 0.00000 | 0.00000 | 0.08333 | 0.00000 | 0.00000 | 0.10000 | 0.00000 | 0.18182 | 0.40000 | 0.10000 | 0.00000 |
| 21-118 | 0.00000 | 0.00000 | 0.00000 | 0.00000 | 0.00000 | 0.00000 | 0.00000 | 0.00000 | 0.00000 | 0.00000 | 0.00000 | 0.08333 | 0.00000 | 0.00000 | 0.00000 | 0.00000 | 0.00000 | 0.00000 | 0.10000 | 0.00000 |
| 21-119 | 0.12500 | 0.00000 | 0.00000 | 0.00000 | 0.00000 | 0.00000 | 0.00000 | 0.00000 | 0.00000 | 0.00000 | 0.00000 | 0.00000 | 0.00000 | 0.00000 | 0.00000 | 0.00000 | 0.00000 | 0.00000 | 0.00000 | 0.00000 |
| 21-120 | 0.00000 | 0.30000 | 0.36364 | 0.66667 | 0.25000 | 0.16667 | 0.16667 | 0.00000 | 0.08333 | 0.00000 | 0.16667 | 0.00000 | 0.00000 | 0.25000 | 0.10000 | 0.00000 | 0.27273 | 0.10000 | 0.00000 | 0.00000 |
| 21-121 | 0.00000 | 0.20000 | 0.36364 | 0.66667 | 0.25000 | 0.00000 | 0.16667 | 0.00000 | 0.08333 | 0.00000 | 0.16667 | 0.00000 | 0.00000 | 0.25000 | 0.10000 | 0.10000 | 0.18182 | 0.10000 | 0.00000 | 0.16667 |
| 21-122 | 0.00000 | 0.00000 | 0.00000 | 0.08333 | 0.00000 | 0.16667 | 0.16667 | 0.00000 | 0.16667 | 0.00000 | 0.00000 | 0.25000 | 0.00000 | 0.00000 | 0.00000 | 0.00000 | 0.09091 | 0.00000 | 0.10000 | 0.16667 |
| 21-123 | 0.00000 | 0.00000 | 0.00000 | 0.00000 | 0.00000 | 0.00000 | 0.00000 | 0.00000 | 0.00000 | 0.00000 | 0.00000 | 0.00000 | 0.00000 | 0.00000 | 0.00000 | 0.00000 | 0.00000 | 0.00000 | 0.10000 | 0.00000 |
| 21-124 | 0.00000 | 0.80000 | 0.54545 | 0.58333 | 0.75000 | 0.66667 | 0.08333 | 0.66667 | 0.08333 | 0.00000 | 0.16667 | 0.25000 | 0.00000 | 0.50000 | 0.30000 | 0.10000 | 0.45455 | 0.70000 | 0.90000 | 0.33333 |
| 21-125 | 1.00000 | 0.90000 | 0.90909 | 0.91667 | 0.91667 | 0.66667 | 0.08333 | 0.66667 | 0.33333 | 0.44444 | 0.33333 | 0.41667 | 0.00000 | 0.58333 | 0.30000 | 0.10000 | 0.54545 | 0.80000 | 0.90000 | 0.33333 |
| 21-126 | 1.00000 | 0.90000 | 0.63636 | 0.66667 | 0.83333 | 0.33333 | 0.41667 | 0.66667 | 0.41667 | 0.44444 | 0.08333 | 0.33333 | 0.00000 | 0.25000 | 0.00000 | 0.00000 | 0.36364 | 0.60000 | 0.60000 | 0.00000 |
| 21-127 | 0.00000 | 0.30000 | 0.18182 | 0.25000 | 0.16667 | 0.00000 | 0.00000 | 0.00000 | 0.00000 | 0.11111 | 0.00000 | 0.00000 | 0.00000 | 0.00000 | 0.00000 | 0.00000 | 0.00000 | 0.10000 | 0.10000 | 0.00000 |
| 21-128 | 0.00000 | 0.10000 | 0.09091 | 0.00000 | 0.00000 | 0.00000 | 0.08333 | 0.00000 | 0.00000 | 0.11111 | 0.00000 | 0.00000 | 0.00000 | 0.00000 | 0.00000 | 0.00000 | 0.18182 | 0.00000 | 0.30000 | 0.33333 |
| 21-129 | 0.00000 | 0.00000 | 0.00000 | 0.00000 | 0.00000 | 0.00000 | 0.08333 | 0.00000 | 0.00000 | 0.00000 | 0.00000 | 0.00000 | 0.00000 | 0.00000 | 0.00000 | 0.00000 | 0.00000 | 0.00000 | 0.10000 | 0.00000 |
| 21-130 | 0.00000 | 0.00000 | 0.00000 | 0.00000 | 0.00000 | 0.00000 | 0.08333 | 0.00000 | 0.00000 | 0.00000 | 0.08333 | 0.00000 | 0.00000 | 0.00000 | 0.00000 | 0.00000 | 0.00000 | 0.00000 | 0.00000 | 0.33333 |
| 21-131 | 0.00000 | 0.00000 | 0.00000 | 0.00000 | 0.00000 | 0.00000 | 0.00000 | 0.00000 | 0.00000 | 0.11111 | 0.00000 | 0.00000 | 0.00000 | 0.00000 | 0.00000 | 0.00000 | 0.00000 | 0.10000 | 0.00000 | 0.00000 |
| 21-132 | 0.00000 | 0.00000 | 0.00000 | 0.00000 | 0.00000 | 0.00000 | 0.08333 | 0.00000 | 0.00000 | 0.11111 | 0.08333 | 0.00000 | 0.00000 | 0.08333 | 0.00000 | 0.00000 | 0.18182 | 0.10000 | 0.00000 | 0.16667 |
| 21-133 | 0.00000 | 0.10000 | 0.09091 | 0.00000 | 0.08333 | 0.00000 | 0.08333 | 0.00000 | 0.00000 | 0.00000 | 0.00000 | 0.00000 | 0.00000 | 0.08333 | 0.00000 | 0.00000 | 0.18182 | 0.00000 | 0.00000 | 0.00000 |
| 21-134 | 0.00000 | 0.00000 | 0.00000 | 0.00000 | 0.00000 | 0.00000 | 0.00000 | 0.00000 | 0.00000 | 0.00000 | 0.00000 | 0.00000 | 0.00000 | 0.00000 | 0.00000 | 0.00000 | 0.00000 | 0.00000 | 0.20000 | 0.00000 |
| 21-135 | 0.00000 | 0.00000 | 0.00000 | 0.00000 | 0.00000 | 0.00000 | 0.00000 | 0.00000 | 0.00000 | 0.00000 | 0.00000 | 0.00000 | 0.00000 | 0.00000 | 0.00000 | 0.00000 | 0.00000 | 0.00000 | 0.10000 | 0.00000 |
| 21-136 | 0.50000 | 0.40000 | 0.45455 | 0.58333 | 0.25000 | 0.66667 | 0.33333 | 1.00000 | 1.00000 | 0.00000 | 0.66667 | 0.16667 | 0.00000 | 0.08333 | 0.00000 | 0.00000 | 0.27273 | 0.00000 | 0.20000 | 0.33333 |
| 21-137 | 0.12500 | 0.00000 | 0.00000 | 0.00000 | 0.00000 | 0.00000 | 0.08333 | 0.00000 | 0.25000 | 0.00000 | 0.00000 | 0.00000 | 0.00000 | 0.00000 | 0.00000 | 0.00000 | 0.00000 | 0.10000 | 0.00000 | 0.00000 |
| 21-138 | 0.00000 | 0.00000 | 0.00000 | 0.00000 | 0.00000 | 0.00000 | 0.08333 | 0.00000 | 0.00000 | 0.00000 | 0.00000 | 0.00000 | 0.00000 | 0.00000 | 0.00000 | 0.00000 | 0.00000 | 0.00000 | 0.00000 | 0.00000 |
| 21-139 | 0.00000 | 0.00000 | 0.00000 | 0.00000 | 0.00000 | 0.00000 | 0.08333 | 0.00000 | 0.00000 | 0.00000 | 0.00000 | 0.00000 | 0.00000 | 0.00000 | 0.00000 | 0.00000 | 0.00000 | 0.00000 | 0.00000 | 0.00000 |
| 21-140 | 0.00000 | 0.00000 | 0.00000 | 0.00000 | 0.00000 | 0.00000 | 0.00000 | 0.00000 | 0.00000 | 0.00000 | 0.00000 | 0.00000 | 0.00000 | 0.00000 | 0.00000 | 0.00000 | 0.00000 | 0.00000 | 0.00000 | 0.16667 |
| 21-141 | 0.00000 | 0.00000 | 0.09091 | 0.00000 | 0.00000 | 0.16667 | 0.00000 | 0.00000 | 0.08333 | 0.11111 | 0.00000 | 0.00000 | 0.00000 | 0.00000 | 0.00000 | 0.00000 | 0.00000 | 0.10000 | 0.00000 | 0.00000 |
| 21-142 | 1.00000 | 0.60000 | 0.09091 | 0.25000 | 0.16667 | 0.33333 | 0.25000 | 0.16667 | 0.75000 | 0.44444 | 0.83333 | 0.16667 | 0.00000 | 0.50000 | 0.00000 | 0.00000 | 0.54545 | 0.70000 | 0.40000 | 0.33333 |
| 21-143 | 0.00000 | 0.10000 | 0.18182 | 0.08333 | 0.16667 | 0.00000 | 0.16667 | 0.00000 | 0.83333 | 0.11111 | 0.41667 | 0.00000 | 0.00000 | 0.08333 | 0.00000 | 0.00000 | 0.45455 | 0.10000 | 0.00000 | 0.00000 |
| 21-144 | 1.00000 | 0.50000 | 0.27273 | 0.16667 | 0.08333 | 0.16667 | 0.33333 | 0.16667 | 0.25000 | 0.33333 | 0.75000 | 0.16667 | 0.00000 | 0.41667 | 0.00000 | 0.00000 | 0.27273 | 0.70000 | 0.40000 | 0.33333 |
| 21-145 | 0.00000 | 0.20000 | 0.00000 | 0.08333 | 0.00000 | 0.00000 | 0.00000 | 0.00000 | 0.25000 | 0.00000 | 0.08333 | 0.00000 | 0.00000 | 0.00000 | 0.00000 | 0.00000 | 0.18182 | 0.10000 | 0.00000 | 0.00000 |
| 21-146 | 0.00000 | 0.00000 | 0.00000 | 0.00000 | 0.00000 | 0.00000 | 0.00000 | 0.00000 | 0.00000 | 0.00000 | 0.00000 | 0.00000 | 0.00000 | 0.00000 | 0.00000 | 0.00000 | 0.00000 | 0.00000 | 0.00000 | 0.16667 |
| 21-147 | 0.00000 | 0.20000 | 0.09091 | 0.08333 | 0.08333 | 0.00000 | 0.00000 | 0.00000 | 0.00000 | 0.00000 | 0.00000 | 0.00000 | 0.00000 | 0.00000 | 0.00000 | 0.00000 | 0.09091 | 0.00000 | 0.00000 | 0.00000 |
| 21-148 | 0.00000 | 0.00000 | 0.00000 | 0.00000 | 0.00000 | 0.00000 | 0.08333 | 0.00000 | 0.16667 | 0.00000 | 0.00000 | 0.00000 | 0.00000 | 0.00000 | 0.00000 | 0.00000 | 0.09091 | 0.00000 | 0.00000 | 0.00000 |
| 21-149 | 0.00000 | 0.00000 | 0.00000 | 0.00000 | 0.00000 | 0.00000 | 0.00000 | 0.00000 | 0.08333 | 0.00000 | 0.00000 | 0.00000 | 0.00000 | 0.00000 | 0.00000 | 0.00000 | 0.00000 | 0.00000 | 0.10000 | 0.00000 |
| 21-150 | 0.00000 | 0.00000 | 0.00000 | 0.16667 | 0.00000 | 0.00000 | 0.00000 | 0.50000 | 0.00000 | 0.00000 | 0.16667 | 0.00000 | 0.00000 | 0.00000 | 0.00000 | 0.00000 | 0.00000 | 0.00000 | 0.20000 | 0.00000 |
| 21-151 | 0.12500 | 0.00000 | 0.00000 | 0.08333 | 0.00000 | 0.00000 | 0.00000 | 0.00000 | 0.08333 | 0.00000 | 0.08333 | 0.00000 | 0.00000 | 0.00000 | 0.00000 | 0.00000 | 0.00000 | 0.00000 | 0.00000 | 0.00000 |
| 21-152 | 0.12500 | 0.00000 | 0.00000 | 0.08333 | 0.00000 | 0.00000 | 0.00000 | 0.50000 | 0.00000 | 0.11111 | 0.08333 | 0.00000 | 0.00000 | 0.00000 | 0.00000 | 0.00000 | 0.00000 | 0.00000 | 0.00000 | 0.00000 |
| 21-153 | 0.00000 | 0.00000 | 0.09091 | 0.08333 | 0.00000 | 0.16667 | 0.00000 | 0.16667 | 0.00000 | 0.11111 | 0.16667 | 0.00000 | 0.00000 | 0.00000 | 0.00000 | 0.00000 | 0.00000 | 0.00000 | 0.00000 | 0.00000 |
| 21-154 | 0.00000 | 0.00000 | 0.09091 | 0.00000 | 0.00000 | 0.00000 | 0.00000 | 0.16667 | 0.00000 | 0.00000 | 0.00000 | 0.00000 | 0.10000 | 0.00000 | 0.00000 | 0.00000 | 0.00000 | 0.00000 | 0.00000 | 0.00000 |
| 21-155 | 0.00000 | 0.00000 | 0.00000 | 0.00000 | 0.00000 | 0.00000 | 0.00000 | 0.00000 | 0.00000 | 0.00000 | 0.00000 | 0.00000 | 0.00000 | 0.00000 | 0.00000 | 0.00000 | 0.00000 | 0.00000 | 0.10000 | 0.33333 |
| 21-156 | 0.00000 | 0.10000 | 0.00000 | 0.00000 | 0.00000 | 0.00000 | 0.00000 | 0.00000 | 0.00000 | 0.00000 | 0.00000 | 0.00000 | 0.00000 | 0.00000 | 0.00000 | 0.00000 | 0.00000 | 0.00000 | 0.10000 | 0.66667 |
| 21-157 | 0.12500 | 0.00000 | 0.00000 | 0.00000 | 0.00000 | 0.00000 | 0.00000 | 0.00000 | 0.00000 | 0.00000 | 0.00000 | 0.00000 | 0.00000 | 0.00000 | 0.00000 | 0.00000 | 0.00000 | 0.00000 | 0.00000 | 0.00000 |
| 21-158 | 0.12500 | 0.00000 | 0.00000 | 0.00000 | 0.00000 | 0.00000 | 0.00000 | 0.00000 | 0.00000 | 0.00000 | 0.00000 | 0.00000 | 0.00000 | 0.00000 | 0.00000 | 0.00000 | 0.00000 | 0.00000 | 0.00000 | 0.16667 |
| 21-159 | 0.00000 | 0.00000 | 0.00000 | 0.00000 | 0.00000 | 0.00000 | 0.00000 | 0.00000 | 0.00000 | 0.00000 | 0.00000 | 0.00000 | 0.00000 | 0.00000 | 0.00000 | 0.00000 | 0.09091 | 0.00000 | 0.00000 | 0.00000 |
| 21-160 | 0.00000 | 0.00000 | 0.00000 | 0.00000 | 0.00000 | 0.00000 | 0.00000 | 0.00000 | 0.00000 | 0.00000 | 0.00000 | 0.08333 | 0.00000 | 0.00000 | 0.00000 | 0.00000 | 0.00000 | 0.20000 | 0.00000 | 0.33333 |
| 31-001 | 0.00000 | 0.00000 | 0.00000 | 0.00000 | 0.00000 | 0.16667 | 0.16667 | 0.00000 | 0.00000 | 0.12500 | 0.00000 | 0.00000 | 0.00000 | 0.00000 | 0.00000 | 0.14286 | 0.16667 | 0.00000 | 0.00000 | 0.16667 |
| 31-002 | 0.00000 | 0.00000 | 0.00000 | 0.00000 | 0.00000 | 0.16667 | 0.00000 | 0.00000 | 0.00000 | 0.00000 | 0.00000 | 0.00000 | 0.00000 | 0.00000 | 0.00000 | 0.14286 | 0.16667 | 0.00000 | 0.00000 | 0.00000 |
| 31-003 | 0.00000 | 0.00000 | 0.00000 | 0.00000 | 0.00000 | 0.00000 | 0.08333 | 0.00000 | 0.00000 | 0.00000 | 0.00000 | 0.00000 | 0.00000 | 0.00000 | 0.00000 | 0.00000 | 0.00000 | 0.00000 | 0.00000 | 0.33333 |
| 31-004 | 0.00000 | 0.00000 | 0.00000 | 0.00000 | 0.00000 | 0.00000 | 0.08333 | 0.00000 | 0.00000 | 0.12500 | 0.00000 | 0.00000 | 0.00000 | 0.00000 | 0.00000 | 0.00000 | 0.00000 | 0.00000 | 0.00000 | 0.00000 |
| 31-005 | 0.00000 | 0.00000 | 0.10000 | 0.00000 | 0.00000 | 0.16667 | 0.16667 | 0.00000 | 0.08333 | 0.25000 | 0.00000 | 0.00000 | 0.00000 | 0.00000 | 0.00000 | 0.00000 | 0.00000 | 0.00000 | 0.00000 | 0.00000 |
| 31-006 | 0.00000 | 0.00000 | 0.10000 | 0.00000 | 0.00000 | 0.00000 | 0.00000 | 0.00000 | 0.08333 | 0.00000 | 0.00000 | 0.00000 | 0.00000 | 0.00000 | 0.00000 | 0.00000 | 0.00000 | 0.00000 | 0.00000 | 0.16667 |
| 31-007 | 0.00000 | 0.00000 | 0.00000 | 0.00000 | 0.00000 | 0.16667 | 0.33333 | 0.00000 | 0.00000 | 0.12500 | 0.10000 | 0.00000 | 0.00000 | 0.00000 | 0.00000 | 0.00000 | 0.00000 | 0.00000 | 0.00000 | 0.16667 |
| 31-008 | 0.00000 | 0.00000 | 0.00000 | 0.00000 | 0.00000 | 0.00000 | 0.00000 | 0.16667 | 0.00000 | 0.00000 | 0.00000 | 0.00000 | 0.00000 | 0.00000 | 0.00000 | 0.00000 | 0.00000 | 0.00000 | 0.00000 | 0.00000 |
| 31-009 | 0.00000 | 0.00000 | 0.00000 | 0.00000 | 0.00000 | 0.00000 | 0.00000 | 0.16667 | 0.00000 | 0.00000 | 0.00000 | 0.00000 | 0.00000 | 0.00000 | 0.00000 | 0.00000 | 0.00000 | 0.00000 | 0.00000 | 0.00000 |
| 31-010 | 0.00000 | 0.00000 | 0.00000 | 0.00000 | 0.00000 | 0.16667 | 0.08333 | 0.50000 | 0.00000 | 0.00000 | 0.00000 | 0.00000 | 0.00000 | 0.00000 | 0.00000 | 0.00000 | 0.00000 | 0.00000 | 0.00000 | 0.00000 |
| 31-011 | 0.00000 | 0.40000 | 0.70000 | 0.33333 | 0.63636 | 0.50000 | 0.25000 | 0.83333 | 0.58333 | 0.12500 | 0.10000 | 0.10000 | 0.33333 | 0.11111 | 0.50000 | 0.00000 | 0.00000 | 0.00000 | 0.37500 | 0.33333 |
| 31-012 | 0.00000 | 0.00000 | 0.00000 | 0.00000 | 0.00000 | 0.00000 | 0.08333 | 0.00000 | 0.00000 | 0.00000 | 0.00000 | 0.00000 | 0.00000 | 0.00000 | 0.00000 | 0.00000 | 0.00000 | 0.00000 | 0.00000 | 0.16667 |
| 31-013 | 0.00000 | 0.00000 | 0.00000 | 0.00000 | 0.00000 | 0.00000 | 0.16667 | 0.00000 | 0.00000 | 0.12500 | 0.00000 | 0.00000 | 0.00000 | 0.00000 | 0.00000 | 0.00000 | 0.00000 | 0.00000 | 0.00000 | 0.33333 |
| 31-014 | 0.00000 | 0.00000 | 0.00000 | 0.00000 | 0.00000 | 0.00000 | 0.00000 | 0.00000 | 0.00000 | 0.00000 | 0.00000 | 0.00000 | 0.00000 | 0.00000 | 0.00000 | 0.00000 | 0.00000 | 0.00000 | 0.12500 | 0.16667 |
| 31-015 | 0.25000 | 0.00000 | 0.00000 | 0.00000 | 0.00000 | 0.16667 | 0.16667 | 0.00000 | 0.00000 | 0.12500 | 0.10000 | 0.00000 | 0.00000 | 0.00000 | 0.00000 | 0.00000 | 0.00000 | 0.00000 | 0.00000 | 0.00000 |
| 31-016 | 0.00000 | 0.00000 | 0.00000 | 0.00000 | 0.00000 | 0.33333 | 0.16667 | 0.00000 | 0.00000 | 0.12500 | 0.00000 | 0.00000 | 0.00000 | 0.00000 | 0.00000 | 0.00000 | 0.00000 | 0.00000 | 0.12500 | 0.33333 |
| 31-017 | 0.00000 | 0.00000 | 0.00000 | 0.00000 | 0.00000 | 0.16667 | 0.00000 | 0.00000 | 0.00000 | 0.00000 | 0.00000 | 0.00000 | 0.00000 | 0.00000 | 0.00000 | 0.00000 | 0.00000 | 0.00000 | 0.00000 | 0.16667 |
| 31-018 | 0.00000 | 0.00000 | 0.00000 | 0.00000 | 0.00000 | 0.16667 | 0.00000 | 0.00000 | 0.00000 | 0.00000 | 0.00000 | 0.00000 | 0.00000 | 0.00000 | 0.00000 | 0.00000 | 0.00000 | 0.00000 | 0.00000 | 0.00000 |
| 31-019 | 0.00000 | 0.00000 | 0.00000 | 0.00000 | 0.00000 | 0.00000 | 0.25000 | 0.00000 | 0.00000 | 0.12500 | 0.00000 | 0.00000 | 0.00000 | 0.00000 | 0.00000 | 0.00000 | 0.00000 | 0.00000 | 0.00000 | 0.33333 |
| 31-020 | 0.00000 | 0.00000 | 0.00000 | 0.00000 | 0.00000 | 0.00000 | 0.16667 | 0.00000 | 0.00000 | 0.12500 | 0.00000 | 0.00000 | 0.00000 | 0.00000 | 0.00000 | 0.00000 | 0.00000 | 0.00000 | 0.00000 | 0.00000 |
| 31-021 | 0.00000 | 0.00000 | 0.00000 | 0.00000 | 0.00000 | 0.00000 | 0.00000 | 0.16667 | 0.08333 | 0.00000 | 0.00000 | 0.00000 | 0.00000 | 0.00000 | 0.00000 | 0.00000 | 0.00000 | 0.00000 | 0.00000 | 0.00000 |
| 31-022 | 0.00000 | 0.50000 | 0.50000 | 0.44444 | 0.72727 | 0.83333 | 0.33333 | 1.00000 | 0.83333 | 0.12500 | 0.50000 | 0.20000 | 0.00000 | 0.11111 | 0.00000 | 0.00000 | 0.16667 | 0.00000 | 0.37500 | 0.33333 |
| 31-023 | 0.00000 | 0.00000 | 0.10000 | 0.00000 | 0.00000 | 0.33333 | 0.08333 | 0.00000 | 0.00000 | 0.00000 | 0.00000 | 0.00000 | 0.00000 | 0.00000 | 0.12500 | 0.14286 | 0.16667 | 0.00000 | 0.00000 | 0.00000 |
| 31-024 | 0.00000 | 0.40000 | 0.40000 | 0.22222 | 0.45455 | 0.50000 | 0.25000 | 0.83333 | 0.66667 | 0.00000 | 0.20000 | 0.00000 | 0.00000 | 0.11111 | 0.12500 | 0.00000 | 0.16667 | 0.00000 | 0.00000 | 0.16667 |
| 31-025 | 0.00000 | 0.00000 | 0.00000 | 0.00000 | 0.09091 | 0.00000 | 0.00000 | 0.00000 | 0.08333 | 0.00000 | 0.00000 | 0.00000 | 0.00000 | 0.00000 | 0.00000 | 0.00000 | 0.00000 | 0.00000 | 0.00000 | 0.16667 |
| 31-026 | 0.00000 | 0.00000 | 0.00000 | 0.00000 | 0.00000 | 0.16667 | 0.00000 | 0.00000 | 0.00000 | 0.00000 | 0.00000 | 0.00000 | 0.00000 | 0.00000 | 0.00000 | 0.00000 | 0.00000 | 0.00000 | 0.00000 | 0.00000 |
| 31-027 | 0.00000 | 0.00000 | 0.00000 | 0.00000 | 0.00000 | 0.33333 | 0.08333 | 0.00000 | 0.00000 | 0.00000 | 0.00000 | 0.00000 | 0.00000 | 0.00000 | 0.00000 | 0.00000 | 0.00000 | 0.00000 | 0.12500 | 0.00000 |
| 31-028 | 0.00000 | 0.00000 | 0.00000 | 0.00000 | 0.00000 | 0.00000 | 0.00000 | 0.00000 | 0.00000 | 0.00000 | 0.00000 | 0.00000 | 0.00000 | 0.00000 | 0.00000 | 0.00000 | 0.00000 | 0.00000 | 0.00000 | 0.16667 |
| 31-029 | 0.00000 | 0.00000 | 0.00000 | 0.00000 | 0.00000 | 0.00000 | 0.00000 | 0.00000 | 0.00000 | 0.00000 | 0.00000 | 0.00000 | 0.00000 | 0.00000 | 0.00000 | 0.00000 | 0.00000 | 0.00000 | 0.00000 | 0.33333 |
| 31-030 | 0.00000 | 0.00000 | 0.00000 | 0.00000 | 0.00000 | 0.16667 | 0.25000 | 0.00000 | 0.00000 | 0.00000 | 0.00000 | 0.00000 | 0.00000 | 0.00000 | 0.00000 | 0.00000 | 0.00000 | 0.00000 | 0.00000 | 0.16667 |
| 31-031 | 0.00000 | 0.00000 | 0.00000 | 0.00000 | 0.00000 | 0.00000 | 0.41667 | 0.00000 | 0.00000 | 0.12500 | 0.00000 | 0.00000 | 0.00000 | 0.00000 | 0.00000 | 0.00000 | 0.00000 | 0.00000 | 0.00000 | 0.00000 |
| 31-032 | 0.00000 | 0.00000 | 0.00000 | 0.00000 | 0.00000 | 0.00000 | 0.16667 | 0.00000 | 0.00000 | 0.12500 | 0.00000 | 0.00000 | 0.00000 | 0.00000 | 0.00000 | 0.00000 | 0.00000 | 0.00000 | 0.00000 | 0.33333 |
| 31-033 | 0.00000 | 0.00000 | 0.00000 | 0.00000 | 0.00000 | 0.00000 | 0.16667 | 0.00000 | 0.00000 | 0.12500 | 0.00000 | 0.00000 | 0.00000 | 0.00000 | 0.00000 | 0.00000 | 0.00000 | 0.00000 | 0.00000 | 0.33333 |
| 31-034 | 0.00000 | 0.00000 | 0.00000 | 0.00000 | 0.00000 | 0.00000 | 0.00000 | 0.00000 | 0.00000 | 0.00000 | 0.00000 | 0.00000 | 0.00000 | 0.00000 | 0.00000 | 0.00000 | 0.00000 | 0.00000 | 0.00000 | 0.00000 |
| 31-035 | 0.00000 | 0.00000 | 0.00000 | 0.00000 | 0.00000 | 0.00000 | 0.00000 | 0.00000 | 0.00000 | 0.00000 | 0.00000 | 0.00000 | 0.00000 | 0.00000 | 0.00000 | 0.00000 | 0.00000 | 0.00000 | 0.00000 | 0.16667 |
| 31-036 | 0.00000 | 0.00000 | 0.00000 | 0.00000 | 0.00000 | 0.16667 | 0.00000 | 0.00000 | 0.00000 | 0.00000 | 0.00000 | 0.00000 | 0.00000 | 0.00000 | 0.00000 | 0.00000 | 0.00000 | 0.00000 | 0.25000 | 0.16667 |
| 31-037 | 0.00000 | 0.00000 | 0.00000 | 0.00000 | 0.00000 | 0.00000 | 0.00000 | 0.00000 | 0.00000 | 0.00000 | 0.00000 | 0.00000 | 0.00000 | 0.00000 | 0.00000 | 0.00000 | 0.00000 | 0.00000 | 0.00000 | 0.16667 |
| 31-038 | 0.00000 | 0.00000 | 0.00000 | 0.00000 | 0.00000 | 0.16667 | 0.00000 | 0.00000 | 0.00000 | 0.00000 | 0.00000 | 0.00000 | 0.00000 | 0.00000 | 0.00000 | 0.00000 | 0.00000 | 0.00000 | 0.00000 | 0.00000 |
| 31-039 | 0.00000 | 0.00000 | 0.20000 | 0.00000 | 0.09091 | 0.00000 | 0.16667 | 0.00000 | 0.08333 | 0.00000 | 0.00000 | 0.00000 | 0.00000 | 0.00000 | 0.00000 | 0.00000 | 0.00000 | 0.00000 | 0.12500 | 0.16667 |
| 31-040 | 0.00000 | 0.00000 | 0.40000 | 0.00000 | 0.27273 | 0.66667 | 0.25000 | 0.66667 | 0.66667 | 0.12500 | 0.00000 | 0.20000 | 0.66667 | 0.22222 | 0.37500 | 0.00000 | 0.16667 | 0.16667 | 0.37500 | 0.33333 |
| 31-041 | 0.50000 | 0.20000 | 0.70000 | 0.33333 | 0.18182 | 0.66667 | 0.25000 | 0.83333 | 0.83333 | 0.37500 | 0.10000 | 0.40000 | 1.00000 | 0.88889 | 0.75000 | 0.71429 | 0.50000 | 0.50000 | 0.75000 | 0.33333 |
| 31-042 | 0.50000 | 0.60000 | 0.50000 | 0.44444 | 0.18182 | 0.16667 | 0.16667 | 0.33333 | 0.33333 | 0.62500 | 0.30000 | 0.70000 | 0.22222 | 0.66667 | 0.62500 | 0.71429 | 0.33333 | 0.50000 | 0.50000 | 0.33333 |
| 31-043 | 0.00000 | 0.00000 | 0.00000 | 0.00000 | 0.00000 | 0.00000 | 0.25000 | 0.00000 | 0.00000 | 0.12500 | 0.00000 | 0.00000 | 0.00000 | 0.00000 | 0.00000 | 0.00000 | 0.00000 | 0.00000 | 0.00000 | 0.00000 |
| 31-044 | 0.00000 | 0.00000 | 0.00000 | 0.00000 | 0.00000 | 0.16667 | 0.25000 | 0.16667 | 0.00000 | 0.00000 | 0.00000 | 0.10000 | 0.11111 | 0.00000 | 0.00000 | 0.14286 | 0.00000 | 0.00000 | 0.12500 | 0.00000 |
| 31-045 | 0.00000 | 0.00000 | 0.00000 | 0.00000 | 0.00000 | 0.16667 | 0.08333 | 0.16667 | 0.08333 | 0.12500 | 0.30000 | 0.00000 | 0.11111 | 0.00000 | 0.00000 | 0.00000 | 0.00000 | 0.16667 | 0.25000 | 0.16667 |
| 31-046 | 0.00000 | 0.00000 | 0.00000 | 0.00000 | 0.00000 | 0.16667 | 0.00000 | 0.00000 | 0.25000 | 0.00000 | 0.30000 | 0.20000 | 0.00000 | 0.00000 | 0.00000 | 0.14286 | 0.00000 | 0.16667 | 0.25000 | 0.33333 |
| 31-047 | 0.00000 | 0.00000 | 0.00000 | 0.00000 | 0.00000 | 0.16667 | 0.00000 | 0.16667 | 0.16667 | 0.00000 | 0.00000 | 0.00000 | 0.00000 | 0.00000 | 0.00000 | 0.28571 | 0.16667 | 0.00000 | 0.00000 | 0.00000 |
| 31-048 | 0.00000 | 0.00000 | 0.00000 | 0.00000 | 0.00000 | 0.00000 | 0.00000 | 0.00000 | 0.00000 | 0.00000 | 0.00000 | 0.00000 | 0.00000 | 0.00000 | 0.00000 | 0.14286 | 0.00000 | 0.00000 | 0.00000 | 0.00000 |
| 31-049 | 0.75000 | 0.00000 | 0.00000 | 0.00000 | 0.00000 | 0.00000 | 0.00000 | 0.00000 | 0.00000 | 0.00000 | 0.00000 | 0.00000 | 0.00000 | 0.00000 | 0.00000 | 0.00000 | 0.00000 | 0.00000 | 0.00000 | 0.00000 |
| 31-050 | 0.50000 | 0.00000 | 0.00000 | 0.00000 | 0.00000 | 0.00000 | 0.00000 | 0.00000 | 0.00000 | 0.00000 | 0.00000 | 0.00000 | 0.00000 | 0.00000 | 0.00000 | 0.00000 | 0.00000 | 0.00000 | 0.00000 | 0.00000 |
| 31-051 | 0.00000 | 0.00000 | 0.00000 | 0.00000 | 0.00000 | 0.00000 | 0.00000 | 0.00000 | 0.00000 | 0.00000 | 0.00000 | 0.00000 | 0.00000 | 0.00000 | 0.00000 | 0.00000 | 0.00000 | 0.00000 | 0.00000 | 0.16667 |
| 31-052 | 0.00000 | 0.00000 | 0.00000 | 0.00000 | 0.00000 | 0.00000 | 0.16667 | 0.00000 | 0.00000 | 0.12500 | 0.00000 | 0.00000 | 0.00000 | 0.00000 | 0.00000 | 0.00000 | 0.16667 | 0.00000 | 0.00000 | 0.16667 |
| 31-053 | 0.00000 | 0.00000 | 0.00000 | 0.00000 | 0.00000 | 0.00000 | 0.16667 | 0.00000 | 0.00000 | 0.12500 | 0.00000 | 0.00000 | 0.00000 | 0.00000 | 0.00000 | 0.00000 | 0.00000 | 0.00000 | 0.00000 | 0.00000 |
| 31-054 | 0.00000 | 0.00000 | 0.00000 | 0.00000 | 0.09091 | 0.16667 | 0.08333 | 0.33333 | 0.50000 | 0.00000 | 0.30000 | 0.00000 | 0.00000 | 0.00000 | 0.12500 | 0.14286 | 0.00000 | 0.00000 | 0.00000 | 0.00000 |
| 31-055 | 0.00000 | 0.00000 | 0.00000 | 0.00000 | 0.00000 | 0.00000 | 0.08333 | 0.00000 | 0.16667 | 0.00000 | 0.00000 | 0.00000 | 0.00000 | 0.00000 | 0.00000 | 0.00000 | 0.00000 | 0.00000 | 0.00000 | 0.00000 |
| 31-056 | 0.00000 | 0.00000 | 0.00000 | 0.00000 | 0.00000 | 0.00000 | 0.16667 | 0.16667 | 0.00000 | 0.00000 | 0.00000 | 0.00000 | 0.00000 | 0.00000 | 0.00000 | 0.00000 | 0.00000 | 0.00000 | 0.00000 | 0.00000 |
| 31-057 | 0.00000 | 0.00000 | 0.00000 | 0.00000 | 0.00000 | 0.00000 | 0.08333 | 0.00000 | 0.00000 | 0.12500 | 0.00000 | 0.00000 | 0.00000 | 0.00000 | 0.00000 | 0.00000 | 0.00000 | 0.00000 | 0.00000 | 0.00000 |
| 31-058 | 0.00000 | 0.00000 | 0.00000 | 0.00000 | 0.00000 | 0.00000 | 0.08333 | 0.00000 | 0.00000 | 0.00000 | 0.00000 | 0.00000 | 0.00000 | 0.00000 | 0.00000 | 0.00000 | 0.00000 | 0.00000 | 0.00000 | 0.00000 |
| 31-059 | 0.00000 | 0.00000 | 0.00000 | 0.00000 | 0.00000 | 0.00000 | 0.08333 | 0.66667 | 0.41667 | 0.00000 | 0.00000 | 0.00000 | 0.00000 | 0.00000 | 0.00000 | 0.00000 | 0.00000 | 0.00000 | 0.00000 | 0.00000 |
| 31-060 | 0.00000 | 0.00000 | 0.00000 | 0.00000 | 0.00000 | 0.00000 | 0.08333 | 0.33333 | 0.33333 | 0.00000 | 0.00000 | 0.00000 | 0.00000 | 0.00000 | 0.00000 | 0.00000 | 0.00000 | 0.00000 | 0.00000 | 0.00000 |

**Table S2 | Nineteen environmental variables used in this study.**

| Temperature  (period 1950-2000) | Bio1: Annual mean temperature ( ℃×10) |
| --- | --- |
|  | Bio2: Mean diurnal range (Mean of monthly (max temp - min temp)) |
|  | Bio3: Isothermality (Bio2/Bio7) (×100) |
|  | Bio4: Temperature seasonality (standard deviation ×100) |
|  | Bio5: Max temperature of warmest month ( ℃×10) |
|  | Bio6: Min temperature of coldest month ( ℃×10) |
|  | Bio7: Temperature annual range (E5-E6) |
|  | Bio8: Mean temperature of wettest quarter ( ℃×10) |
|  | Bio9: Mean temperature of driest quarter ( ℃×10) |
|  | Bio10: Mean temperature of warmest quarter ( ℃×10) |
|  | Bio11: Mean temperature of coldest quarter ( ℃×10) |
| Precipitation  (period 1950-2000) | Bio12: Annual precipitation (mm) |
|  | Bio13: Precipitation of wettest month (mm) |
|  | Bio14: Precipitation of driest month (mm) |
|  | Bio15: Precipitation seasonality (coefficient of variation) |
|  | Bio16: Precipitation of wettest quarter (mm) |
|  | Bio17: Precipitation of driest quarter (mm) |
|  | Bio18: Precipitation of warmest quarter (mm) |
|  | Bio19: Precipitation of coldest quarter (mm) |

**Table S3 | Environmental variables for each location from the WorldClim database.**

| **Population no. and code** | **Environmental variables** | | | | | | | | | | | | | | | | | | |
| --- | --- | --- | --- | --- | --- | --- | --- | --- | --- | --- | --- | --- | --- | --- | --- | --- | --- | --- | --- |
|  | **Bio1** | **Bio2** | **Bio3** | **Bio4** | **Bio5** | **Bio6** | **Bio7** | **Bio8** | **Bio9** | **Bio10** | **Bio11** | **Bio12** | **Bio13** | **Bio14** | **Bio15** | **Bio16** | **Bio17** | **Bio18** | **Bio19** |
| 1.SXWT | 1.0 | 11.7 | 29.1 | 1069.0 | 19.9 | -20.1 | 40 | 13.7 | -12.8 | 13.7 | -12.8 | 591 | 158 | 5 | 102.9 | 370 | 23 | 370 | 23 |
| 2.SDYM | 12.8 | 11.5 | 29.3 | 1062.0 | 31.2 | -7.9 | 39.1 | 25.3 | -1.0 | 25.3 | -1.0 | 689 | 217 | 8 | 113.1 | 450 | 28 | 450 | 28 |
| 3.SDTM | 9.1 | 8.7 | 24.8 | 1002.6 | 25.6 | -9.4 | 35 | 21.0 | -3.9 | 21.0 | -3.9 | 889 | 268 | 11 | 110.0 | 575 | 36 | 575 | 36 |
| 4.SDMM | 13.3 | 10.7 | 28.8 | 1032.2 | 31 | -6.3 | 37.3 | 25.6 | -0.1 | 25.6 | -0.1 | 769 | 232 | 9 | 107.3 | 491 | 34 | 491 | 34 |
| 5.SDBD | 13.1 | 10.2 | 28.1 | 1012.9 | 30.3 | -5.9 | 36.2 | 25.2 | 0.0 | 25.2 | 0.0 | 791 | 224 | 11 | 101.3 | 494 | 41 | 494 | 41 |
| 6.SXTL | 10.2 | 13.6 | 31.6 | 1084.4 | 30.2 | -12.8 | 43 | 21.4 | -3.9 | 23.0 | -3.9 | 433 | 115 | 4 | 104.6 | 273 | 13 | 264 | 13 |
| 7.SXLK | 9.6 | 13.1 | 31.6 | 1032.2 | 28.8 | -12.6 | 41.4 | 20.1 | -4.2 | 21.7 | -4.2 | 575 | 149 | 5 | 102.0 | 353 | 18 | 350 | 18 |
| 8.SXBT | 9.8 | 12.8 | 31.3 | 1021.6 | 28.9 | -12.1 | 41 | 20.4 | -3.6 | 21.9 | -3.6 | 537 | 118 | 3 | 94.2 | 320 | 12 | 298 | 12 |
| 9.HBWZ | 11.4 | 12.7 | 31.3 | 1043.7 | 30.2 | -10.2 | 40.4 | 21.9 | -2.3 | 23.8 | -2.3 | 603 | 172 | 4 | 108.7 | 389 | 15 | 367 | 15 |
| 10.HNJL | 11.2 | 12.0 | 30.5 | 1013.5 | 29.6 | -9.6 | 39.2 | 21.6 | -2.2 | 23.3 | -2.2 | 655 | 170 | 7 | 97.2 | 388 | 24 | 386 | 24 |
| 11.SXHM | 10.0 | 10.3 | 27.5 | 996.7 | 27.7 | -9.8 | 37.5 | 20.4 | -2.9 | 22.0 | -2.9 | 567 | 118 | 5 | 84.5 | 314 | 17 | 289 | 17 |
| 12.SXWL | 10.1 | 9.4 | 26.5 | 963.6 | 27 | -8.6 | 35.6 | 20.3 | -2.3 | 21.8 | -2.3 | 631 | 128 | 8 | 77.4 | 329 | 26 | 304 | 26 |
| 13.HNSM | 11.9 | 11.3 | 30.5 | 976.0 | 29.4 | -7.7 | 37.1 | 22.1 | -0.7 | 23.6 | -0.7 | 724 | 164 | 10 | 80.7 | 387 | 36 | 373 | 36 |
| 14.SXLJ | 10.3 | 9.0 | 26.2 | 926.0 | 26.8 | -7.6 | 34.4 | 20.1 | -1.6 | 21.6 | -1.6 | 726 | 143 | 9 | 73.4 | 364 | 31 | 335 | 31 |
| 15.HNLJ | 7.8 | 9.1 | 27.8 | 870.8 | 23.4 | -9.4 | 32.8 | 17.2 | -3.4 | 18.4 | -3.4 | 900 | 180 | 13 | 72.4 | 446 | 45 | 424 | 45 |
| 16.HNLY | 8.7 | 9.8 | 28.9 | 889.0 | 24.8 | -9 | 33.8 | 18.3 | -2.7 | 19.6 | -2.7 | 885 | 182 | 14 | 73.3 | 443 | 47 | 425 | 47 |
| 17.HBWD | 11.9 | 9.7 | 29.4 | 854.2 | 27.8 | -5.1 | 32.9 | 21.3 | 1.1 | 22.4 | 1.1 | 970 | 173 | 16 | 65.8 | 448 | 55 | 431 | 55 |
| 18.HNTB | 15.0 | 9.6 | 28.1 | 920.9 | 31.3 | -2.8 | 34.1 | 26.3 | 3.3 | 26.3 | 3.3 | 883 | 175 | 15 | 68.8 | 423 | 57 | 423 | 57 |
| 19.HNJG | 14.4 | 8.5 | 25.8 | 915.3 | 30.2 | -2.7 | 32.9 | 25.5 | 2.7 | 25.5 | 2.7 | 1168 | 213 | 24 | 60.0 | 524 | 101 | 524 | 101 |
| 20.HBDH | 15.0 | 8.5 | 26.2 | 898.9 | 30.7 | -1.9 | 32.6 | 25.9 | 3.5 | 25.9 | 3.5 | 1005 | 184 | 22 | 60.5 | 447 | 82 | 447 | 82 |

**Table s4 | The outlier loci identified by BayeScan and FDIST2.**

|  | **BayeScan** | | **FDIST2** | |
| --- | --- | --- | --- | --- |
| **Locus** | **Posterior probability** | **log10(PO)** | **Observe F_ST_** | **F_ST_ P-value** |
| 4-015 |  |  | 0.010 | 0.022 |
| 4-023 |  |  | 0.392 | 0.015 |
| 4-025 |  |  | 0.340 | 0.030 |
| 4-026 | 0.989 | 1.938 | 0.326 | 0.028 |
| 4-029 |  |  | -0.020 | 0.007 |
| 4-037 | 0.910 | 1.007 |  |  |
| 4-038 | 0.963 | 1.415 |  |  |
| 4-039 | 0.988 | 1.901 | 0.329 | 0.030 |
| 4-044 |  |  | 0.329 | 0.037 |
| 4-046 | 0.825 | 0.673 | 0.309 | 0.044 |
| 4-094 |  |  | 0.020 | 0.044 |
| 4-117 | 0.975 | 1.591 | 0.332 | 0.029 |
| 4-118 | 1.000 | 1000 | 0.352 | 0.014 |
| 5-001 | 0.871 | 0.830 |  |  |
| 5-013 |  |  | -0.024 | 0.009 |
| 5-018 | 1.000 | 1000 | 0.419 | 0.005 |
| 5-021 | 0.991 | 2.052 |  |  |
| 5-029 |  |  | -0.014 | 0.020 |
| 5-062 | 0.978 | 1.656 |  |  |
| 5-083 | 0.991 | 2.062 | 0.278 | 0.044 |
| 5-088 |  |  | 0.369 | 0.019 |
| 5-104 |  |  | 0.009 | 0.020 |
| 5-106 |  |  | 0.365 | 0.022 |
| 5-109 | 0.974 | 1.567 |  |  |
| 5-110 | 0.986 | 1.848 |  |  |
| 5-120 |  |  | 0.468 | 0.005 |
| 6-022 |  |  | 0.000 | 0.000 |
| 6-038 |  |  | -0.019 | 0.009 |
| 6-049 |  |  | 0.009 | 0.018 |
| 6-065 |  |  | 0.023 | 0.037 |
| 6-069 |  |  | -0.009 | 0.021 |
| 6-087 |  |  | -0.001 | 0.038 |
| 6-088 |  |  | 0.027 | 0.048 |
| 6-089 |  |  | -0.007 | 0.014 |
| 9-003 |  |  | 0.007 | 0.012 |
| 9-017 |  |  | -0.019 | 0.016 |
| 9-018 |  |  | 0.020 | 0.045 |
| 9-052 |  |  | 0.023 | 0.048 |
| 9-053 |  |  | 0.023 | 0.047 |
| 9-077 | 0.994 | 2.234 |  |  |
| 9-078 |  |  | 0.005 | 0.006 |
| 9-079 |  |  | 0.007 | 0.008 |
| 9-080 | 0.997 | 2.522 |  |  |
| 9-084 |  |  | 0.017 | 0.037 |
| 9-085 |  |  | -0.020 | 0.006 |
| 9-086 |  |  | -0.008 | 0.015 |
| 9-087 |  |  | -0.002 | 0.018 |
| 9-091 |  |  | 0.023 | 0.032 |
| 9-110 |  |  | -0.007 | 0.027 |
| 12-009 |  |  | 0.009 | 0.019 |
| 12-013 |  |  | 0.002 | 0.003 |
| 12-027 |  |  | 0.004 | 0.006 |
| 12-030 |  |  | -0.015 | 0.018 |
| 12-034 | 1.000 | 1000 |  |  |
| 12-041 |  |  | 0.011 | 0.023 |
| 12-100 |  |  | -0.007 | 0.029 |
| 14-001 |  |  | 0.014 | 0.031 |
| 14-003 |  |  | -0.011 | 0.020 |
| 14-013 |  |  | -0.002 | 0.022 |
| 14-014 |  |  | -0.017 | 0.010 |
| 14-015 |  |  | -0.037 | 0.002 |
| 14-020 |  |  | -0.006 | 0.013 |
| 14-030 |  |  | 0.009 | 0.011 |
| 14-034 |  |  | -0.005 | 0.013 |
| 14-036 |  |  | 0.003 | 0.006 |
| 14-038 |  |  | -0.025 | 0.004 |
| 14-048 |  |  | -0.014 | 0.012 |
| 14-050 |  |  | 0.011 | 0.014 |
| 14-060 |  |  | -0.017 | 0.009 |
| 14-061 |  |  | 0.012 | 0.028 |
| 14-062 |  |  | 0.023 | 0.049 |
| 14-066 |  |  | 0.012 | 0.018 |
| 14-067 |  |  | -0.002 | 0.030 |
| 14-070 |  |  | 0.008 | 0.012 |
| 14-078 |  |  | 0.007 | 0.007 |
| 14-084 |  |  | -0.033 | 0.005 |
| 14-097 |  |  | -0.022 | 0.010 |
| 14-107 |  |  | 0.009 | 0.016 |
| 14-118 | 0.862 | 0.796 |  |  |
| 17-011 |  |  | 0.014 | 0.028 |
| 17-016 |  |  | 0.003 | 0.007 |
| 17-019 |  |  | -0.001 | 0.032 |
| 17-020 |  |  | -0.004 | 0.037 |
| 17-024 | 0.796 | 0.590 |  |  |
| 17-026 |  |  | 0.004 | 0.009 |
| 17-027 |  |  | 0.025 | 0.044 |
| 17-028 |  |  | -0.011 | 0.012 |
| 17-029 |  |  | -0.034 | 0.006 |
| 17-030 |  |  | -0.001 | 0.026 |
| 17-031 |  |  | -0.038 | 0.003 |
| 17-038 |  |  | 0.010 | 0.011 |
| 17-040 |  |  | -0.028 | 0.009 |
| 17-043 |  |  | 0.008 | 0.018 |
| 17-045 |  |  | 0.014 | 0.034 |
| 17-049 | 0.926 | 1.096 |  |  |
| 17-056 |  |  | -0.010 | 0.021 |
| 17-057 |  |  | -0.007 | 0.021 |
| 17-063 |  |  | 0.006 | 0.013 |
| 17-069 |  |  | 0.015 | 0.020 |
| 17-082 | 0.886 | 0.892 |  |  |
| 17-098 |  |  | 0.021 | 0.046 |
| 17-104 | 0.931 | 1.130 |  |  |
| 18-004 |  |  | -0.005 | 0.016 |
| 18-007 |  |  | 0.011 | 0.026 |
| 18-010 |  |  | -0.032 | 0.004 |
| 18-011 |  |  | -0.038 | 0.002 |
| 18-012 |  |  | 0.030 | 0.048 |
| 18-017 |  |  | 0.002 | 0.005 |
| 18-022 |  |  | 0.325 | 0.040 |
| 18-027 |  |  | 0.021 | 0.039 |
| 18-028 |  |  | 0.028 | 0.043 |
| 18-030 |  |  | 0.006 | 0.009 |
| 18-031 |  |  | 0.004 | 0.005 |
| 18-033 |  |  | -0.014 | 0.011 |
| 18-037 | 0.794 | 0.585 | 0.535 | 0.002 |
| 18-039 |  |  | 0.374 | 0.021 |
| 18-042 |  |  | -0.019 | 0.006 |
| 18-051 | 0.824 | 0.671 |  |  |
| 18-060 |  |  | 0.367 | 0.023 |
| 18-062 |  |  | 0.018 | 0.043 |
| 18-078 |  |  | -0.001 | 0.039 |
| 18-079 |  |  | -0.013 | 0.017 |
| 18-084 |  |  | -0.022 | 0.011 |
| 18-087 |  |  | 0.014 | 0.028 |
| 21-004 | 1.000 | 1000 | 0.384 | 0.014 |
| 21-005 | 1.000 | 1000 | 0.386 | 0.012 |
| 21-008 | 0.921 | 1.065 |  |  |
| 21-009 | 1.000 | 1000 | 0.407 | 0.005 |
| 21-011 | 0.888 | 0.900 |  |  |
| 21-020 | 0.882 | 0.873 |  |  |
| 21-021 | 0.932 | 1.140 |  |  |
| 21-023 | 0.922 | 1.075 |  |  |
| 21-024 |  |  | 0.004 | 0.007 |
| 21-030 |  |  | -0.001 | 0.033 |
| 21-045 | 1.000 | 1000 | 0.340 | 0.018 |
| 21-046 | 0.996 | 2.442 | 0.313 | 0.036 |
| 21-061 | 1.000 | 1000 | 0.414 | 0.003 |
| 21-063 | 0.980 | 1.686 |  |  |
| 21-065 |  |  | 0.017 | 0.041 |
| 21-067 | 0.975 | 1.598 |  |  |
| 21-073 | 0.971 | 1.522 | 0.560 | 0.000 |
| 21-075 |  |  | -0.023 | 0.011 |
| 21-076 | 1.000 | 1000 | 0.595 | 0.000 |
| 21-077 | 1.000 | 1000 | 0.478 | 0.009 |
| 21-081 | 0.990 | 2.014 | 0.329 | 0.030 |
| 21-082 | 0.990 | 1.996 |  |  |
| 21-083 | 0.956 | 1.333 |  |  |
| 21-084 | 0.999 | 3.000 |  |  |
| 21-085 | 0.985 | 1.829 |  |  |
| 21-091 | 1.000 | 1000 | 0.346 | 0.022 |
| 21-092 | 0.999 | 2.853 |  |  |
| 21-094 | 0.878 | 0.856 |  |  |
| 21-097 | 0.999 | 3.097 |  |  |
| 21-108 | 0.894 | 0.927 |  |  |
| 21-109 | 0.863 | 0.800 |  |  |
| 21-112 | 0.798 | 0.595 |  |  |
| 21-115 | 1.000 | 1000 | 0.536 | 0.025 |
| 21-116 | 1.000 | 1000 | 0.565 | 0.007 |
| 21-124 | 1.000 | 1000 | 0.312 | 0.029 |
| 21-125 | 1.000 | 1000 |  |  |
| 21-126 | 1.000 | 1000 |  |  |
| 21-136 | 1.000 | 1000 | 0.382 | 0.005 |
| 21-142 | 1.000 | 1000 | 0.294 | 0.034 |
| 21-143 | 0.998 | 2.657 | 0.311 | 0.042 |
| 21-144 | 0.997 | 2.467 |  |  |
| 31-022 | 0.995 | 2.335 | 0.332 | 0.017 |
| 31-041 | 0.923 | 1.079 |  |  |
| 31-044 |  |  | 0.017 | 0.041 |
| 31-059 |  |  | 0.404 | 0.011 |
